# Cationic Aluminium Complexes as Catalysts for Imine Hydrogenation

**DOI:** 10.1002/chem.202100641

**Published:** 2021-05-02

**Authors:** Alexander Friedrich, Jonathan Eyselein, Holger Elsen, Jens Langer, Jürgen Pahl, Michael Wiesinger, Sjoerd Harder

**Affiliations:** ^1^ Inorganic and Organometallic Chemistry Friedrich-Alexander-Universität Erlangen-Nürnberg Egerlandstraße 1 91058 Erlangen Germany

**Keywords:** Aluminium, cations, imine, hydrogenation, DFT

## Abstract

Strongly Lewis acidic cationic aluminium complexes, stabilized by β–diketiminate (BDI) ligands and free of Lewis bases, have been prepared as their B(C_6_F_5_)_4_^−^ salts and were investigated for catalytic activity in imine hydrogenation. The backbone (R1) and N (R2) substituents on the ^R1,R2^BDI ligand (^R1,R2^BDI=HC[C(R1)N(R2)]_2_) influence sterics and Lewis acidity. Ligand bulk increases along the row ^Me,DIPP^BDI<^Me,DIPeP^BDI≈^*t*Bu,DIPP^BDI<^*t*Bu,DIPeP^BDI; DIPP=2,6‐C(H)Me_2_‐phenyl, DIPeP=2,6‐C(H)Et_2_‐phenyl. The Gutmann‐Beckett test showed acceptor numbers of: (^*t*Bu,DIPP^BDI)AlMe^+^ 85.6, (^*t*Bu,DIPeP^BDI)AlMe^+^ 85.9, (^Me,DIPP^BDI)AlMe^+^ 89.7, (^Me,DIPeP^BDI)AlMe^+^ 90.8, (^Me,DIPP^BDI)AlH^+^ 95.3. Steric and electronic factors need to be balanced for catalytic activity in imine hydrogenation. Open, highly Lewis acidic, cations strongly coordinate imine rendering it inactive as a Frustrated Lewis Pair (FLP). The bulkiest cations do not coordinate imine but its combination is also not an active catalyst. The cation (^*t*Bu,DIPP^BDI)AlMe^+^ shows the best catalytic activity for various imines and is also an active catalyst for the Tishchenko reaction of benzaldehyde to benzylbenzoate. DFT calculations on the mechanism of imine hydrogenation catalysed by cationic Al complexes reveal two interconnected catalytic cycles operating in concert. Hydrogen is activated either by FLP reactivity of an Al⋅⋅⋅imine couple or, after formation of significant quantities of amine, by reaction with an Al⋅⋅⋅amine couple. The latter autocatalytic Al⋅⋅⋅amine cycle is energetically favoured.

## Graphical abstract

**A series of strongly Lewis acidic** cationic aluminium complexes, stabilized by β–diketiminate (BDI) ligands and free of Lewis bases, have been prepared as their B(C_6_F_5_)_4_^−^ salts. Their catalytic activity in imine hydrogenation depends strongly on the substituents in the BDI ligand. DFT calculations on the mechanism reveal two interconnected catalytic cycles. Hydrogen is activated either by Al⋅⋅⋅imine or Al⋅⋅⋅amine Frustrated‐Lewis‐Pairs.

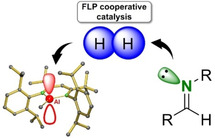

## Introduction

**Scheme 1:**
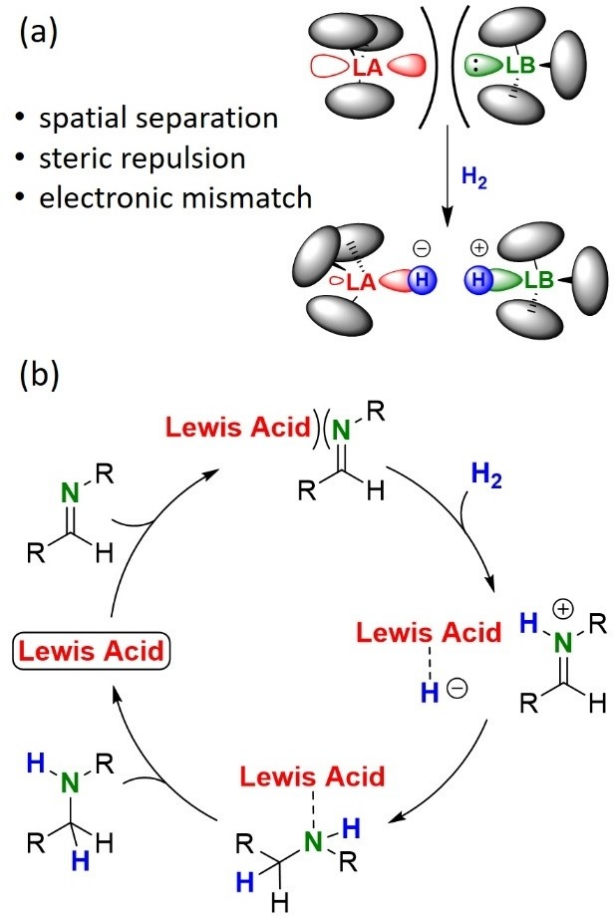
(a) Activation of H_2_ with a sterically congested Lewis acid (LA) / Lewis base (LB) Frustrated Lewis Pair. (b) General catalytic cycle for imine hydrogenation by a Lewis acid.

Lewis acids are frequently used as highly robust catalysts in many industrial applications.^[1]^ Especially solid Lewis acids that can be used under harsh conditions have proven to be powerful heterogeneous catalysts in the oil refining industry. In strong contrast, the combination of bulky molecular Lewis acids and bases has been shown to break bonds under much milder conditions (Scheme 1a).^[2]^ Preventing the formation of a Lewis acid/base pair has led to highly active mixtures that show reactivities akin to that of transition metal complexes. This rapidly growing field of Frustrated Lewis Pair (FLP) chemistry developed from stoichiometric molecule activation to catalytic transformation.[^3^, ^4^, ^5^, ^6^] First applications of FLP's in catalysis involved the reduction of imines with H_2_ (Scheme 1b).^[7]^ It was soon realized that, provided the imine substrate is bulky, only the Lewis acidic FLP component is needed.[^8^, ^9^] Indeed, the single action of B(C_6_F_5_)_3_, which is the standard Lewis acid in FLP chemistry, suffices to catalyse imine hydrogenation (Scheme 1). Although the field of FLP catalysis rapidly evolved to hydrogenation of numerous unsaturated substrates,^[6]^ most investigations on imine hydrogenation are limited to variation of substituent patterns on the borane Lewis acid catalyst.[^10^, ^11^, ^12^, ^13^]

Our group has been interested in molecule activation with highly Lewis acidic cationic main group metal complexes.[^14^, ^15^, ^16^, ^17^, ^18^, ^19^] We recently reported that Jordan's cationic β‐diketiminate aluminium complex, (^Me,DIPP^BDI)AlMe^+^, can be used as a highly Lewis acidic component in FLP activation of alkynes or CO_2_.^[20]^ Attempts to convert these stoichiometric reactions to a catalytic protocol failed. After the first demonstration of the high reactivity of Al‐based FLP's,^[21]^ the field has been enormously expanded.[^22^, ^23^, ^24^, ^25^, ^26^] By far most of these FLP applications concern stoichiometric molecule activation and only a limited number of catalytic procedures partially focused on FLP polymerization have been reported.[^27^, ^28^, ^29^, ^30^] This is inherently due to the much higher reactivity of aluminium complexes when compared to boron reagents, often leading to decomposition of the Al‐based Lewis acid.[^21^, ^22^] However, the high reactivity of Al complexes may also hold promise for efficient catalysis. Motivated by the high potential of cationic Al catalysts like AlEt_2_^+^ in CO_2_ to methane conversion,^[31]^ we systematically investigated the application of cationic Al catalysts in imine hydrogenation. While it is known that neutral Al compounds like Al*i*Bu_3_ catalyse this reaction under harsh conditions (>100 bar H_2_, 100 °C)^[32]^ and LiAlH_4_ is an effective catalyst under relatively mild conditions (1 bar, 80 °C),[^33^, ^34^] we now report a series of highly Lewis acidic cationic β‐diketiminate (BDI) Al catalysts. Similar as in the recently reported imine hydrogenation with cationic Zr catalysts,^[35]^ the advantage of cationic (BDI)Al^+^ catalysts is the facile control over electronics and sterics by tuning the ligand through variation of substituents. A potential mechanism is based on the isolation of intermediates in the catalytic cycles and supported by DFT calculations.

## Results and Discussion

### Complex syntheses and structures

**Scheme 2:**
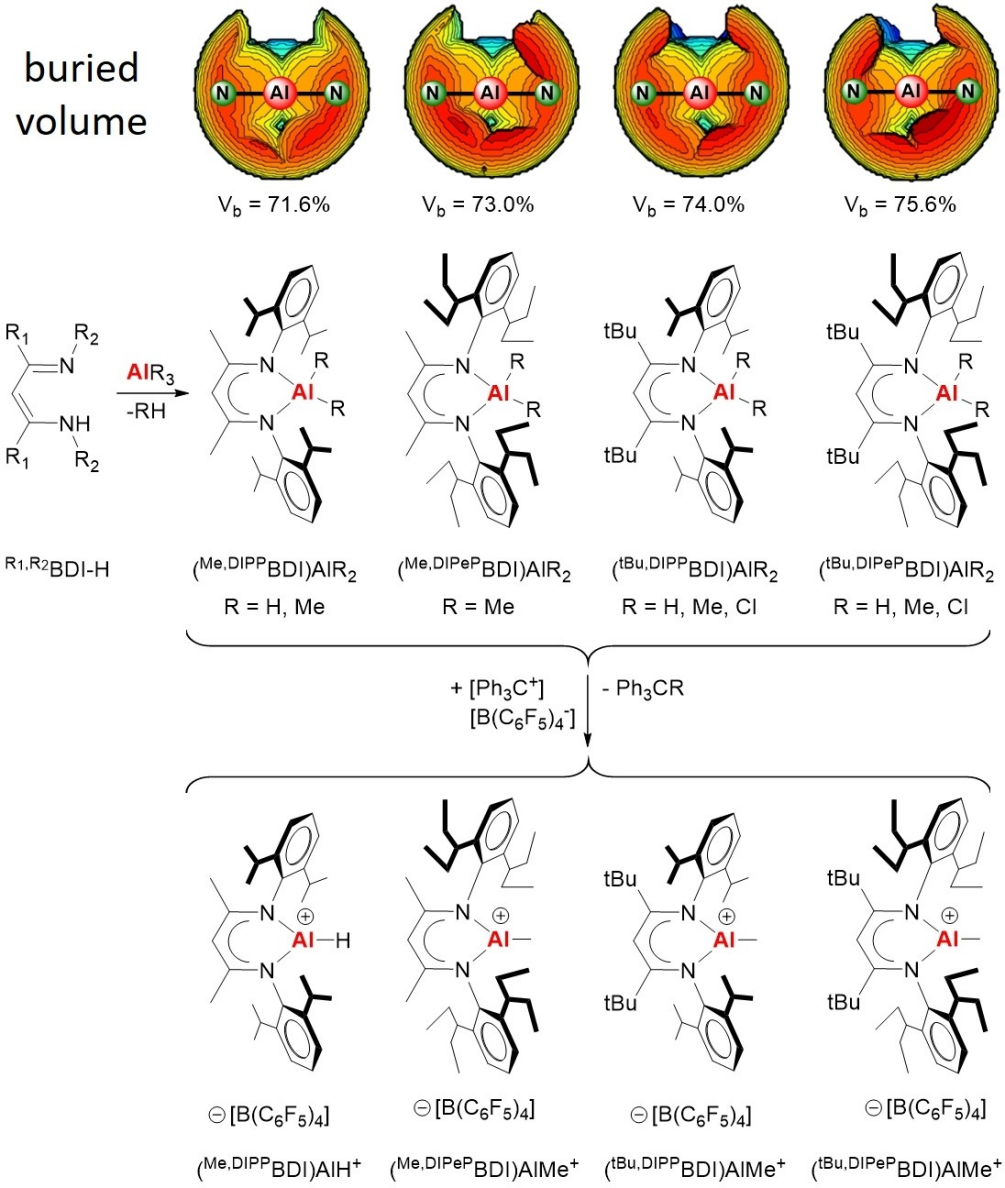
Synthesis of cationic aluminium complexes.

Our studies focussed on a series of BDI ligands in which the steric bulk was controlled by variation of the backbone substituent R1 (Me or *t*Bu) and N‐substituent R2 (DIPP or DIPeP); see Scheme 2 (DIPP=2,6‐C(H)Me_2_‐phenyl, DIPeP=2,6‐C(H)Et_2_‐phenyl). Deprotonation of the β‐diketimines with either AlMe_3_ or AlH_3_⋅NMe_3_ gave the corresponding aluminium methyl or hydride complexes. Although aluminium methyl and hydride complexes with the smallest ligand, ^Me,DIPP^BDI, have been reported,[^36^, ^37^] those with the bulkier BDI ligands were hitherto unknown. It was found that deprotonation of the β‐diketimine proligands becomes more difficult with increasing ligand bulk. While deprotonations of ^Me,DIPP^BDI−H and ^*t*Bu,DIPP^BDI−H with AlMe_3_ are complete within a few hours at room temperature, formation of the bulkier complexes needed harsher conditions: (^Me,DIPeP^BDI)AlMe_2_ (70 °C, 12 h) and (^*t*Bu,DIPeP^BDI)AlMe_2_ (95 °C, 140 h). In case of ^Me,DIPeP^BDI−H deprotonation, we have also been able to isolate the coordination complex (^Me,DIPeP^BDI−H)⋅AlMe_3_ which could be considered the first intermediate along the reaction coordinate.

**Figure 1:**
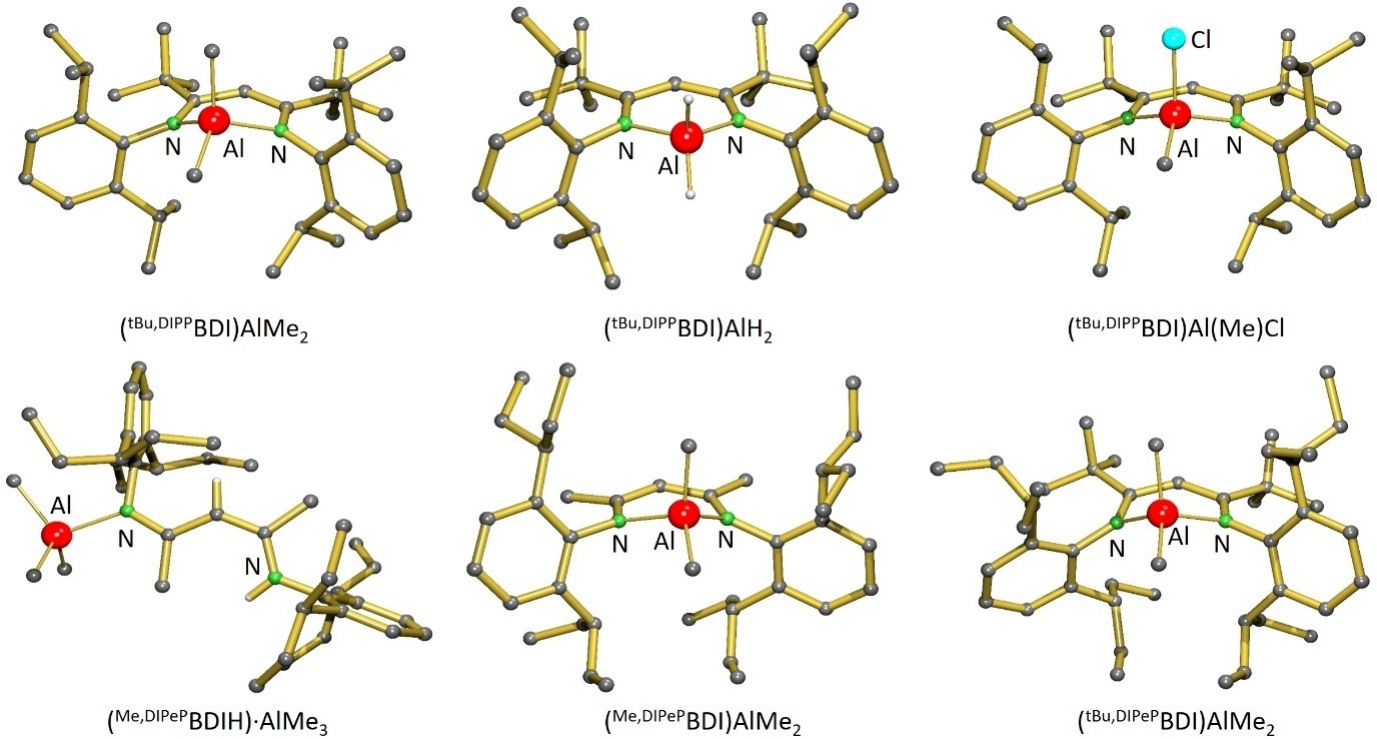
Crystal structures of neutral Al complexes; H atoms are partially omitted for clarity.

Crystal structures of the aluminium hydride complex (^*t*Bu,DIPP^BDI)AlH_2_, the aluminium methyl complexes (^*t*Bu,DIPP^BDI)AlMe_2_, (^Me,DIPeP^BDI)AlMe_2_ and (^*t*Bu,DIPeP^BDI)AlMe_2_, as well as the intermediate (^Me,DIPeP^BDI−H)⋅AlMe_3_, are shown in Figure 1. Like in the previously reported structures of (^Me,DIPP^BDI)AlMe_2_ and (^Me,DIPP^BDI)AlH_2_,[^36^, ^37^] the (BDI)AlR_2_ complexes exhibit Al centres with a tetrahedral coordination geometry and in all cases Al resides out of the BDI ligand plane. Calculation of the buried volume for comparable aluminium alkyl complexes (Scheme 2) show that ligand bulk increases along the row ^Me,DIPP^BDI<^Me,DIPeP^BDI≈^*t*Bu,DIPP^BDI<^*t*Bu,DIPeP^BDI.

**Figure 2:**
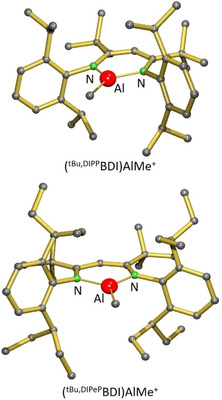
Crystal structures of the cations in [(^*t*Bu,DIPP^BDI)AlMe^+^][B(C_6_F_5_)_4_^−^] and [(^*t*Bu,DIPeP^BDI)AlMe^+^][B(C_6_F_5_)_4_^−^]; H atoms are omitted for clarity.

The neutral (BDI)AlR_2_ complexes were converted into (BDI)AlR^+^ cations by reaction with [Ph_3_C^+^][B(C_6_F_5_)_4_^−^] in the polar but weakly coordinating solvent chlorobenzene (Scheme 1). Like observed previously in the syntheses of comparable (BDI)Mg^+^ cations[^14^, ^15^, ^16^, ^17^] or (BDI)Zn^+^ cations,^[18]^ the colour change from dark orange towards pale yellow or colourless indicated completion of the reaction. Purification of the borate salts, however, was challenging and afforded the laborious development of individual procedures for each individual complex. All crystallization attempts were hampered by the formation of clathrates which is typical for these type of complexes.[^19^, ^20^, ^36^, ^38^, ^39^, ^40^, ^41^] In some cases crystallization could be enforced by scratching the glass walls with a spatula. Thus, the cations (^Me,DIPP^BDI)AlH^+^, (^Me,DIPeP^BDI)AlMe^+^, (^*t*Bu,DIPP^BDI)AlMe^+^ and (^*t*Bu,DIPeP^BDI)AlMe^+^ were isolated in the form of their borate salts as off‐white microcrystalline solids in yields of 65–99 %. All complexes were fully characterized by NMR methods and elemental analysis and for [(^*t*Bu,DIPP^BDI)AlMe^+^][B(C_6_F_5_)_4_^−^] and [(^*t*Bu,DIPeP^BDI)AlMe^+^][B(C_6_F_5_)_4_^−^] crystals structures could be determined (Figure 2). These reveal charge‐separated species with a trigonal planar coordination geometry for the Al centres. In contrast, the previously reported complex [(^Me,DIPP^BDI)AlMe^+^][B(C_6_F_5_)_4_^‐^]^[38]^ features an Al centre with an additional Al⋅⋅⋅FC_6_F_4_B(C_6_F_5_)_3_ contact. All cationic Al complexes dissolve moderately in bromobenzene‐*d*_5_ and ^1^H, ^13^C, ^11^B and ^19^F NMR spectra are in agreement with the highly symmetric species as observed in the solid state structures.

Attempts to prepare similar (BDI)AlCl^+^ cations, in which the Al centre should be considerably more Lewis acidic, failed. Reaction of (^*t*Bu,DIPP^BDI)Al(Me)Cl, prepared by deprotonation of the β‐diketimine with AlMe_2_Cl, with the trityl salt [Ph_3_C^+^][B(C_6_F_5_)_4_^−^] gave a mixture of (^*t*Bu,DIPP^BDI)AlCl_2_ and [(^*t*Bu,DIPP^BDI)AlMe^+^][B(C_6_F_5_)_4_^−^]. The *in situ* generated cation (^*t*Bu,DIPP^BDI)AlCl^+^ is presumably too Lewis acidic to be isolated and after abstraction of another Cl from the starting material (^*t*Bu,DIPP^BDI)Al(Me)Cl the cation (^*t*Bu,DIPP^BDI)AlMe^+^ is formed.

The Lewis acidity of various cationic Al complexes was determined by the Gutmann‐Beckett method (Table 1). Therein the perturbation of the ^31^P NMR shift of Et_3_PO coordinated to the Lewis acid of interest is converted into an acceptor number (AN) that ranges from hexane (AN=0) to SbCl_5_ (AN=100).[^42^, ^43^] Benchmark Lewis acids like B(C_6_F_5_)_3_ (AN=77.1) and AlCl_3_ (AN=87) are known to be strong Lewis acids and therefore located in the top quarter of this scale.

**Table 1:** Quantification of the Lewis acidity of different cationic Al complexes (as B(C_6_F_5_)_4_^−^ salt) with the Gutmann‐Beckett method (based on ^31^P NMR shifts in bromobenzene‐*d*_5_).

Cation	Acceptor Number (AN)^[a]^
(^*t*Bu,DIPP^BDI)AlMe^+^	85.6
(^*t*Bu,DIPeP^BDI)AlMe^+^	85.9
(^Me,DIPP^BDI)AlMe^+^	89.7
(^Me,DIPeP^BDI)AlMe^+^	90.8
(^Me,DIPP^BDI)AlH^+^	95.3

[a] AN=2.21×[δ^31^P(Et3PO complex)−41]

The highest AN of 95.3 was observed for the cation (^Me,DIPP^BDI)AlH^+^. This is significantly higher than the AN of 89.7 for Jordan's cation (^Me,DIPP^BDI)AlMe^+^ which we previously reported.^[20]^ The much higher Lewis acidity of the hydride *vs*. the methyl complex could be explained by the electron releasing properties of the Me group (Hammett parameters: σ_m_=−0.07, σ_p_=−0.17)^[44]^ but partially also could be related to steric factors. The better accessibility of the metal centre in (^Me,DIPP^BDI)AlH^+^ may lead to stronger complexation of Et_3_PO. Indeed, the AN for the most sterically congested cations (^*t*Bu,DIPP^BDI)AlMe^+^ (85.6) and (^*t*Bu,DIPeP^BDI)AlMe^+^ (85.9) is considerably lower than that for (^Me,DIPP^BDI)AlMe^+^ (89.7). Since the AN for (^Me,DIPeP^BDI)AlMe^+^ is 90.8, also electronic factors could be important: *t*Bu substituents in the backbone are much more electron releasing than the Me substituents.

## Catalytic imine hydrogenation

All cationic Al complexes were investigated as Lewis acidic catalysts for imine hydrogenation in a chlorobenzene/C_6_D_6_ mixture (2/1); see ESI for further details (Figures S52–S71). It was found that the cationic Al complexes with DIPeP substituents at N were fully inactive. Considerable activity was observed for the cation (^*t*Bu,DIPP^BDI)AlMe^+^ but the somewhat less bulky cation (^Me,DIPP^BDI)AlMe^+^ showed only slight activities (Table 2). In contrast, a similar hydride complex (^Me,DIPP^BDI)AlH^+^, which was found to be the most Lewis acidic in the series, showed no activity. These results clearly demonstrate that imine hydrogenation with Lewis acidic cationic Al complexes requires a fine balance between sterics and electronics.

**Table 2:** Imine hydrogenation catalysed by cationic aluminium complexes with either (^*t*Bu,DIPP^BDI)AlMe^+^ or (^Me,DIPP^BDI)AlMe^+^ cations. All reactions were performed in chlorobenzene/C_6_D_6_ (2/1; v/v).

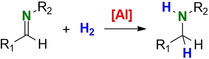								
Entry	[Al]	mol %	R_1_	R_2_	H_2_ [bar]	*T* [°C]	*t* [h]	Conv.
1	*t*Bu,DIPP	10	Ph	*t*Bu	1.5	60	3.5	>99 %
2	Me,DIPP	10	Ph	*t*Bu	1.5	60	90	98 %
3	*t*Bu,DIPP	10	Ph	*t*Bu	1.5	25	66	>99 %
4	*t*Bu,DIPP	10	Ph	*t*Bu	6	25	19	98 %
5	*t*Bu,DIPP	5	Ph	*t*Bu	6	25	90	88 %
6	*t*Bu,DIPP	5	Ph	*t*Bu	1.5	60	240	95 %
7	*t*Bu,DIPP	5	Ph	*t*Bu	1.5	80	66	>99 %
8	*t*Bu,DIPP	10	*p*‐Cl−C_6_H_4_	*t*Bu	1.5	60	3	>99 %
9	Me,DIPP	10	*p*‐Cl−C_6_H_4_	*t*Bu	1.5	60	40	95 %
10	*t*Bu,DIPP	10	*p*‐Cl−C_6_H_4_	*t*Bu	1.5	25	8	>99 %
11	*t*Bu,DIPP	5	*p*‐Cl−C_6_H_4_	*t*Bu	1.5	60	66	>99 %
12	*t*Bu,DIPP	5	*p*‐Cl−C_6_H_4_	*t*Bu	1.5	80	16	>99 %
13	*t*Bu,DIPP	10	*p*‐Me−C_6_H_4_	*t*Bu	1.5	60	120	>99 %
14	*t*Bu,DIPP	10	*p*‐Me−C_6_H_4_	*t*Bu	6	60	66	>99 %
15	*t*Bu,DIPP	10	*p*‐Me−C_6_H_4_	*t*Bu	6	25	160	20 %
16	*t*Bu,DIPP	10	Mes	*t*Bu	1.5	60	37	>99 %
17	*t*Bu,DIPP	10	Mes	*t*Bu	1.5	25	144	>99 %
18	*t*Bu,DIPP	10	Mes	*t*Bu	6	60	90	>99 %
19	*t*Bu,DIPP	5	Mes	*t*Bu	1.5	60	66	>99 %
20	*t*Bu,DIPP	10	*t*Bu	*i*Pr	1.5	60	17.5	>99 %
21	*t*Bu,DIPP	10	*t*Bu	*i*Pr	6	25	210	>99 %
22	*t*Bu,DIPP	10	*t*Bu	*i*Pr	1.5	25	120	>99 %
23	*t*Bu,DIPP	5	*t*Bu	*i*Pr	1.5	25	17	30 %
24	*t*Bu,DIPP	10	*t*Bu	*t*Bu	1.5	60	16	>99 %
25	*t*Bu,DIPP	10	*t*Bu	*t*Bu	1.5	25	17	>99 %
26	*t*Bu,DIPP	5	*t*Bu	*t*Bu	1.5	80	144	67 %
27	*t*Bu,DIPP	10	*n*Pr	*t*Bu	1.5	80	140	28 %

Within the FLP concept, bulky ligands at Al are required. If the Al centre is accessible for imine coordination, a stable (BDI)Al^+^⋅⋅⋅imine complex is formed which does not react with H_2_. Indeed, NMR studies show strong complexation between (^Me,DIPP^BDI)AlH^+^ and PhC(H)=N*t*Bu to give a tightly bound complex that is fully unreactive towards H_2_ (Figure S56). Also heating this complex to 80 °C did not lead to insertion of the imine in the Al−H bond. The Al cations protected by a BDI ligand with DIPeP‐substituents at N do not form a complex with PhC(H)=N*t*Bu (Figure S57–S58). Also (^*t*Bu,DIPP^BDI)AlMe^+^ does not form a complex with PhC(H)=N*t*Bu but its combination reacts smoothly with H_2_ (Figure S52–S53).

Within the FLP concept, a strongly Lewis acidic metal centre is required for H_2_ activation. However, if the metal's Lewis acidity is too strong, the strongly bound Al hydride complex formed after H_2_ activation is not hydridic enough to react with the imine. The most active cation, (^*t*Bu,DIPP^BDI)AlMe^+^, incorporates the perfect balance between moderate sterics and moderate Lewis acidity.

At low H_2_ pressure (1.5–6 bar) the cation (^*t*Bu,DIPP^BDI)AlMe^+^ performed well in the hydrogenation of PhC(H)=N*t*Bu, the benchmark substrate in imine hydrogenation (Table 2). Catalyst loadings could be lowered to 5 mol% and temperatures to 25 °C. The performance of (^*t*Bu,DIPP^BDI)AlMe^+^ is comparable to that of B(C_6_F_5_)_3_ or to that of B/P or Zr/P FLP's.[^7^, ^8^, ^35^] The catalyst tolerates (*sp* ^2^)C−Cl in (*p*‐Cl−C_6_H_4_)C(H)=N*t*Bu as a functional group and, due to the beneficial electronic effect of a *para*‐Cl substituent (σ_p_=+0.23),^[44]^ its hydrogenation is significantly faster (entries 8–12). Reduction of (*p*‐Me−C_6_H_4_)C(H)=N*t*Bu is considerably slower (σ_p_=−0.17)^[44]^ but full conversion could be reached at 60 °C (Table 2, entries 13–15). A bulky mesityl substituent at C also retards conversion (entries 16–19) which is related to steric hindrance impeding hydride transfer to C. Alkyl substituents on the imine C slow down conversion by electron release, making the imine C less electrophilic. Consequently, long reaction times are needed for hydrogenation of *t*BuC(H)=N*t*Bu, *t*BuC(H)=N*i*Pr, or *n*PrC(H)=N*t*Bu (entries 20–27). No conversion was found for PhC(H)=NPh, a substrate with a conjugated (activated) C=N bond. This is likely due to formation of intermediate PhCH_2_N(Ph)^−^ which is stabilized by resonance. Also the imines CF_3_C(H)=N*t*Bu, MesC(H)=NMes, *i*PrC(H)=N*t*Bu or ketimines could not be converted due to a combination of steric or electronic factors. Although the catalyst could also not reduce ketones with H_2_, it was found that using 10 mol % (^*t*Bu,DIPP^BDI)AlMe^+^ converts benzaldehyde to benzylbenzoate at 60 °C in quantitative yield (10 mol % cat., 60 °C, 4 days), irrelevant whetherH_2_ is present or not (Figure S59). This transformation, known as the Tishchenko reaction, is traditionally catalysed by Al alkoxides.[^45^, ^46^, ^47^] The ability of cationic Al complexes to mediate this reaction was demonstrated by the group of Venugopal just recently.^[48]^

## Mechanism: experimental and theoretical investigations

**Scheme 3:**
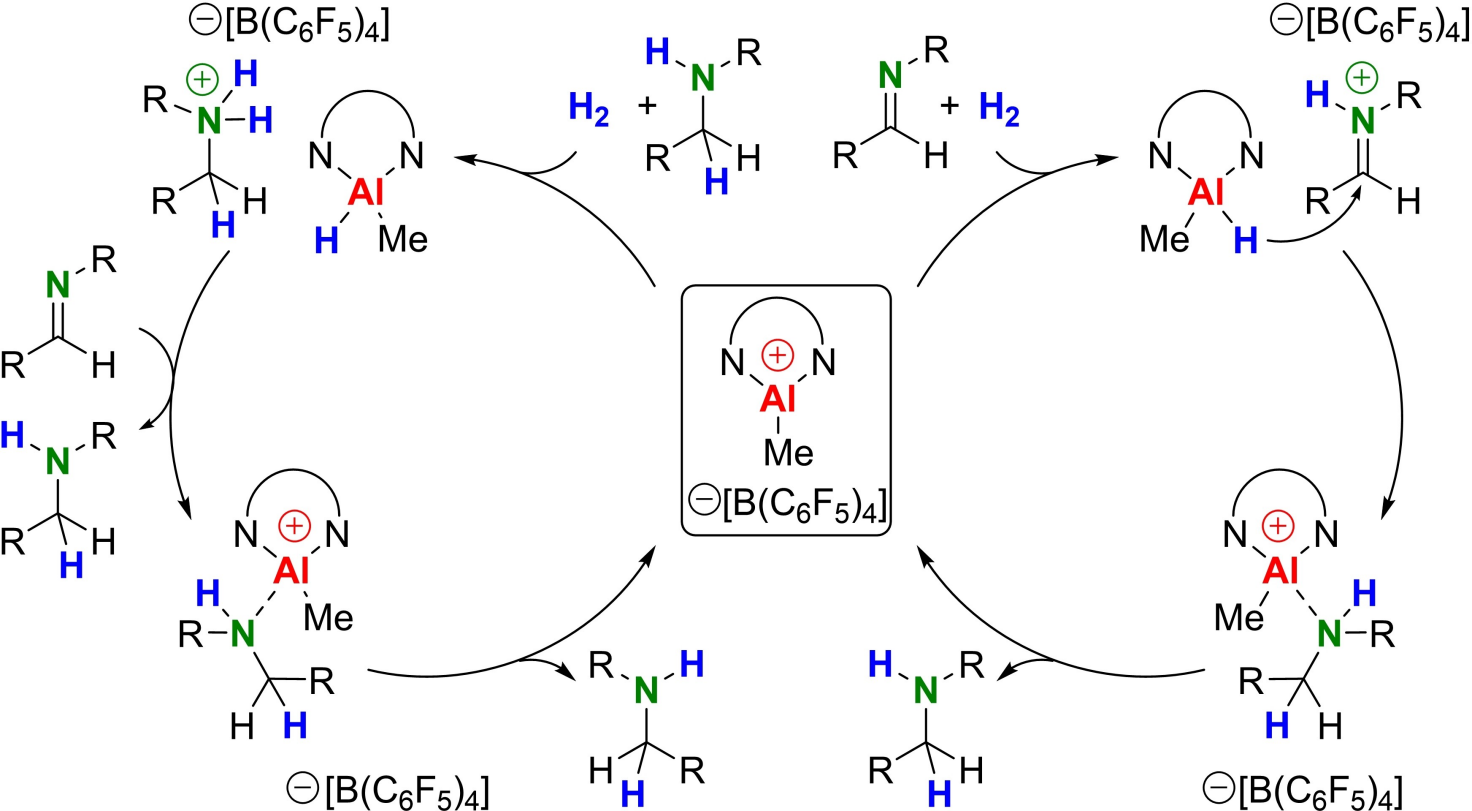
Interconnected catalytic cycles for imine hydrogenation with [(^*t*Bu,DIPP^BDI)AlMe^+^][B(C_6_F_5_)_4_^−^].

The requirement that for any reactivity all three, the catalyst, imine and H_2_, need to be present simultaneously, implies a FLP type mechanism similar to that proposed by Stephan and co‐workers (Scheme 1b).^[8]^ After the catalytic imine hydrogenation, the original catalyst [(^*t*Bu,DIPP^BDI)AlMe^+^][B(C_6_F_5_)_4_^−^] could be successfully recycled by crystallization. We also have been able to crystallize borate salts of iminium and ammonium cations (see Figure S80–S82). Therefore, we propose that the reaction starts with the iminium catalytic cycle in Scheme 1b but with increasing amine concentrations a switch to an autocatalytic ammonium cycle is predicted (Scheme 3). The possibility that amine product and Lewis acid form an active FLP was already suggested by Klankermeyer^[9]^ and later verified by DFT calculations in the group of Papai.^[49]^ These interconnected cycles of FLP activation with either imine or amine as the Lewis base are now a generally accepted working hypothesis in FLP‐catalysed imine hydrogenation.[^13^, ^50^, ^51^, ^52^]

**Scheme 4:**
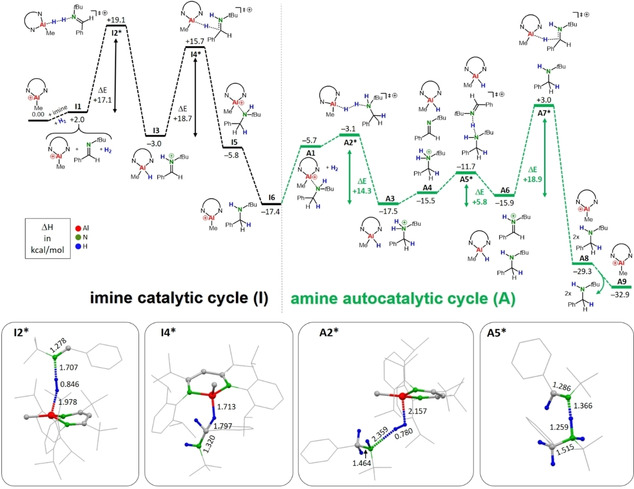
Energy profile for imine hydrogenation with catalyst (^*t*Bu,DIPP^BDI)AlMe^+^; the non‐coordinating anion B(C_6_F_5_)_4_^−^ has been neglected for simplicity (B3PW91/def2TZVP, PCM=PhCl, relative ΔH values at 298 K and 1 bar are given in kcal mol^−1^).

Comprehensive DFT calculations on catalytic hydrogenation of PhC(H)=N*t*Bu with the catalyst (^*t*Bu,DIPP^BDI)AlMe^+^ (the borate anion was neglected for simplicity) have been performed at the B3PW91/def2TZVP level of theory with solvent correction using the PCM method for PhCl. Scheme 4 shows the energy profile for the imine cycle and the integrated autocatalytic pathway (ΔH in kcal mol^−1^).

Combination of (^*t*Bu,DIPP^BDI)AlMe^+^, imine and H_2_ does not lead to any complex formation (**I1**). In transition I2* the cation and imine cooperate in breaking the H−H bond which needs an activation energy of +17.1 kcal/mol. Formation of (^*t*Bu,DIPP^BDI)Al(Me)H and the iminium cation is exothermic by −5.0 kcal/mol. The activation energy for hydride attack at the iminium cation is +18.7 kcal/mol which is slightly higher (**I3**→I4*) than that for H_2_ cleavage. Due to steric congestion in complex **I5**, the release of amine product is exothermic by −11.6 kcal/mol.

The presence of amine opens up the autocatalytic cycle in which Al and amine activate H_2_. Taking the Al‐amine complex from the former cycle as a starting point, the activation energy is only +2.6 kcal/mol. Starting from the separate Al cation and amine, also only+14.3 kcal/mol is required to reach transition state A2*. Note that the transition states for H_2_ activation with Al/imine and Al/amine are quite different (selected transition states are shown in Scheme 3; all other calculated structures are shown in Figure S83). Whereas the Al/imine transition state I2* is close to linear (Al⋅⋅⋅H−H: 161.3°, H−H⋅⋅⋅N 174.7°) and late on the reaction coordinate (H−H: 0.846 Å), the Al/amine transition state A2* is bent (Al⋅⋅⋅H−H: 108.5°, H−H⋅⋅⋅N 164.4°) and early on the reaction coordinate (H−H: 0.780 Å). Subsequent proton transfer from the ammonium cation to the imine is a low energy process with an activation energy of +5.8 kcal/mol. This is followed by hydride→iminium attack which needs an activation energy of +18.9 kcal/mol.

The energy profile shows that the rate determining step in both cycles is nucleophilic hydride→iminium attack. The resting states in the catalytic cycles are therefore the iminium and ammonium borate salts. This explains why these could be successfully crystallized from reaction mixtures during catalysis. Cleavage of the H−H bond is more efficient with the Al/amine FLP which means that the autocatalytic cycle becomes more important with reaction progress. Similar conclusions were drawn from calculational studies on imine hydrogenation with B(C_6_F_5_)_3_.[^49^, ^52^]

## Conclusion

We present a detailed experimental and computational study on the catalytic transformation of imines with low pressure of dihydrogen at ambient temperatures mediated by cationic Al complexes. Cationic β‐diketiminate Al complexes are readily available in good yields by reaction of (BDI)AlR_2_ (R=Me or H) with [Ph_3_C^+^][B(C_6_F_5_)_4_^−^]. β‐Diketiminate ligands of increasing bulk have been used: ^Me,DIPP^BDI<^Me,DIPeP^BDI≈^*t*Bu,DIPP^BDI<^*t*Bu,DIPeP^BDI. Crystal structures of [(^*t*Bu,DIPP^BDI)AlMe^+^][B(C_6_F_5_)_4_^−^] and [(^*t*Bu,DIPeP^BDI)AlMe^+^][B(C_6_F_5_)_4_^−^] revealed charge separate cation‐anion pairs with planar trigonal coordination geometries for Al. Quantification of the Lewis acidity with the Gutmann‐Beckett method gave a wide span of acceptor numbers ranging from AN=85.6 for (^*t*Bu,DIPP^BDI)AlMe^+^ to AN=95.3 for the cationic Al hydride (^Me,DIPP^BDI)AlH^+^.

The catalytic activity of these cationic Al complexes depends strongly on steric and electronic effects which require a fine balance. While the open, highly Lewis acidic cation (^Me,DIPP^BDI)AlH^+^ strongly coordinates imines, rendering it essentially inert for FLP activation of H_2_, the most shielded cation (^*t*Bu,DIPeP^BDI)AlMe^+^ of lower Lewis‐acidity does not bind imines but also not activate H_2_. High activities were observed for (^*t*Bu,DIPP^BDI)AlMe^+^ which efficiently reduced various imines.

Isolation of iminium and ammonium reaction intermediates suggests that two catalytic cycles operate in concert. Hydrogen is activated either by FLP reactivity of an Al⋅⋅⋅imine couple or, after formation of significant quantities of amine, by reaction with an Al⋅⋅⋅amine couple. DFT calculations show that the latter autocatalytic Al⋅⋅⋅amine cycle is energetically the most favourable pathway. The most important message of this work is that small changes in the ligand environment of cationic Al complexes can have major consequences for successful FLP catalysis.

## Supporting Information

Experimental data, crystallographic details including ORTEP plots, ^1^H, ^11^B, ^19^F and ^13^C NMR spectra, details for the catalysis and DFT calculations including XYZ‐files.

## Conflict of interest

The authors declare no conflict of interest.

## Supporting information

As a service to our authors and readers, this journal provides supporting information supplied by the authors. Such materials are peer reviewed and may be re‐organized for online delivery, but are not copy‐edited or typeset. Technical support issues arising from supporting information (other than missing files) should be addressed to the authors.

Supplementary
