# Supplementary material for: Cationic Aluminium Complexes as Catalysts for Imine Hydrogenation
Source: Chemistry. 2021 May 2;27(28):7756–63. doi: 10.1002/chem.202100641 (PMC8252007; doi:10.1002/chem.202100641)
Supplement: Supplementary file 1 — Supplementary [file CHEM-27-7756-s001.pdf]

Chemistry–A European Journal

Supporting Information

# Cationic Aluminium Complexes as Catalysts for Imine Hydrogenation

Alexander Friedrich, Jonathan Eyselein, Holger Elsen, Jens Langer, Jürgen Pahl, Michael Wiesinger, and Sjoerd Harder*

# Contents


**1. Supporting Experimental data** **S2**

**1.1 Synthetic procedures** S2

**1.2 NMR spectra of synthesized compounds** S7

1.2.1 Spectra of ($^{tBu,DIPP}$BDI)AlMe$_2$ S7
1.2.2 Spectra of ($^{tBu,DIPP}$BDI)AlH$_2$ S9
1.2.3 Spectra of ($^{tBu,DIPP}$BDI)AlMeCl S11
1.2.4 Spectra of ($^{Me,DIPeP}$BDI)AlMe$_2$ S12
1.2.5 Spectra of ($^{tBu,DIPeP}$BDI)AlMe$_2$ S14
1.2.6 Spectra of [($^{tBu,DIPP}$BDI)AlMe$^+$][B(C$_6$F$_5$)$_4^-$] S15
1.2.7 Spectra of [($^{Me,DIPP}$BDI)AlH$^+$][B(C$_6$F$_5$)$_4^-$] S18
1.2.8 Spectra of [($^{Me,DIPeP}$BDI)AlMe$^+$][B(C$_6$F$_5$)$_4^-$] S22
1.2.9 Spectra of [($^{tBu,DIPeP}$BDI)AlMe$^+$][B(C$_6$F$_5$)$_4^-$] S25

**1.3 Lewis Acidity measurement (Gutmann-Beckett Method)** S29

1.3.1 Measurement for [($^{tBu,DIPP}$BDI)AlMe$^+$][B(C$_6$F$_5$)$_4^-$] S29
1.3.2 Measurement for [($^{Me,DIPP}$BDI)AlH$^+$][B(C$_6$F$_5$)$_4^-$] S30
1.3.3 Measurement for [($^{Me,DIPeP}$BDI)AlMe$^+$][B(C$_6$F$_5$)$_4^-$] S31
1.3.4 Measurement for [($^{tBu,DIPeP}$BDI)AlMe$^+$][B(C$_6$F$_5$)$_4^-$] S32

**1.4 NMR data related to catalytic imine hydrogenation** S34

**1.5 Single crystal X-ray diffraction** S47

**1.6 Computational details** S58

1.6.1. Calculated structures of the simple and auto-induced cycle S59
1.6.2. XYZ files S60

**2. References** **S110**

# 1. Supporting Experimental data

## 1.1. Synthetic procedures

**General Considerations:** All experiments were conducted under an inert nitrogen atmosphere using standard Schlenk and glovebox techniques (MBraun, Labmaster SP). All solvents were degassed with nitrogen, dried over activated aluminium oxide (Solvent Purification System: Pure Solv 400−4−MD, Innovative Technology) and stored over 3Å molecular sieves unless noted otherwise. Chlorobenzene was dried over calcium hydride, distilled under $N_2$ atmosphere and stored over molecular sieves 3Å. $C_6D_6$ and $C_6D_5Br$ (99.6% D, Sigma Aldrich) were dried over 3Å molecular sieves. $[CPh_3^+][B(C_6F_5)_4^-]$ (Boulder Scientific) was used as received. $^{(tBu,DIPP)}$BDIH,[S1] $^{(Me,DIPeP)}$BDIH,[S2] $^{(tBu,DIPeP)}$BDIH,[S3] ($^{Me,DIPP}$BDI)$AlH_2$[S4] and [($^{Me,DIPP}$BDI)$AlMe^+$][$B(C_6F_5)_4^-$][S5] were synthesized according to literature procedures. NMR spectra were recorded with a Bruker Avance III HD 400 MHz or a Bruker Avance III HD 600MHz spectrometer. The spectra were referenced to the respective residual signals of the deuterated solvents. Elemental analysis was performed with an Euro EA 3000 (Euro Vector) analyzer. All crystal structures have been measured on a SuperNova (Agilent) diffractometer with dual Cu and Mo microfocus sources and an Atlas S2 detector. Crystallographic data have been deposited with the Cambridge Crystallographic Data Centre as supplementary publication no. 2063692-2063702 (see ESI for details). Copies of the data can be obtained free of charge on application to CCDC, 12 Union Road, Cambridge CB21EZ, UK (fax: (+44)1223-336-033; E-mail: deposit@ccdc.cam.ac.uk).

**Synthesis of ($^{tBu,DIPP}$BDI)$AlMe_2$:** $^{(tBu,DIPP)}$BDI-H (2.00 g, 3.98 mmol, 1 eq.) was dissolved in hexane (200 mL) and $AlMe_3$ (0.96 mL, 10 mmol, 2.5 eq.) was added while stirring rapidly. The reaction mixture was stirred for further 12 h and subsequently all volatiles including unreacted $AlMe_3$ were removed *in vacuo*. The product ($^{tBu,DIPP}$BDI)$AlMe_2$was obtained as an off-white solid (2.11 g) in 95% yield. Crystals suitable for X-ray diffraction were grown from a concentrated hexane solution at −20°C. **$^1$H NMR** ($C_6D_6$, 400 MHz, 298 K): δ 7.12−7.02 (m, 6H, aryl−*H*), 5.85 (s, 1H, CC*H*C), 3.49 (sept, $^3J_{HH}$ = 6.8 Hz, 4H, C*H*($CH_3$)$_2$), 1.31 (d, $^3J_{HH}$ = 6.8 Hz, 12H, CH(C*H*$_3$)$_2$), 1.30 (d, $^3J_{HH}$ = 6.8 Hz, 12H, CH(C*H*$_3$)$_2$), 1.10 (s, 18H, C(C*H*$_3$)$_3$), −0.45 (s, 6H, Al(C*H*$_3$)$_2$) ppm. **$^{13}$C NMR** ($C_6D_6$, 101 MHz, 298 K): δ 179.1 (s, *C*CH*C*), 144.5 (s, aryl−*C*), 142.9 (s, aryl−*C*), 126.6 (s, aryl−*C*), 124.4 (s, aryl−*C*), 101.1 (s, C*C*HC), 43.2 (s, *C*($CH_3$)$_3$), 32.5 (s, C(*C*H$_3$)$_3$), 28.4 (s, *C*H($CH_3$)$_2$), 26.8 (s, CH(*C*H$_3$)$_2$), 24.6 (s, CH(*C*H$_3$)$_2$), −9.7 (s, Al(*C*H$_3$)$_2$) ppm. **Elemental analysis** Found: C, 79.67; H, 10.70; N, 4.86. Calc. for $C_{37}H_{59}AlN_2$: C, 79.52; H, 10.64; N, 5.01%.

**Synthesis of ($^{tBu,DIPP}$BDI)$AlH_2$:** $^{(tBu,DIPP)}$BDI-H (67 mg, 133 µmol, 1 eq.) was dissolved in hexane and $AlH_3$·$NMe_3$ (12 mg, 133 µmol, 1 eq.) was added. The reaction mixture was stirred at 50°C for 2 d and concentrated under reduced pressure to initiate crystallization. Decantation of the mother liquor and washing with cold pentane (3 x 2 mL) gave the product ($^{tBu,DIPP}$BDI)$AlH_2$ as needle shaped crystals in

56% yield (39 mg). Crystals suitable for X-ray diffraction were grown from the reaction mixture. The complex is only poorly soluble in $C_6D_6$. **$^1$H NMR** ($C_6D_6$, 400 MHz, 298 K): δ 7.10 (m, 4H, aryl−*H*), 7.01 (m, 2H, aryl−*H*), 5.70 (s, 1H, CC*H*C), 3.35 (sept, $^3J_{HH}$ = 6.8 Hz, 4H, C*H*($CH_3$)$_2$), 1.29 (d, $^3J_{HH}$ = 6.8 Hz, 12H, CH(C$H_3$)$_2$), 1.27 (d, $^3J_{HH}$ = 6.8 Hz, 12H, CH(C$H_3$)$_2$), 1.10 (s, 18H, C(C$H_3$)$_3$) ppm. ($C_6D_5Br$ (for acquisition of 13C NMR), 400 MHz, 298 K): δ 7.06 (m, 6H, aryl−*H*), 5.72 (s, 1H, CC*H*C), 3.48 (sept, $^3J_{HH}$ = 6.8 Hz, 4H, C*H*($CH_3$)$_2$), 1.40 (d, $^3J_{HH}$ = 6.8 Hz, 12H, CH(C$H_3$)$_2$), 1.29 (d, $^3J_{HH}$ = 6.8 Hz, 12H, CH(C$H_3$)$_2$), 1.10 (s, 18H, C(C$H_3$)$_3$) ppm. Signals for Al$H_2$ unit was not obtained. **$^{13}$C NMR** ($C_6D_5Br$, 101 MHz, 298 K): δ 179.2 (s, *C*CH*C*), 144.8 (s, aryl−*C*), 141.3 (s, aryl−*C*), 124.6 (s, aryl−*C*), 123.2 (s, aryl−*C*), 98.5 (s, C*C*HC), 43.2 (s, *C*($CH_3$)$_3$), 32.8 (s, C(*C*$H_3$)$_3$), 28.6 (s, *C*H($CH_3$)$_2$), 27.2 (s, CH(*C*$H_3$)$_2$), 24.4 (s, CH(*C*$H_3$)$_2$ ppm. **Elemental analysis** Found: C, 79.38; H, 10.33; N, 5.84. Calc. for $C_{35}H_{55}AlN_2$: C, 79.20; H, 10.44; N, 5.28%.

**Synthesis of ($^{tBu,DIPP}$BDI)Al(Me)Cl:** $^{(tBu,DIPP)}$BDI-H (0.5 g, 0.99 mmol, 1 eq.) was dissolved in hexane (6 mL) and $AlMe_2Cl$ (1.5 mL, 1.5 mmol, 1.5 eq.) was added dropwise. After stirring of the reaction mixture for 3 d at room temperature all volatiles were removed under reduced pressure. The crude product was recrystallized from a concentrated benzene solution to give ($^{tBu,DIPP}$BDI)Al(Me)Cl as microcrystalline solid in 70% yield (0.4 g). Crystals suitable for X-ray diffraction were grown from the concentrated benzene solution. **$^1$H NMR** ($C_6D_6$, 400 MHz, 298 K): δ 7.08 (m, 4H, aryl−*H*), 6.99 (m, 2H, aryl−*H*), 6.01 (s, 1H, CC*H*C), 4.18 (sept, $^3J_{HH}$ = 6.6 Hz, 2H, C*H*($CH_3$)$_2$), 3.17 (sept, $^3J_{HH}$ = 6.7 Hz, 2H, C*H*($CH_3$)$_2$), 1.47 (d, $^3J_{HH}$ = 6.6 Hz, 6H, CH(C$H_3$)$_2$), 1.35 (d, $^3J_{HH}$ = 6.7 Hz, 6H, CH(C$H_3$)$_2$), 1.23 (d, $^3J_{HH}$ = 6.8 Hz, 6H, CH(C$H_3$)$_2$), 1.20 (d, $^3J_{HH}$ = 6.8 Hz, 6H, CH(C$H_3$)$_2$), 1.11 (s, 18H, C(C$H_3$)$_3$), -0.70 (s, 3H, Al*Me*Cl) ppm. **$^{13}$C NMR** ($C_6D_6$, 101 MHz, 298 K): δ 179.9 (s, *C*CH*C*), 146.2 (s, aryl−*C*), 143.9 (s, aryl−*C*), 141.6 (s, aryl−*C*), 127.3 (s, aryl−*C*), 125.1 (s, aryl−*C*), 124.1 (s, aryl−*C*), 101.9 (s, C*C*HC), 43.4 (s, *C*($CH_3$)$_3$), 32.3 (s, C(*C*$H_3$)$_3$), 28.8 (s, *C*H($CH_3$)$_2$), 28.4 (s, *C*H($CH_3$)$_2$), 27.9 (s, CH(*C*$H_3$)$_2$), 26.1 (s, CH(*C*$H_3$)$_2$), 24.8 (s, CH(*C*$H_3$)$_2$), 24.4 (s, CH(*C*$H_3$)$_2$) ppm. **Elemental analysis** Found: C, 74.89; H, 10.10; N, 4.55. Calc. for $C_{36}H_{56}AlClN_2$: C, 74.64; H, 9.74; N, 4.84%.

**Synthesis of ($^{Me,DIPeP}$BDI)AlMe$_2$:** $^{(Me,DIPeP)}$BDIH (0.25 g, 0.471 mmol, 1 eq.) was dissolved in toluene (8 mL) and $AlMe_3$ (0.113 mL, 1.18 mmol, 2.5 eq.) was added. The reaction mixture was stirred at 70°C for 12 h and subsequently all volatiles were removed *in vacuo*. The product ($^{Me,DIPeP}$BDI)AlMe$_2$ was obtained as a white solid in 96% yield (0.265 g). Crystals suitable for X−ray diffraction were grown from a concentrated hexane solution at −20°C. **$^1$H NMR** ($C_6D_6$, 400 MHz, 298 K): δ 7.15 (t$_{(covered\ by\ C_6D_6)}$, 2H, aryl−*H*), 7.03 (d, $^3J_{HH}$ = 7.7 Hz, 4H, aryl−*H*), 4.94 (s, 1H, CC*H*C), 3.29 (br. pent, 4H, C*H*($CH_2CH_3$)$_2$), 1.76 (m, 8H, CH(C$H_2$CH$_3$)$_2$), 1.59 (m, 8H, CH(C$H_2$CH$_3$)$_2$), 1.53 (s, 6H, *Me*BDI), 0.95 (t, $^3J_{HH}$ = 7.5 Hz, 12H, CH(CH$_2$C$H_3$)$_2$), 0.90 (t, $^3J_{HH}$ = 7.5 Hz, 12H, CH(CH$_2$C$H_3$)$_2$), -0.29 (s, 6H, Al*Me$_2$*) ppm. **$^{13}$C NMR** ($C_6D_6$, 151 MHz, 298 K): δ 170.8 (s, *C*CH*C*), 144.9 (s, aryl−*C*), 141.0 (s, aryl−*C*), 126.2 (s, aryl−*C*), 125.7 (s, aryl−*C*), 98.4 (s, C*C*HC), 40.2 (s, *Me*BDI), 28.8 (s, *C*H($CH_2CH_3$)$_2$), 27.9 (s, CH(*C*$H_2$CH$_3$)$_2$), 23.9 (s, CH(*C*$H_2$CH$_3$)$_2$), 11.5

(s, $CH(CH_2CH_3)_2$), 11.3 (s, $CH(CH_2CH_3)_2$), −8.2 (s, Al*Me₂*) ppm. **Elemental analysis** Found: C, 79.64; H, 10.95; N, 4.80. Calc. for $C_{39}H_{63}AlN_2$: C, 79.81; H, 10.82; N, 4.77%.

**Synthesis of ($^{tBu,DIPeP}$BDI)AlMe$_2$:** $^{(tBu,DIPeP)}$BDIH (0.500 g, 0.813 mmol, 1 eq.) was dissolved in toluene (5 mL). $AlMe_3$ (0.156 mL, 1.63 mmol, 2 eq.) was added dropwise. The reaction mixture was stirred at 95°C for 140 h and subsequently concentrated *in vacuo* until precipitation of a white solid. After decantation of the mother liquor the crude product was recrystallized form hexane at -30 °C to yield ($^{tBu,DIPeP}$BDI)AlMe$_2$ as pale yellow crystals in 89% yield (0.483 g). Crystals suitable for X−ray diffraction were grown from a concentrated hexane solution at −30°C. **$^1$H NMR** ($C_6D_6$, 400 MHz, 298 K): δ 7.08 (m, 2H, aryl−*H*), 6.98 (d, $^3J_{HH}$ = 7.6 Hz, 4H, aryl−*H*), 5.78 (s, 1H, CC*H*C), 3.38 (br. pent, 4H, C*H*($CH_2CH_3)_2$), 1.78 (m, 16H, CH(C*H₂*$CH_3)_2$), 1.08 (s, 18H, $C(CH_3)_3$), 1.03 (t, $^3J_{HH}$ = 7.4 Hz, 12H, $CH(CH_2C$*H₃*$)_2$), 0.86 (t, $^3J_{HH}$ = 7.4 Hz, 12H, $CH(CH_2C$*H₃*$)_2$), -0.31 (s, 6H, Al*Me₂*) ppm. **$^{13}$C NMR** ($C_6D_6$, 151 MHz, 298 K): δ 179.9 (s, *C*CH*C*), 144.9 (s, aryl−*C*), 141.6 (s, aryl−*C*), 126.4 (s, aryl−*C*), 124.8 (s, aryl−*C*), 102.1 (s, C*C*HC), 43.5 (s, *C*($CH_3)_3$), 39.4 (s, *C*H($CH_2CH_3)_2$), 32.5 (s, C(*C*$H_3)_3$), 26.8 (s, CH(*C*$H_2CH_3)_2$), 26.3 (s, CH(*C*$H_2CH_3)_2$), 11.0 (s, $CH(CH_2$*C*$H_3)_2$), 10.2 (s, $CH(CH_2$*C*$H_3)_2$), −7.6 (s, Al*Me₂*) ppm. **Elemental analysis** Found: C, 80.78; H, 10.88; N, 4.23. Calc. for $C_{45}H_{75}AlN_2$: C, 80.54; H, 11.27; N, 4.17%.

**Synthesis of [($^{tBu,DIPP}$BDI)AlMe$^+$][B($C_6F_5)_4^-$]:** ($^{tBu,DIPP}$BDI)AlMe$_2$ (0.218 g, 0.390 mmol, 1.2 eq.) and [$CPh_3^+$][B($C_6F_5)_4^-$] (0.300 g, 0.325 mmol, 1 eq.) were dissolved in chlorobenzene (3 mL). The brown solution was stirred overnight and subsequently the solvent was removed *in vacuo*. The partially solid residue was treated with hexane (3 mL) to give an off-white precipitate, which was thoroughly washed with hexane (7 x 3 mL) to yield [($^{tBu,DIPP}$BDI)AlMe$^+$][B($C_6F_5)_4^-$] in 99% yield (0.389 g). X-ray quality crystals were grown from a concentrated chlorobenzene/benzene mixture (1/1; v/v) layered with hexane.**$^1$H NMR** ($C_6D_5Br$, 400 MHz, 298 K): δ 7.17 (t, $^3J_{HH}$ = 7.8 Hz, 2H, aryl−*H*), 6.99 (d, $^3J_{HH}$ = 7.8 Hz, 4H, aryl−*H*), 6.25 (s, 1H, CC*H*C), 2.66 (sept, $^3J_{HH}$ = 6.8 Hz, 4H, C*H*($CH_3)_2$), 1.18 (d, $^3J_{HH}$ = 6.8 Hz, 12H, CH(C*H₃*)$_2$), 1.02 (s, 18H, C(C*H₃*)$_3$), 0.97 (d, $^3J_{HH}$ = 6.8 Hz, 12H, CH(C*H₃*)$_2$), −1.00 (s, 3H, AlC*H₃*) ppm. **$^{11}$B NMR** ($C_6D_5Br$, 128 MHz, 298 K): δ −16.0 (s, 1B, *B*($C_6F_5)_4^-$) ppm. **$^{19}$F NMR** ($C_6D_5Br$, 376 MHz, 298 K): δ −165.8 (t, 8F, *ortho* aryl−*F*), −162.1 (t, 4F, *para* aryl−*F*), −131.4, (d, 8F, *meta* aryl−*F*) ppm. **$^{13}$C NMR** ($C_6D_5Br$, 101 MHz, 298 K): δ 184.9 (s, *C*CH*C*), 148.9 (d, $J_{CF}$ = 244 Hz, B(*C₆*$F_5)_4^-$), 142.7 (s, aryl−*C*), 138.7 (d, $J_{CF}$ = 244 Hz, B(*C₆*$F_5)_4^-$), 136.8 (d, $J_{CF}$ = 244 Hz, B(*C₆*$F_5)_4^-$), 135.4 (s, aryl−*C*), 130.4 (s, aryl−*C*), 125.1 (s, aryl−*C*), 103.9 (s, C*C*HC), 44.2 (s, *C*($CH_3)_3$), 31.9 (s, C(*C*$H_3)_3$), 29.0 (s, *C*H($CH_3)_2$), 25.4 (s, CH(*C*$H_3)_2$), 22.6 (s, CH(*C*$H_3)_2$), −13.4 (s, Al*C*$H_3$) ppm. Signal for B-C B(*C₆*$F_5)_4^-$ was not observed. **Elemental analysis** Found: C, 58.92; H, 4.78; N, 2.24. Calc. for $C_{60}H_{56}AlBF_{20}N_2$: C, 58.93; H, 4.62; N, 2.29%.

**Synthesis of [($^{Me,DIPP}$BDI)AlH$^+$][B($C_6F_5)_4^-$]:** ($^{Me,DIPP}$BDI)AlH$_2$ (48 mg, 107.5 µmol, 1.05 eq.) was dissolved in chlorobenzene (1 mL) and subsequently [$CPh_3^+$][B($C_6F_5)_4^-$] (94 mg, 102.4 µmol, 1 eq.) was added. The orange solution was stirred at room temperature for 6 h after which a color change to colorless was

observed. The addition of hexane (1 mL) resulted in formation of an orange clathrate which complicated further purification. Washing with hexane (5 x 2 mL) resulted in solidification. The product [($^{Me,DIPP}$BDI)AlH$^+$][B($C_6F_5$)$_4^-$] was finally obtained as a white solid in 76% yield (87 mg). **$^1$H NMR** ($C_6D_5Br$, 600 MHz, 298 K): δ 7.27 (t, $^3J_{HH}$ = 7.8 Hz, 2H, aryl−*H*), 7.10 (d, $^3J_{HH}$ = 7.8 Hz, 4H, aryl−*H*), 5.28 (s, 1H, CC*H*C), 4.41 (br s, 1H, Al*H*$^+$), 2.89 (sept, $^3J_{HH}$ = 6.7 Hz, 4H, C*H*($CH_3$)$_2$), 1.66 (s, 6H, $^{Me}$BDI), 1.03 (2x br. d, 24H, CH(C*H*$_3$)$_2$) ppm. **$^{11}$B NMR** ($C_6D_5Br$, 193 MHz, 298 K): δ −15.9 (s, 1B, *B*($C_6F_5$)$_4^-$) ppm. **$^{13}$C NMR** ($C_6D_5Br$, 101 MHz, 298 K): δ 175.9 (s, *C*CH*C*), 144.0 (s, aryl−*C*), 135.1 (s, aryl−*C*), 130.3 (s, aryl−*C*), 126.3 (s, aryl−*C*), 101.4 (s, C*C*HC), 29.4 (s, *C*H($CH_3$)$_2$), 25.2 (s, CH(*C*H$_3$)$_2$), 24.9 (s, CH(*C*H$_3$)$_2$ 23.7 (s, $^{Me}$BDI) ppm. Signal for B-C B(*C*$_6$F$_5$)$_4^-$ was not observed. **$^{19}$F NMR** ($C_6D_5Br$, 565 MHz, 298 K): δ −165.7 (br s, 8F, *meta* aryl−*F*), −161.9 (br s, 4F, *para* aryl−*F*), −131.4 (br s, 8F, *ortho* aryl−*F*) ppm. Due to purification problems of the clathrate-like substance, a satisfying elemental analysis could not be obtained.

**Synthesis of [($^{Me,DIPeP}$BDI)AlMe$^+$][B($C_6F_5$)$_4^-$]:** ($^{Me,DIPeP}$BDI)AlMe$_2$ (49.6 mg, 84.5 µmol, 1.05 eq.) was dissolved in chlorobenzene (2 mL). [$Ph_3C^+$][B($C_6F_5$)$_4^-$] (74.2 mg, 80.5 µmol, 1 eq.) was added and the reaction mixture was stirred for 12 h at room temperature. After layering the solution with hexane (6 mL) a dark orange oil formed. The mother liquor was removed and the oil was washed with hexane (3 x 2 mL) until solidification. Drying in vacuum gave [($^{Me,DIPeP}$BDI)AlMe$^+$][B($C_6F_5$)$_4^-$] as a beige powder in 65% yield (65.0 mg). **$^1$H NMR** ($C_6D_5Br$, 600 MHz, 298 K): δ 7.15 (t, $^3J_{HH}$ = 7.8 Hz, 2H, aryl−*H*), 7.00 (d, $^3J_{HH}$ = 7.8 Hz, 4H, aryl−*H*), 5.64 (s, 1H, CC*H*C), 2.14 (pent, $^3J_{HH}$ = 6.3 Hz, 4H, C*H*($CH_2CH_3$)$_2$), 1.78 (s, 6H, *Me*BDI), 1.41 (m, 16H, CH(C*H*$_2$CH$_3$)$_2$), 0.72 (t, $^3J_{HH}$ = 7.4 Hz, 12H, CH(CH$_2$C*H*$_3$)$_2$), 0.66 (t, $^3J_{HH}$ = 7.4 Hz, 12H, CH(CH$_2$C*H*$_3$)$_2$), −0.64 (s, 3H, Al*Me*$^+$) ppm. **$^{11}$B NMR** ($C_6D_5Br$, 193 MHz, 298 K): δ −15.6 (s, 1B, *B*($C_6F_5$)$_4^-$) ppm. **$^{13}$C NMR** ($C_6D_5Br$, 151 MHz, 298 K): δ 177.3 (s, *C*CH*C*), 149.2 (br. d, $^1J_{CF}$ = 244 Hz, B($C_6F_5$)$_4^-$),139.8 (s, aryl−*C*), 138.7 (br. d, $^1J_{CF}$ = 244 Hz, B(*C*$_6$F$_5$)$_4^-$), 136.7 (s, aryl−*C*), 135.8 (br. d, $^1J_{CF}$ = 244 Hz, B(*C*$_6$F$_5$)$_4^-$), 129.8 (s, aryl−*C*), 127.0 (s, aryl−*C*), 104.1 (s, C*C*HC), 43.9 (s, *Me*BDI), 29.0 (s, *C*H($CH_2CH_3$)$_2$), 26.9 (s, CH($CH_2CH_3$)$_2$), 23.8 (s, CH(*C*H$_2$CH$_3$)$_2$), 12.2 (s, CH(CH$_2$*C*H$_3$)$_2$), 12.2 (s, CH(CH$_2$*C*H$_3$)$_2$) ppm. Signals for Al*Me*$^+$ and B-C B(*C*$_6$F$_5$)$_4^-$ were not observed. **$^{19}$F NMR** ($C_6D_5Br$, 565 MHz, 298 K): δ −165.4 (br s, 8F, *meta* aryl−*F*), −161.6 (br s, 4F, *para* aryl−*F*), −131.0 (br s, 8F, *ortho* aryl−*F*) ppm. **Elemental analysis** Found: C, 58.53; H, 4.77; N, 2.11. Calc. for $C_{62}H_{60}AlBF_{20}N_2$: C, 59.53; H, 4.83; N, 2.24%. Although these results are outside the range viewed as established analytically pure, they are provided to illustrate the best values obtained to date.

**Synthesis of [($^{tBu,DIPeP}$BDI)AlMe$^+$][B($C_6F_5$)$_4^-$]:** [$Ph_3C^+$][B($C_6F_5$)$_4^-$] (65 mg, 71.0 µmol, 1 eq.) was added to a solution of ($^{tBu,DIPeP}$BDI)AlMe$_2$ (50 mg, 74.5 µmol, 1.05 eq.) in chlorobenzene (1.5 mL). After stirring for 1 min the reaction mixture was layered with hexane (2 mL). After 2 d colorless crystals formed which were collected and washed with hexane to give [($^{tBu,DIPeP}$BDI)AlMe$^+$][B($C_6F_5$)$_4^-$] in 97% yield (92 mg). Crystals suitable for single crystal X-ray analysis were obtained from the washing solution. **$^1$H NMR**

($C_6D_5Br$, 400 MHz, 298 K): δ 7.18 (t, $^3J_{HH}$ = 7.8 Hz, 2H, aryl–*H*), 6.93 (d$_{\text{(partially covered by C6D5Br)}}$, 4H, aryl–*H*), 6.23 (s, 1H, CC*H*C), 2.40 (br. pent, 4H, C*H*($CH_2CH_3$)$_2$), 1.57 (m, 12H, CH(C$H_2$CH$_3$)$_2$), 1.18 (m, 4H, CH(C$H_2$CH$_3$)$_2$), 1.02 (s, 18H, C(C$H_3$)$_3$), 0.83 (t, $^3J_{HH}$ = 7.3 Hz, 12H, CH(CH$_2$C$H_3$)$_2$), 0.65 (t, $^3J_{HH}$ = 7.3 Hz, 12H, CH(CH$_2$C$H_3$)$_2$), −0.98 (s, 3H, Al*Me*$^+$) ppm. **$^{11}$B NMR** ($C_6D_5Br$, 193 MHz, 298 K): δ −15.5 (s, 1B, *B*($C_6F_5$)$_4^-$) ppm. **$^{13}$C NMR** ($C_6D_6$, 151 MHz, 298 K): δ 185.8 (s, *C*CH*C*), 139.9 (s, aryl–*C*), 137.6 (s, aryl–*C*), 129.1 (s, aryl–*C*), 127.5 (s, aryl–*C*), 105.0 (s, C*C*HC), 44.7 (s, *C*(CH$_3$)$_3$), 42.5 (s, *C*H($CH_2CH_3$)$_2$), 32.6 (s, C(*C*H$_3$)$_3$), 27.7 (s, CH(*C*H$_2$CH$_3$)$_2$), 25.0 (s, CH(*C*H$_2$CH$_3$)$_2$), 12.0 (s, CH(CH$_2$*C*H$_3$)$_2$), 9.9 (s, CH(CH$_2$*C*H$_3$)$_2$) ppm. Signals for Al*Me*$^+$ and B-C B(*C*$_6$F$_5$)$_4^-$ were not observed. **$^{19}$F NMR** ($C_6D_5Br$, 565 MHz, 298 K): δ −165.4 (br s, 8F, *meta* aryl–*F*), −161.6 (br s, 4F, *para* aryl–*F*), −131.0 (br s, 8F, *ortho* aryl–*F*) ppm. **Elemental analysis** Found: C, 61.45; H, 5.64; N, 2.13. Calc. for $C_{68}H_{72}AlBF_{20}N_2$: C, 61.18; H, 5.44; N, 2.10%.

**General procedure for catalytic imine hydrogenation:** Under inert atmosphere a *J. Young*- or a pressure-tube was charged with catalyst (amounts ranged from 1-10 mg) and dissolved in a solvent mixture of PhCl/$C_6D_6$ (0.4 mL/0.2 mL). Depending on the catalyst loading (5 or 10 mol%), the correct amount of imine was added and the solution was freed of $N_2$ gas by freeze-thawing three times. The tube was then pressurized with $H_2$ to the desired pressure and shaken mechanically at the given temperature. All experiments were monitored by $^1$H NMR. Starting from 10 mol% catalyst loading, 1.5 bar $H_2$ and 60°C, the conditions were optimized to give the data summarized in Table 2.

## 1.2. NMR spectra of synthesized compounds

### 1.2.1. Spectra of ($^{tBu,DIPP}$BDI)AlMe$_2$

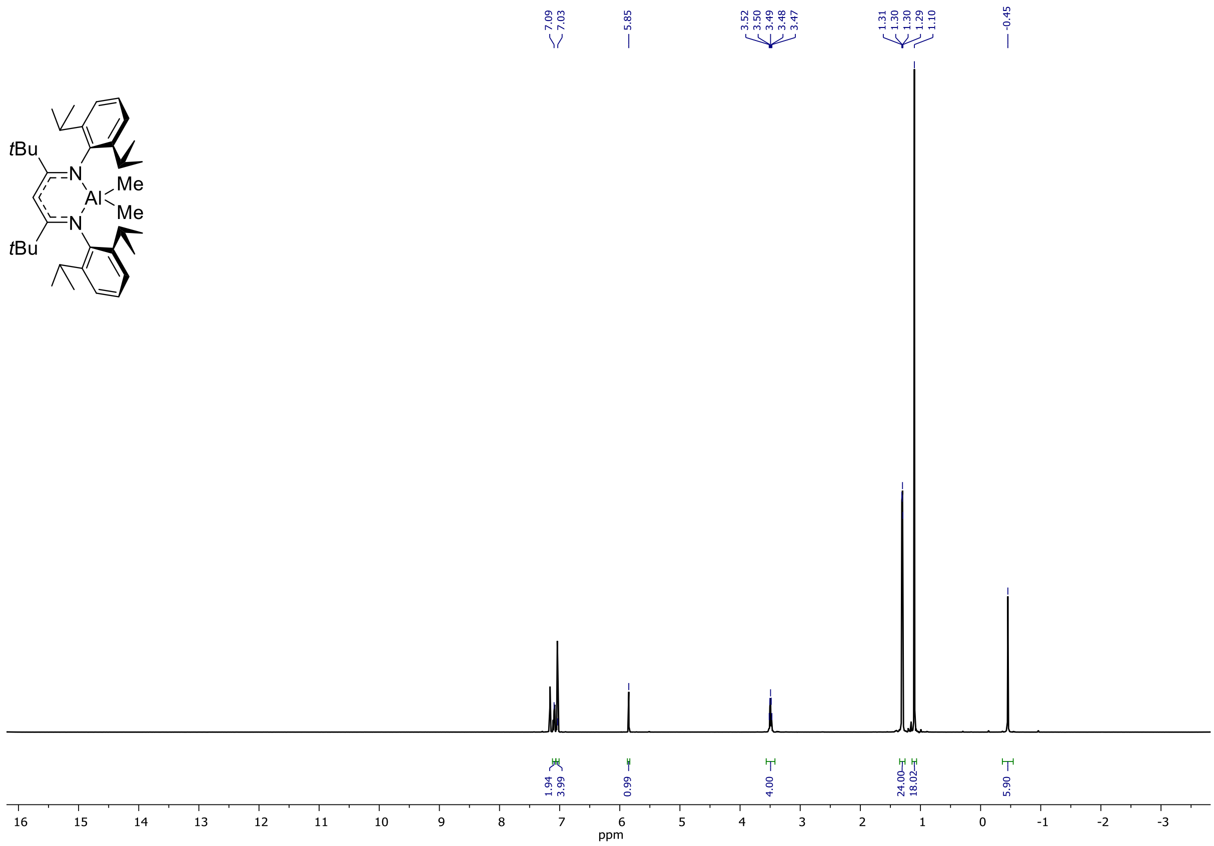


**Figure S1:** $^1$H NMR (600 MHz, 298 K) spectrum of ($^{tBu,DIPP}$BDI)AlMe$_2$ in $C_6D_6$.

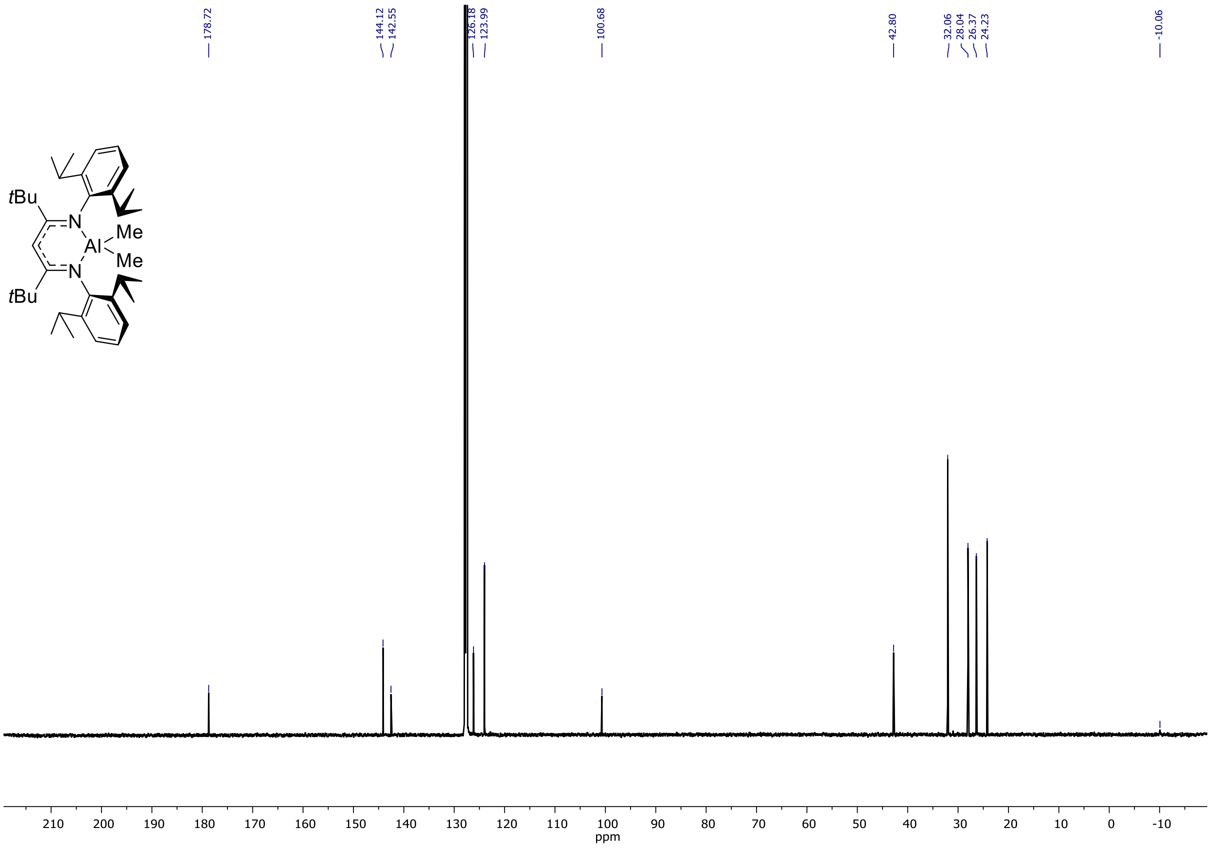


**Figure S2:** $^{13}$C NMR (151 MHz, 298 K) spectrum of ($^{tBu,DIPP}$BDI)AlMe$_2$ in $C_6D_6$.

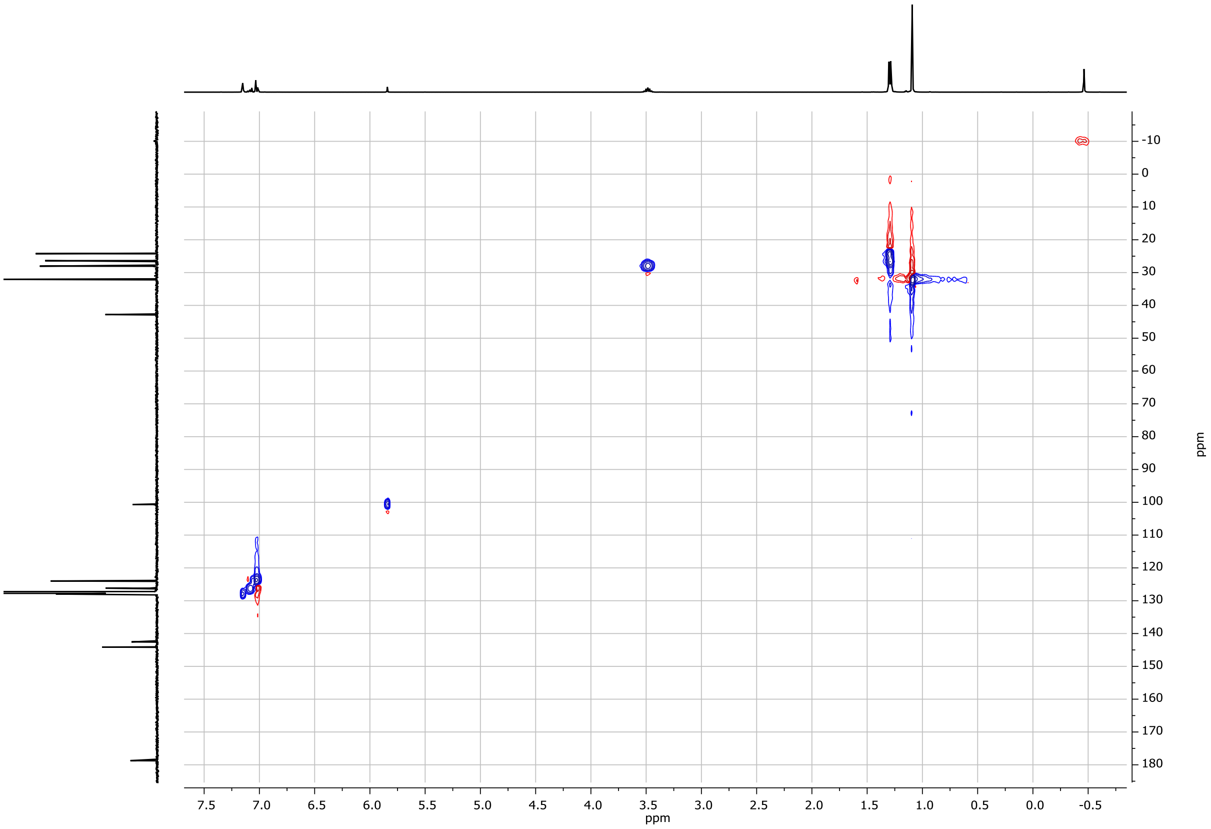


**Figure S3:** 2D-HSQC NMR (298 K) spectrum of ($^{tBu,DIPP}$BDI)AlMe$_2$ in $C_6D_6$.

## 1.2.2. Spectra of ($^{tBu,DIPP}$BDI)AlH$_2$

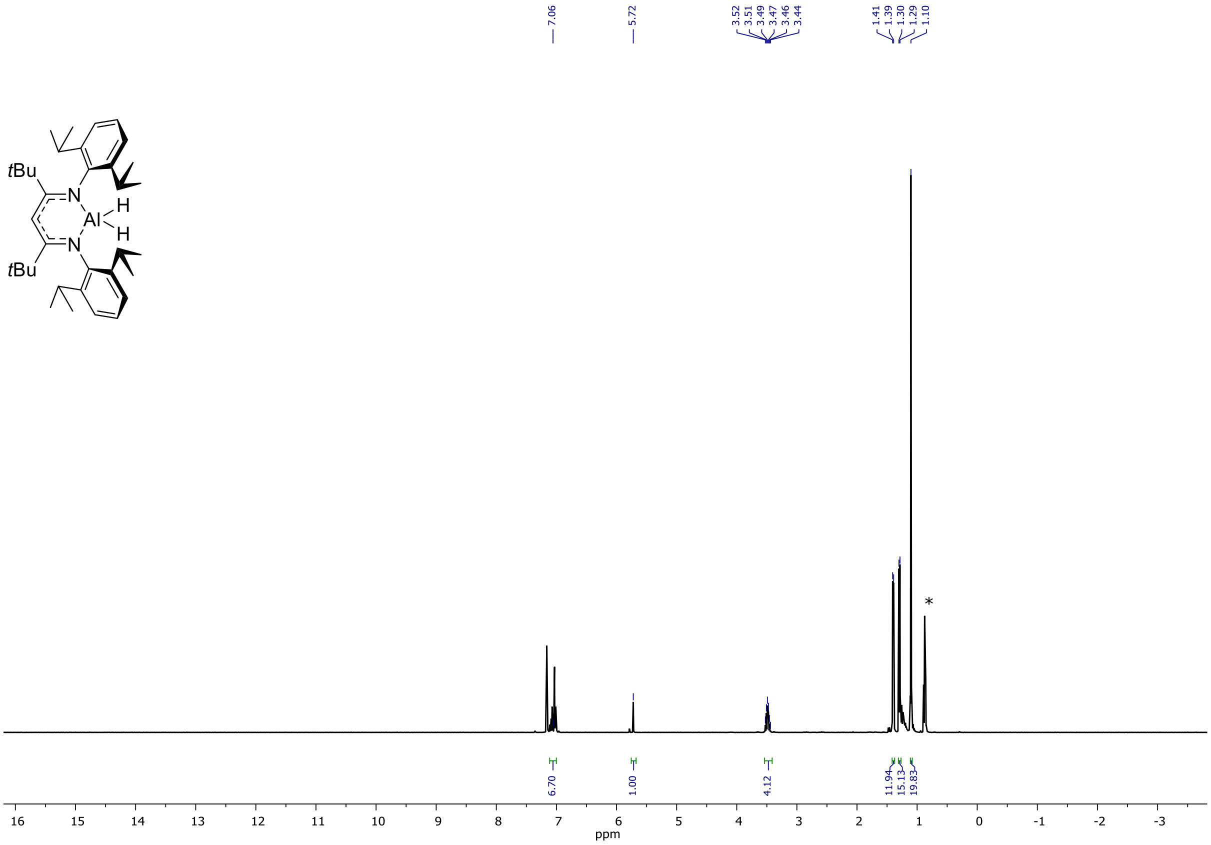

**Figure S4:** $^1$H NMR (600 MHz, 298 K) spectrum of ($^{tBu,DIPP}$BDI)AlH$_2$ in $C_6D_6$.

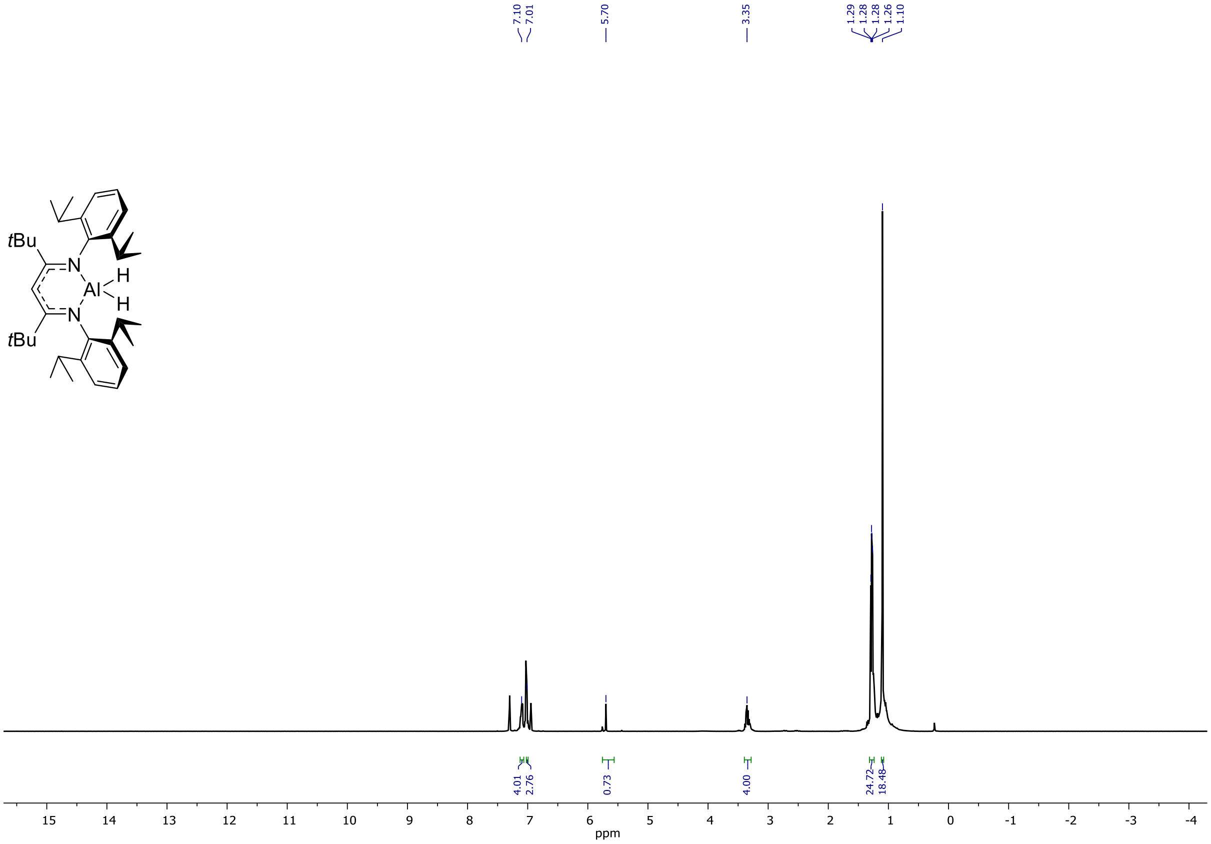

**Figure S5:** $^1$H NMR (400 MHz, 298 K) spectrum of ($^{tBu,DIPP}$BDI)AlH$_2$ in $C_6D_5Br$.

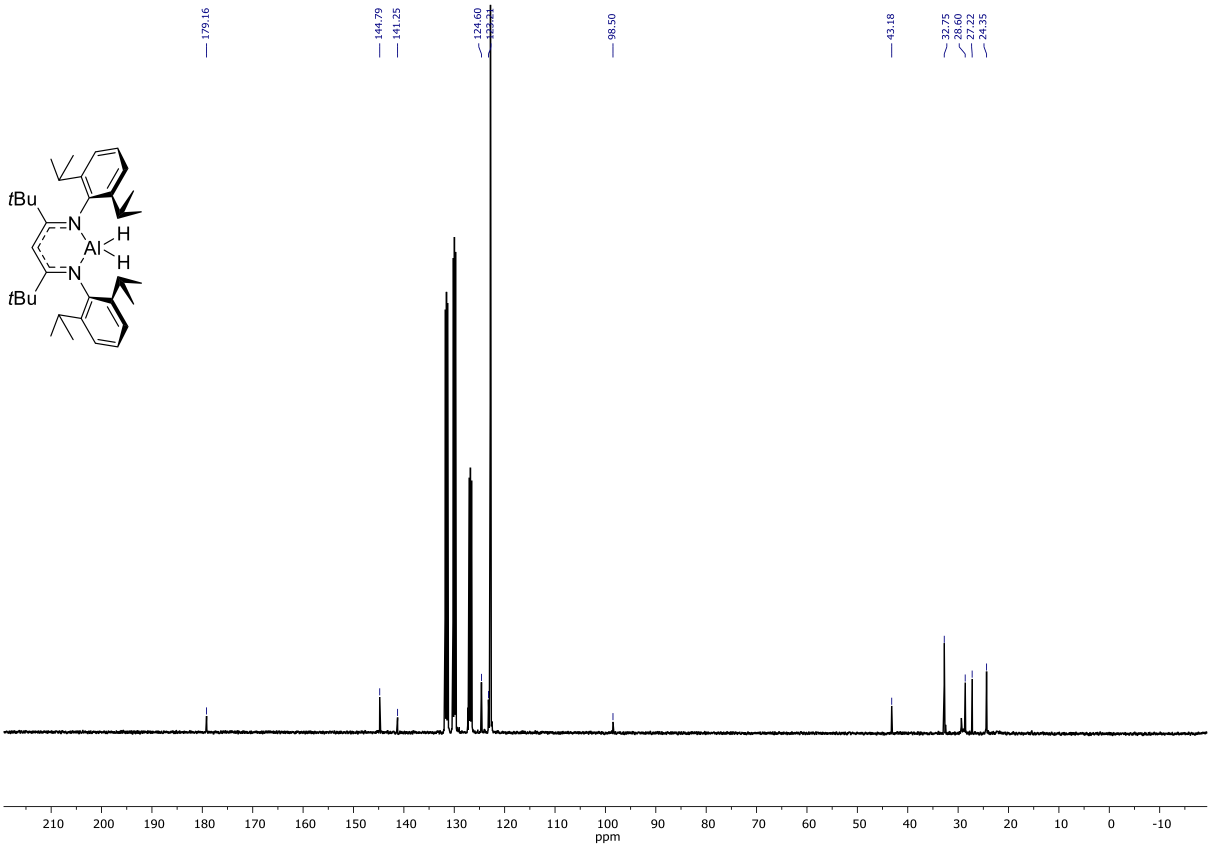


**Figure S6:** $^{13}C$ NMR (151 MHz, 298 K) spectrum of ($^{tBu,DIPP}$BDI)$AlH_2$ in $C_6D_5Br$.

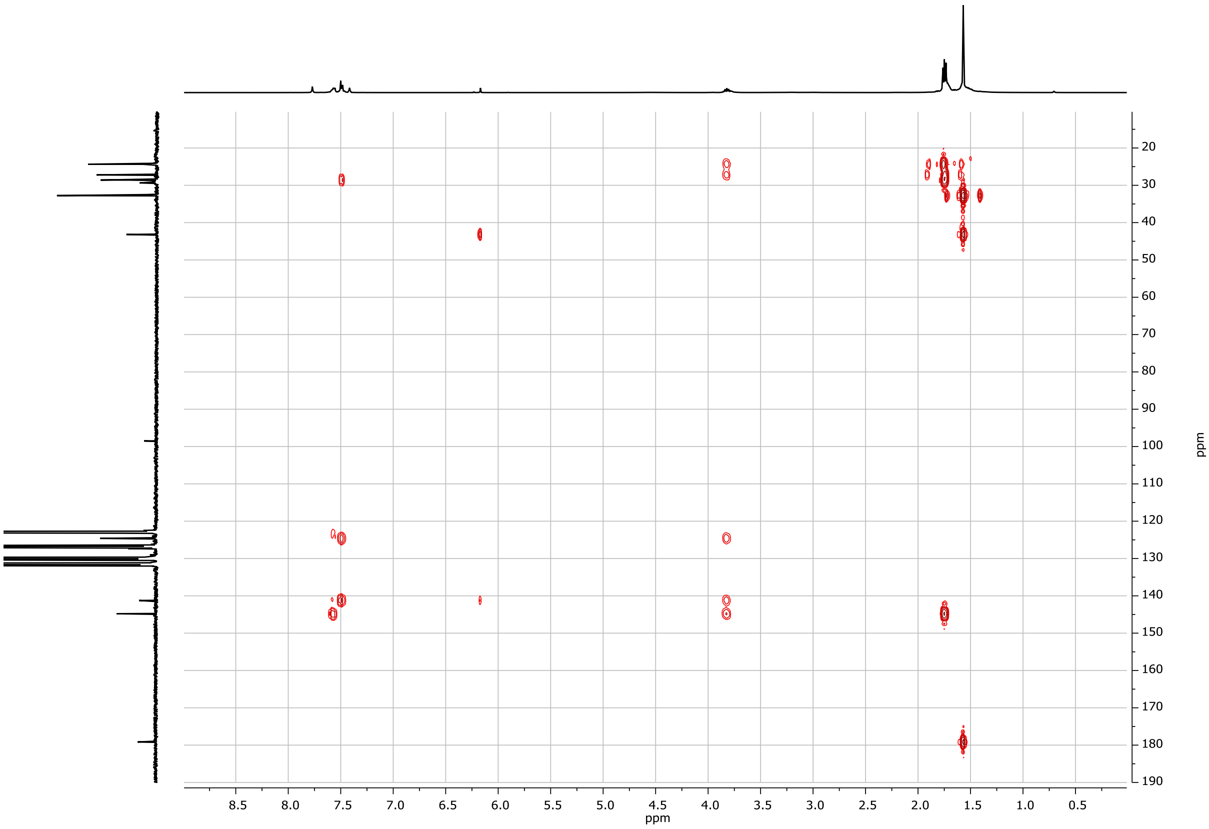


**Figure S7:** 2D HMBC NMR (298 K) spectrum of ($^{tBu,DIPP}$BDI)$AlH_2$ in $C_6D_5Br$.

### 1.2.3. Spectra of ($^{tBu,DIPP}$BDI)AlMeCl

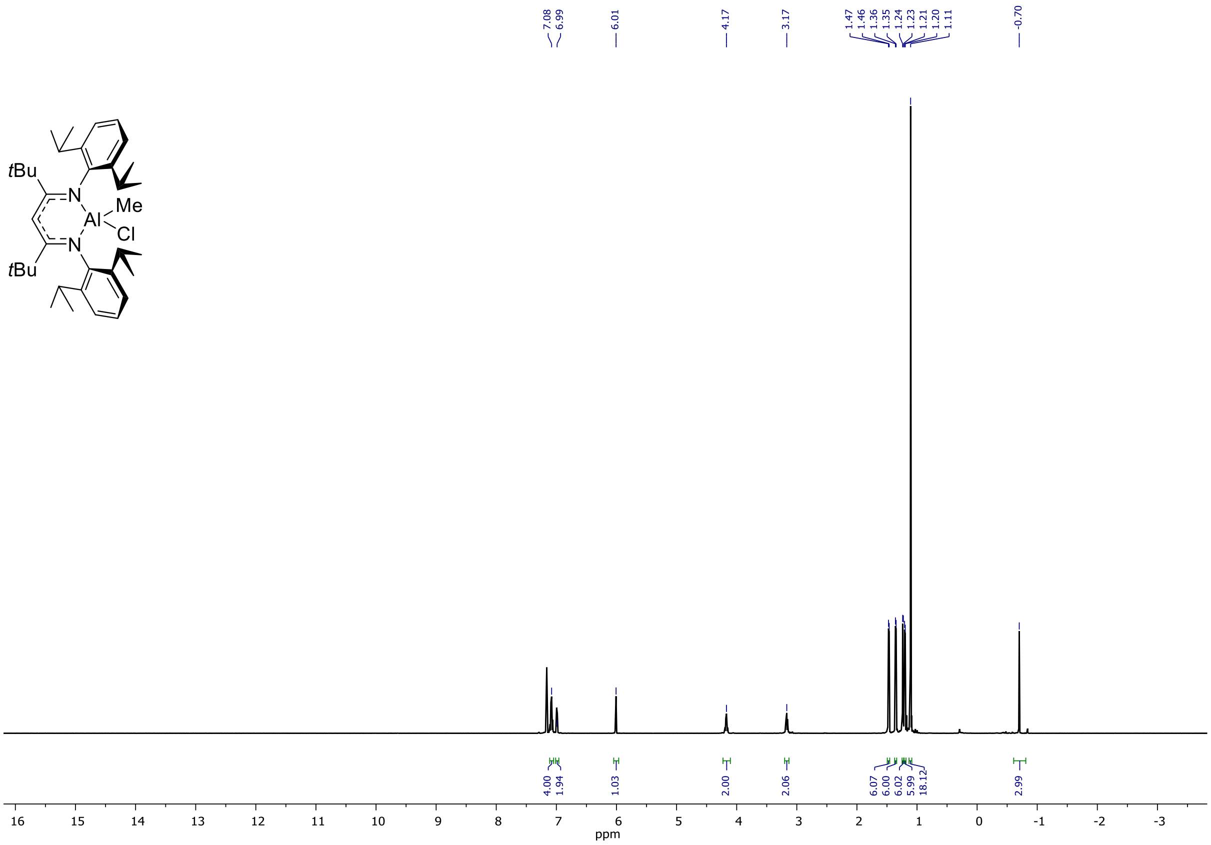


**Figure S8:** $^{1}$H NMR (600 MHz, 298 K) spectrum of ($^{tBu,DIPP}$BDI)AlMeCl in $C_6D_6$.

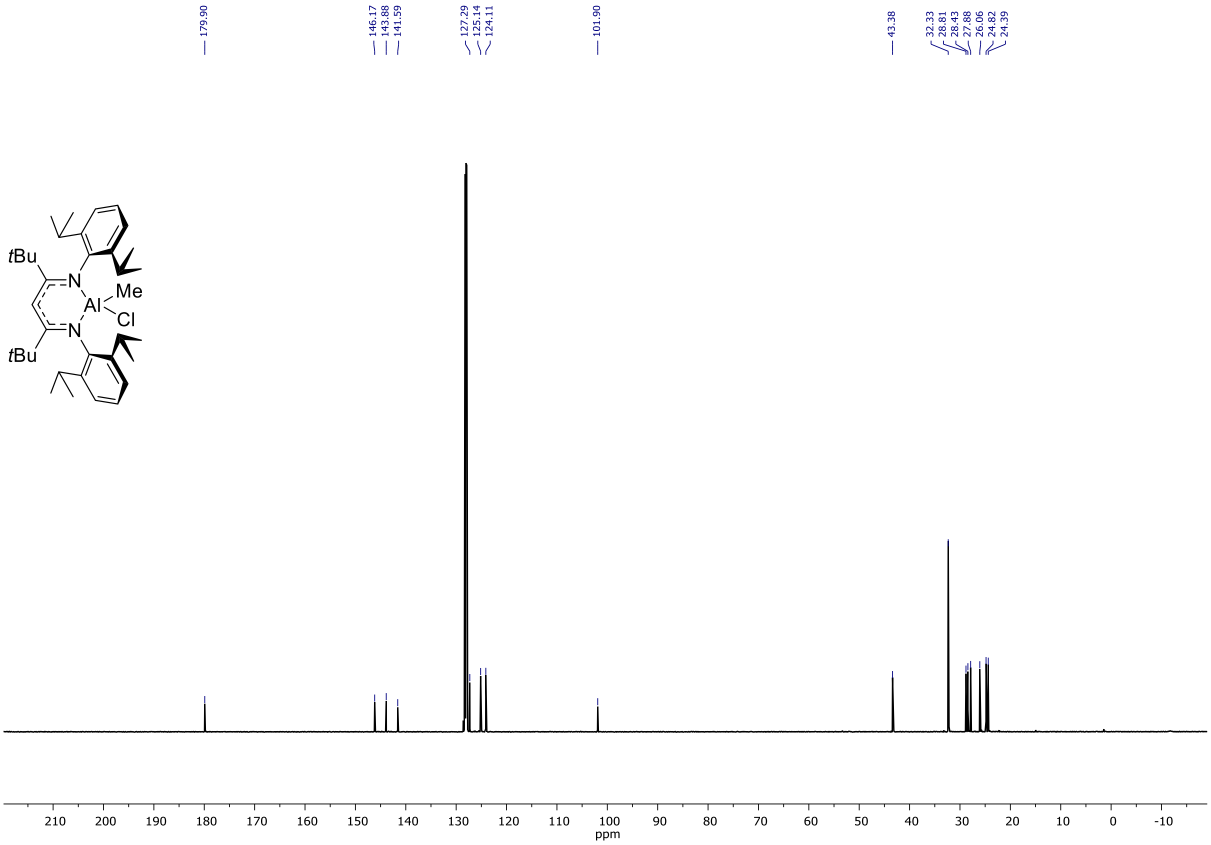


**Figure S9:** $^{13}$C NMR (151 MHz, 298 K) spectrum of ($^{tBu,DIPP}$BDI)AlMeCl in $C_6D_6$.

### 1.2.4. Spectra of ($^{Me,DIPeP}$BDI)AlMe$_2$

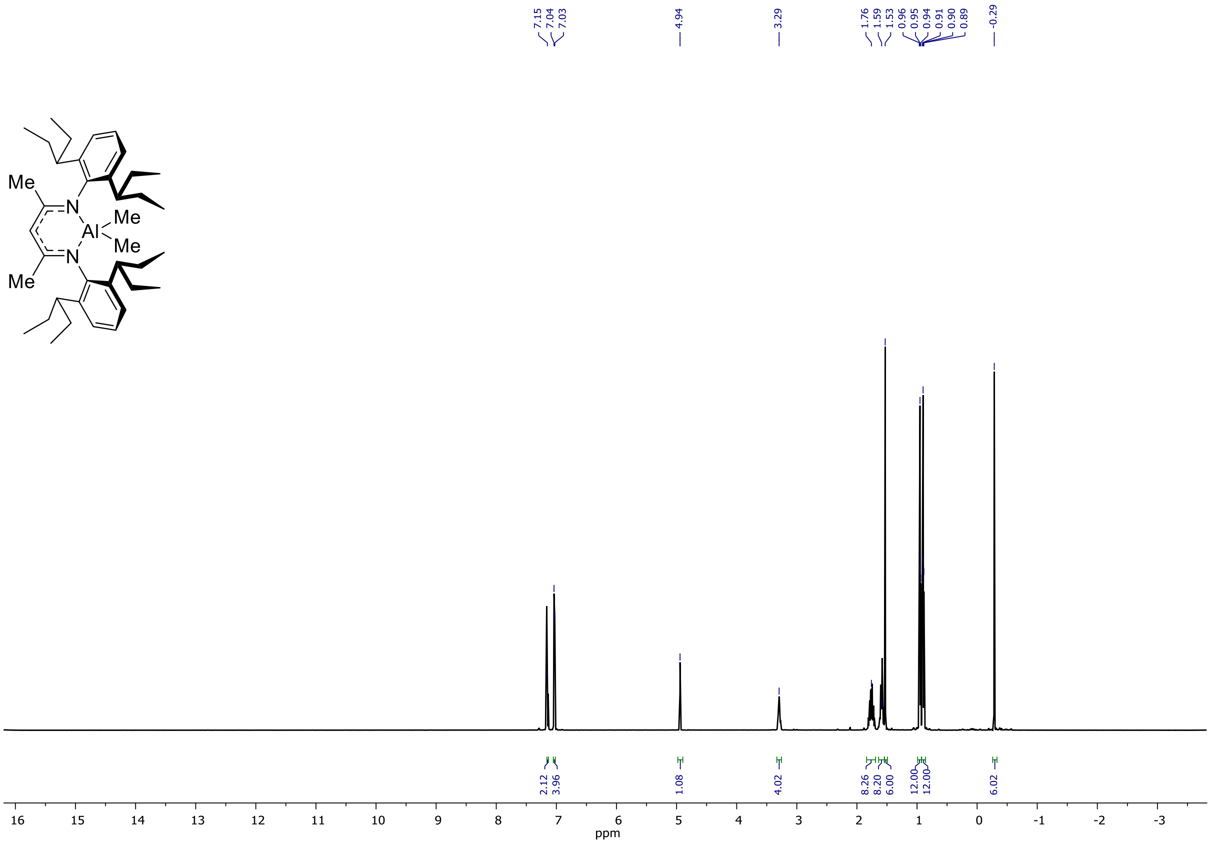


**Figure S10:** $^{1}$H NMR (600 MHz, 298 K) spectrum of ($^{Me,DIPeP}$BDI)AlMe$_2$ in $C_6D_6$.

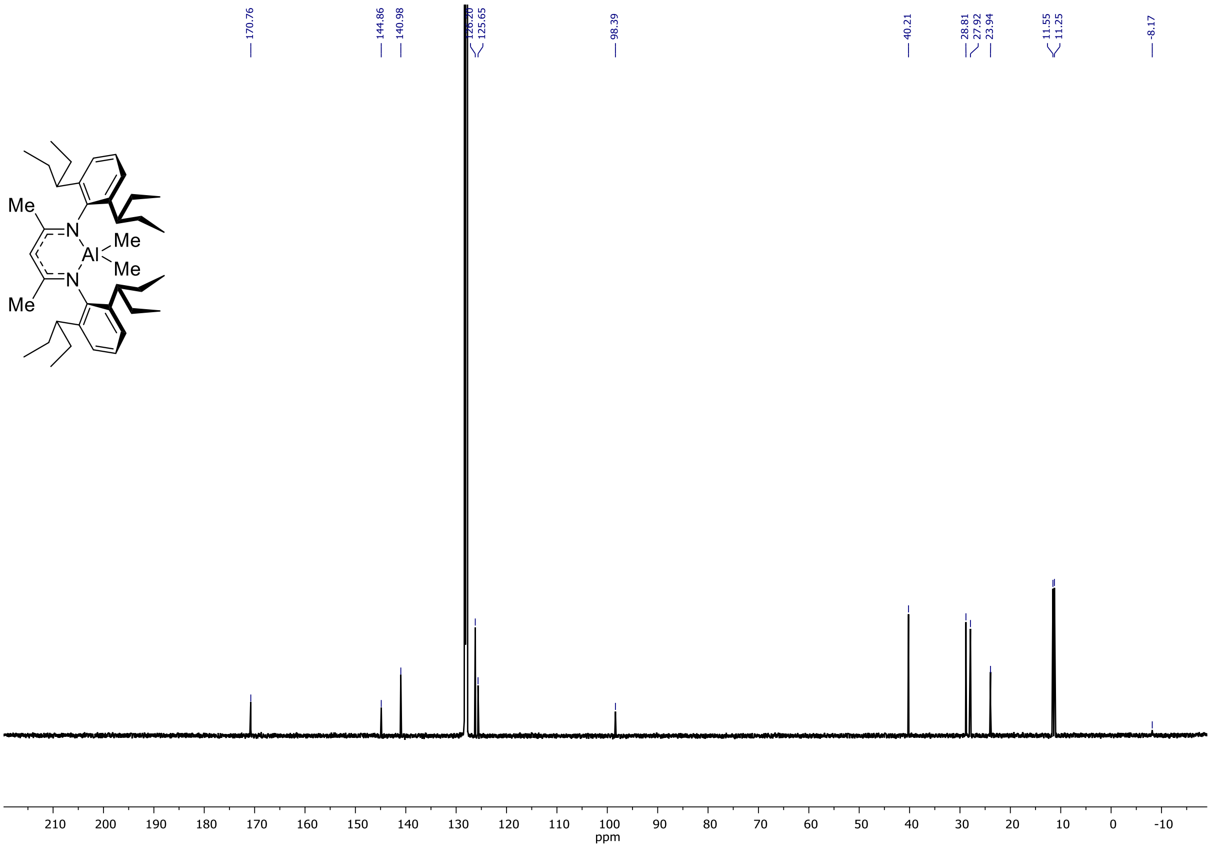


**Figure S11:** $^{13}$C NMR (151 MHz, 298 K) spectrum of ($^{Me,DIPeP}$BDI)AlMe$_2$ in $C_6D_6$.

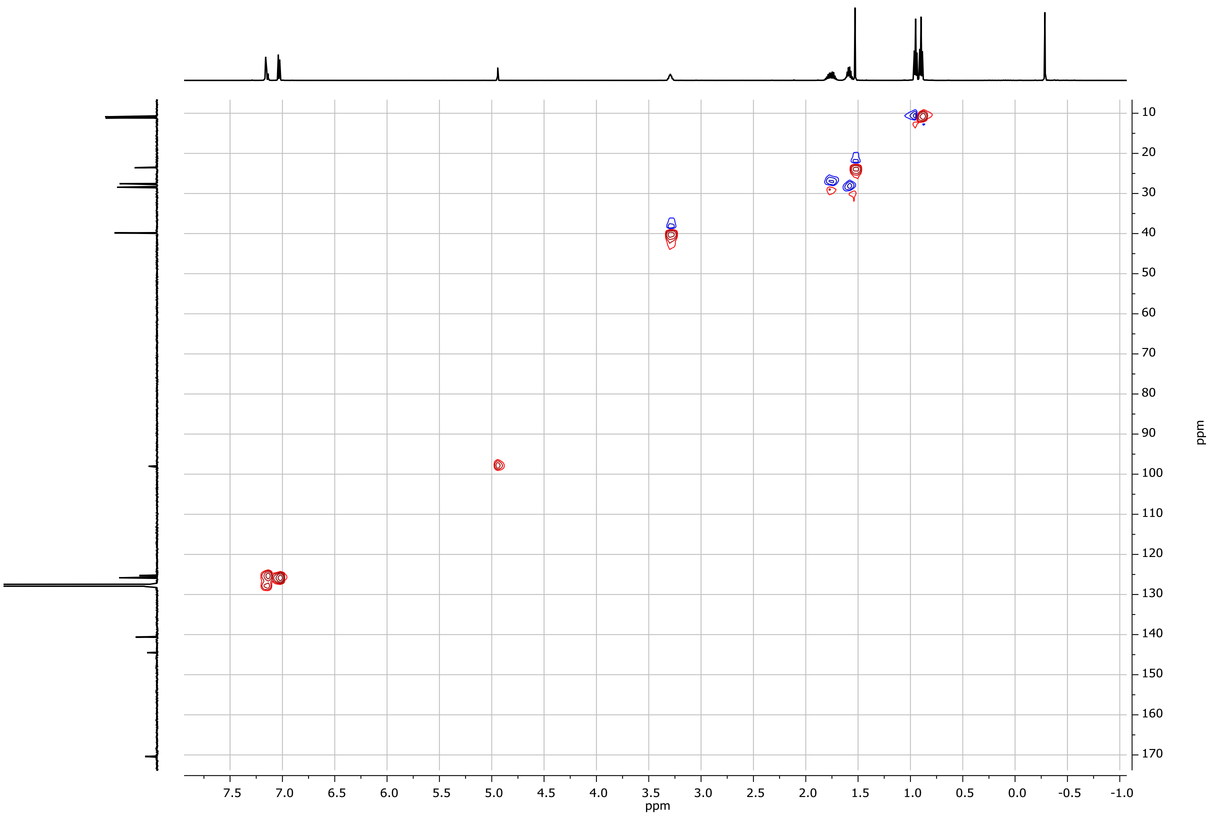

**Figure S12:** 2D HSQC NMR (298 K) spectrum of ($^{Me,DIPeP}$BDI)AlMe$_2$ in $C_6D_6$.

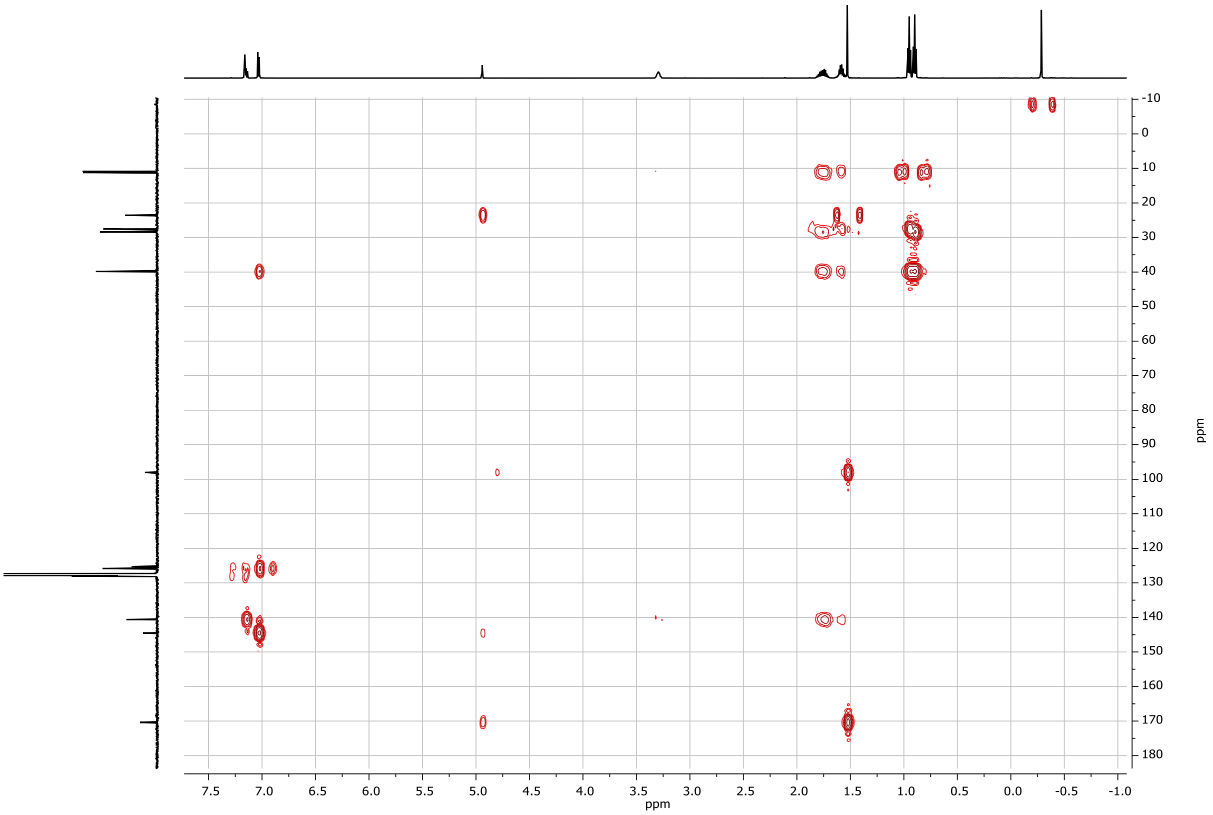

**Figure S13:** 2D HMBC NMR (298 K) spectrum of ($^{Me,DIPeP}$BDI)AlMe$_2$ in $C_6D_6$

### 1.2.5. Spectra of ($^{tBu,DIPeP}$BDI)AlMe$_2$

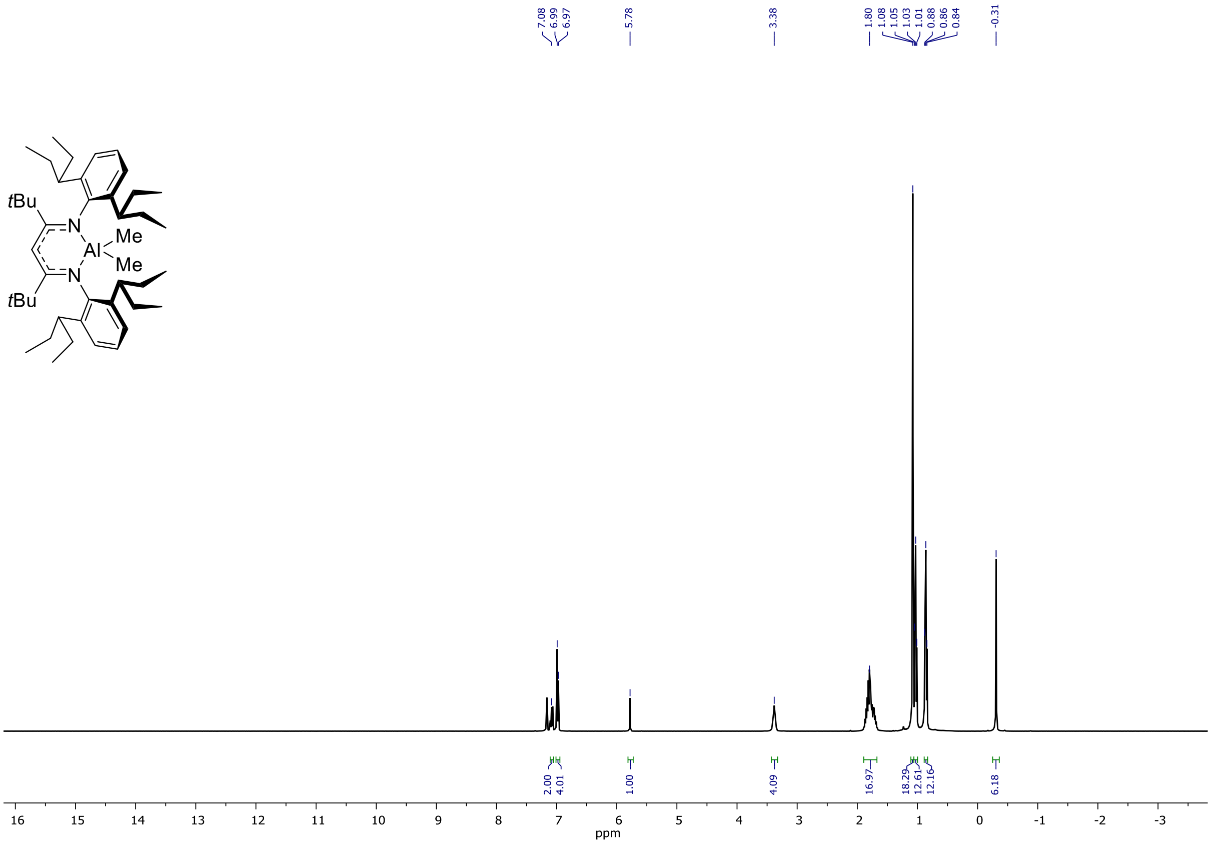


**Figure S14:** $^{1}$H NMR (400 MHz, 298 K) spectrum of ($^{tBu,DIPeP}$BDI)AlMe$_2$ in $C_6D_6$.

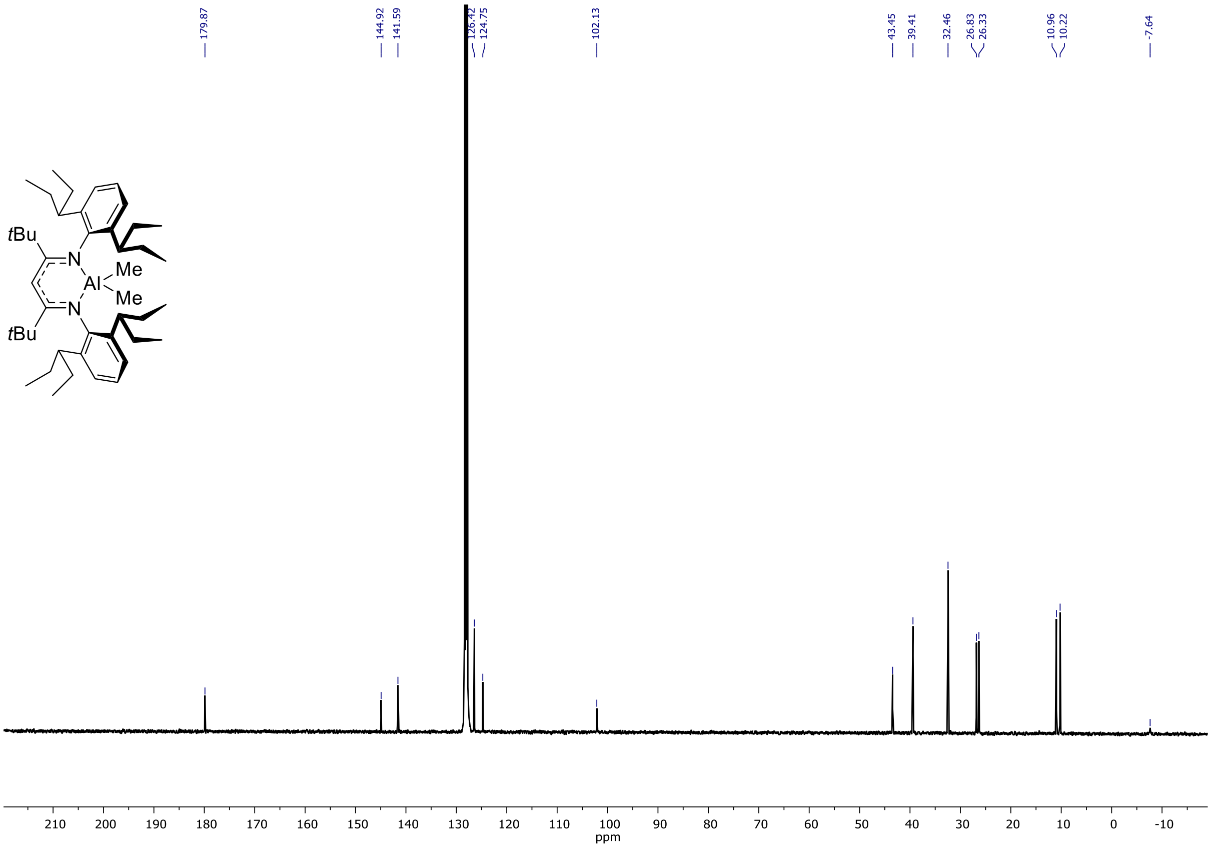


**Figure S15:** $^{13}$C NMR (101 MHz, 298 K) spectrum of ($^{tBu,DIPeP}$BDI)AlMe$_2$ in $C_6D_6$.

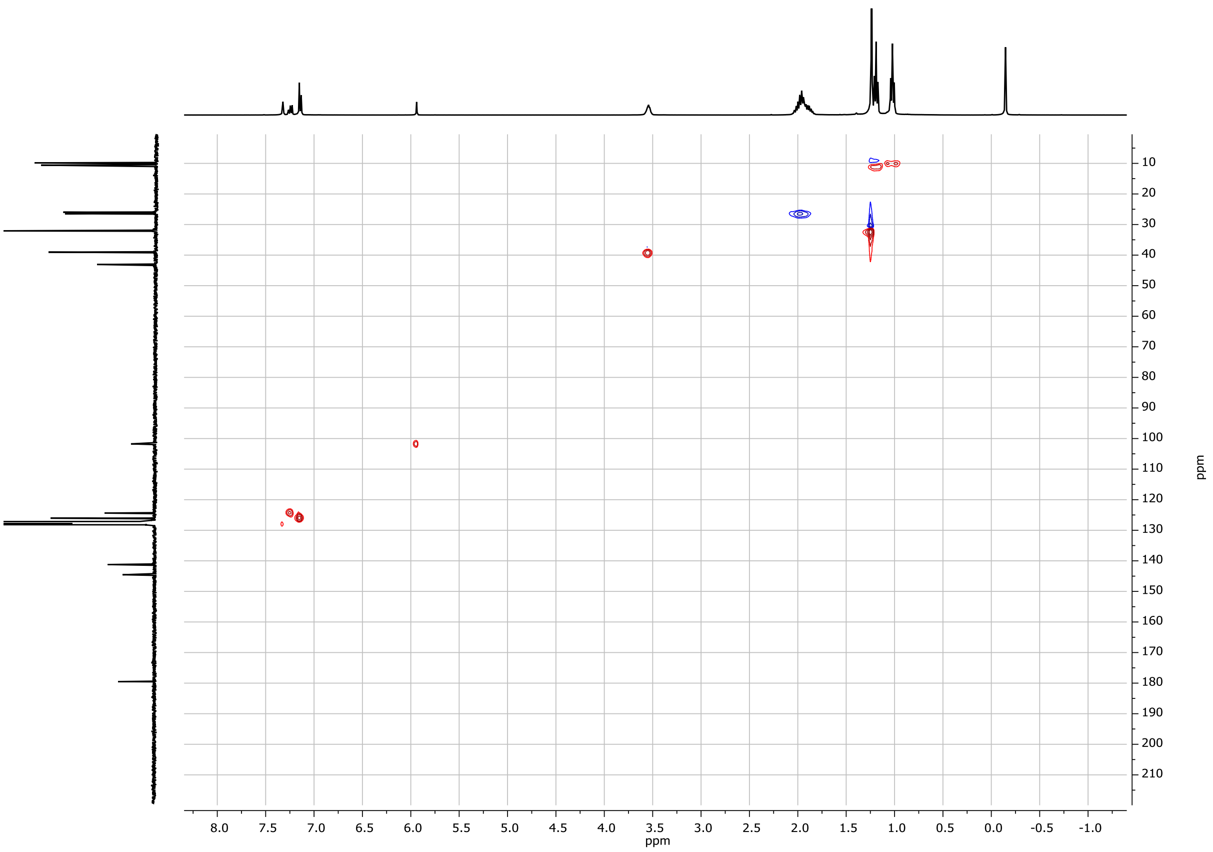

**Figure S16:** 2D-HSQC NMR (298 K) spectrum of ($^{tBu,DIPeP}$BDI)$AlMe_2$ in $C_6D_6$.

### 1.2.6. Spectra of [($^{tBu,DIPP}$BDI)$AlMe^+$][$B(C_6F_5)_4^-$]

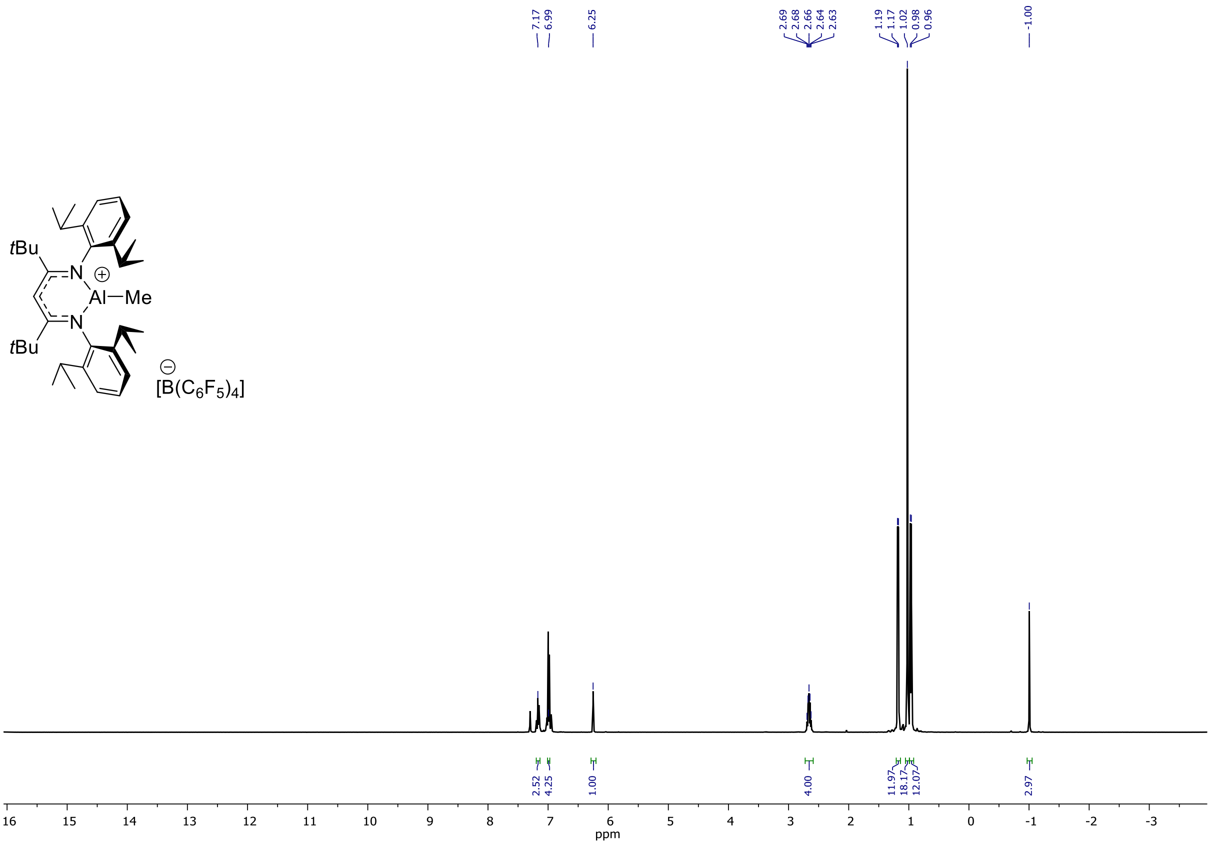


**Figure S17:** $^1$H NMR (400 MHz, 298 K) spectrum of [($^{tBu,DIPP}$BDI)$AlMe^+$][$B(C_6F_5)_4^-$] in $C_6D_5Br$.

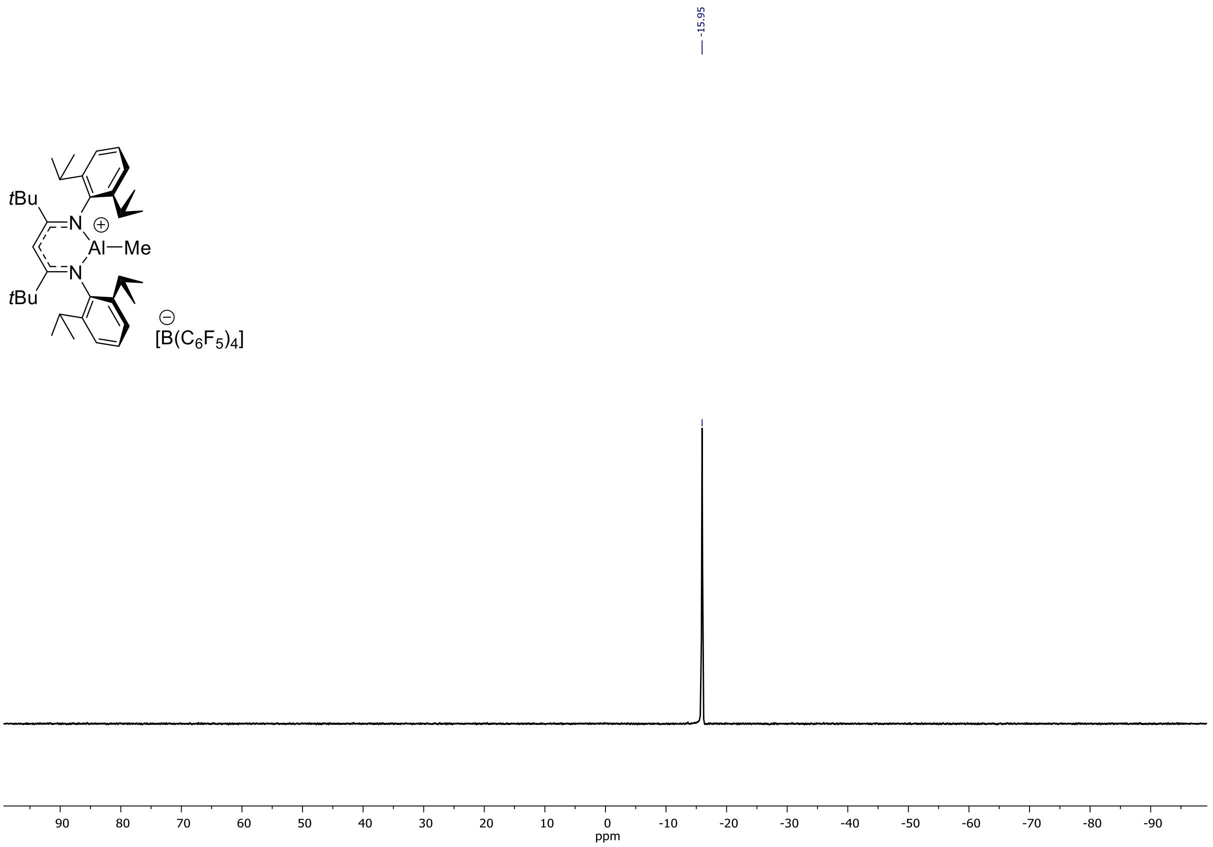


**Figure S18:** $^{11}$B NMR (128 MHz, 298 K) spectrum of [($^{tBu,DIPP}$BDI)AlMe$^{+}$][B(C$_6$F$_5$)$_4$$^{-}$] in C$_6$D$_5$Br.

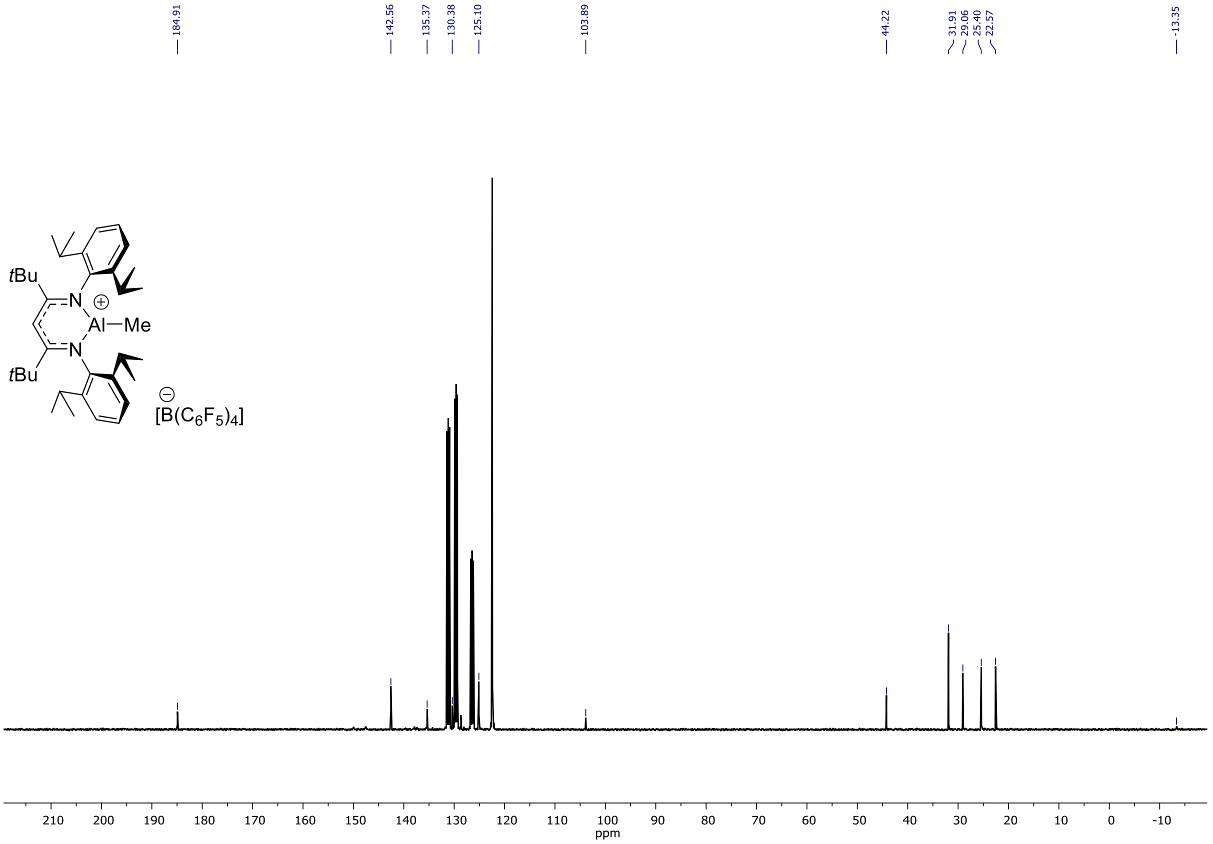


**Figure S19:** $^{13}$C NMR (101 MHz, 298 K) spectrum of [($^{tBu,DIPP}$BDI)AlMe$^{+}$][B(C$_6$F$_5$)$_4$$^{-}$] in C$_6$D$_5$Br.

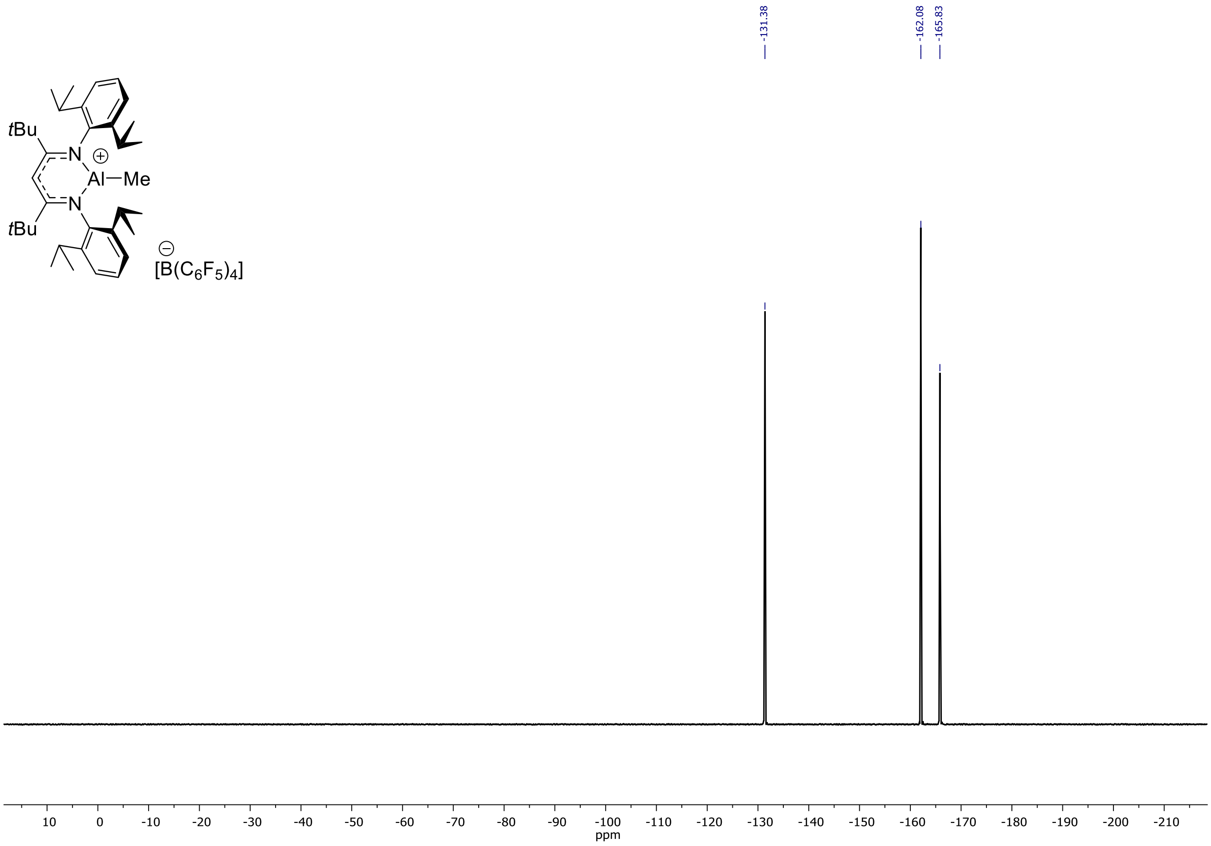


**Figure S20:** $^{19}$F NMR (376 MHz, 298 K) spectrum of [($^{tBu,DIPP}$BDI)AlMe$^+$][B($C_6F_5$)$_4$$^-$] in $C_6D_5Br$.

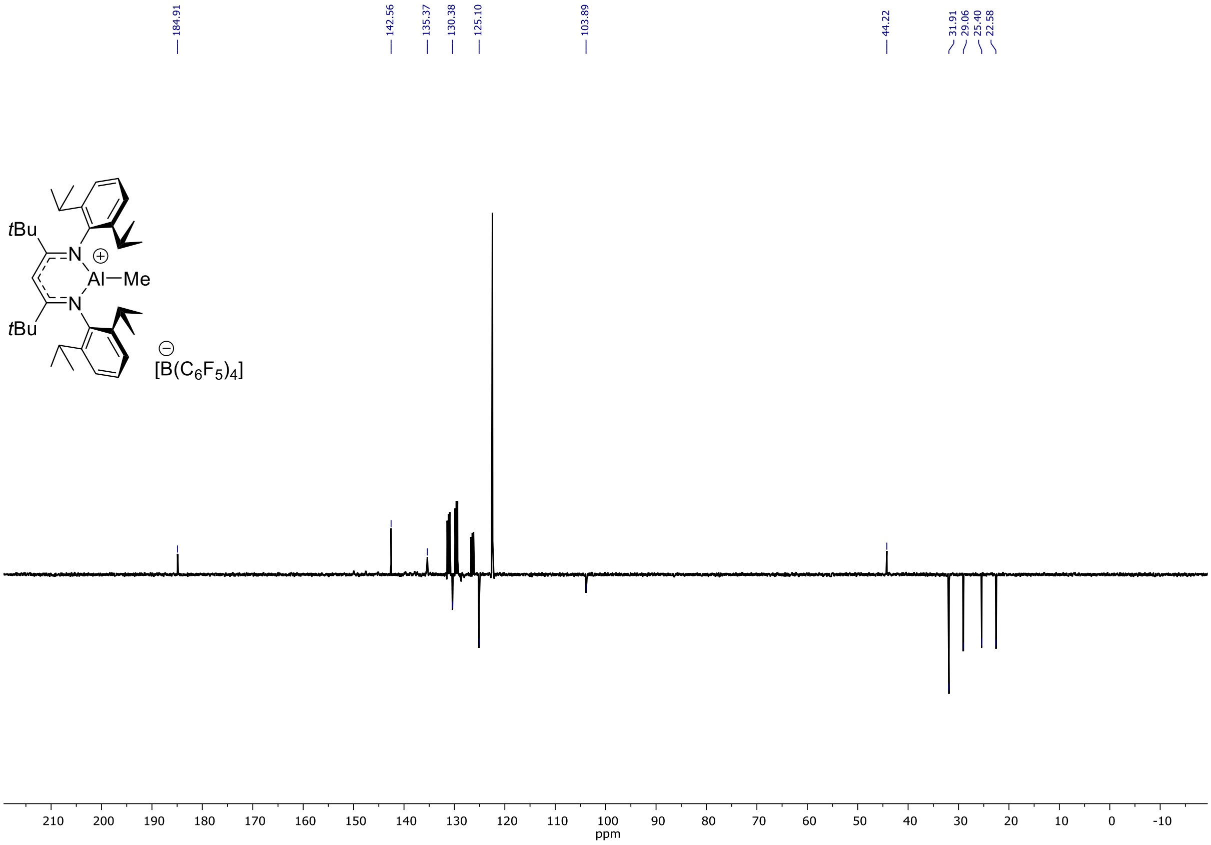


**Figure S21:** $^{13}$C APT NMR (298 K) spectrum of [($^{tBu,DIPP}$BDI)AlMe$^+$][B($C_6F_5$)$_4$$^-$] in $C_6D_5Br$.

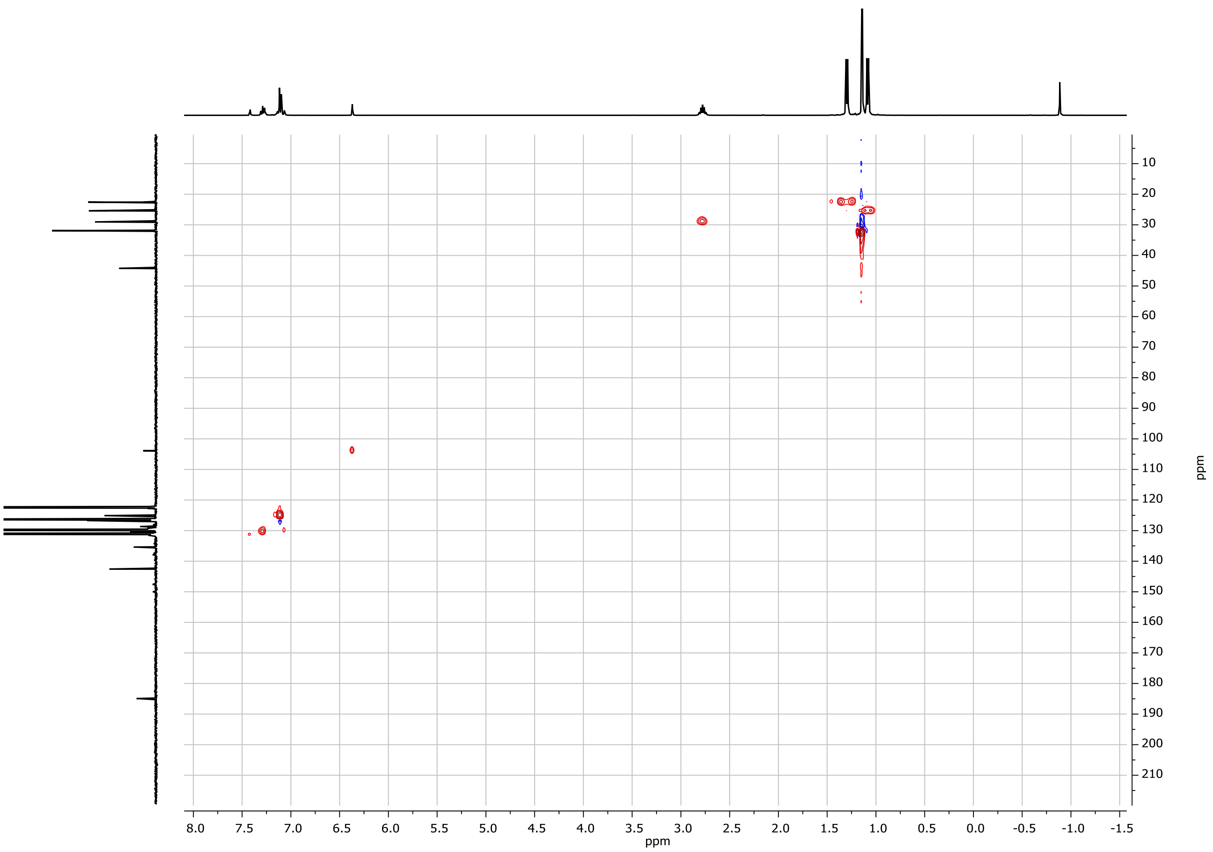

**Figure S22:** 2D HSQC NMR (298 K) spectrum of [($^{tBu,DIPP}$BDI)AlMe$^+$][B($C_6F_5$)$_4^-$] in $C_6D_5Br$.

### 1.2.7. Spectra of [($^{Me,DIPP}$BDI)AlH$^+$][B($C_6F_5$)$_4^-$]

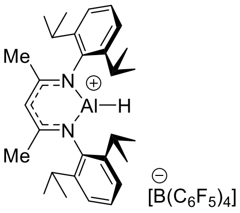

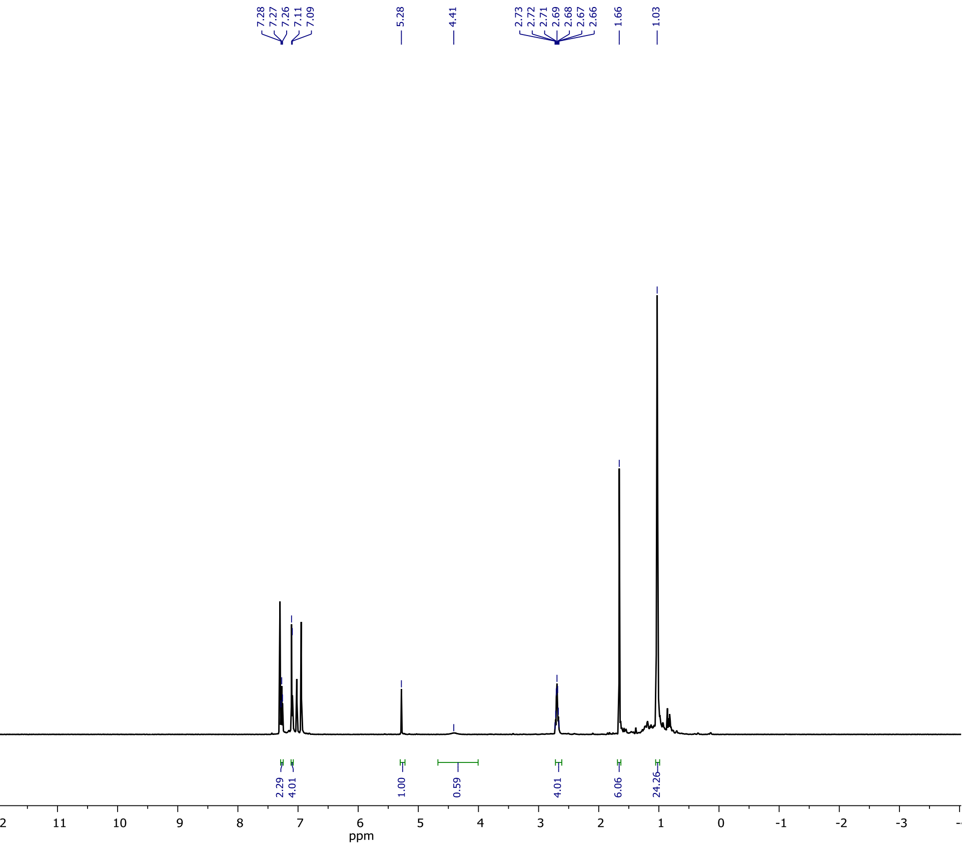

**Figure S23:** $^1$H NMR (600 MHz, 298 K) spectrum of [($^{Me,DIPP}$BDI)AlH$^+$][B($C_6F_5$)$_4^-$] in $C_6D_5Br$.

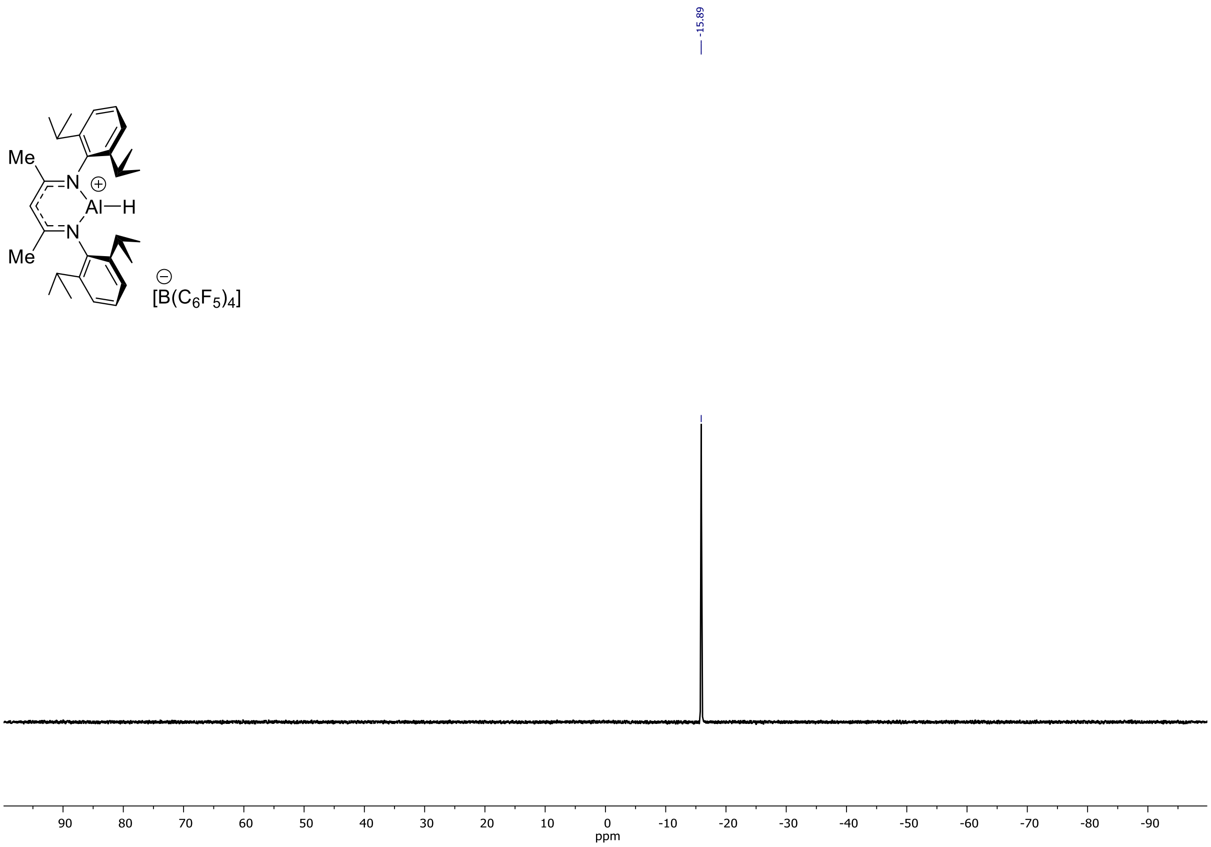


**Figure S24:** $^{11}$B NMR (193 MHz, 298 K) spectrum of [($^{Me,DIPP}$BDI)AlH$^+$][B($C_6F_5$)$_4^-$] in $C_6D_5Br$.

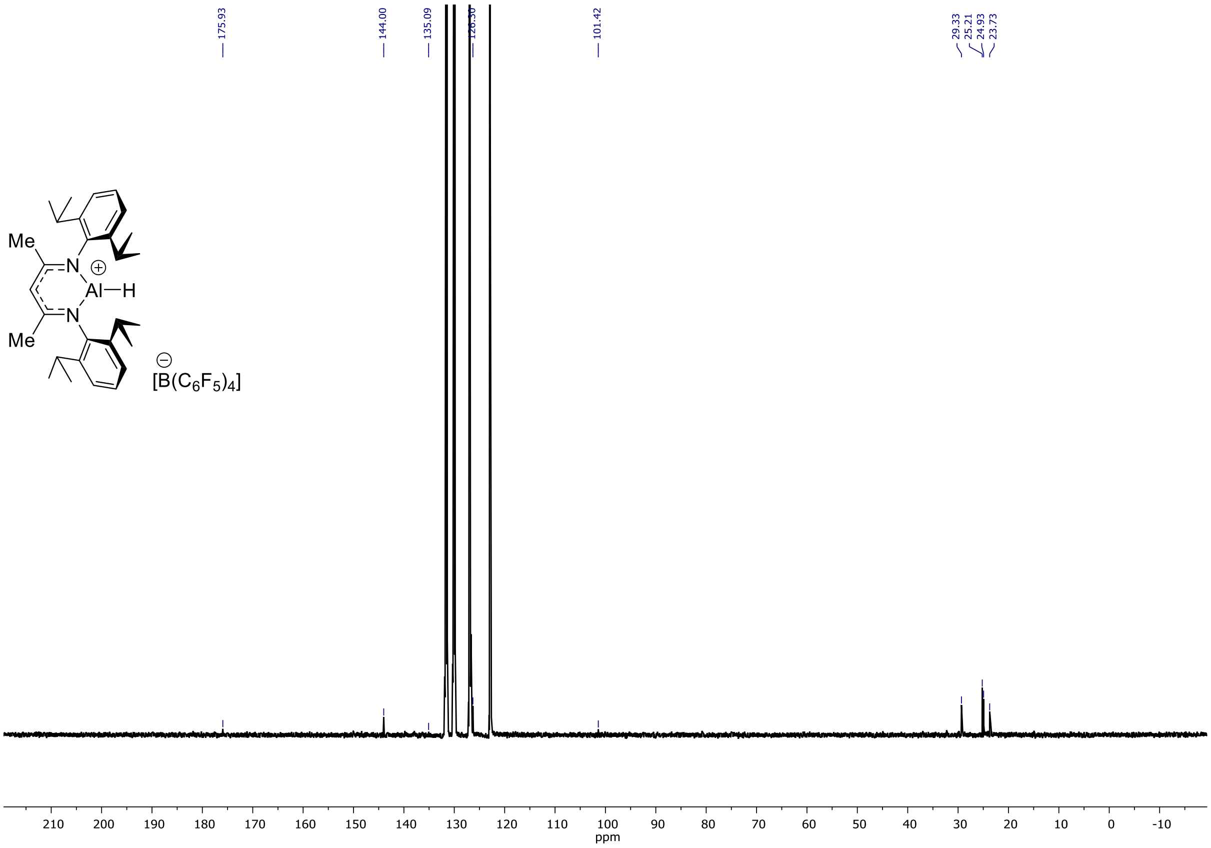


**Figure S25:** $^{13}$C NMR (151 MHz, 298 K) spectrum of [($^{Me,DIPP}$BDI)AlH$^+$][B($C_6F_5$)$_4^-$] in $C_6D_5Br$ (poor solubility).

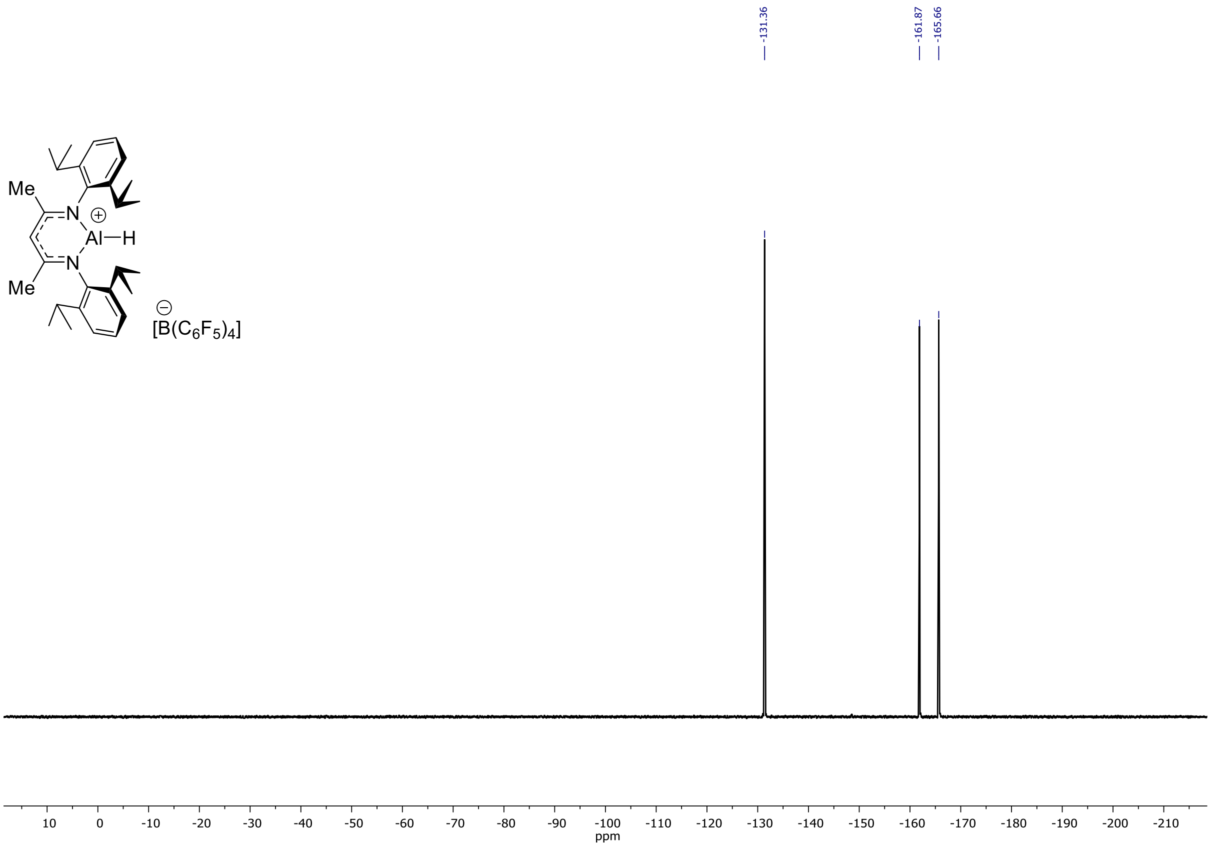


**Figure S26:** $^{19}$F NMR (565 MHz, 298 K) spectrum of [($^{Me,DIPP}$BDI)AlH$^+$][B(C$_6$F$_5$)$_4$$^-$] in C$_6$D$_5$Br.

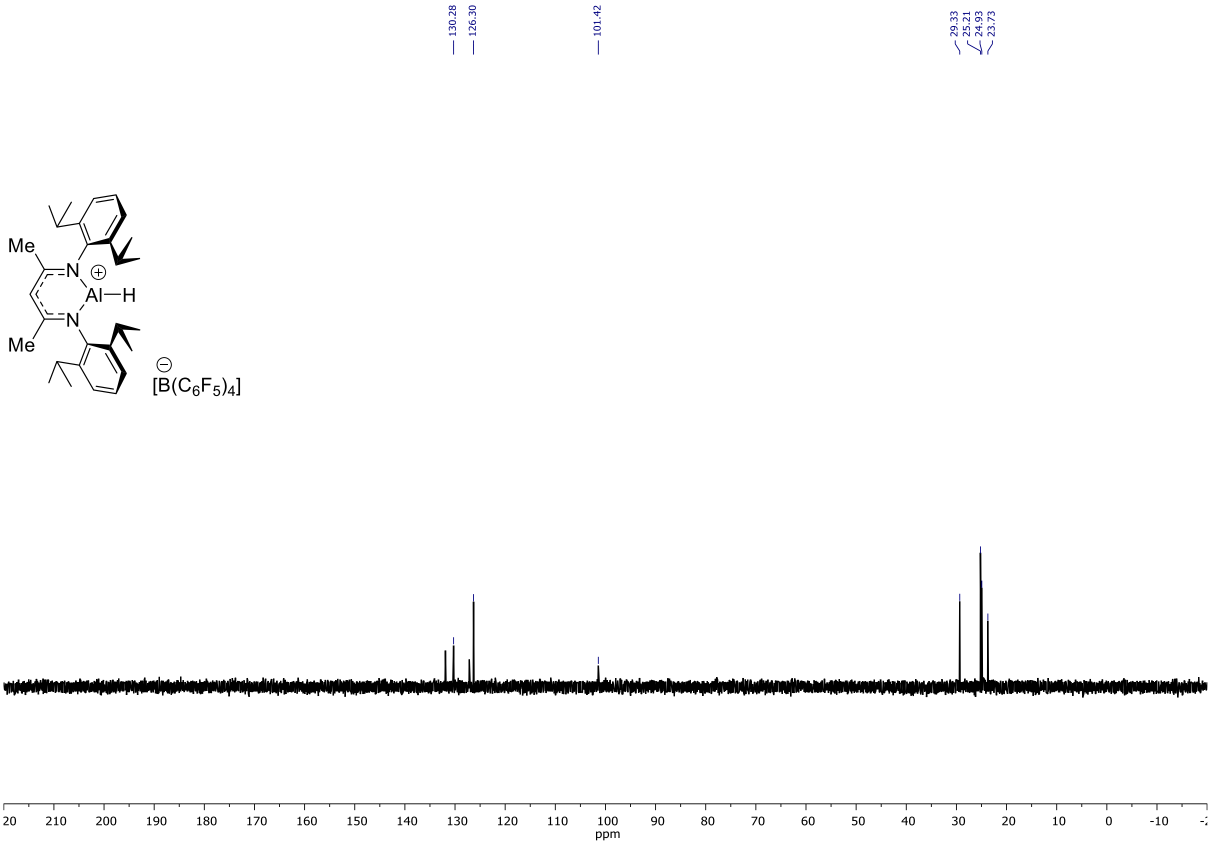


**Figure S27:** $^{13}$C DEPT135 NMR (298 K) spectrum of [($^{Me,DIPP}$BDI)AlH$^+$][B(C$_6$F$_5$)$_4$$^-$] in C$_6$D$_5$Br.

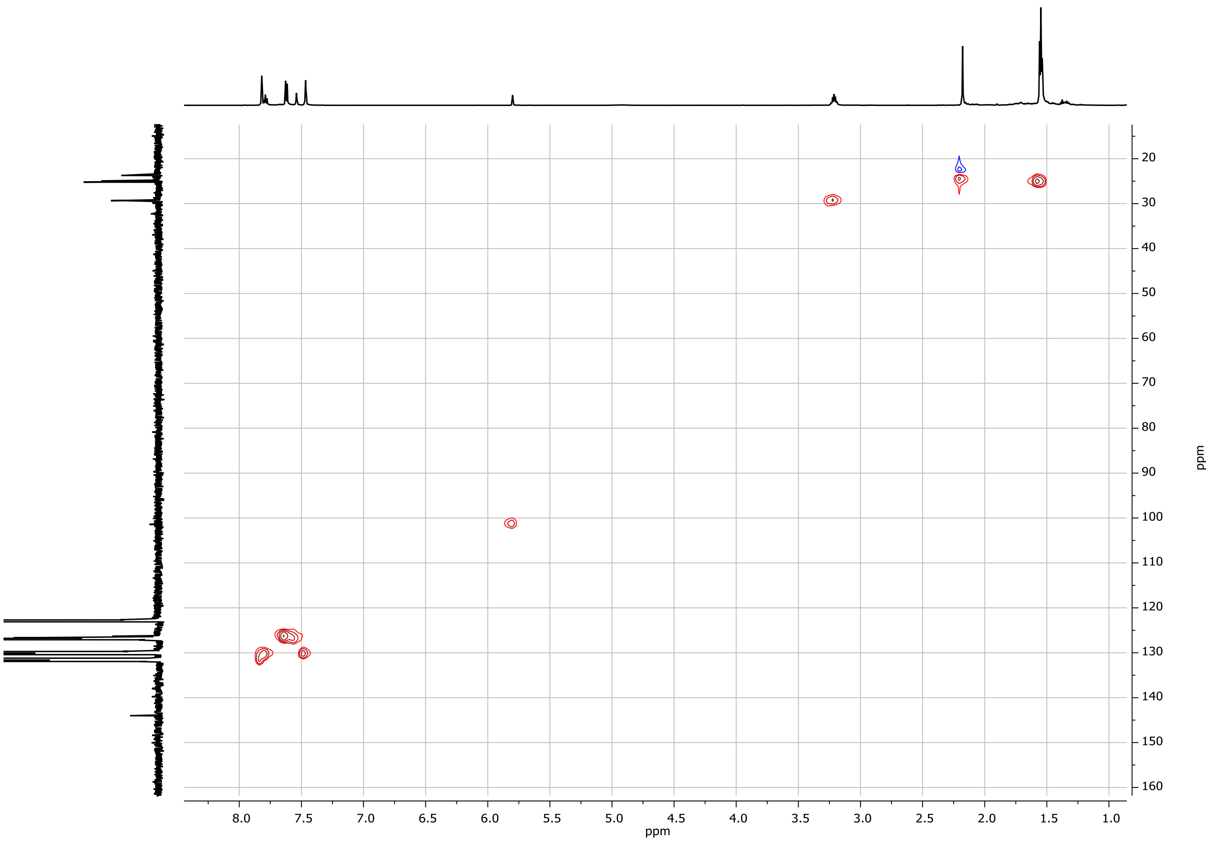


**Figure S28:** 2D HSQC NMR (298 K) spectrum of [($^{Me,DIPP}$BDI)AlH$^{+}$][B($C_6F_5$)$_4^{-}$] in $C_6D_5Br$.

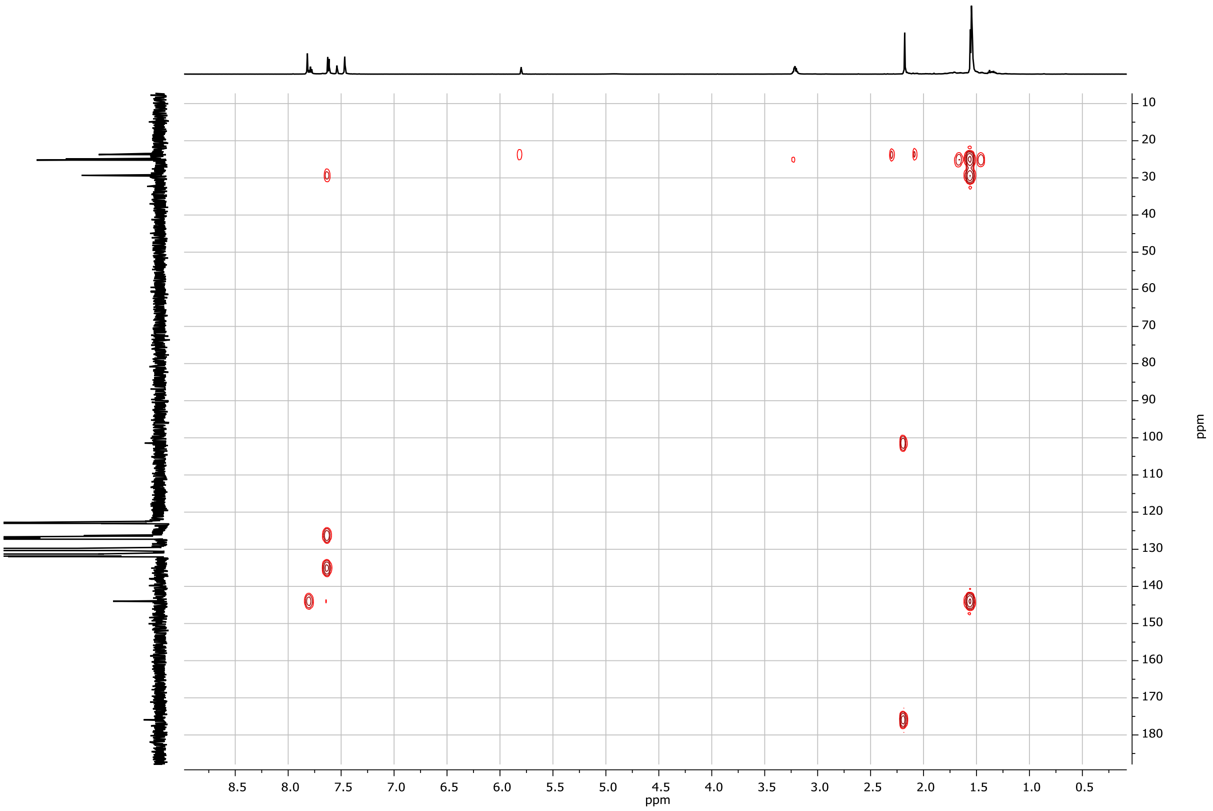


**Figure S29:** 2D HMBC NMR (298 K) spectrum of [($^{Me,DIPP}$BDI)AlH$^{+}$][B($C_6F_5$)$_4^{-}$] in $C_6D_5Br$.

## 1.2.8. Spectra of [($^{Me,DIPeP}$BDI)AlMe$^+$][B(C$_6$F$_5$)$_4$$^-$]

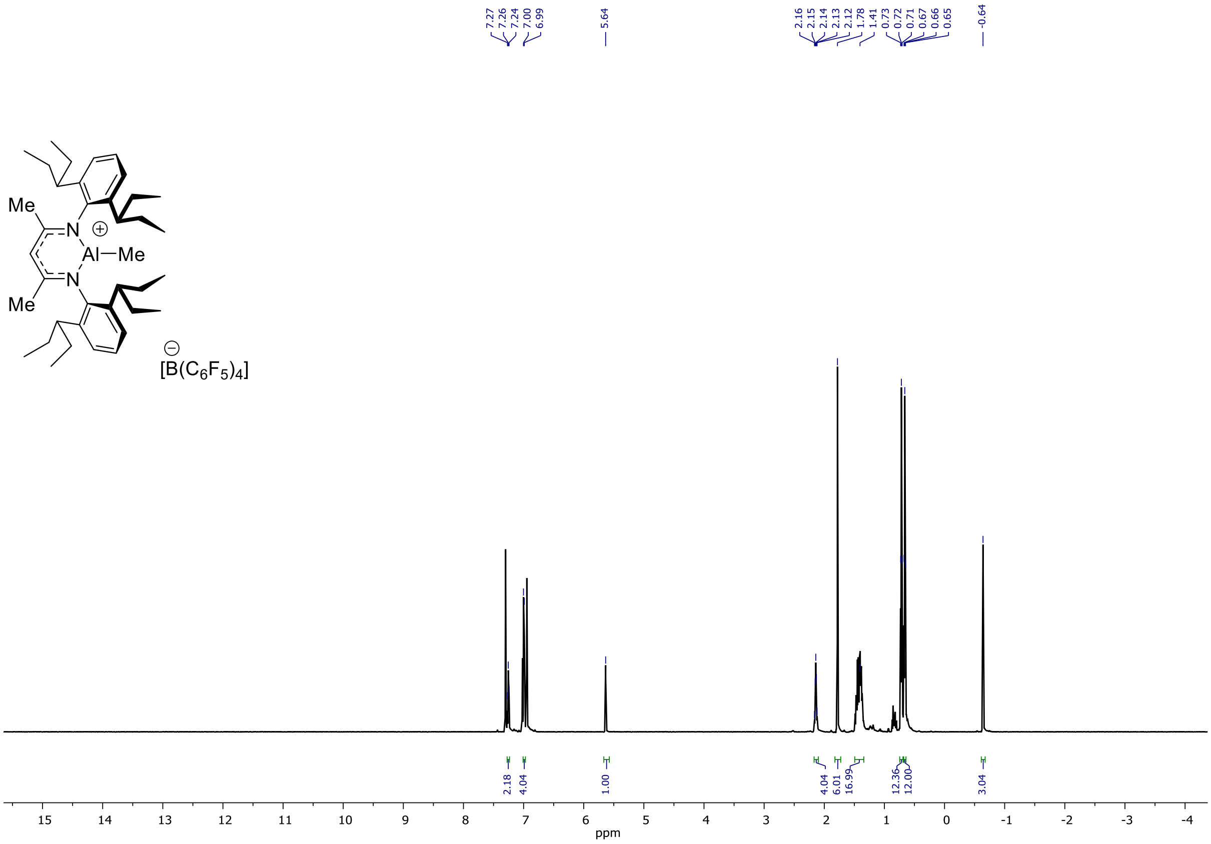


**Figure S30:** $^1$H NMR (600 MHz, 298 K) spectrum of [($^{Me,DIPeP}$BDI)AlMe$^+$][B(C$_6$F$_5$)$_4$$^-$] in C$_6$D$_5$Br.

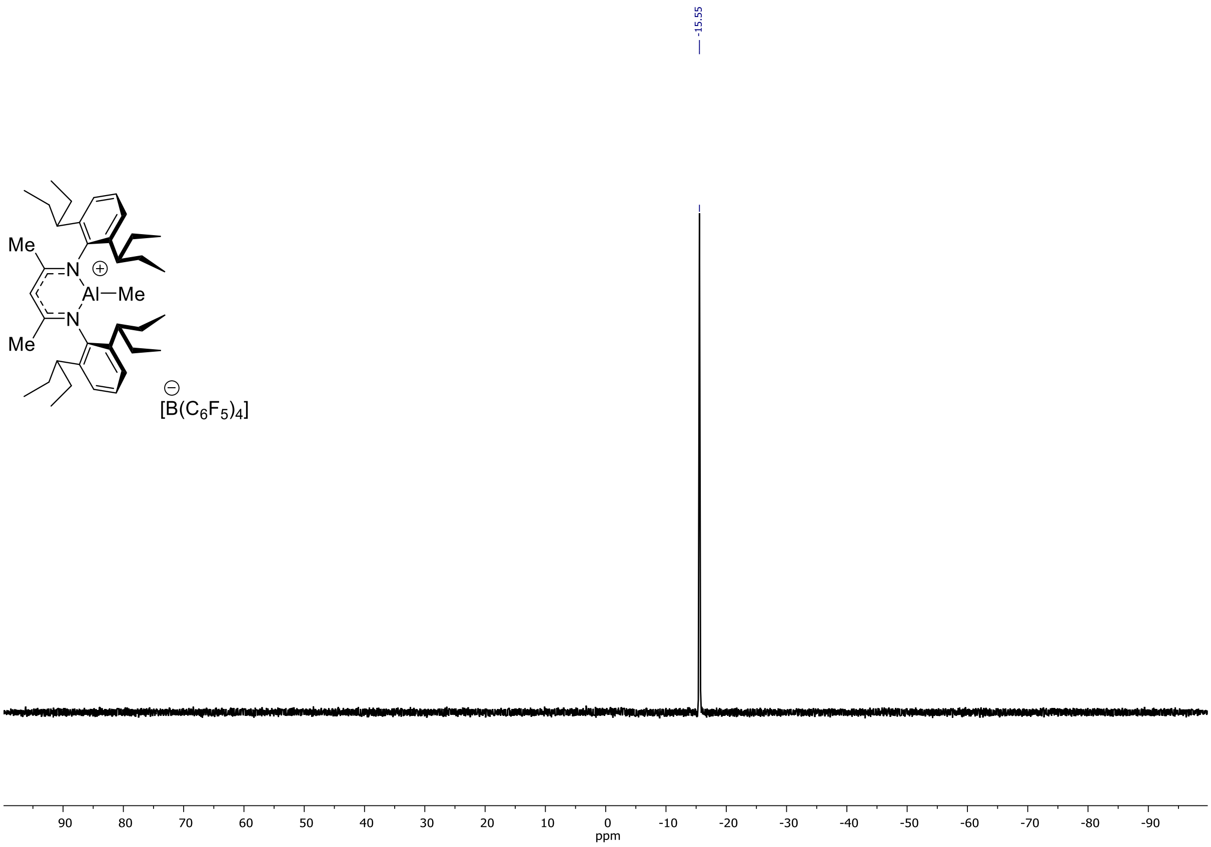


**Figure S31:** $^{11}$B NMR (192 MHz, 298 K) spectrum of [($^{Me,DIPeP}$BDI)AlMe$^+$][B(C$_6$F$_5$)$_4$$^-$] in C$_6$D$_5$Br.

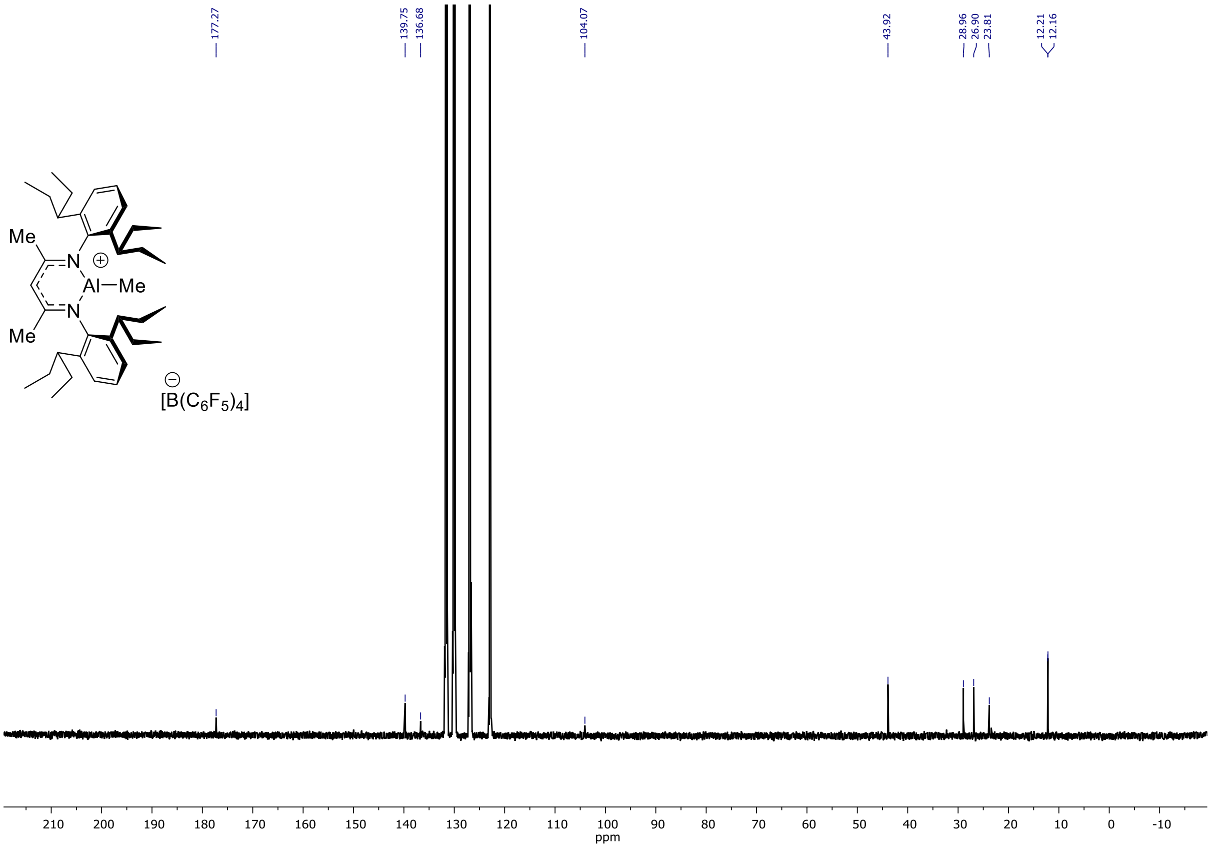


**Figure S32:** $^{13}$C NMR (151 MHz, 298 K) spectrum of [($^{Me,DIPeP}$BDI)AlMe$^+$][B(C$_6$F$_5$)$_4$$^-$] in C$_6$D$_5$Br.

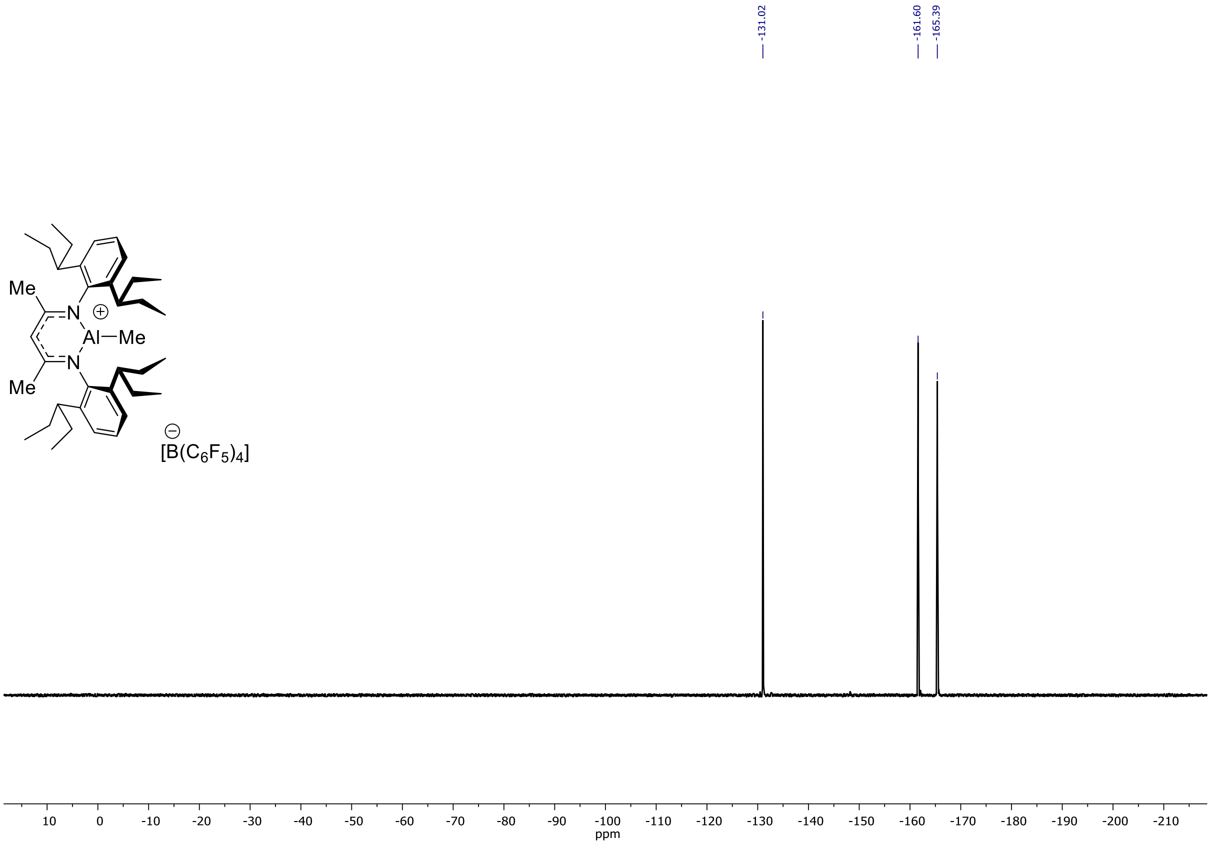


**Figure S33:** $^{19}$F NMR (565 MHz, 298 K) spectrum of [($^{Me,DIPeP}$BDI)AlMe$^+$][B(C$_6$F$_5$)$_4$$^-$] in C$_6$D$_5$Br.

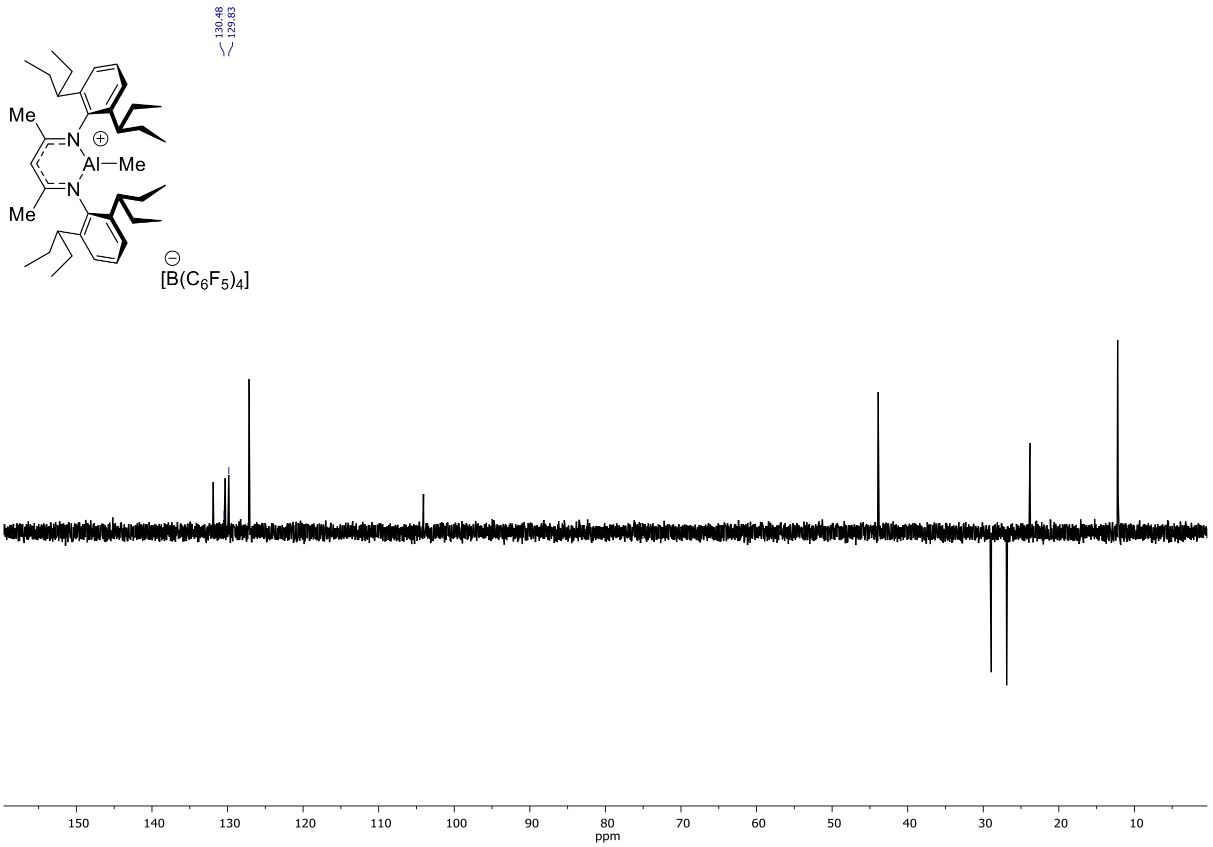


**Figure S34:** $^{13}$C DEPT135 NMR (298 K) spectrum of [($^{Me,DIPeP}$BDI)AlMe$^{+}$][B(C$_6$F$_5$)$_4$$^{-}$] in C$_6$D$_5$Br.

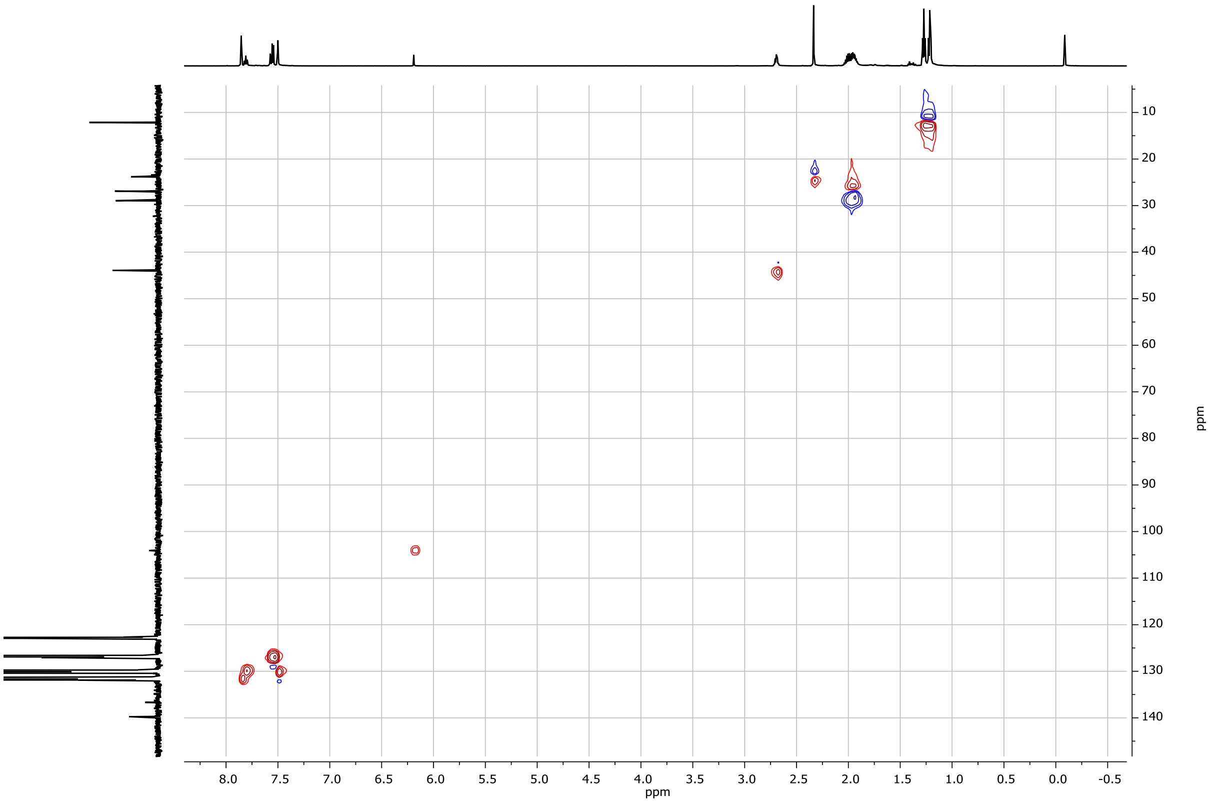


**Figure S35:** 2D HSQC NMR (298 K) spectrum of [($^{Me,DIPeP}$BDI)AlMe$^{+}$][B(C$_6$F$_5$)$_4$$^{-}$] in C$_6$D$_5$Br.

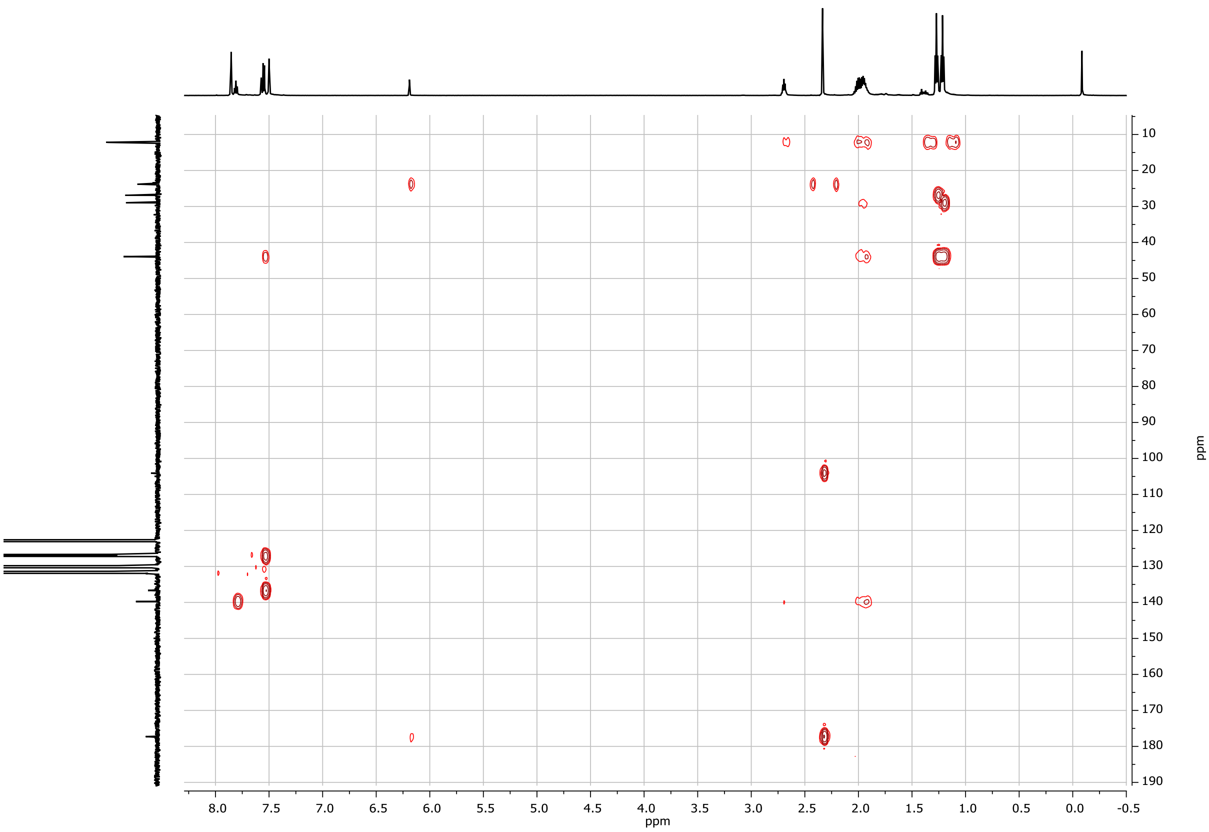


**Figure S36:** 2D HMBC NMR (298 K) spectrum of [($^{Me,DIPeP}$BDI)AlMe$^{+}$][B($C_6F_5$)$_4^{-}$] in $C_6D_5Br$.

### 1.2.9. Spectra of [($^{tBu,DIPeP}$BDI)AlMe$^{+}$][B($C_6F_5$)$_4^{-}$]

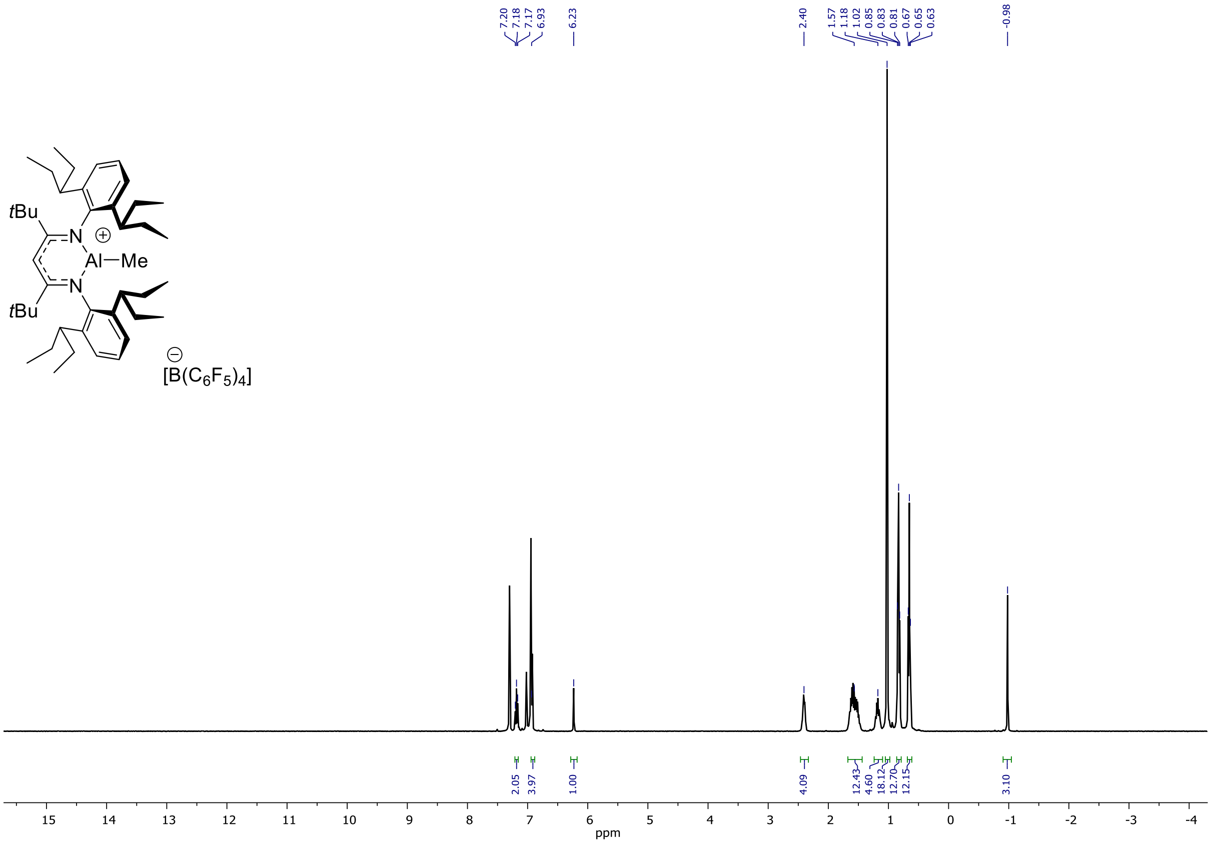


**Figure S37:** $^{1}$H NMR (400 MHz, 298 K) spectrum of [($^{tBu,DIPeP}$BDI)AlMe$^{+}$][B($C_6F_5$)$_4^{-}$] in $C_6D_5Br$.

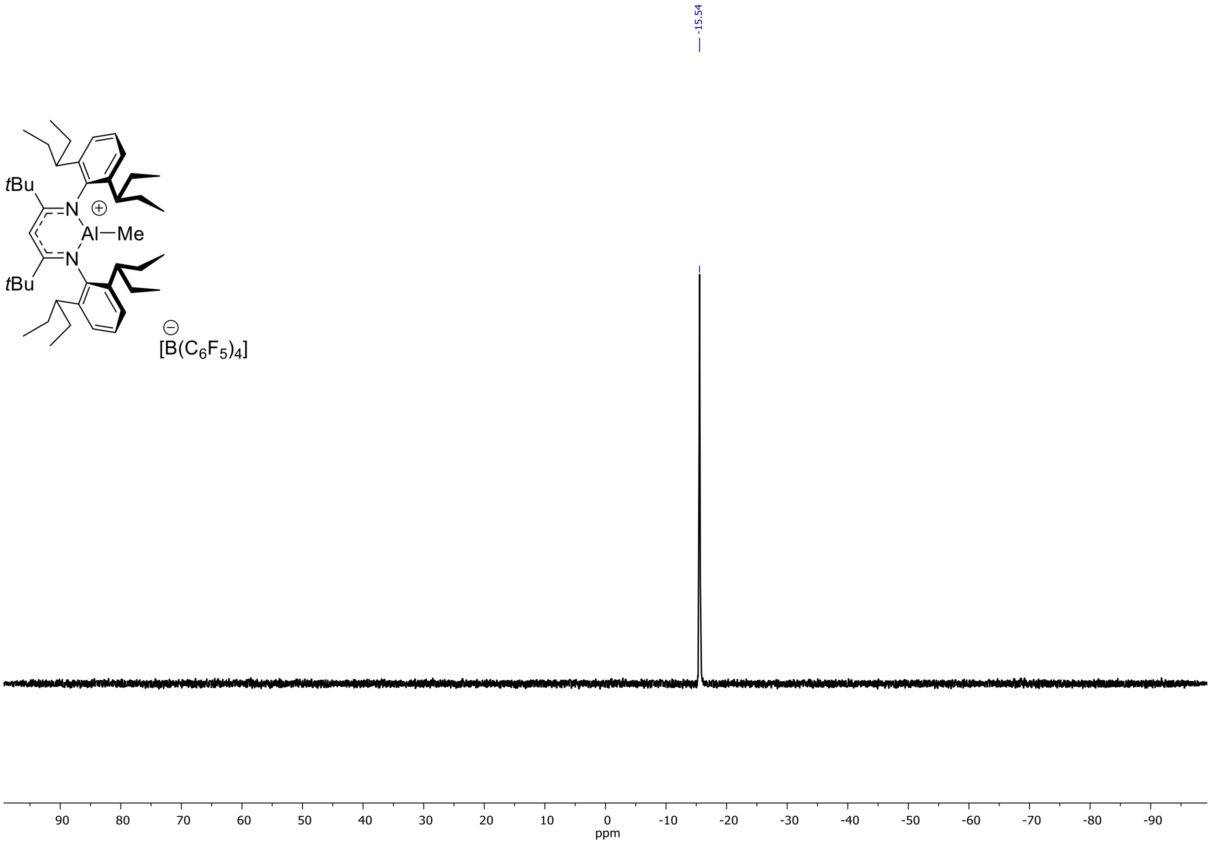


**Figure S38:** $^{11}B$ NMR (128 MHz, 298 K) spectrum of $[(^{tBu,DIPeP}BDI)AlMe^{+}][B(C_6F_5)_4^{-}]$ in $C_6D_5Br$.

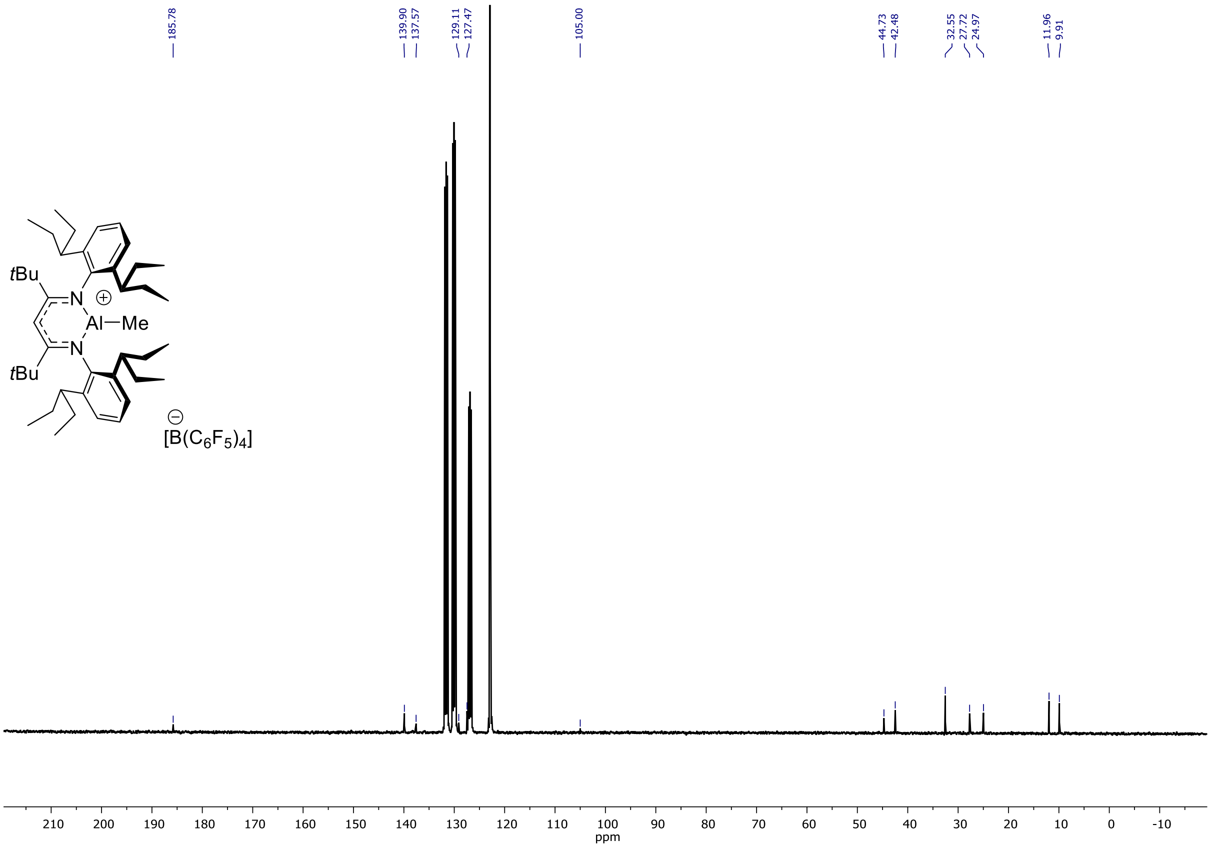


**Figure S39:** $^{13}C$ NMR (101 MHz, 298 K) spectrum of $[(^{tBu,DIPeP}BDI)AlMe^{+}][B(C_6F_5)_4^{-}]$ in $C_6D_5Br$.

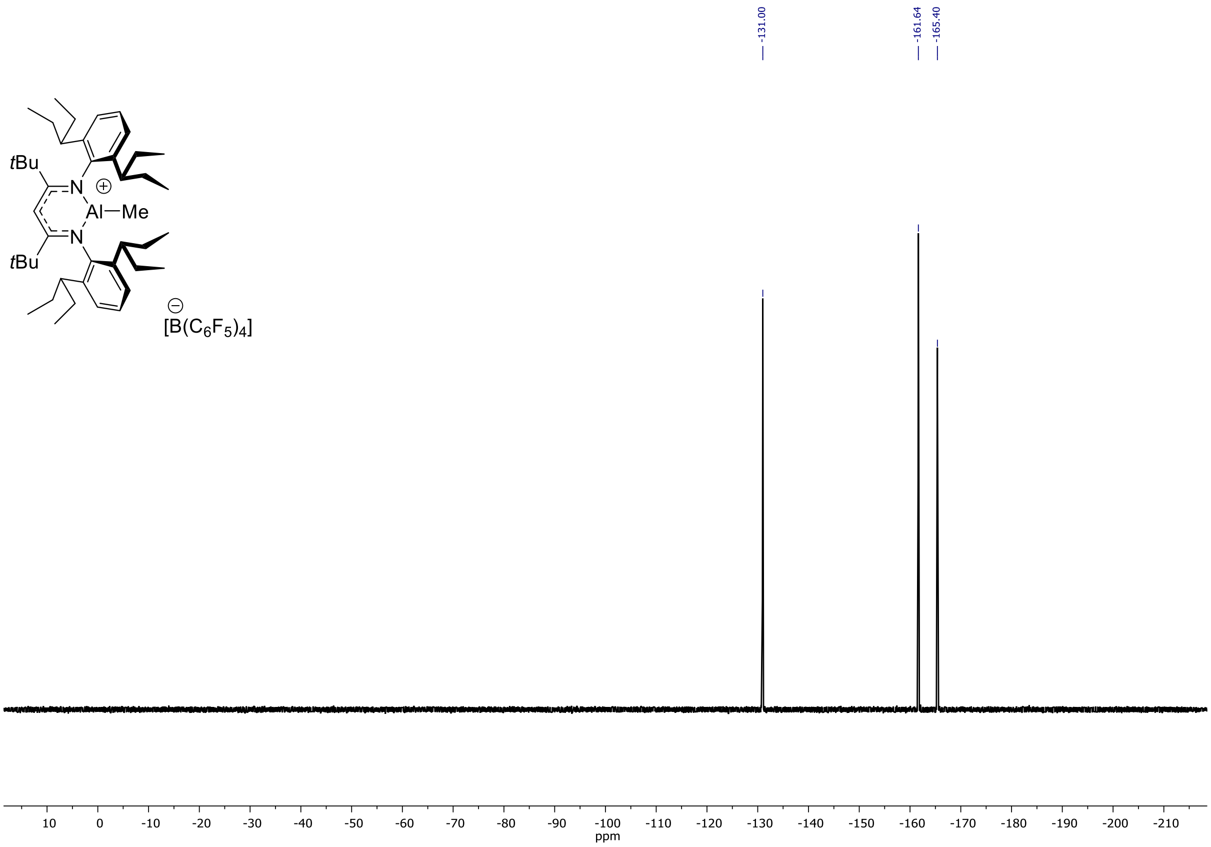


**Figure S40:** $^{19}$F NMR (376 MHz, 298 K) spectrum of $[(^{tBu,DIPeP}BDI)AlMe^{+}][B(C_6F_5)_4^{-}]$ in $C_6D_5Br$.

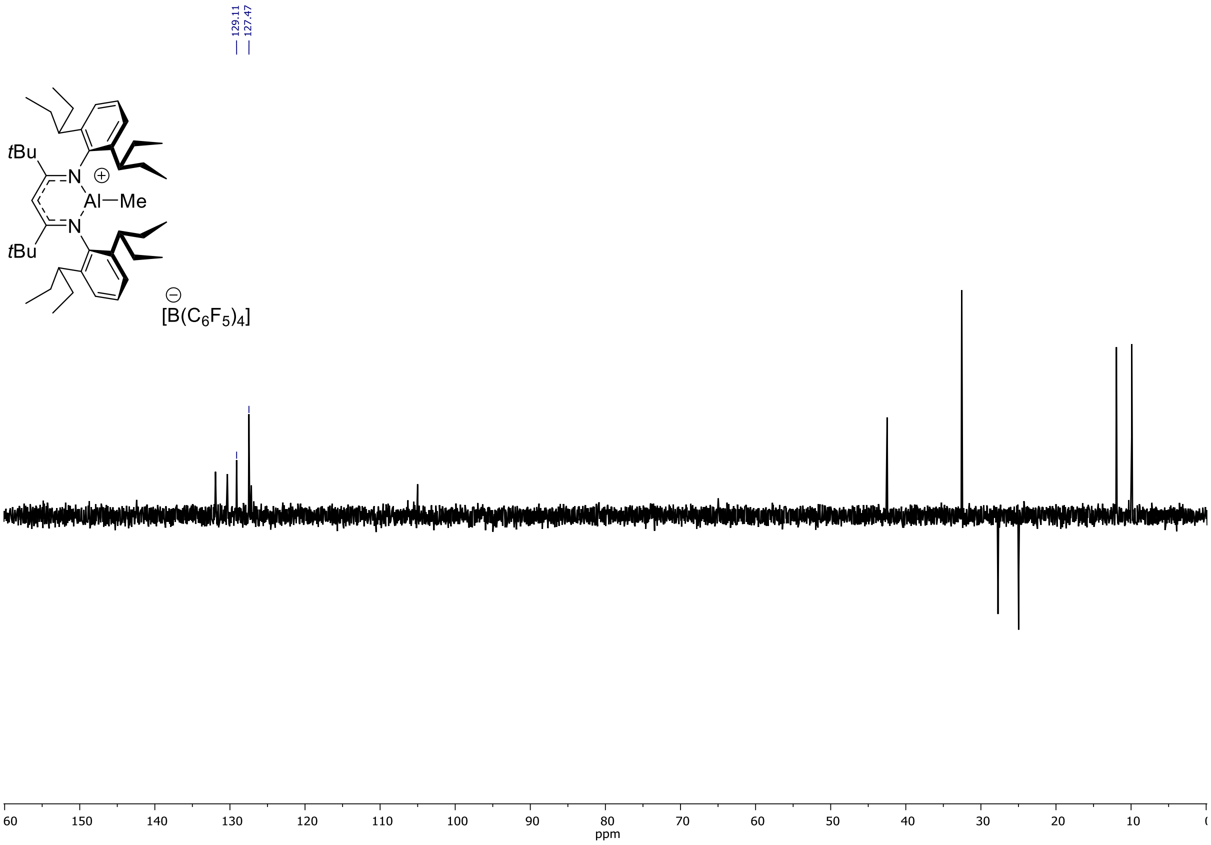


**Figure S41:** $^{13}$C DEPT135 NMR (298 K) spectrum of $[(^{tBu,DIPeP}BDI)AlMe^{+}][B(C_6F_5)_4^{-}]$ in $C_6D_5Br$.

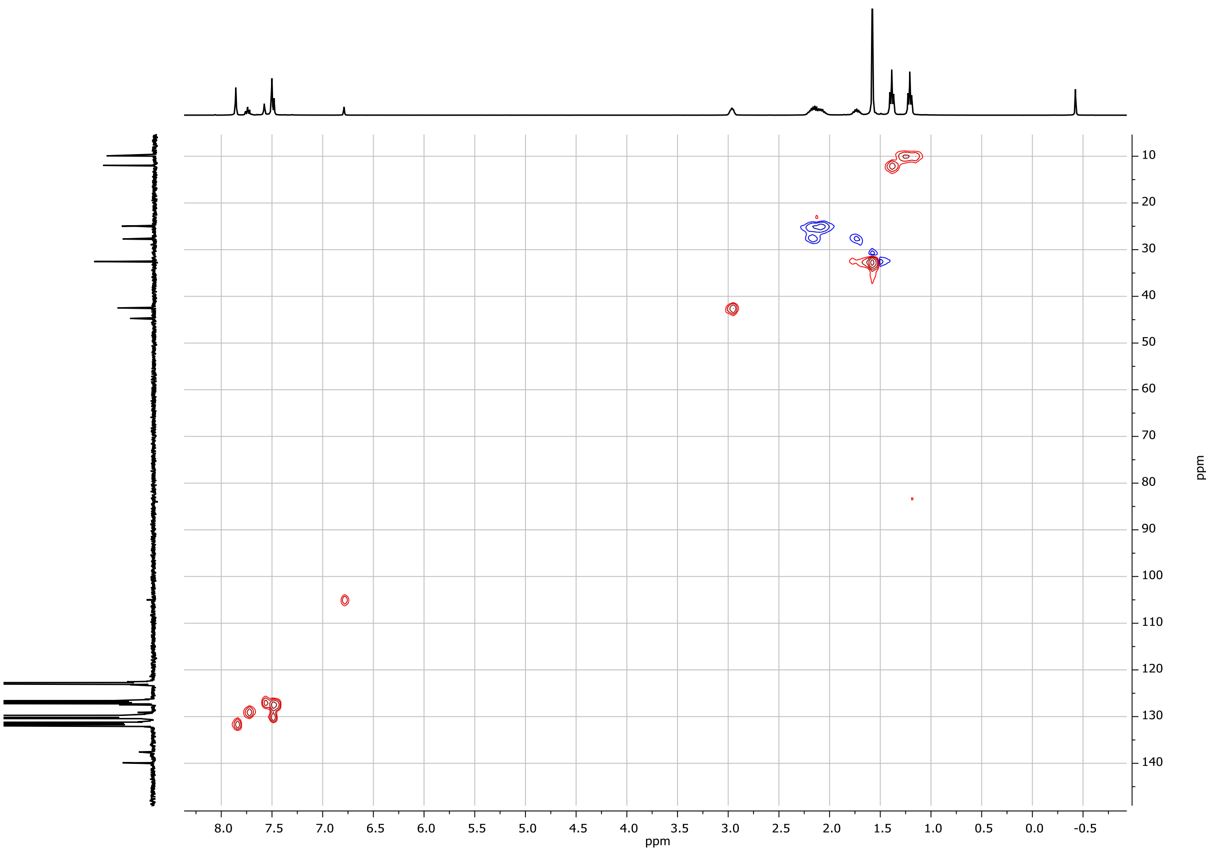

**Figure S42:** 2D HSQC NMR (298 K) spectrum of [($^{tBu,DIPeP}$BDI)AlMe$^+$][B($C_6F_5$)$_4$$^-$] in $C_6D_5Br$.

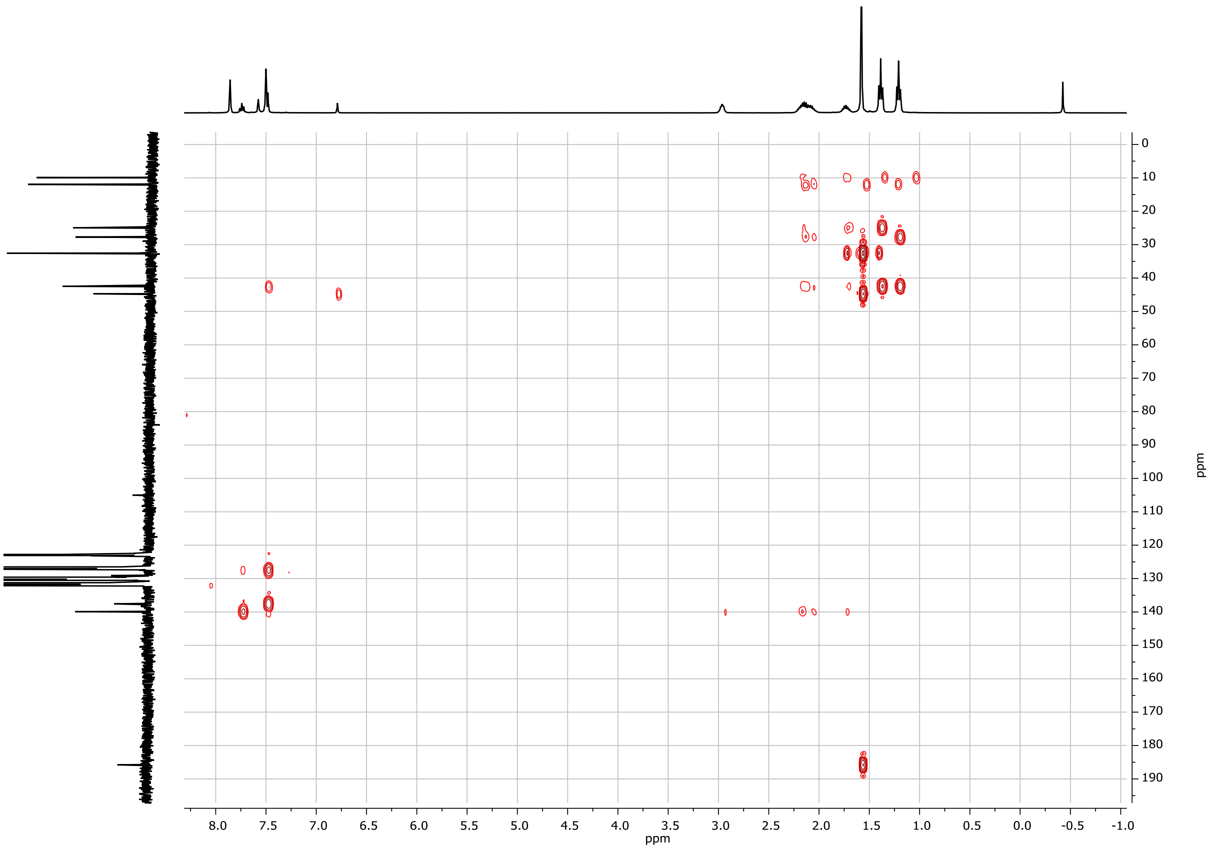

**Figure S43:** 2D HMBC NMR (298 K) spectrum of [($^{tBu,DIPeP}$BDI)AlMe$^+$][B($C_6F_5$)$_4$$^-$] in $C_6D_5Br$.

## 1.3 Lewis acidity measurement (Gutmann Beckett Method)

### 1.3.1. Gutmann Beckett method for [($^{tBu,DIPP}$BDI)AlMe$^+$][B($C_6F_5$)$_4^-$]

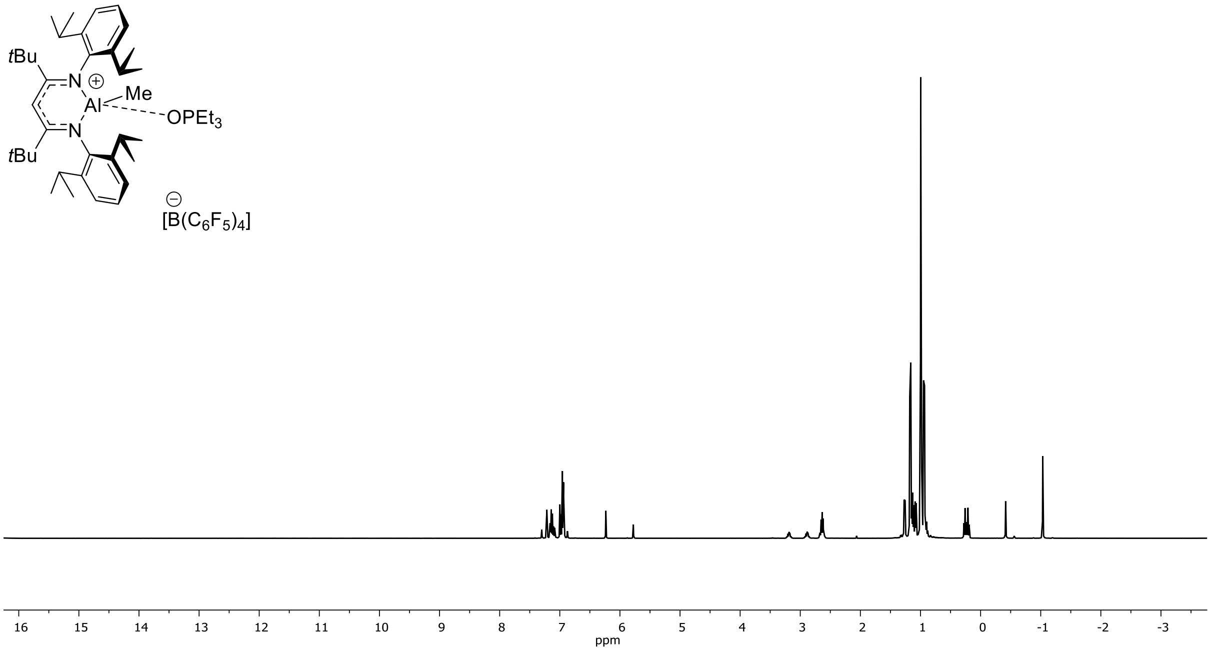


**Figure S44:** $^1$H NMR (400 MHz, 298 K) spectrum of Gutmann Beckett test in $C_6D_5Br$.

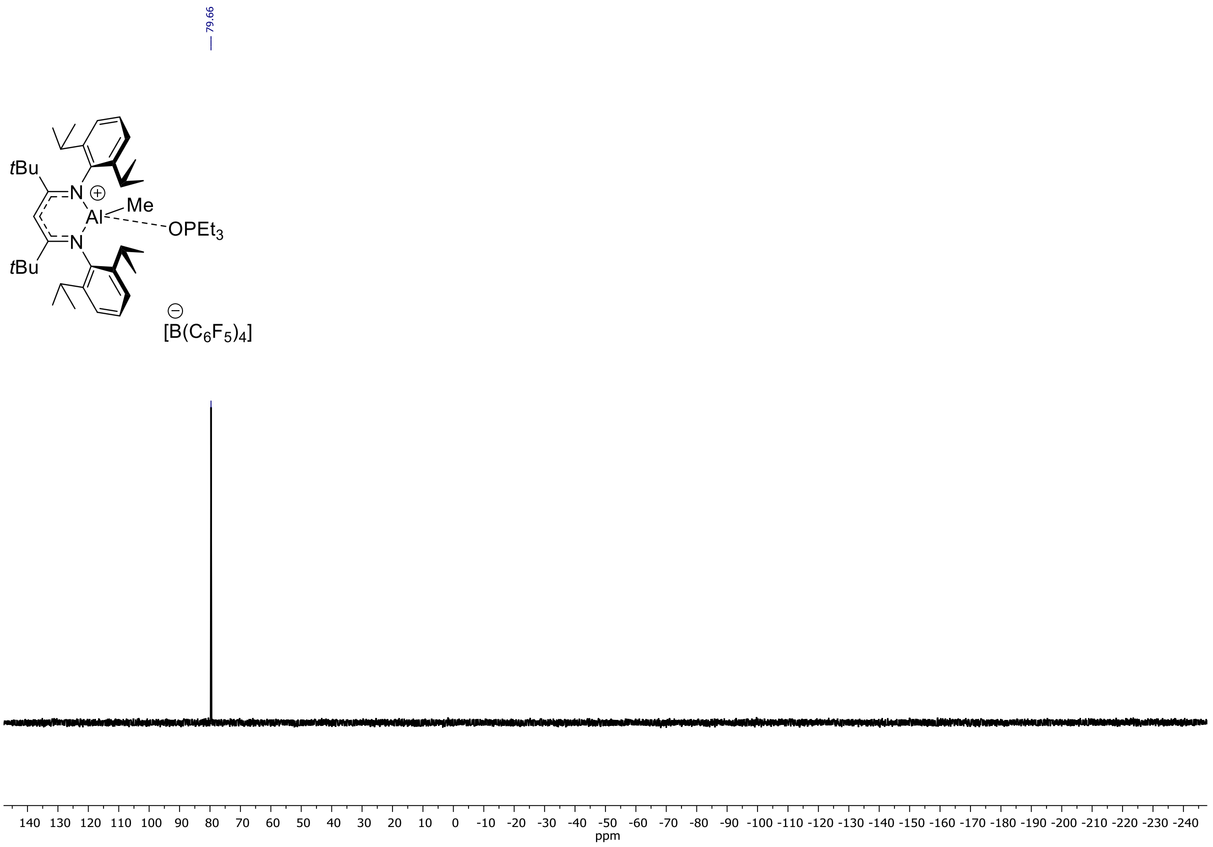


**Figure S45:** $^{31}$P NMR (162 MHz, 298 K) spectrum of Gutmann Beckett test in $C_6D_5Br$.

### 1.3.2. Gutmann Beckett method for [($^{Me,DIPP}$BDI)AlH$^+$][B($C_6F_5$)$_4^-$]

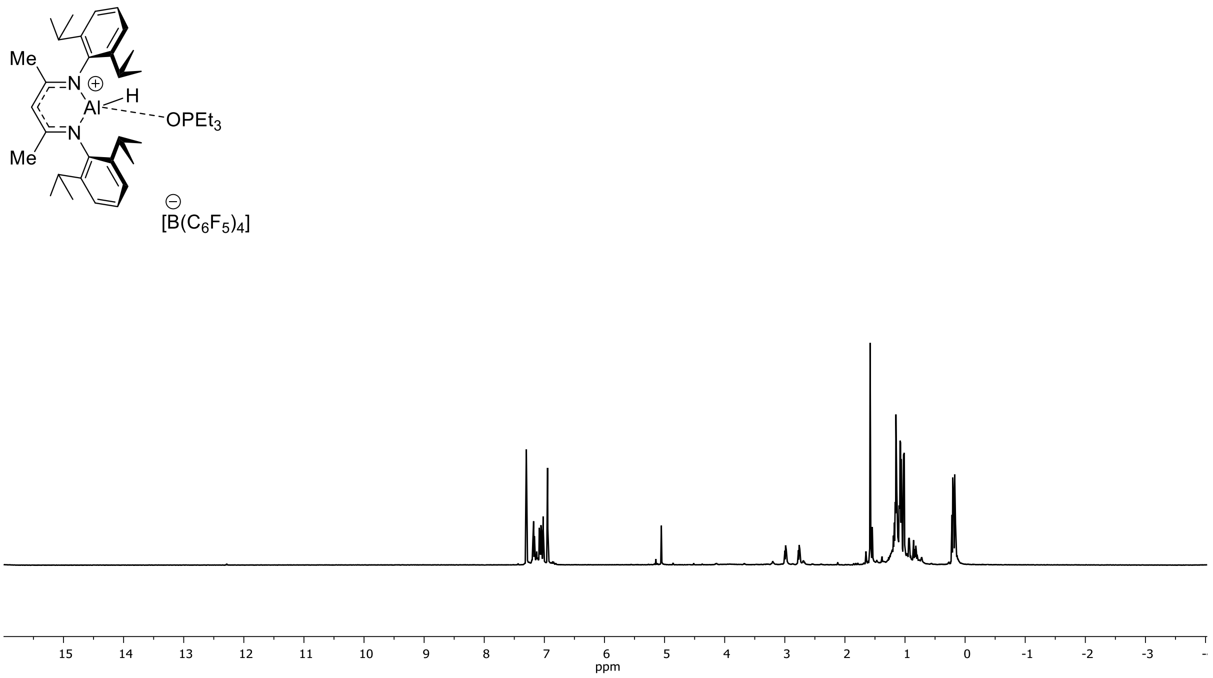


**Figure S46:** $^{1}$H NMR (600 MHz, 298 K) spectrum of Gutmann Beckett test in $C_6D_5Br$.

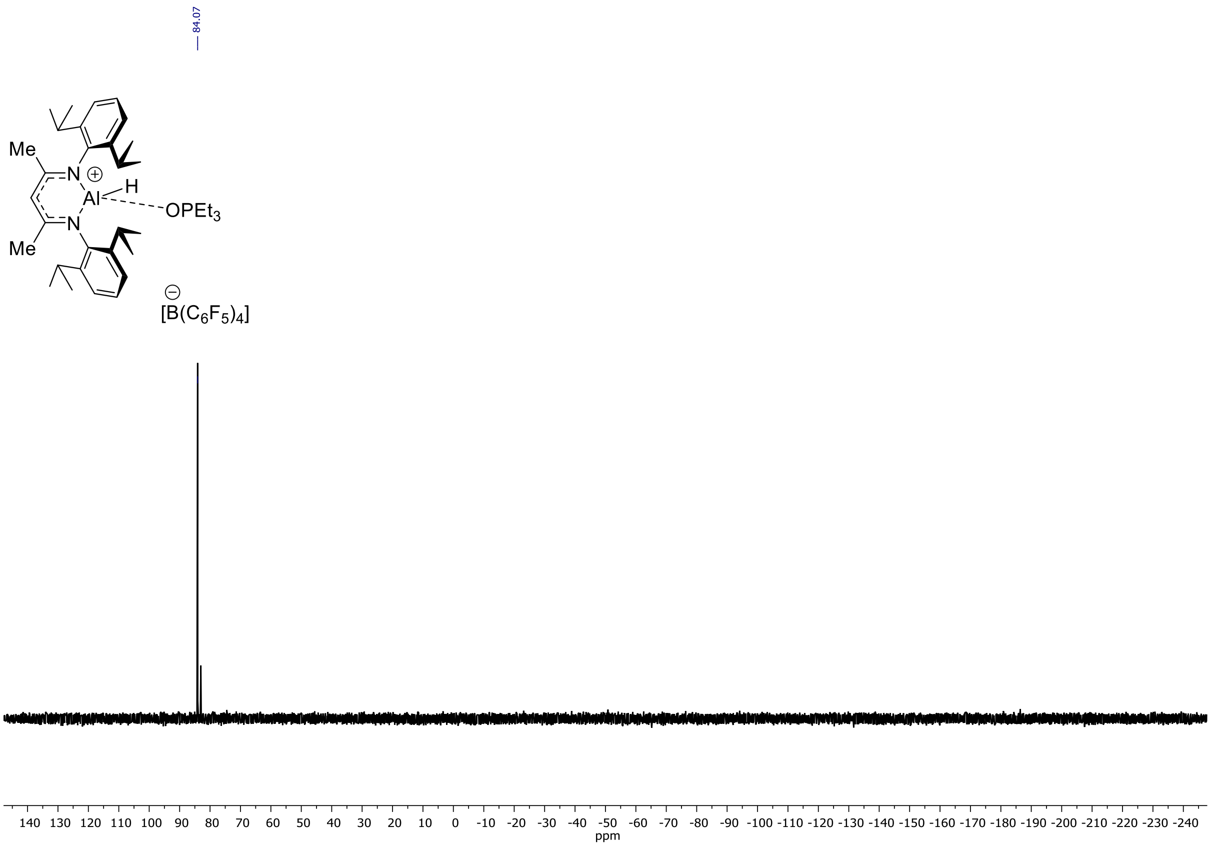


**Figure S47:** $^{31}$P NMR (243 MHz, 298 K) spectrum of Gutmann Beckett test in $C_6D_5Br$

### 1.3.3. Gutmann Beckett method for [($^{Me,DIPeP}$BDI)AlMe$^+$][B($C_6F_5$)$_4^-$]

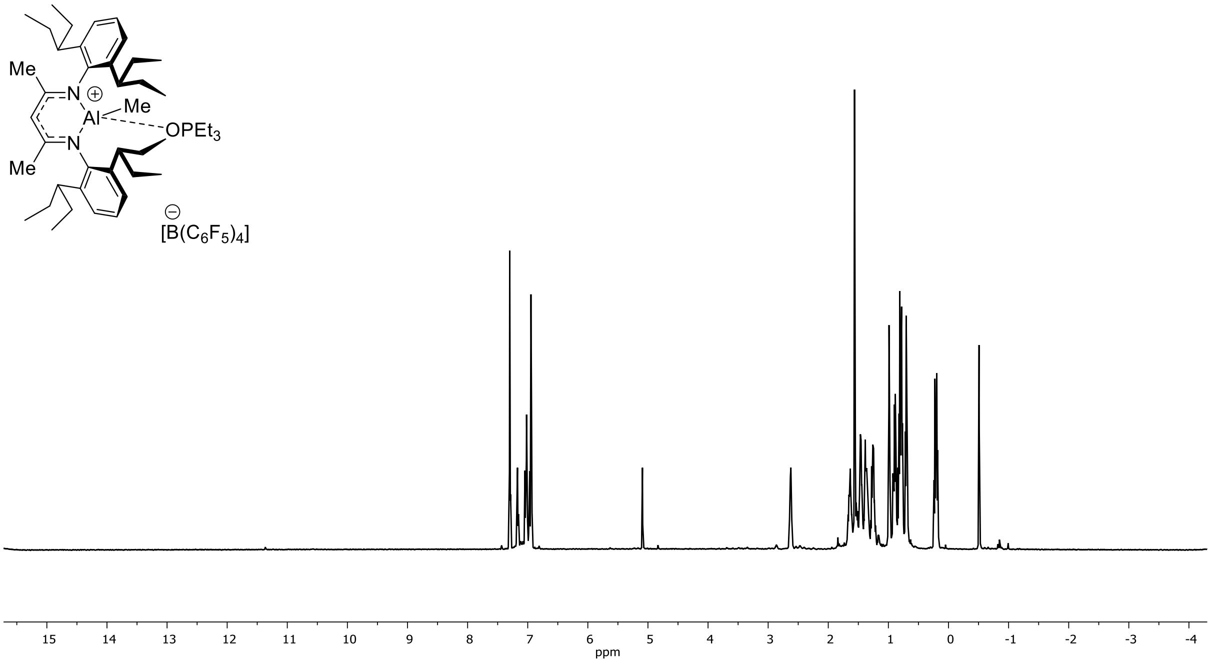


**Figure S48:** $^1$H NMR (600 MHz, 298 K) spectrum of Gutmann Beckett test in $C_6D_5Br$.

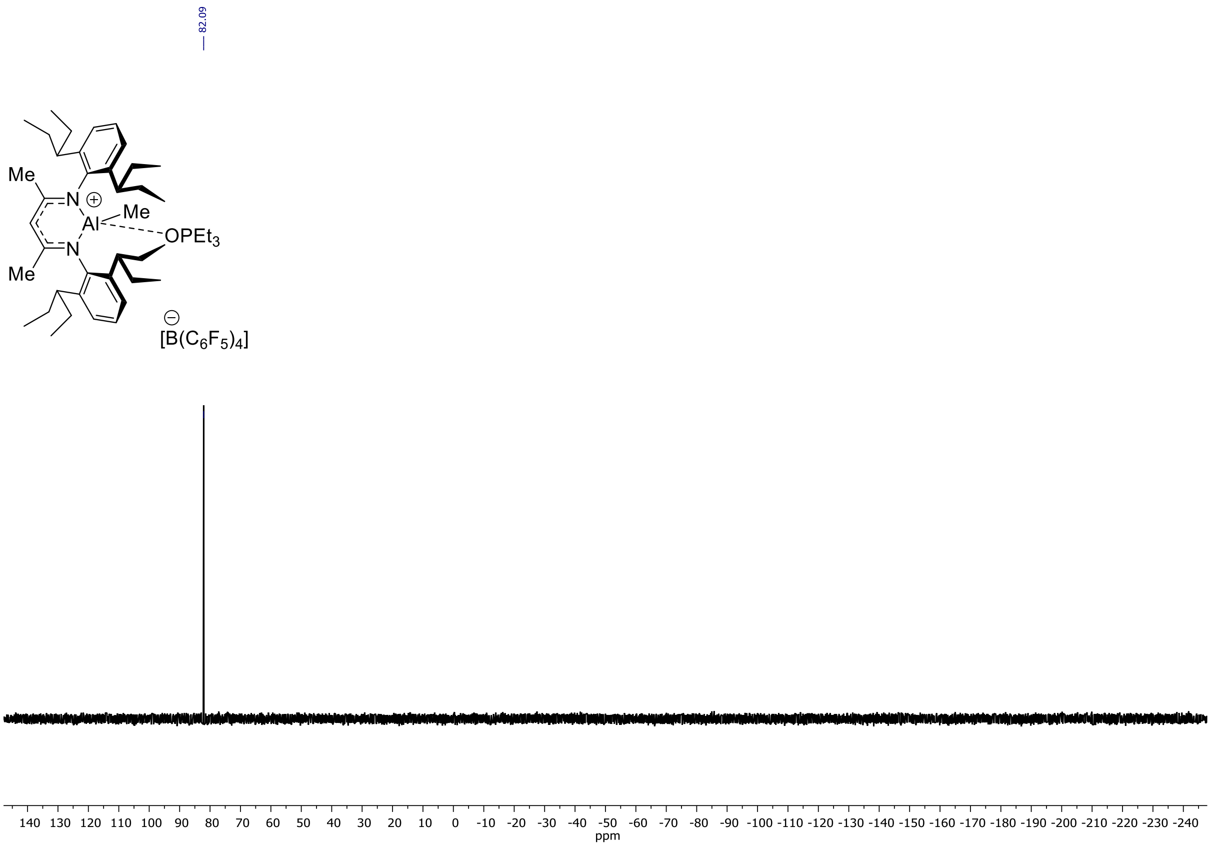


**Figure S49:** $^{31}P$ NMR (243 MHz, 298 K) spectrum of Gutmann Beckett test in $C_6D_5Br$

### 1.3.4. Gutmann Beckett method for $[(^{tBu,DIPeP}BDI)AlMe^+][B(C_6F_5)_4^-]$

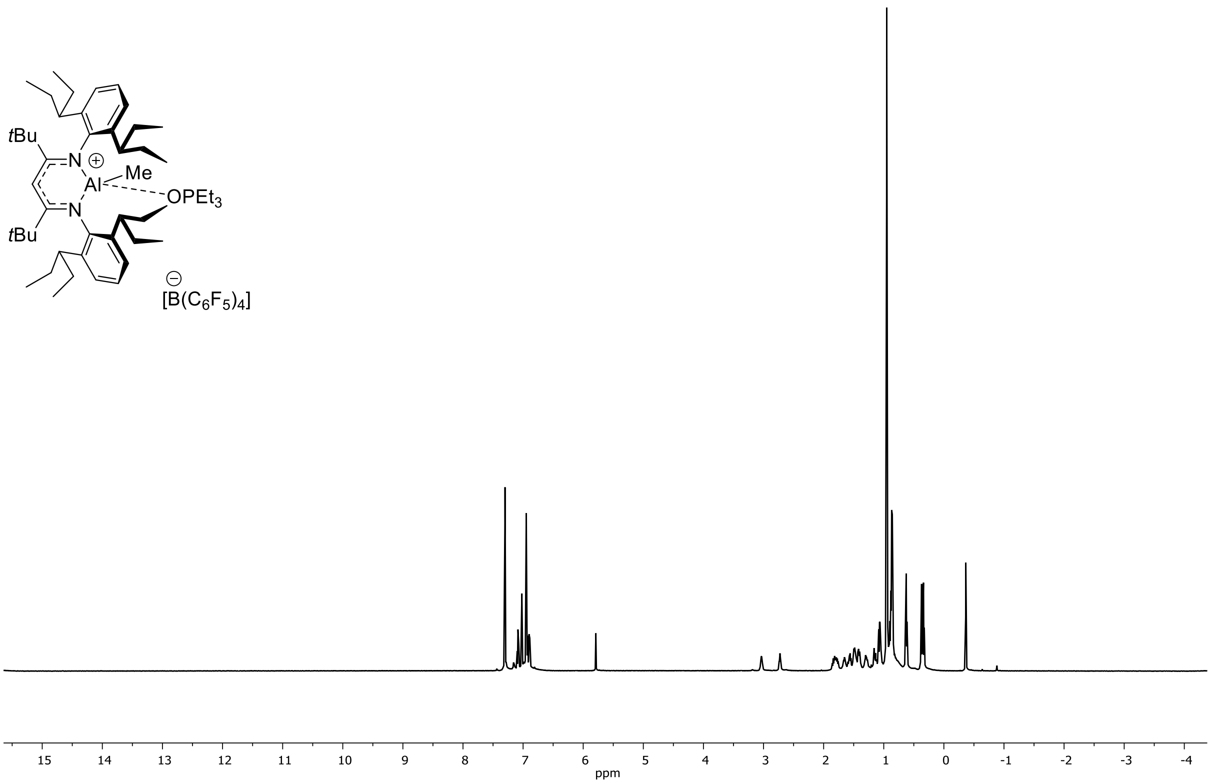


**Figure S50:** $^{1}H$ NMR (600 MHz, 298 K) spectrum of Gutmann Beckett test in $C_6D_5Br$.

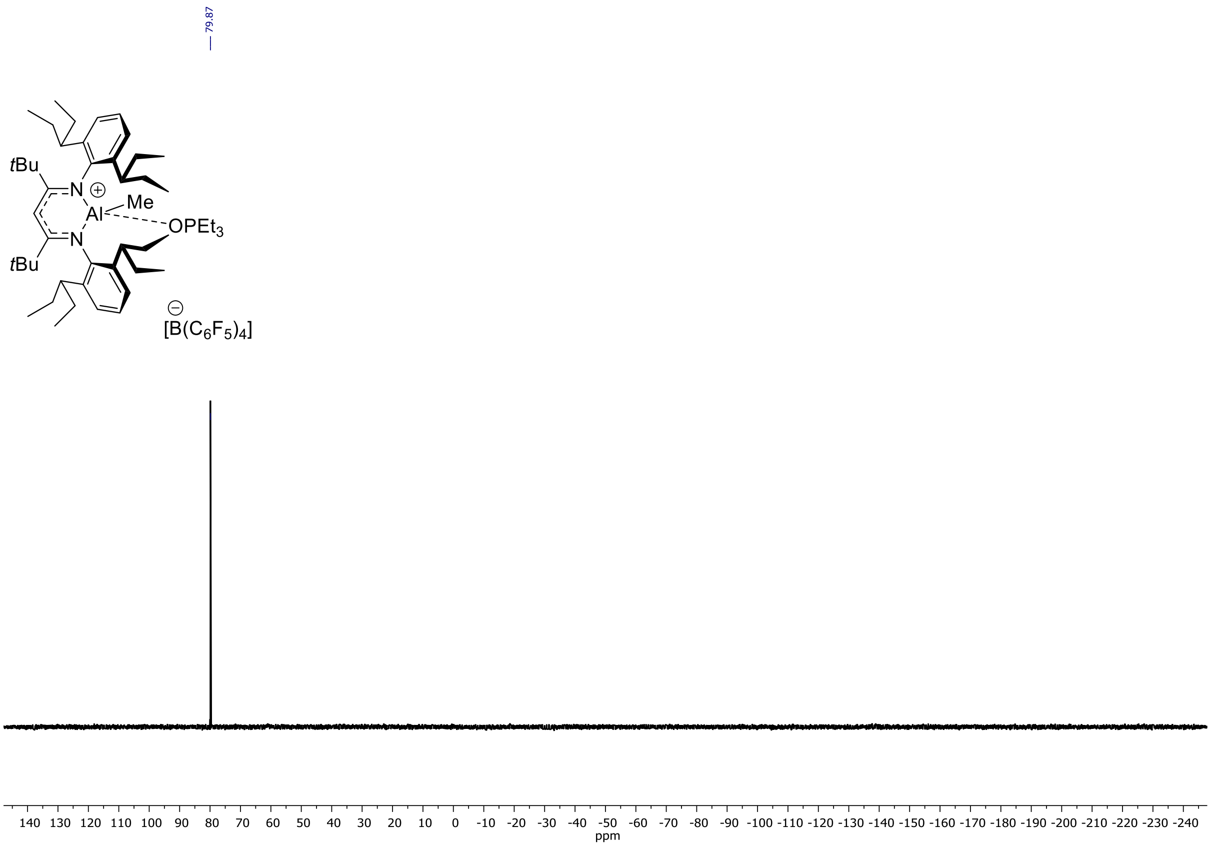


**Figure S51:** $^{31}P$ NMR (162 MHz, 298 K) spectrum of Gutmann Beckett test in $C_6D_5Br$

## 1.4 NMR data related to catalytic imine hydrogenation

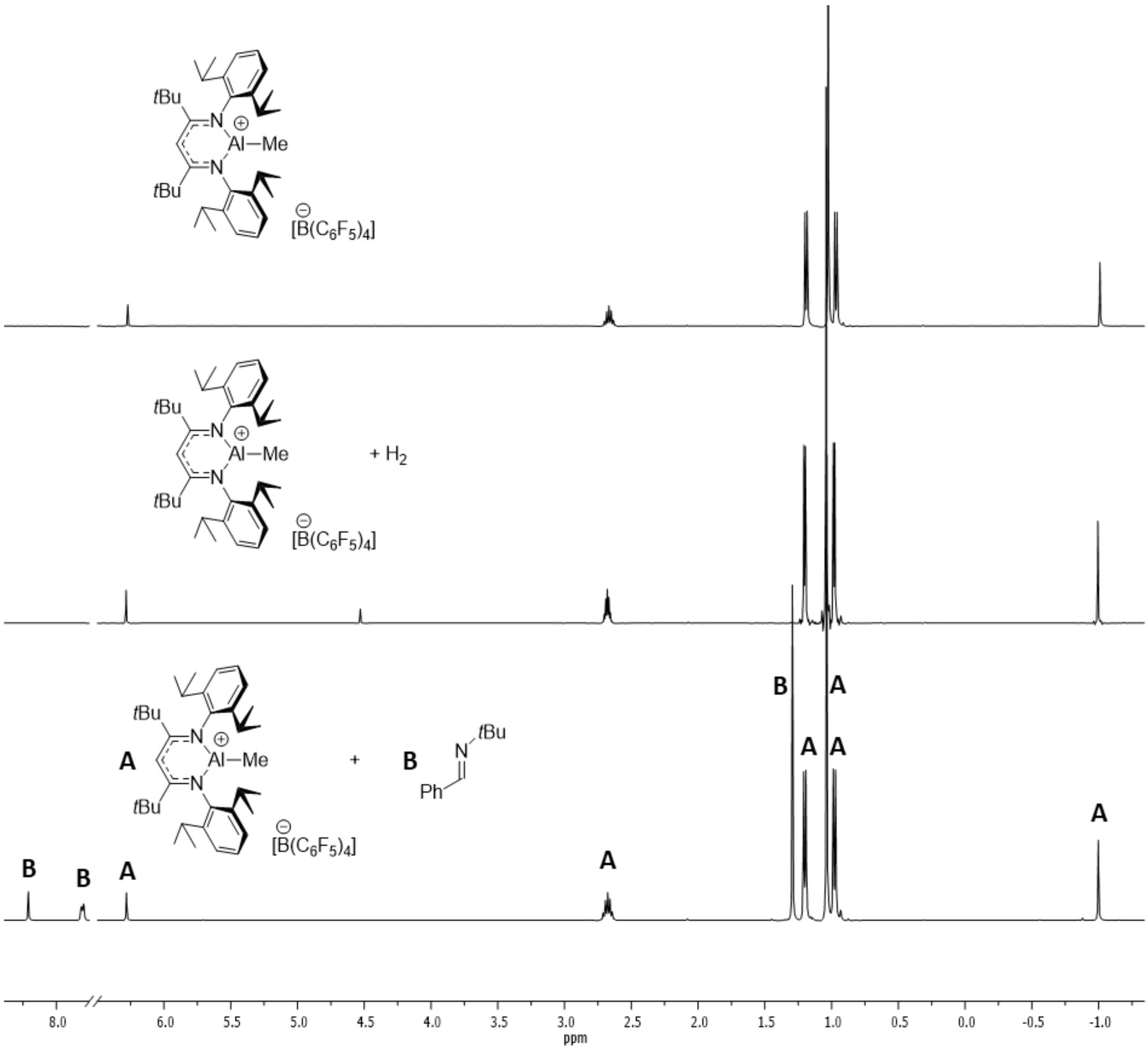


**Figure S52**: $^{1}$H NMR spectra of Top: $[(^{tBu,DIPP}BDI)AlMe^{+}][B(C_6F_5)_4^{-}]$ in $C_6D_6$/PhCl (2/1; v/v); Middle: $[(^{tBu,DIPP}BDI)AlMe^{+}][B(C_6F_5)_4^{-}]$ in $C_6D_6$/PhCl (2/1; v/v) 3x freeze thawed and pressurized with 1.5 bar $H_2$; Bottom: $[(^{tBu,DIPP}BDI)AlMe^{+}][B(C_6F_5)_4^{-}]$ in $C_6D_6$/PhCl (2/1; v/v) and one 1 eq. (Ph)CH=N(tBu). Area 7.0 to 7.5ppm (signals of PhCl) excluded for clarity.

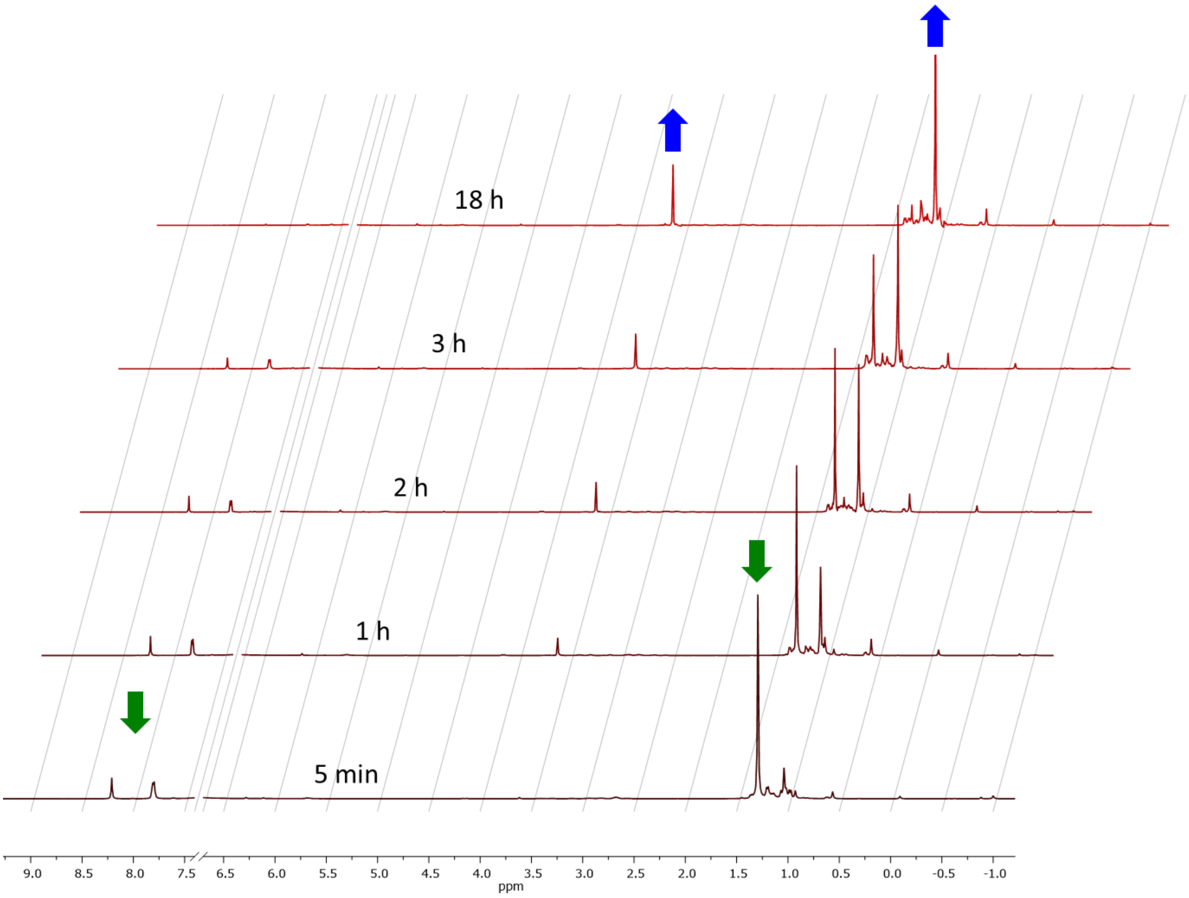


**Figure S53**: $^1$H NMR spectra of the catalytic transformation of (Ph)CH=N(tBu) (marked with green arrow) with $[(^{tBu,DIPP}BDI)AlMe^+][B(C_6F_5)_4^-]$ (10 mol%) and $H_2$ (1.5 bar) at 60 °C in $C_6D_6$/PhCl (2/1; v/v); Amine peaks marked with blue arrow. Area 7.0 to 7.5ppm (signals of PhCl) excluded for clarity.

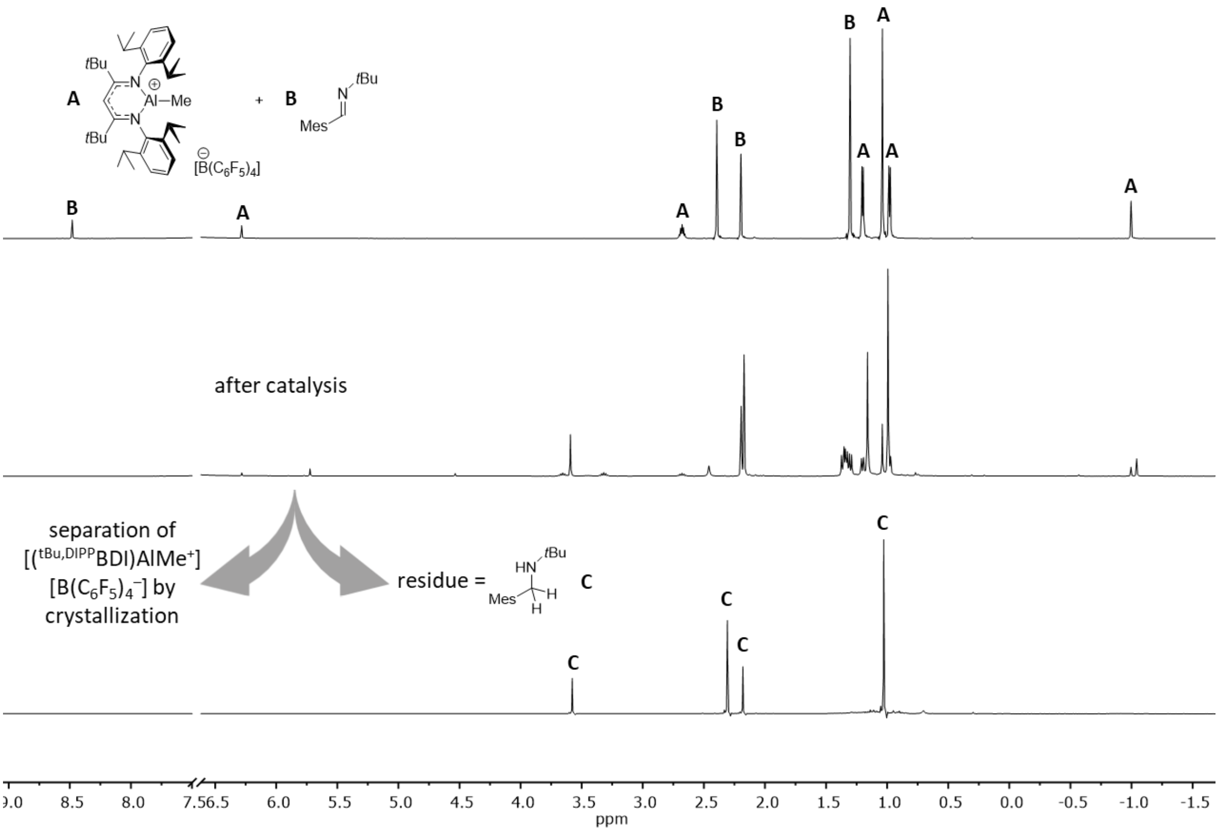


**Figure S54**: $^1$H NMR spectra of Top: $[(^{tBu,DIPP}BDI)AlMe^+][B(C_6F_5)_4^-]$ and (Mes)CH=N(tBu) 1.5 eq. in $C_6D_6$/PhCl (2/1; v/v); Middle: Reaction mixture 3x freeze thawed and pressurized with 1.5 bar $H_2$ after all imine is consumed in $C_6D_6$/PhCl (2/1; v/v); Bottom: $[(^{tBu,DIPP}BDI)AlMe^+][B(C_6F_5)_4^-]$ was separated from the reaction mixture *via* crystallization and the residue was dried *in vacuo* and re-dissolved in $C_6D_6$ to give pure amine. Area 7.0 to 7.5ppm (signals of PhCl) excluded for clarity.

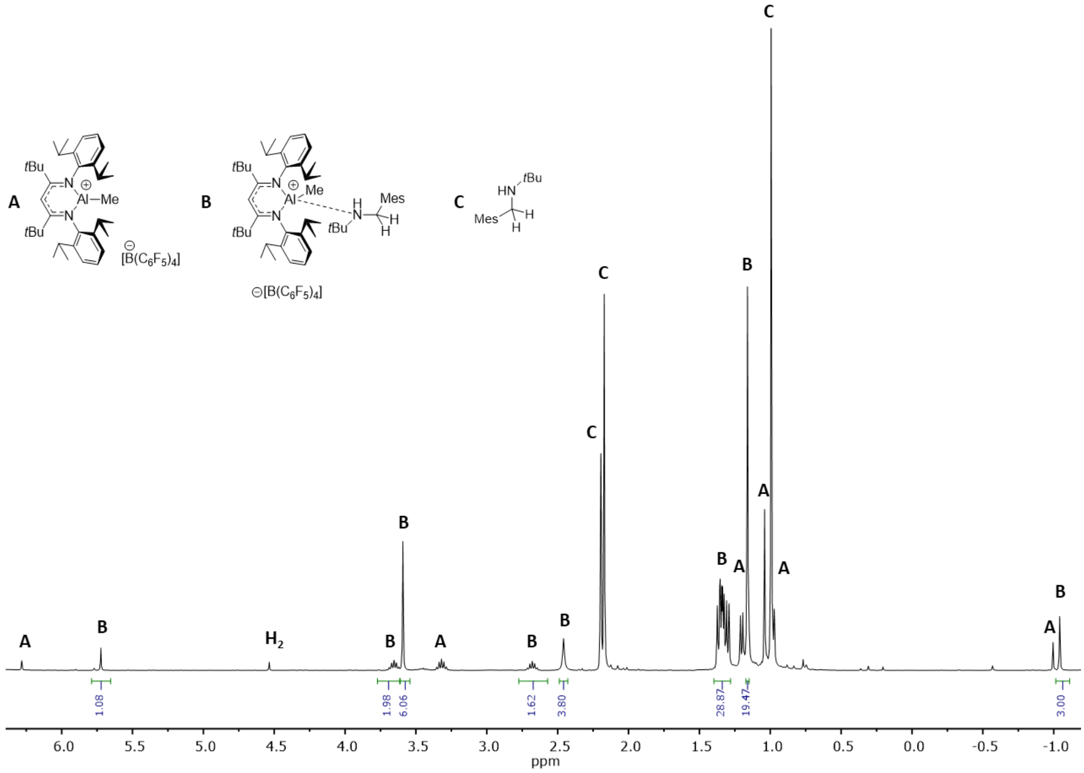


**Figure S55**: Amplified $^1$H NMR spectra of the reaction mixture (Middle, Figure S54).

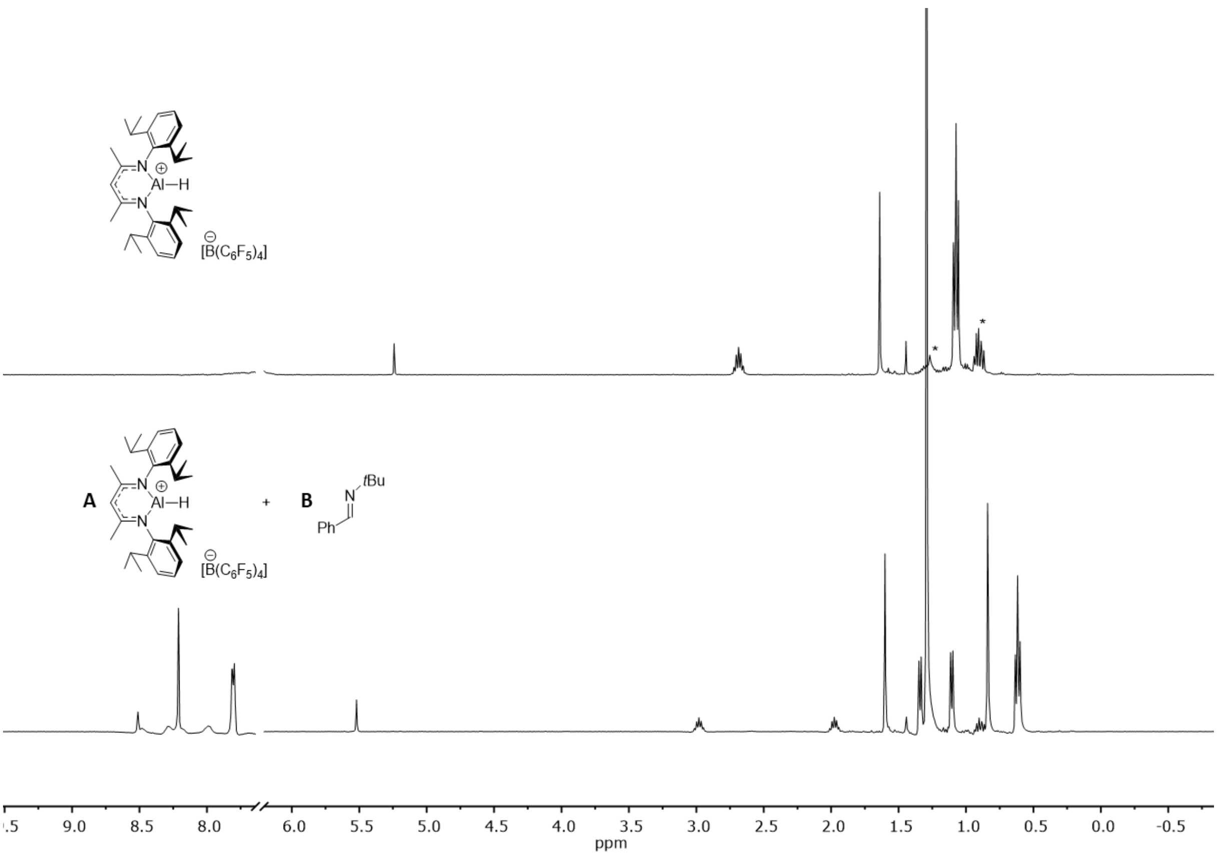


**Figure S56**: $^{1}$H NMR spectra of free [($^{Me,DIPP}$BDI)AlH$^{+}$][B($C_6F_5$)$_4^{-}$] (top, hexane impurities are marked with a star) and formation of a coordination complex upon addition of (Ph)CH=N(tBu) 9 eq. in $C_6D_6$/PhCl (2/1; v/v) (bottom). Area 6.5 to 7.5ppm (signals of PhCl) excluded for clarity.

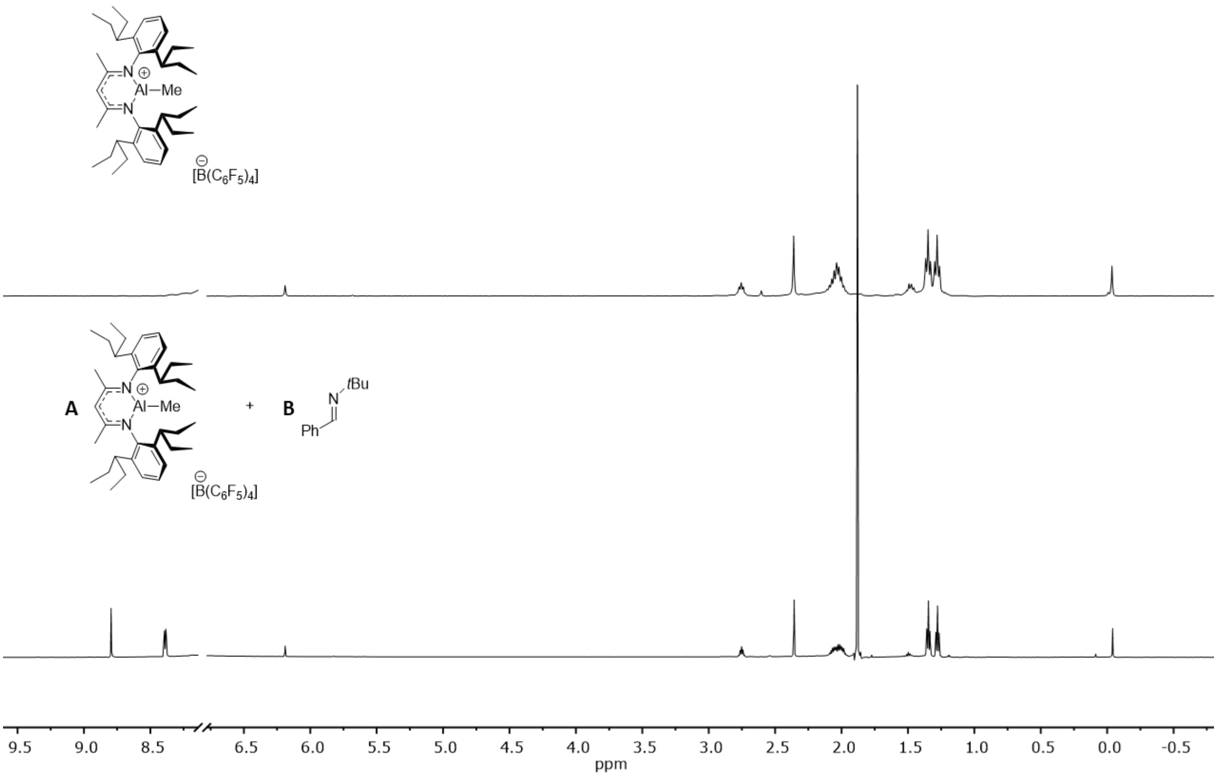


**Figure S57**: [1]H NMR spectra of [($^{Me,DIPeP}$BDI)AlMe$^+$][B($C_6F_5$)$_4^-$] in absence (top) and presence of (Ph)CH=N(tBu) 9 eq. in $C_6D_6$/PhCl (2/1; v/v). No interaction is observed. Area 7.0 to 7.5ppm (signals of PhCl) excluded for clarity.

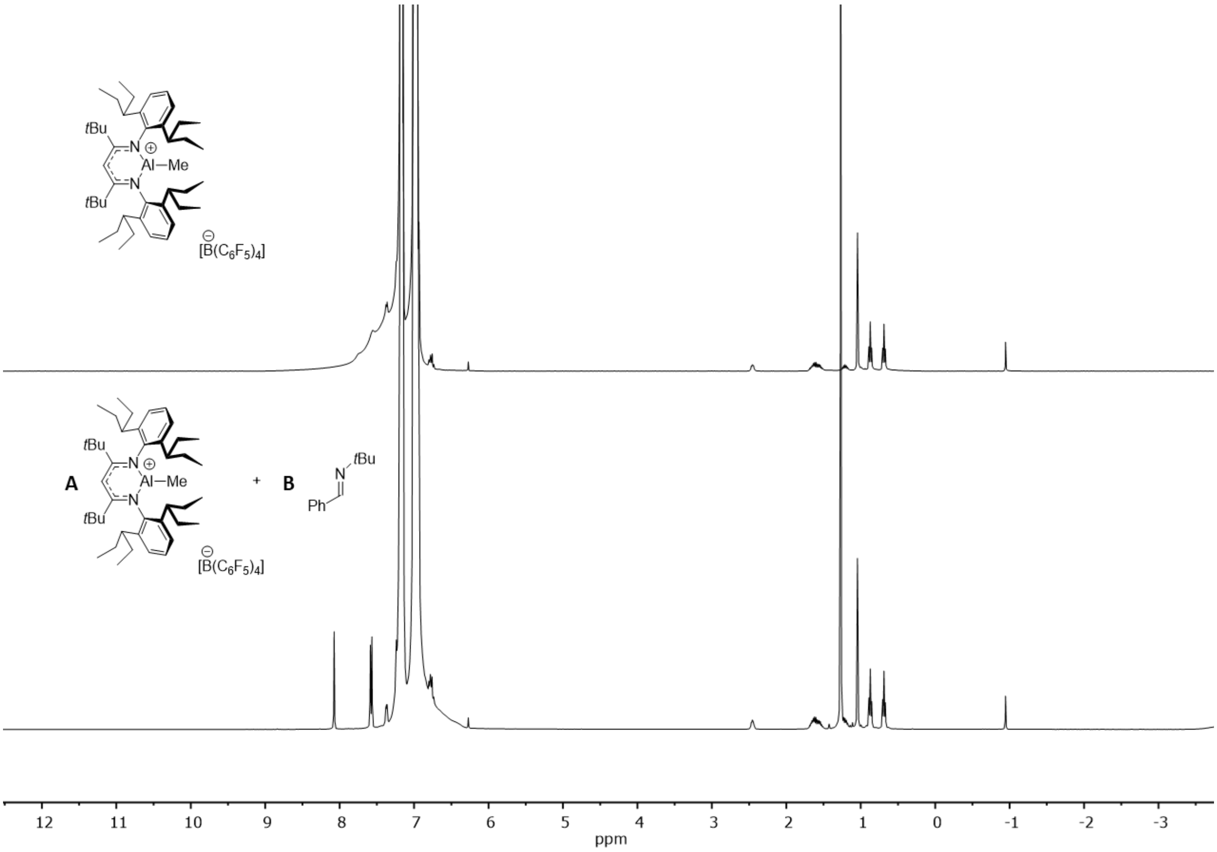


**Figure S58**: $^1$H NMR spectra of [($^{tBu,DIPeP}$BDI)AlMe$^+$][B($C_6F_5$)$_4^-$] in absence (top) and presence of (Ph)CH=N(tBu) 9 eq. in $C_6D_6$/PhCl (2/1; v/v). No interaction is observed. Area 7.0 to 7.5ppm are obscured by signals of PhCl.

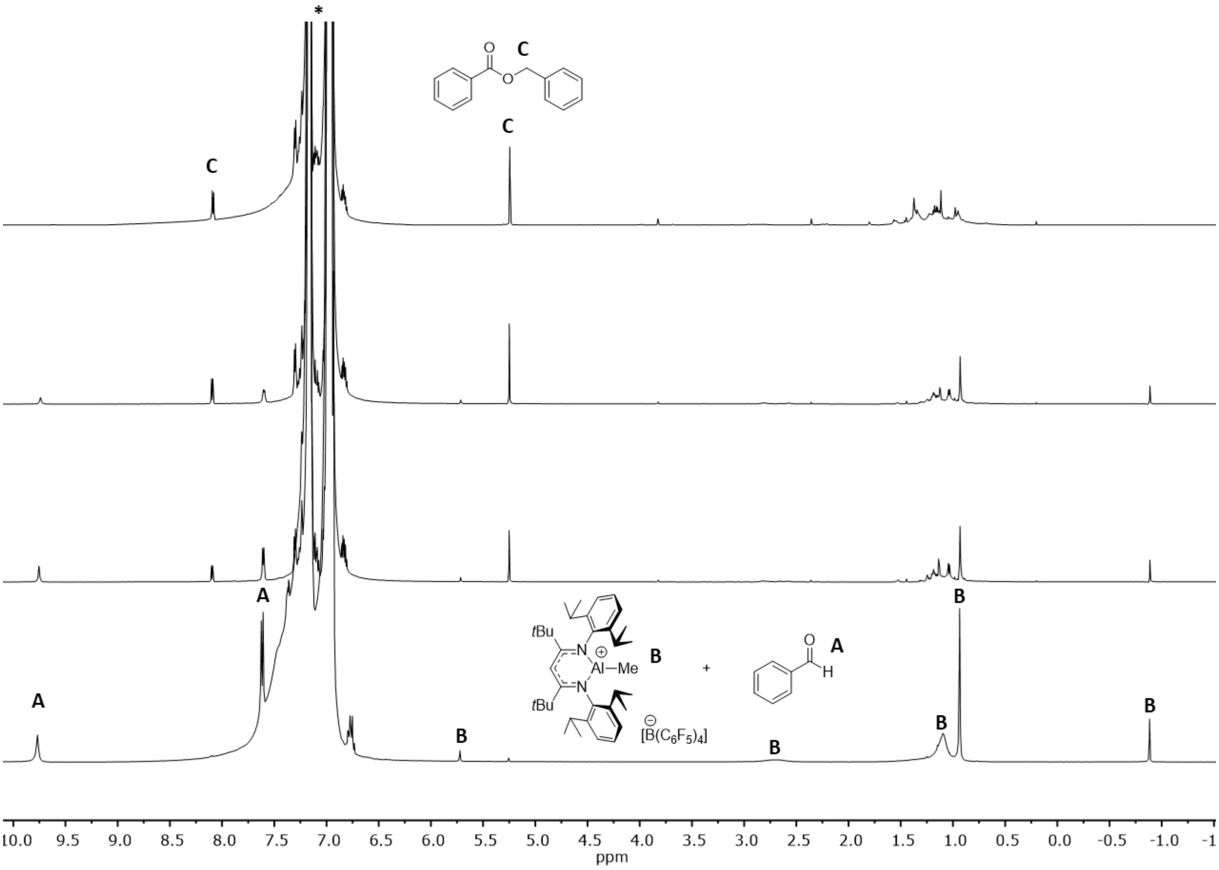


**Figure S59**: $^{1}$H NMR spectra of the catalytic transformation of benzophenone (A) with $[(^{tBu,DIPP}BDI)AlMe^{+}][B(C_6F_5)_4^{-}]$ (10 mol%) (B) at 60 °C in $C_6D_6$/PhCl (2/1; v/v, area marked with a star) to benzyl benzoate (C).

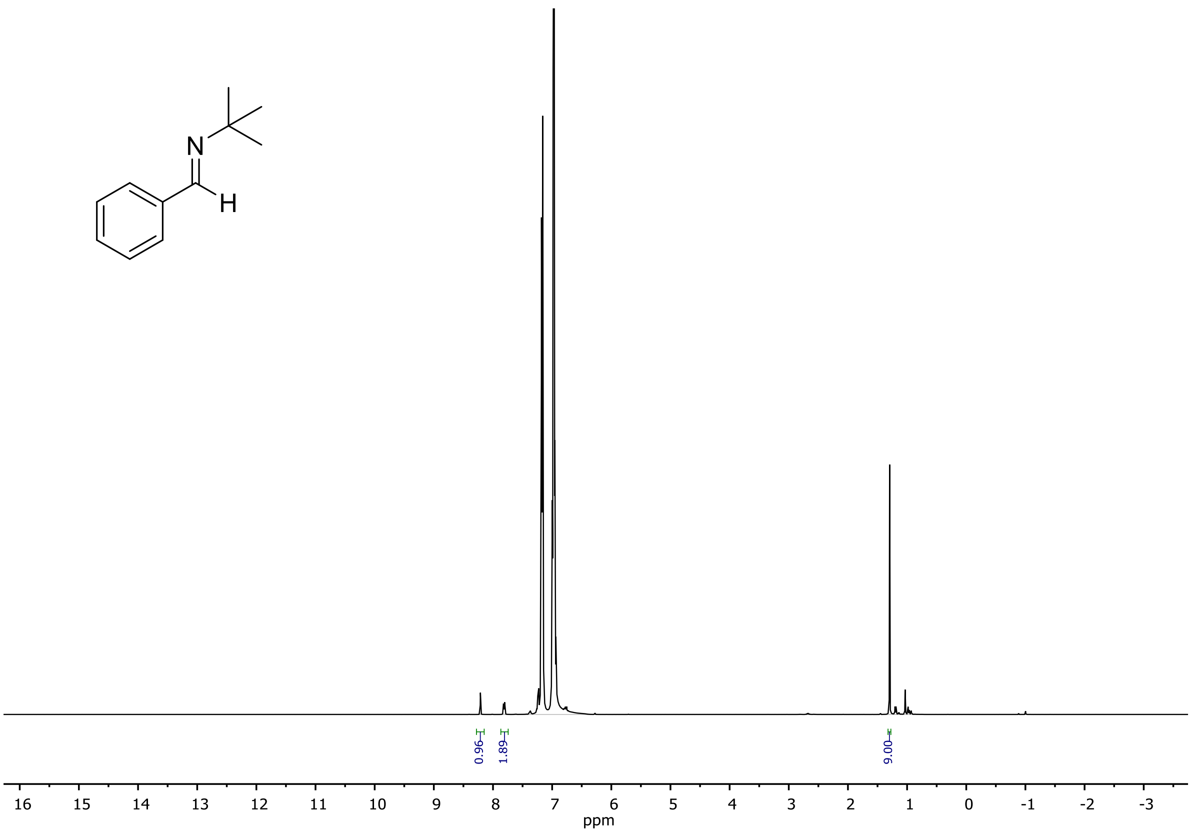


**Figure S60**: [1]H NMR (400 MHz, 298 K) spectrum of imine hydrogenation in $C_6D_6$/PhCl (2/1; v/v) – before $H_2$ addition.

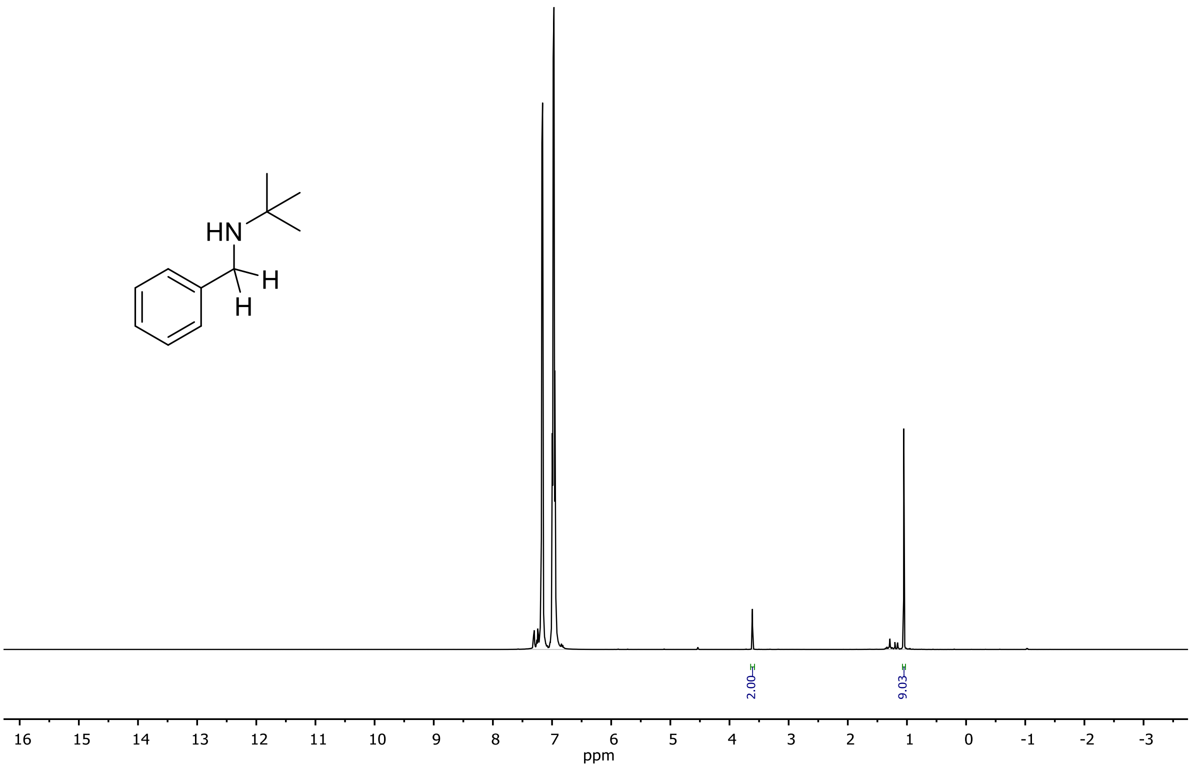


**Figure S61**: [1]H NMR (600 MHz, 298 K) spectrum of imine hydrogenation in $C_6D_6$/PhCl (2/1; v/v) – after catalysis.

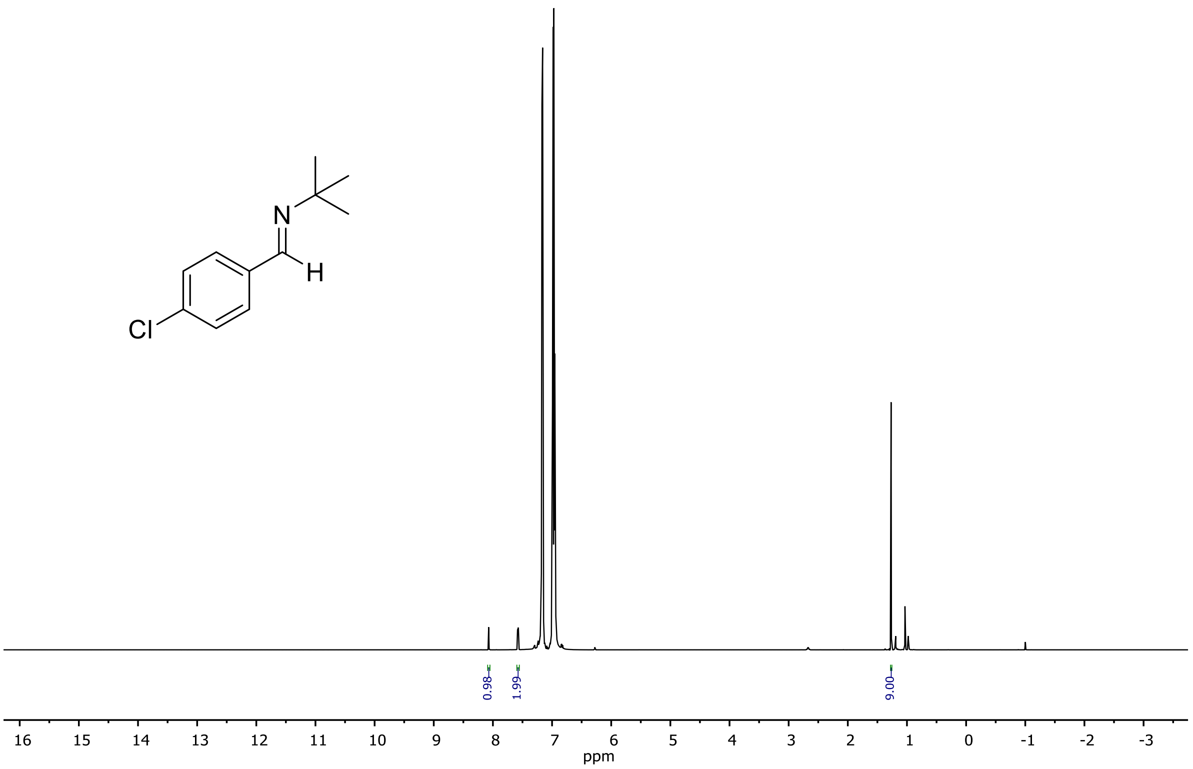


**Figure S62**: [1]H NMR (600 MHz, 298 K) spectrum of imine hydrogenation in $C_6D_6$/PhCl (2/1; v/v) – before $H_2$ addition.

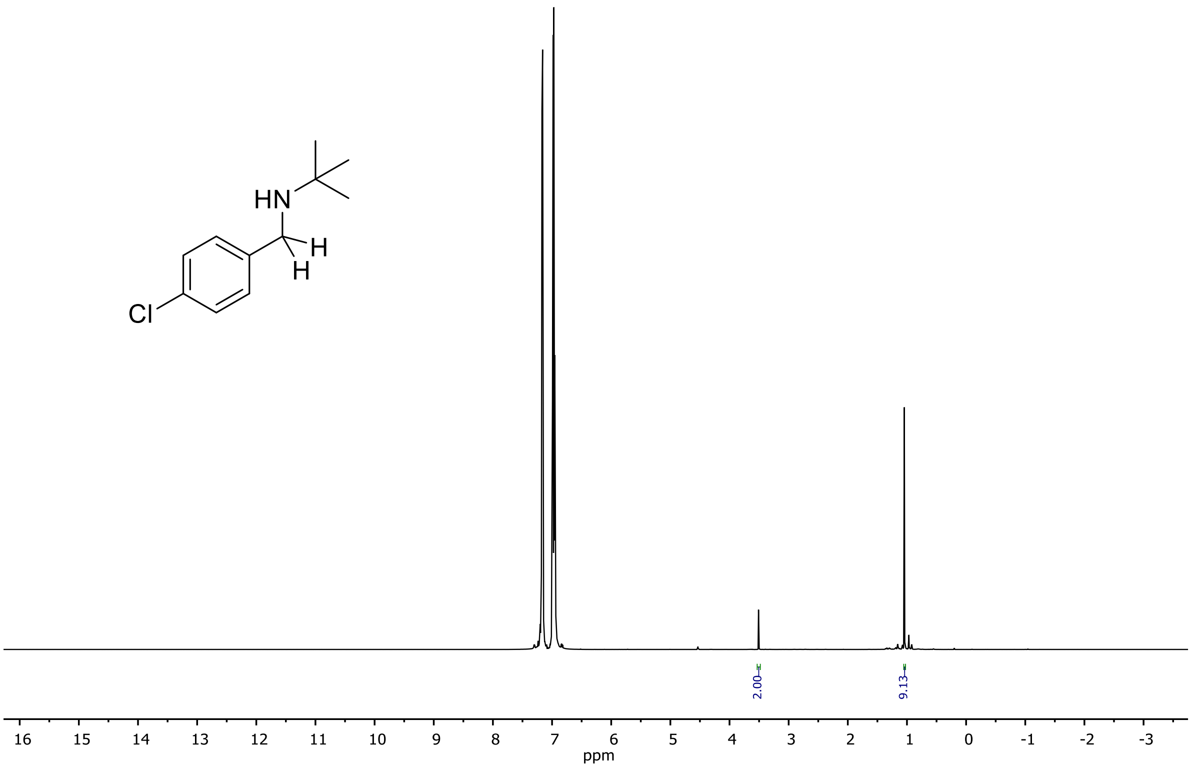


**Figure S63**: [1]H NMR (600 MHz, 298 K) spectrum of imine hydrogenation in $C_6D_6$/PhCl (2/1; v/v) – after catalysis.

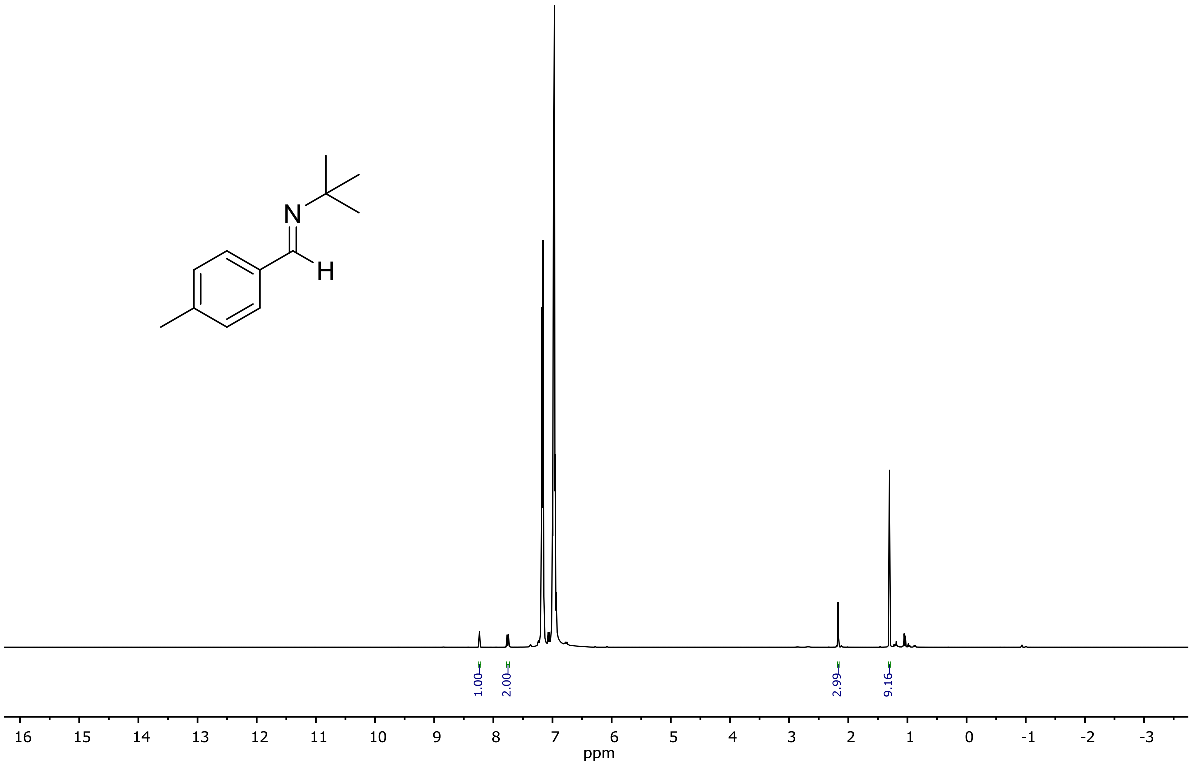


**Figure S64**: $^1$H NMR (400 MHz, 298 K) spectrum of imine hydrogenation in $C_6D_6$/PhCl (2/1; v/v) – before $H_2$ addition.

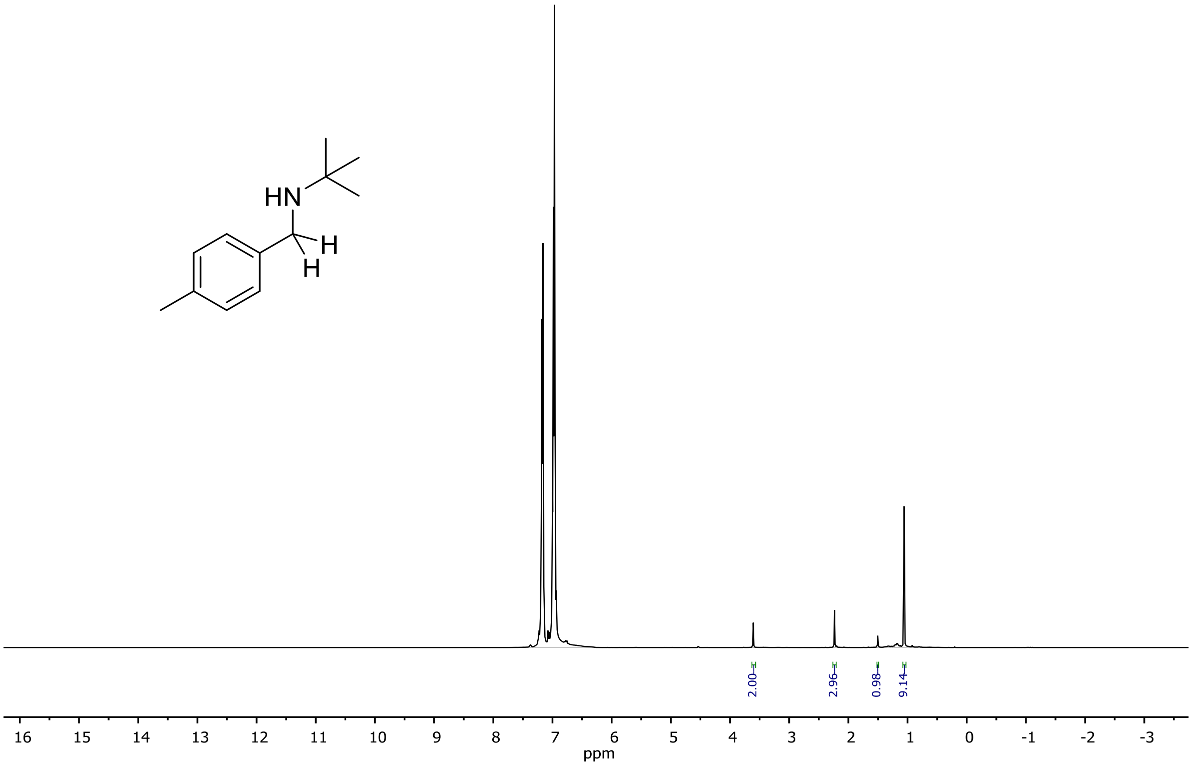


**Figure S65**: $^1$H NMR (400 MHz, 298 K) spectrum of imine hydrogenation in $C_6D_6$/PhCl (2/1; v/v) – after catalysis.

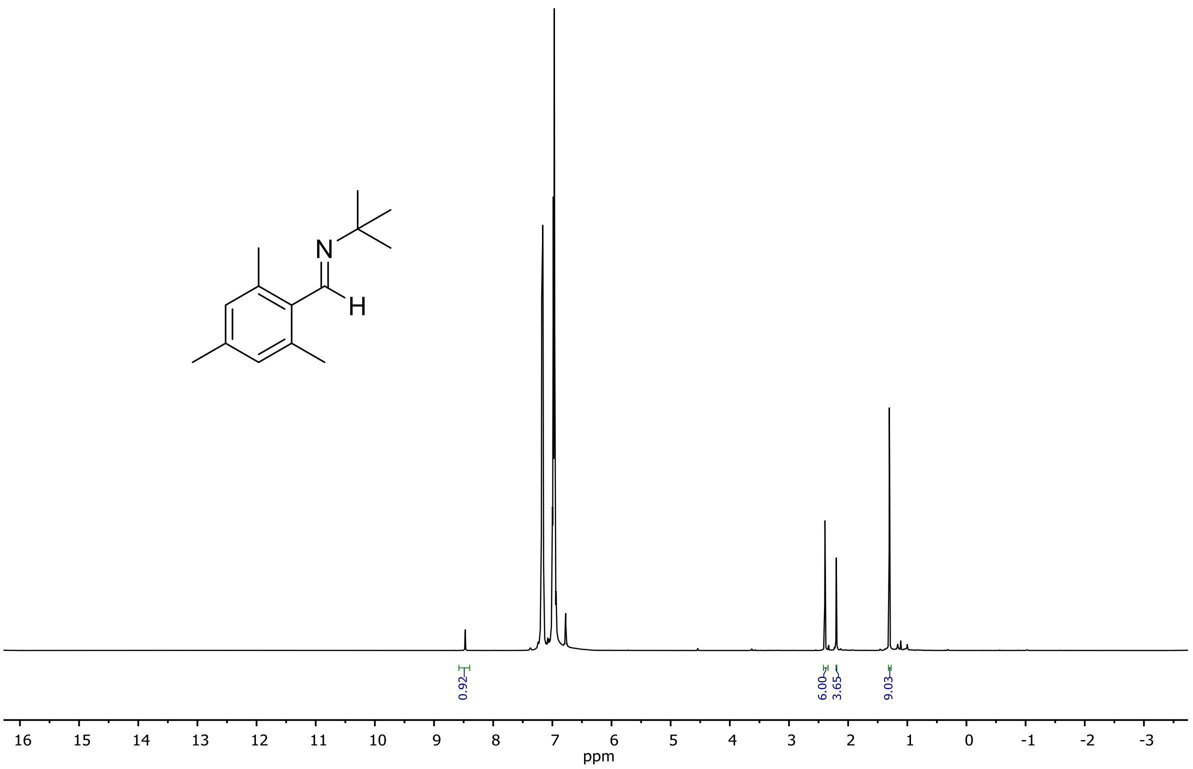


**Figure S66**: $^{1}H$ NMR (400 MHz, 298 K) spectrum of imine hydrogenation in $C_6D_6$/PhCl (2/1; v/v) – before $H_2$ addition.

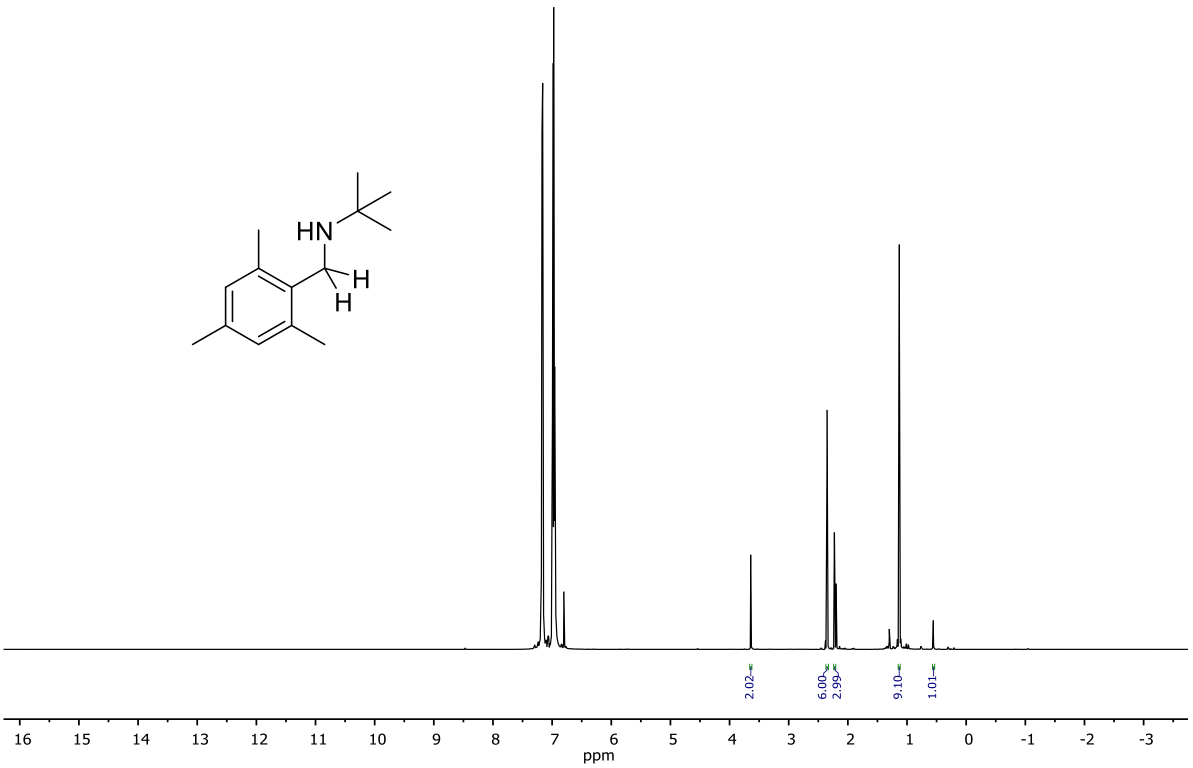


**Figure S67**: $^{1}H$ NMR (600 MHz, 298 K) spectrum of imine hydrogenation in $C_6D_6$/PhCl (2/1; v/v) – after catalysis.

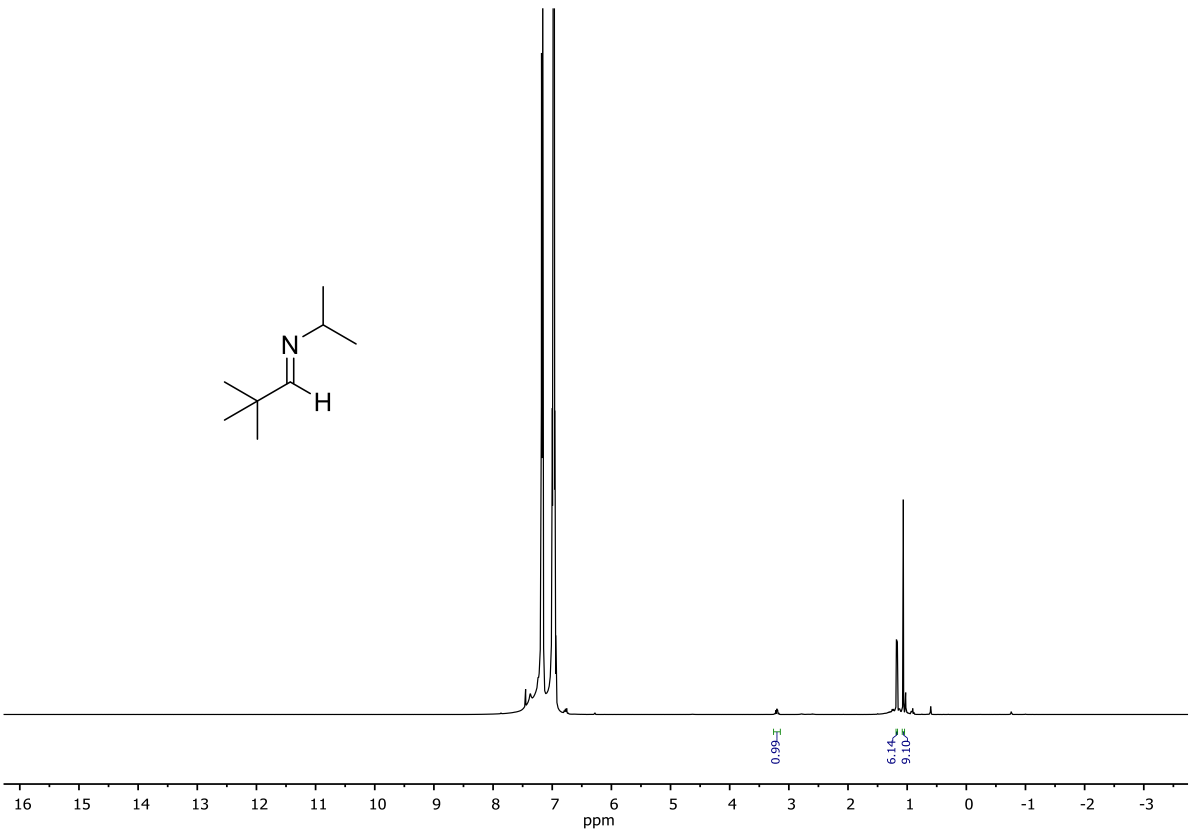


**Figure S68**: [1]H NMR (400 MHz, 298 K) spectrum of imine hydrogenation in $C_6D_6$/PhCl (2/1; v/v) – before $H_2$ addition.

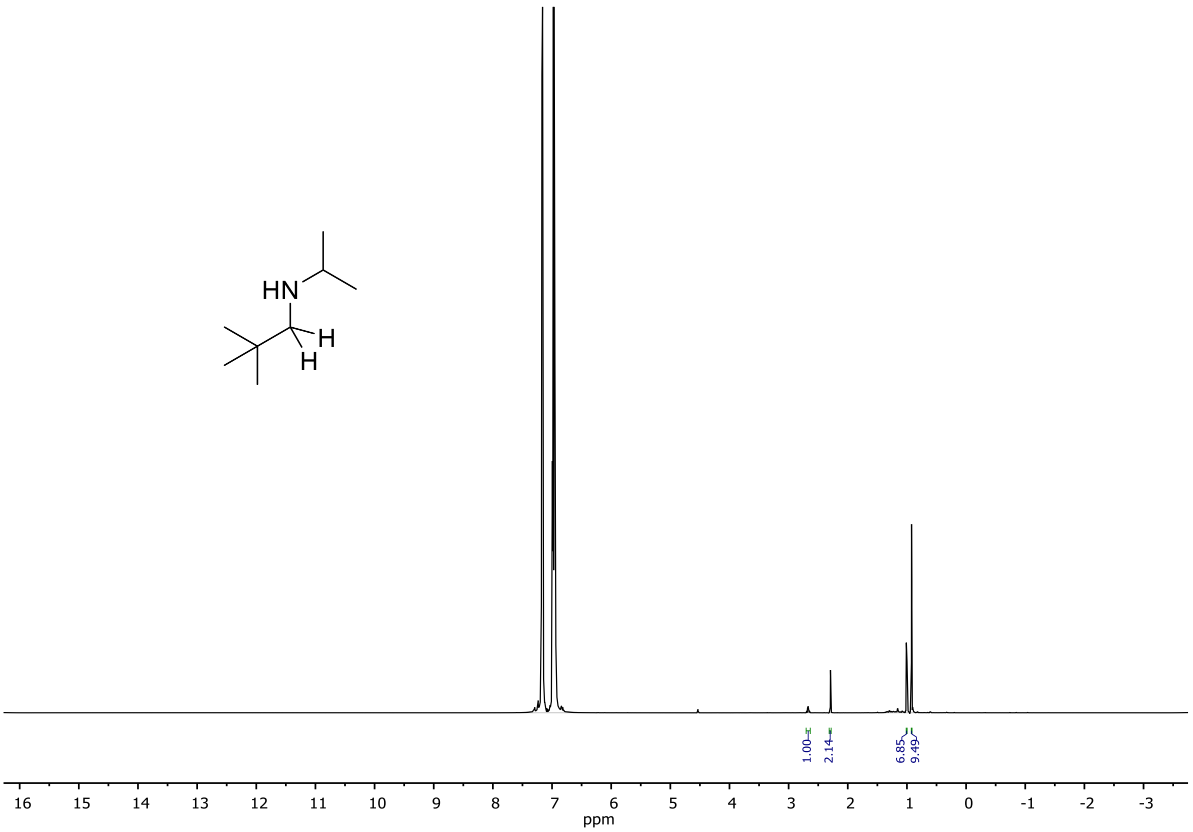


**Figure S69**: [1]H NMR (600 MHz, 298 K) spectrum of imine hydrogenation in $C_6D_6$/PhCl (2/1; v/v) – after catalysis.

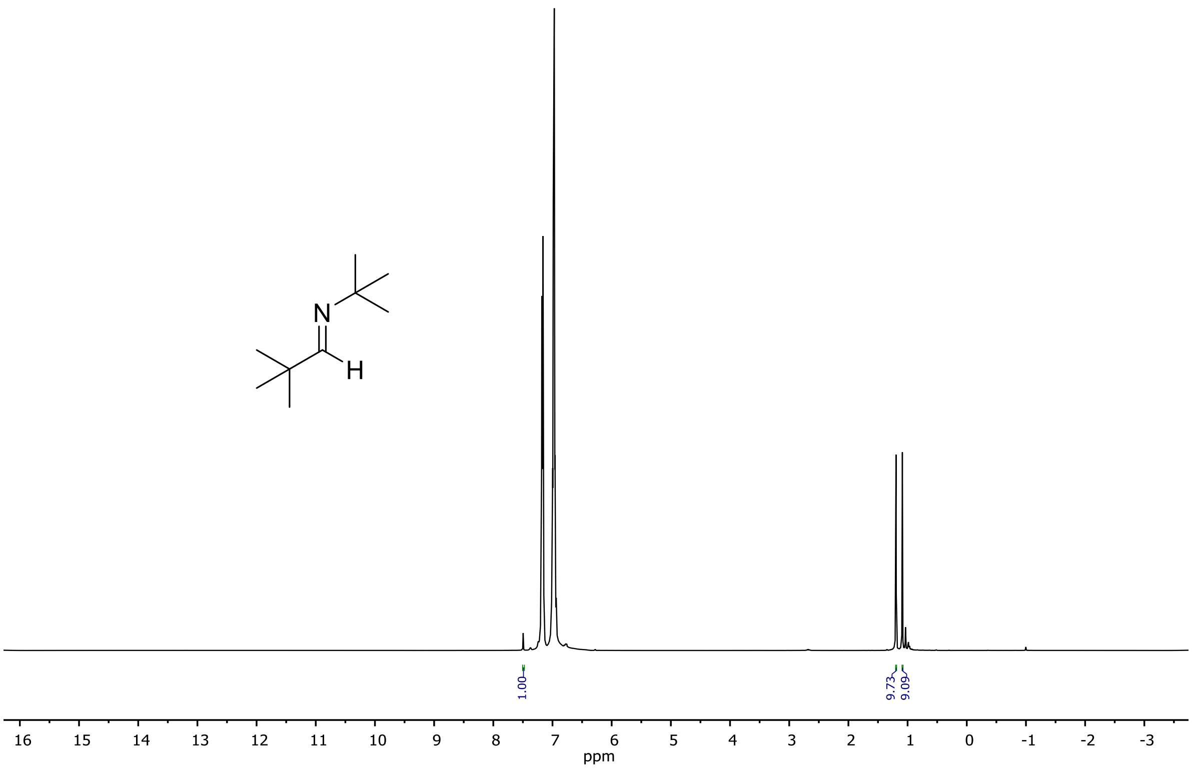


**Figure S70**: $^1$H NMR (400 MHz, 298 K) spectrum of imine hydrogenation in $C_6D_6$/PhCl (2/1; v/v) – before $H_2$ addition.

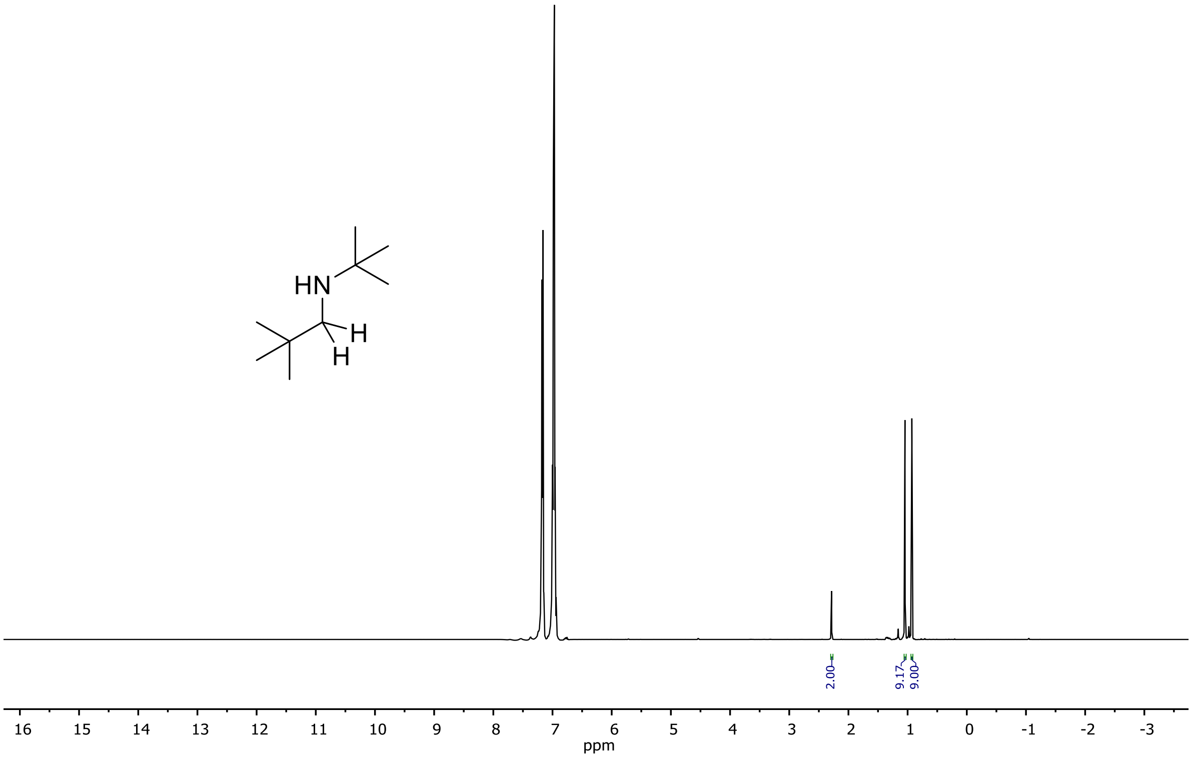


**Figure S71**: $^1$H NMR (400 MHz, 298 K) spectrum of imine hydrogenation in $C_6D_6$/PhCl (2/1; v/v) – after catalysis.

## 1.5 Single Crystal X-Ray Diffraction

A colorless crystal of the corresponding compound was embedded in inert perfluoropolyalkylether (viscosity 1800 cSt; ABCR GmbH) and mounted using a Hampton Research CryoLoop. The crystal was then flash cooled to 100.0(2) K in a nitrogen gas stream and kept at this temperature during the experiment. The crystal structure was measured on a SuperNova diffractometer with Atlas S2 detector using a CuKα microfocus source. The measured data was processed with the CrysAlisPro (v40.53) software package.[S6] Using Olex2 [S7], the structure was solved with the ShelXT [S8] structure solution program using Intrinsic Phasing and refined with the ShelXL [S9] refinement package using Least Squares minimization. All non-hydrogen atoms were refined anisotropically. ($^{t\text{Bu,DIPP}}$BDI)AlMe$_2$: (CCDC: 2063692) All hydrogen atoms were placed in ideal positions and refined as riding atoms with relative isotropic displacement parameters. ($^{t\text{Bu,DIPP}}$BDI)AlH$_2$: (CCDC: 2063693) Most hydrogen atoms were placed in ideal positions and refined as riding atoms with relative isotropic displacement parameters. The positions of the aluminum bound hydrides were observed from difference Fourier maps and refined. ($^{t\text{Bu,DIPP}}$BDI)Al(Me)Cl: (CCDC: 2063694) All hydrogen atoms were placed in ideal positions and refined as riding atoms with relative isotropic displacement parameters. ($^{\text{Me,DIPeP}}$BDI-H)·AlMe$_3$: (CCDC: 2063695) The crystal under investigation showed racemic twinning. The fractional contributions of the two twin domains were refined to 0.60(4) and 0.40(4) later on. Using Olex2,[S7] the structure was solved with the ShelXT[S8] structure solution program using Intrinsic Phasing and refined with the ShelXL[S9] refinement package using Least Squares Minimization. All hydrogen atoms were placed in ideal positions and refined as riding atoms with relative isotropic displacement parameters. Disorder of both 2,6-di(3-pentyl)phenyl moieties was observed. The disorder was modeled with the help of similarity restraints (SIMU, SADI) and rigid bond restraints (RIGU).[S10] The relative occupancies of the two alternative orientations of the 2,6-di(3-pentyl)phenyl groups were refined to 0.771(3)/0.229(3) (moiety 1) and 0.719(7)/0.281(7) (moiety 2), respectively. One of the 3-pentyl groups within the major orientation of moiety 1 was more severely disordered (disorder within a disorder) and a third orientation was introduced. The relative occupancies for this 3-pentyl group group were refined to 0.612(4), 0.229(3) and 0.159(4). ($^{\text{Me,DIPeP}}$BDI)AlMe$_2$: (CCDC: 2063696) Most hydrogen atoms were placed in ideal positions and refined as riding atoms with relative isotropic displacement parameters. Since the hydrogen atom at C3 in the ligand backbone deviates slightly from the idealized geometry, its position was located from difference Fourier maps and refined. ($^{t\text{Bu,DIPeP}}$BDI)AlMe$_2$: (CCDC: 2063697) All hydrogen atoms were placed in ideal positions and refined as riding atoms with relative isotropic displacement parameters. [($^{t\text{Bu,DIPP}}$BDI)AlMe$^+$][B(C$_6$F$_5$)$_4^-$]: (CCDC: 2063698) All hydrogen atoms were placed in ideal positions and refined as riding atoms with relative isotropic displacement parameters. Disorder of one C$_6$F$_5$ group of the anion was observed. The disorder was modeled with the help of similarity restraints (SIMU, SADI). The relative occupancies of the two alternative orientations of this

group were refined to 0.768(10) and 0.232(10). Additionally, a substitutional disorder between chlorobenzene and *n*-hexane was found. This disorder was further complicated by close proximity to a crystallographic inversion center. Beside similarity restraints (SIMU, SADI), rigid bond restraints (RIGU)[S10] and a FLAT restraint were applied to create a sufficient solvent model. [($^{tBu,DIPeP}$BDI)AlMe$^+$][B($C_6F_5$)$_4^-$]: (CCDC: 2063699) All hydrogen atoms were placed in ideal positions and refined as riding atoms with relative isotropic displacement parameters. Disorder of one DIPeP–N fragment was observed and the two alternative orientations were refined to 0.586(4)/0.414(4). Disorder of the other DIPeP–N fragment was observed at the isopentyl moieties. The relative occupancies of the two alternative orientations were refined to 0.641(10)/0.359(10) and 0.786(7)/0.214(7), respectively. [($^{tBu}$N(H)C(H)Ph)$^+$] [B($C_6F_5$)$_4^-$]: (CCDC: 2063700) Most hydrogen atoms were placed in ideal positions and refined as riding atoms with relative isotropic displacement parameters. The hydrogen atoms at N1 were located from difference Fourier maps and refined. [($^{tBu}$N($H_2$)C($H_2$)(*p*-Cl$C_6H_4$))$^+$][B($C_6F_5$)$_4^-$]: (CCDC: 2063701) The crystal under investigation showed racemic twinning. The fractional contributions of the two twin domains were refined to 0.56(3) and 0.44(3) later on. Using Olex2,[S7] the structure was solved with the ShelXT[S8] structure solution program using Intrinsic Phasing and refined with the ShelXL[S9] refinement package using Least Squares Minimization. Most hydrogen atoms were placed in ideal positions and refined as riding atoms with relative isotropic displacement parameters. The hydrogen atoms at N1 were located from difference Fourier maps and refined. [(($^{tBu}$N(H)C($H_2$)(*p*-Me$C_6H_5$))···($^{tBu}$N($H_2$)C($H_2$)(*p*-Me$C_6H_4$)$^+$][B($C_6F_5$)$_4^-$]: (CCDC: 2063702) Most hydrogen atoms were placed in ideal positions and refined as riding atoms with relative isotropic displacement parameters. The hydrogen atoms at N1 and N2 were located from difference Fourier maps and refined. The hydrogen atom, which forms the hydrogen bridge between amine and ammonium moiety, is disorder over two positions and was refined with the help of similarity restraints (SADI, SIMU). Additionally, the co-crystallized solvent is heavily disordered. A suitable disorder model for these solvent molecules could not be built. Since the exact nature of the incorporated solvent is uncertain (a mixture of chlorobenzene, benzene and hexanes (isomeric mixture) was used for crystallization), its contribution to the structure factors was secured by back-Fourier transformation using the solvent mask routine[S11,S12] of the program Olex2.[S7] The solvent accessible voids treated this way had a size of 401.4 Å$^3$ (15.8% of the unit cell) and contained 97.5 electrons/unit cell.

**Table S1.** Crystal data.

| Identification code | ($^{tBu,DIPP}$BDI)AlMe$_2$ | ($^{tBu,DIPP}$BDI)AlH$_2$ | ($^{tBu,DIPP}$BDI)Al(Me)Cl | ($^{Me,DIPeP}$BDI-H)AlMe$_3$ |
|---|---|---|---|---|
| **Empirical formula** | $C_{37}H_{59}AlN_2$ | $C_{35}H_{55}AlN_2$ | $C_{36}H_{56}AlClN_2$ | $C_{40}H_{67}AlN_2$ |
| **Formula weight** | 558.84 | 530.79 | 579.25 | 602.93 |
| **Temperature/K** | 100 | 100.0(2) | 100.0(3) | 100.0(1) |
| **Crystal system** | monoclinic | monoclinic | monoclinic | orthorhombic |
| **Space group** | $P2_1/n$ | $P2_1/n$ | $P2_1/n$ | $P2_12_12_1$ |
| **a/Å** | 12.4992(2) | 8.35380(10) | 9.6178(2) | 11.7264(2) |
| **b/Å** | 18.7598(3) | 16.1742(2) | 22.5123(4) | 17.8055(3) |
| **c/Å** | 15.7345(3) | 24.1626(2) | 15.9396(3) | 18.4574(4) |
| **α/°** | 90 | 90 | 90 | 90 |
| **β/°** | 107.191(2) | 98.8130(10) | 91.821(2) | 90 |
| **γ/°** | 90 | 90 | 90 | 90 |
| **Volume/Å$^3$** | 3524.65(12) | 3226.21(6) | 3449.48(11) | 3853.82(14) |
| **Z** | 4 | 4 | 4 | 4 |
| **$\rho_{calc}$g/cm$^3$** | 1.053 | 1.093 | 1.115 | 1.039 |
| **μ/mm$^{-1}$** | 0.673 | 0.713 | 1.401 | 0.644 |
| **F(000)** | 1232.0 | 1168.0 | 1264.0 | 1336.0 |
| **Crystal size/mm$^3$** | 0.4171 × 0.321 × 0.2062 | 0.197 × 0.1082 × 0.0967 | 0.246 × 0.104 × 0.035 | 0.497 × 0.123 × 0.108 |
| **Crystal color** | colorless | colorless | colorless | colorless |
| **Radiation** | CuKα (λ = 1.54184) | CuKα (λ = 1.54184) | Cu Kα (λ = 1.54184) | CuKα (λ = 1.54184) |
| **2Θ range for data collection/°** | 7.536 to 136.23 | 6.6 to 145.85 | 6.798 to 145.3 | 6.898 to 145.952 |
| **Index ranges** | -13 ≤ h ≤ 15, -22 ≤ k ≤ 15, -17 ≤ l ≤ 18 | -9 ≤ h ≤ 10, -19 ≤ k ≤ 19, -29 ≤ l ≤ 29 | -10 ≤ h ≤ 11, -27 ≤ k ≤ 20, -19 ≤ l ≤ 17 | -13 ≤ h ≤ 14, -14 ≤ k ≤ 22, -22 ≤ l ≤ 20 |
| **Reflections collected** | 11983 | 18385 | 17695 | 13487 |
| **Independent reflections** | 6383 [$R_{int}$ = 0.0267, $R_{sigma}$ = 0.0355] | 6280 [$R_{int}$ = 0.0294, $R_{sigma}$ = 0.0273] | 6686 [$R_{int}$ = 0.0441, $R_{sigma}$ = 0.0464] | 7475 [$R_{int}$ = 0.0222, $R_{sigma}$ = 0.0293] |
| **Data/restraints/parameters** | 6383/0/377 | 6280/0/365 | 6686/0/376 | 7475/2069/728 |
| **Goodness-of-fit on F$^2$** | 1.040 | 1.032 | 1.026 | 1.068 |
| **Final R indexes [I>=2σ (I)]** | $R_1$ = 0.0389, $wR_2$ = 0.0990 | $R_1$ = 0.0432, $wR_2$ = 0.1153 | $R_1$ = 0.0513, $wR_2$ = 0.1335 | $R_1$ = 0.0440, $wR_2$ = 0.1166 |
| **Final R indexes [all data]** | $R_1$ = 0.0419, $wR_2$ = 0.1018 | $R_1$ = 0.0459, $wR_2$ = 0.1183 | $R_1$ = 0.0600, $wR_2$ = 0.1427 | $R_1$ = 0.0469, $wR_2$ = 0.1198 |
| **Largest diff. peak/hole / e Å$^{-3}$** | 0.30/-0.26 | 0.29/-0.33 | 0.44/-0.32 | 0.25/-0.20 |

**Table S2.** Crystal data (continued).

| Identification code | $(^{Me,DIPeP}BDI)AlMe_2$ | $(^{tBu,DIPeP}BDI)AlMe_2$ | $[(^{tBu,DIPP}BDI)AlMe^+][B(C_6F_5)_4^-]$ | $[(^{tBu,DIPeP}BDI)AlMe^+][B(C_6F_5)_4^-]$ |
|---|---|---|---|---|
| **Empirical formula** | $C_{39}H_{63}AlN_2$ | $C_{45}H_{75}AlN_2$ | $C_{60}H_{56}AlBF_{20}N_2$ | $C_{68}H_{72}AlBF_{20}N_2$ |
| **Formula weight** | 586.89 | 671.05 | 1222.85 | 1335.06 |
| **Temperature/K** | 100.0(2) | 100.0(3) | 99.9(4) | 100.00(10) |
| **Crystal system** | monoclinic | triclinic | monoclinic | monoclinic |
| **Space group** | $P2_1/n$ | P-1 | $P2_1/c$ | $P2_1/n$ |
| **a/Å** | 12.96933(14) | 10.5343(7) | 19.5489(7) | 18.3540(2) |
| **b/Å** | 15.82666(17) | 10.6204(7) | 17.0798(6) | 17.5697(2) |
| **c/Å** | 17.9813(2) | 21.0812(9) | 18.0647(6) | 20.3106(3) |
| **α/°** | 90 | 101.576(4) | 90 | 90 |
| **β/°** | 99.6080(11) | 92.247(5) | 92.518(3) | 101.1980(10) |
| **γ/°** | 90 | 115.083(6) | 90 | 90 |
| **Volume/Å$^3$** | 3639.08(7) | 2072.0(2) | 6025.8(4) | 6424.95(14) |
| **Z** | 4 | 2 | 4 | 4 |
| **$\rho_{calc}$g/cm$^3$** | 1.071 | 1.076 | 1.348 | 1.380 |
| **μ/mm$^{-1}$** | 0.672 | 0.643 | 1.195 | 1.166 |
| **F(000)** | 1296.0 | 744.0 | 2512.0 | 2768.0 |
| **Crystal size/mm$^3$** | 0.248 × 0.123 × 0.084 | 0.18 × 0.109 × 0.025 | 0.4008 × 0.3142 × 0.1465 | 0.258 × 0.202 × 0.18 |
| **Crystal color** | colorless | colorless | colorless | colorless |
| **Radiation** | Cu Kα (λ = 1.54184) | Cu Kα (λ = 1.54184) | CuKα (λ = 1.54184) | Cu Kα (λ = 1.54184) |
| **2Θ range for data collection/°** | 7.488 to 145.42 | 8.648 to 145.508 | 6.876 to 136.296 | 7.03 to 144.904 |
| **Index ranges** | -15 ≤ h ≤ 15, -18 ≤ k ≤ 19, -21 ≤ l ≤ 22 | -13 ≤ h ≤ 12, -9 ≤ k ≤ 13, -26 ≤ l ≤ 24 | -23 ≤ h ≤ 21, -20 ≤ k ≤ 6, -21 ≤ l ≤ 19 | -22 ≤ h ≤ 19, -21 ≤ k ≤ 21, -24 ≤ l ≤ 18 |
| **Reflections collected** | 13707 | 12943 | 22185 | 36429 |
| **Independent reflections** | 7003 [$R_{int}$ = 0.0207, $R_{sigma}$ = 0.0277] | 7876 [$R_{int}$ = 0.0431, $R_{sigma}$ = 0.0696] | 10933 [$R_{int}$ = 0.0307, $R_{sigma}$ = 0.0402] | 12390 [$R_{int}$ = 0.0369, $R_{sigma}$ = 0.0330] |
| **Data/restraints/parameters** | 7003/0/395 | 7876/0/449 | 10933/72/860 | 12390/0/1024 |
| **Goodness-of-fit on F$^2$** | 1.054 | 1.022 | 1.051 | 1.021 |
| **Final R indexes [I>=2σ (I)]** | $R_1$ = 0.0371, $wR_2$ = 0.0937 | $R_1$ = 0.0528, $wR_2$ = 0.1270 | $R_1$ = 0.0480, $wR_2$ = 0.1300 | $R_1$ = 0.0493, $wR_2$ = 0.1267 |
| **Final R indexes [all data]** | $R_1$ = 0.0426, $wR_2$ = 0.0976 | $R_1$ = 0.0765, $wR_2$ = 0.1409 | $R_1$ = 0.0526, $wR_2$ = 0.1340 | $R_1$ = 0.0556, $wR_2$ = 0.1331 |
| **Largest diff. peak/hole / e Å$^{-3}$** | 0.25/-0.30 | 0.27/-0.41 | 0.73/-0.55 | 0.50/-0.40 |

**Table S1.** Crystal data (continued).

| Identification code | $[(^{tBu}N(H)C(H)Ph)^+][B(C_6F_5)_4^-]$ | $[(^{tBu}N(H_2)C(H_2)(p\text{-}ClC_6H_4))^+][B(C_6F_5)_4^-]$ | $[((^{tBu}N(H)C(H_2)(p\text{-}MeC_6H_5))\cdots(^{tBu}N(H_2)C(H_2)(p\text{-}MeC_6H_4)^+][B(C_6F_5)_4^-]$ |
|---|---|---|---|
| **Empirical formula** | $C_{35}H_{16}BF_{20}N$ | $C_{35}H_{17}BClF_{20}N$ | $C_{48}H_{39}BF_{20}N_2$ |
| **Formula weight** | 841.30 | 877.76 | 1034.62 |
| **Temperature/K** | 100.0(1) | 100.0(1) | 100.0(1) |
| **Crystal system** | monoclinic | orthorhombic | triclinic |
| **Space group** | $P2_1/n$ | $Pna2_1$ | P-1 |
| **a/Å** | 12.9468(2) | 14.9913(2) | 13.7233(6) |
| **b/Å** | 19.9030(3) | 10.37683(16) | 14.3907(6) |
| **c/Å** | 13.0502(2) | 21.2835(3) | 14.9325(6) |
| **α/°** | 90 | 90 | 92.104(3) |
| **β/°** | 106.2488(17) | 90 | 111.992(4) |
| **γ/°** | 90 | 90 | 109.514(4) |
| **Volume/Å$^3$** | 3228.46(9) | 3310.89(9) | 2532.8(2) |
| **Z** | 4 | 4 | 2 |
| **$\rho_{calc}$g/cm$^3$** | 1.731 | 1.761 | 1.357 |
| **μ/mm$^{-1}$** | 1.652 | 2.365 | 1.160 |
| **F(000)** | 1672.0 | 1744.0 | 1052.0 |
| **Crystal size/mm$^3$** | 0.218 × 0.195 × 0.164 | 0.17 × 0.104 × 0.081 | 0.274 × 0.066 × 0.052 |
| **Crystal color** | colorless | colorless | colorless |
| **Radiation** | CuKα (λ = 1.54184) | CuKα (λ = 1.54184) | CuKα (λ = 1.54184) |
| **2Θ range for data collection/°** | 8.34 to 146.358 | 5.896 to 147.048 | 6.496 to 145.318 |
| **Index ranges** | -15 ≤ h ≤ 11, -24 ≤ k ≤ 20, -15 ≤ l ≤ 16 | -18 ≤ h ≤ 18, -12 ≤ k ≤ 11, -25 ≤ l ≤ 23 | -16 ≤ h ≤ 15, -17 ≤ k ≤ 16, -17 ≤ l ≤ 18 |
| **Reflections collected** | 11745 | 11105 | 16141 |
| **Independent reflections** | 6259 [$R_{int}$ = 0.0192, $R_{sigma}$ = 0.0267] | 5009 [$R_{int}$ = 0.0311, $R_{sigma}$ = 0.0367] | 9697 [$R_{int}$ = 0.0431, $R_{sigma}$ = 0.0627] |
| **Data/restraints/parameters** | 6259/0/521 | 5009/1/527 | 9697/2/665 |
| **Goodness-of-fit on F$^2$** | 1.030 | 1.058 | 0.983 |
| **Final R indexes [I>=2σ (I)]** | $R_1$ = 0.0356, $wR_2$ = 0.0907 | $R_1$ = 0.0371, $wR_2$ = 0.0993 | $R_1$ = 0.0571, $wR_2$ = 0.1460 |
| **Final R indexes [all data]** | $R_1$ = 0.0405, $wR_2$ = 0.0945 | $R_1$ = 0.0403, $wR_2$ = 0.1024 | $R_1$ = 0.0719, $wR_2$ = 0.1606 |
| **Largest diff. peak/hole / e Å$^{-3}$** | 0.47/-0.38 | 0.19/-0.25 | 0.30/-0.38 |

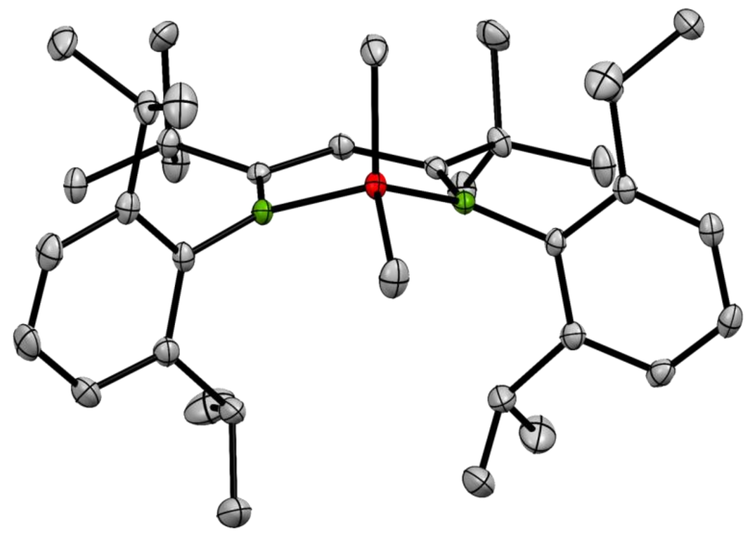

**Figure S72:** ORTEP representation of ($^{tBu,DIPP}$BDI)$AlMe_2$ (probability level 50%). Hydrogen atoms were omitted for clarity.

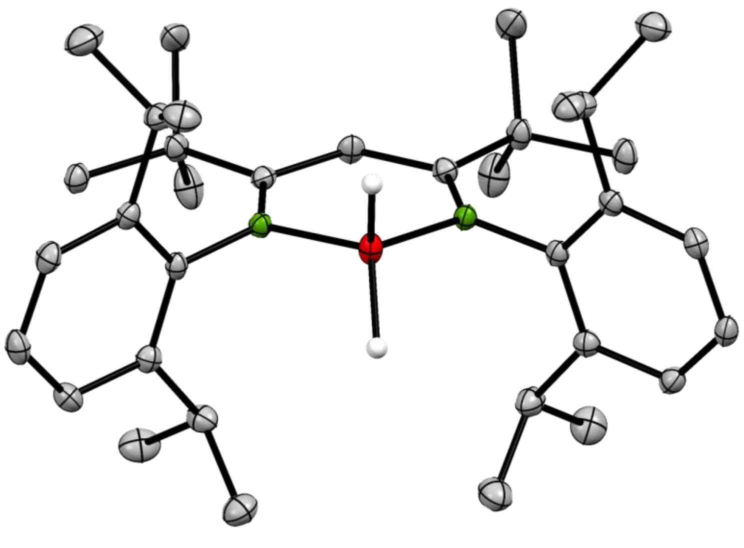

**Figure S73:** ORTEP representation of ($^{tBu,DIPP}$BDI)$AlH_2$ (probability level 50%). Hydrogen atoms were partially omitted for clarity.

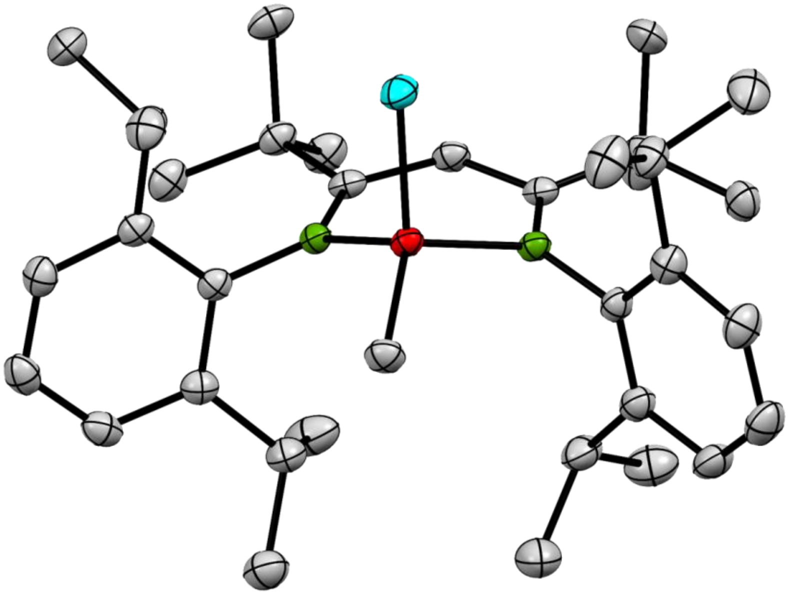

**Figure S74:** ORTEP representation of ($^{tBu,DIPP}$BDI)AlMeCl (probability level 50%). Hydrogen atoms were omitted for clarity.

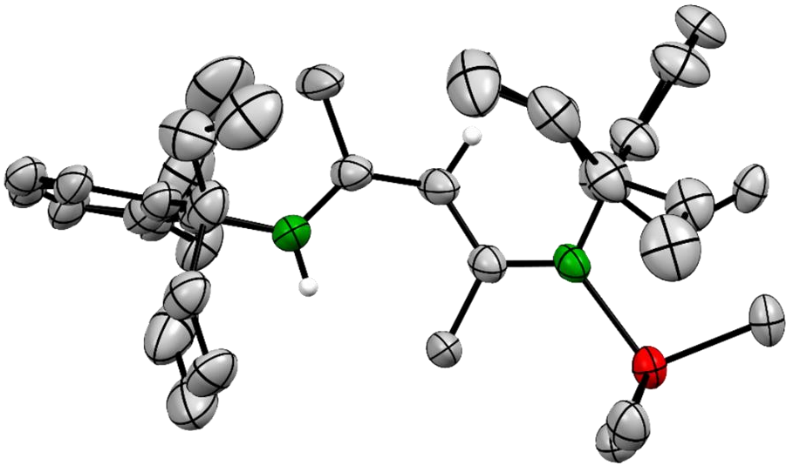

**Figure S75:** ORTEP representation of ($^{Me,DIPeP}$BDI-H)·$AlMe_3$ (probability level 50%). Hydrogen atoms were omitted for clarity.

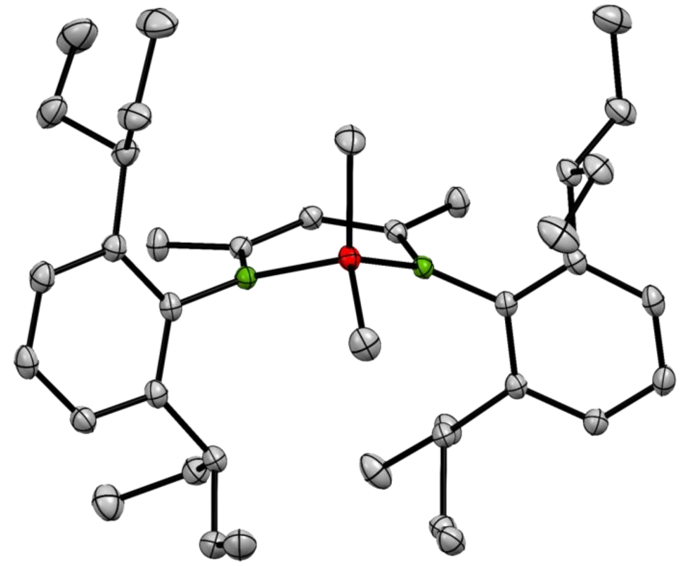

**Figure S76:** ORTEP representation of ($^{Me,DIPeP}$BDI)$AlMe_2$ (probability level 50%). Hydrogen atoms were omitted for clarity.

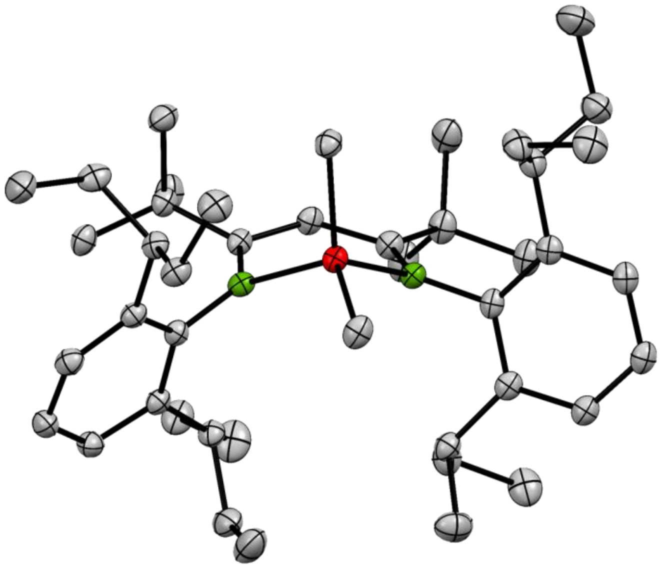

**Figure S77:** ORTEP representation of ($^{tBu,DIPeP}$BDI)$AlMe_2$ (probability level 50%). Hydrogen atoms were omitted for clarity.

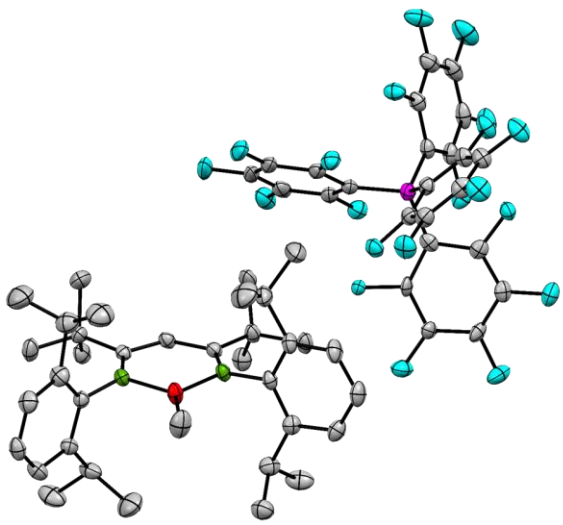

**Figure S78:** ORTEP representation of [($^{tBu}$BDI)AlMe$^{+}$][B($C_6F_5$)$_4^{-}$] (probability level 50%). Hydrogen atoms were omitted for clarity.

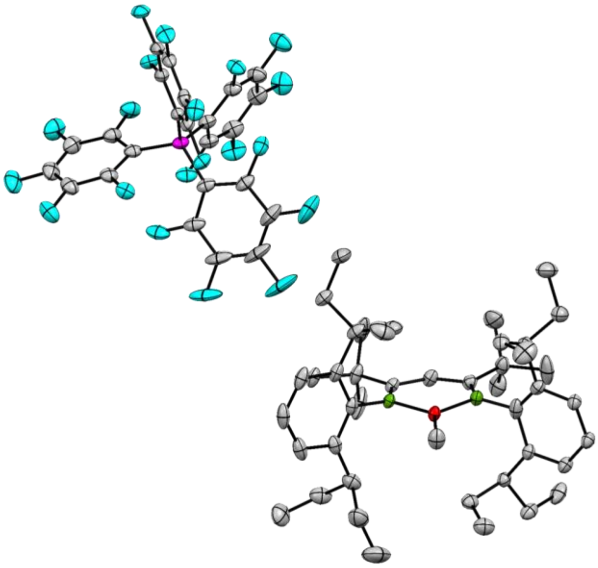

**Figure S79:** ORTEP representation of [($^{tBu,DIPeP}$BDI)AlMe$^{+}$][B($C_6F_5$)$_4^{-}$] (probability level 50%). Hydrogen atoms were omitted for clarity.

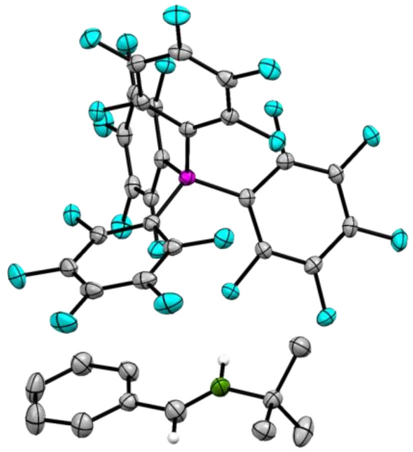

**Figure S80:** ORTEP representation of iminium salt [($^{tBu}$N(H)C(H)Ph)$^{+}$][B($C_6F_5$)$_4$$^{-}$] (probability level 50%). Hydrogen atoms were partially omitted for clarity.

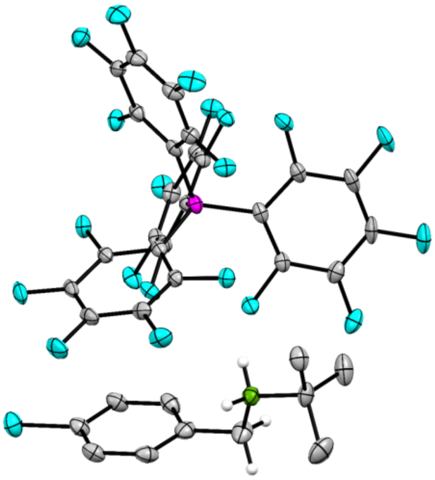

**Figure S81:** ORTEP representation of ammonium salt [($^{tBu}$N($H_2$)C($H_2$)(*p*-Cl$C_6H_4$))$^{+}$][B($C_6F_5$)$_4$$^{-}$] (probability level 50%). Hydrogen atoms were partially omitted for clarity.

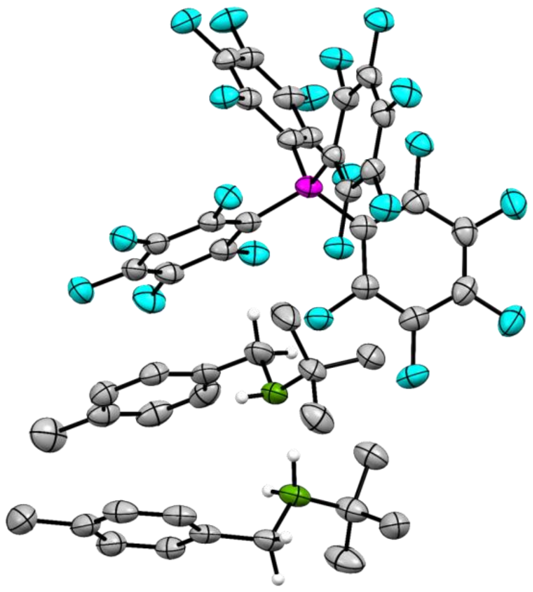

**Figure S82:** ORTEP representation of mixed amine-ammonium salt [((${}^{tBu}$N(H)C($H_2$)(*p*-Me$C_6H_5$))···(${}^{tBu}$N($H_2$)C($H_2$)(*p*-Me$C_6H_4$)$^+$][B($C_6F_5$)$_4^-$] (probability level 50%). Hydrogen atoms were partially omitted for clarity.

## 1.6 Computational details

All calculations were carried out using Gaussian 16A.[S13] All methods were used as implemented. All structures were fully optimized on a B3PW91/def2SVP level of theory.[S14] All structures were characterized as true minima (Nimag = 0) or transition states (Nimag = 1) by frequency calculations on the same level of theory. Energies were determined at a B3PW91/def2TZVP level of theory. Solvation effects were approximated using a PCM field of PhCl.[S15] All structures were evaluated using Molecule 2.3.[S16]

## 1.6.1. Calculated structures of the simple and auto-induced cycle

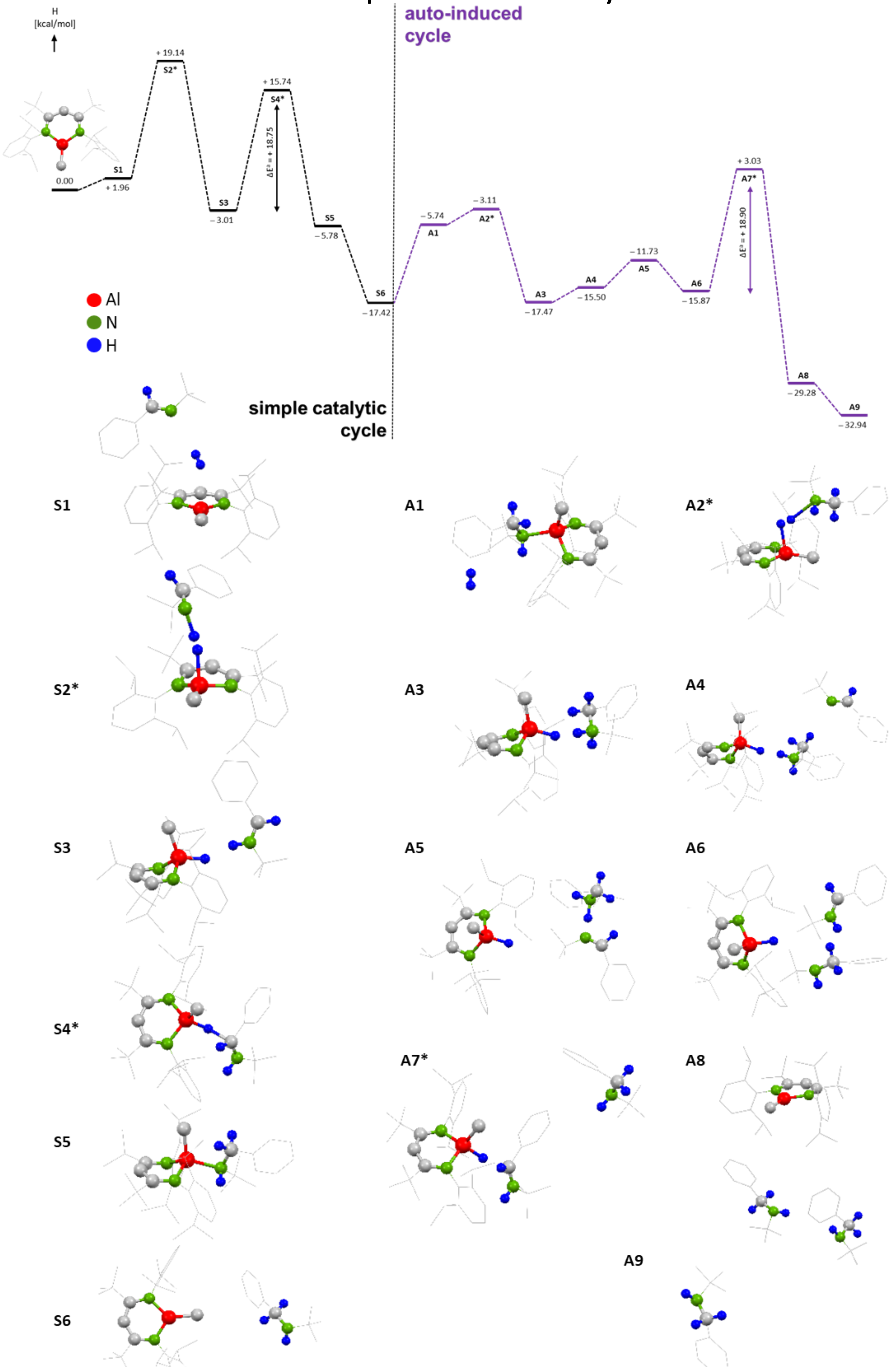


**Figure S83:** Calculated energy profile and structures of the intermediates for catalytic hydrogenation of PhC(H)=NtBu with ($^{tBu,DIPP}$BDI)AlMe$^+$ as catalyst..

## 1.6.2. X-Y-Z files

2

H2

| | | | |
|---|---|---|---|
| H | 0.000000 | -0.000000 | 0.379949 |
| H | 0.000000 | -0.000000 | -0.379949 |

27

PhC(H)=NtBu

| | | | |
|---|---|---|---|
| N | -1.121304 | -0.346760 | -0.000078 |
| C | -0.228107 | 0.555142 | -0.000216 |
| H | -0.461190 | 1.637685 | -0.000488 |
| C | 1.213595 | 0.238685 | -0.000277 |
| C | 1.666623 | -1.090837 | -0.000239 |
| C | 3.029746 | -1.368224 | -0.000035 |
| C | 3.961509 | -0.323897 | 0.000173 |
| C | 2.154152 | 1.278596 | -0.000095 |
| C | 3.521441 | 1.000249 | 0.000164 |
| H | 0.920018 | -1.888111 | -0.000202 |
| H | 3.374341 | -2.405848 | 0.000069 |
| H | 5.032161 | -0.545082 | 0.000286 |
| H | 1.807932 | 2.316893 | -0.000062 |
| H | 4.245514 | 1.819117 | 0.000306 |
| C | -2.550705 | -0.048303 | 0.000036 |
| C | -3.124712 | -0.724153 | 1.255788 |
| C | -2.917660 | 1.440767 | -0.000724 |
| C | -3.125375 | -0.725580 | -1.254688 |
| H | -4.222032 | -0.634711 | 1.281412 |
| H | -2.857019 | -1.790805 | 1.271140 |
| H | -2.718775 | -0.259750 | 2.167987 |
| H | -4.011861 | 1.557374 | -0.000405 |
| H | -2.531154 | 1.958806 | 0.891466 |
| H | -2.531767 | 1.957739 | -0.893801 |
| H | -4.222693 | -0.636173 | -1.280007 |
| H | -2.719783 | -0.262254 | -2.167604 |
| H | -2.857602 | -1.792230 | -1.268863 |

29

PhC(H)2-N(H)tBu

| | | | |
|---|---|---|---|
| N | -1.337966 | 0.112300 | -0.957014 |
| C | -0.268596 | -0.865413 | -1.012140 |
| C | 1.067732 | -0.381852 | -0.474902 |
| C | 1.984677 | -1.288565 | 0.071126 |
| C | 3.239849 | -0.862156 | 0.509056 |
| C | 3.595544 | 0.483845 | 0.409191 |
| C | 1.433506 | 0.966062 | -0.568557 |
| C | 2.686344 | 1.396403 | -0.130669 |
| H | 1.711389 | -2.345355 | 0.158352 |
| H | 3.941111 | -1.584333 | 0.936174 |

| | | | |
|---|---|---|---|
| H | 4.576273 | 0.821238 | 0.754865 |
| H | 0.707210 | 1.670718 | -0.981011 |
| H | 2.955791 | 2.453425 | -0.210089 |
| C | -2.264202 | 0.108127 | 0.186614 |
| C | -3.194052 | 1.311027 | -0.007982 |
| C | -3.108446 | -1.179704 | 0.248360 |
| C | -1.483185 | 0.276480 | 1.493909 |
| H | -3.926278 | 1.384574 | 0.810763 |
| H | -2.612585 | 2.244160 | -0.045824 |
| H | -3.759119 | 1.225019 | -0.951518 |
| H | -3.813889 | -1.156213 | 1.094076 |
| H | -3.696815 | -1.306564 | -0.675508 |
| H | -2.478599 | -2.074465 | 0.374128 |
| H | -2.177752 | 0.336318 | 2.346078 |
| H | -0.804088 | -0.569673 | 1.678573 |
| H | -0.876257 | 1.193231 | 1.472605 |
| H | -0.521764 | -1.824559 | -0.515830 |
| H | -0.115730 | -1.129163 | -2.073940 |
| H | -1.878163 | 0.090014 | -1.816842 |

95

tBuBDI Al Me (+)

| | | | |
|---|---|---|---|
| Al | 0.002419 | 0.015466 | -0.899794 |
| N | 1.461052 | -0.050883 | 0.243795 |
| N | -1.462233 | 0.036914 | 0.238627 |
| C | -1.294728 | 0.047420 | 1.586540 |
| C | -2.707379 | -0.111483 | -0.499079 |
| C | 1.291303 | -0.094363 | 1.590401 |
| C | 2.698171 | 0.120858 | -0.502962 |
| C | 2.472060 | -0.222090 | 2.596726 |
| C | -0.002805 | -0.030399 | 2.145413 |
| C | -2.478860 | 0.153034 | 2.591856 |
| C | -2.001058 | 0.334518 | 4.045178 |
| C | -3.348943 | 1.376490 | 2.260145 |
| C | -3.355641 | -1.114938 | 2.570415 |
| C | 1.990303 | -0.445306 | 4.042697 |
| C | 3.350808 | -1.429053 | 2.233109 |
| C | 3.345212 | 1.048056 | 2.610644 |
| H | -0.005091 | -0.043641 | 3.222585 |
| C | -0.004156 | 0.062060 | -2.834792 |
| H | -1.021349 | -0.096485 | -3.223924 |
| H | 0.364967 | 1.026580 | -3.215089 |
| H | 0.650451 | -0.711684 | -3.260751 |
| C | 3.140335 | 1.450768 | -0.740653 |
| C | 4.244219 | 1.641216 | -1.578415 |
| C | 4.888338 | 0.566986 | -2.183475 |
| C | 3.329840 | -0.984931 | -1.129468 |
| C | 4.429820 | -0.724268 | -1.958873 |
| C | 2.433182 | 2.670194 | -0.152392 |
| H | 4.606392 | 2.653477 | -1.766151 |
| H | 5.747964 | 0.738996 | -2.835365 |
| C | 2.887015 | -2.438867 | -0.972269 |

| | | | |
|---|---|---|---|
| H | 4.938604 | -1.560535 | -2.443366 |
| C | -3.341990 | 1.011826 | -1.087100 |
| C | -4.475493 | 0.781991 | -1.879701 |
| C | -4.959796 | -0.499150 | -2.109105 |
| C | -3.165019 | -1.431129 | -0.757840 |
| C | -4.301456 | -1.593455 | -1.556857 |
| C | -2.847386 | 2.448911 | -0.945489 |
| H | -4.988607 | 1.633556 | -2.331814 |
| H | -5.847045 | -0.648130 | -2.728885 |
| C | -2.425041 | -2.664624 | -0.248179 |
| H | -4.677674 | -2.598142 | -1.757019 |
| H | 2.874333 | -0.587295 | 4.680673 |
| H | 1.442551 | 0.416157 | 4.452051 |
| H | 1.367891 | -1.346407 | 4.147050 |
| H | 4.110437 | -1.567744 | 3.016807 |
| H | 2.764786 | -2.358294 | 2.171842 |
| H | 3.882277 | -1.278041 | 1.288789 |
| H | 4.114357 | 0.935000 | 3.389729 |
| H | 3.866347 | 1.209460 | 1.659882 |
| H | 2.757872 | 1.945212 | 2.856737 |
| H | -2.886822 | 0.461629 | 4.683835 |
| H | -1.457562 | -0.539895 | 4.432175 |
| H | -1.376296 | 1.230413 | 4.176795 |
| H | -4.124214 | -1.022043 | 3.352716 |
| H | -3.877198 | -1.250467 | 1.615985 |
| H | -2.769280 | -2.019206 | 2.791846 |
| H | -4.107515 | 1.499646 | 3.047472 |
| H | -2.754361 | 2.301794 | 2.225826 |
| H | -3.881472 | 1.258403 | 1.311581 |
| C | 3.389224 | 3.799914 | 0.244619 |
| C | 1.361312 | 3.208996 | -1.106108 |
| H | 1.914714 | 2.350981 | 0.763145 |
| H | 0.866347 | 4.095357 | -0.680530 |
| H | 1.790275 | 3.489696 | -2.080541 |
| H | 0.565921 | 2.471544 | -1.312289 |
| H | 2.844829 | 4.573338 | 0.807543 |
| H | 4.214230 | 3.440626 | 0.876646 |
| H | 3.830572 | 4.296697 | -0.632943 |
| C | 4.031422 | -3.366417 | -0.532836 |
| H | 2.107146 | -2.472689 | -0.194006 |
| C | 2.286447 | -2.995293 | -2.271946 |
| H | 3.636701 | -4.367487 | -0.300619 |
| H | 4.773041 | -3.493356 | -1.336288 |
| H | 4.565226 | -3.001154 | 0.354037 |
| H | 1.963639 | -4.038117 | -2.130015 |
| H | 1.416957 | -2.422483 | -2.619244 |
| H | 3.028325 | -2.987666 | -3.085470 |
| C | -3.344245 | -3.832397 | 0.121618 |
| C | -1.379027 | -3.125975 | -1.269099 |
| H | -1.881853 | -2.380912 | 0.665149 |
| C | -3.941155 | 3.426326 | -0.490226 |
| H | -2.051336 | 2.458541 | -0.182766 |

| | | | |
|---|---|---|---|
| C | -2.249532 | 2.956136 | -2.266961 |
| H | -1.843681 | 3.971927 | -2.143000 |
| H | -1.444039 | 2.311755 | -2.643202 |
| H | -3.018812 | 3.001746 | -3.053539 |
| H | -3.502787 | 4.417081 | -0.294997 |
| H | -4.706373 | 3.563549 | -1.269484 |
| H | -4.454027 | 3.099576 | 0.423877 |
| H | -0.819020 | -3.998093 | -0.898534 |
| H | -1.844905 | -3.397245 | -2.229014 |
| H | -0.635472 | -2.340867 | -1.498709 |
| H | -2.764407 | -4.625715 | 0.617447 |
| H | -4.147475 | -3.523996 | 0.806457 |
| H | -3.813458 | -4.287603 | -0.763970 |

97

tBuBDI Al Me (+) + H2

| | | | |
|---|---|---|---|
| Al | -0.002060 | -0.076025 | -0.899828 |
| N | -1.466661 | -0.082269 | 0.238779 |
| N | 1.457184 | -0.015594 | 0.243311 |
| C | 1.287909 | 0.035289 | 1.589701 |
| C | 2.692799 | -0.200165 | -0.502830 |
| C | -1.299074 | -0.088076 | 1.586704 |
| C | -2.710764 | 0.071813 | -0.499474 |
| C | -2.483848 | -0.181299 | 2.592514 |
| C | -0.006575 | -0.016797 | 2.145120 |
| C | 2.469678 | 0.158915 | 2.595362 |
| C | 1.989698 | 0.393967 | 4.040051 |
| C | 3.332246 | -1.118340 | 2.616204 |
| C | 3.358451 | 1.356547 | 2.225375 |
| C | -2.007237 | -0.360445 | 4.046514 |
| C | -3.351671 | 1.092724 | 2.566198 |
| C | -3.362570 | -1.399851 | 2.265585 |
| H | -0.008632 | 0.001385 | 3.222220 |
| C | -0.008690 | -0.131812 | -2.834575 |
| H | 0.652218 | 0.634586 | -3.264060 |
| H | 0.352801 | -1.101100 | -3.210048 |
| H | -1.024494 | 0.032915 | -3.224776 |
| C | -3.353160 | -1.049390 | -1.083029 |
| C | -4.485099 | -0.814830 | -1.876491 |
| C | -4.960505 | 0.468723 | -2.110933 |
| C | -3.159170 | 1.393581 | -0.763518 |
| C | -4.294507 | 1.560627 | -1.563111 |
| C | -2.868561 | -2.489290 | -0.935677 |
| H | -5.004139 | -1.664596 | -2.325213 |
| H | -5.846753 | 0.621406 | -2.731245 |
| C | -2.410375 | 2.623805 | -0.258870 |
| H | -4.663744 | 2.567095 | -1.767262 |
| C | 3.124832 | -1.534474 | -0.734295 |
| C | 4.227006 | -1.737195 | -1.571437 |
| C | 4.878950 | -0.670723 | -2.181827 |
| C | 3.332525 | 0.897866 | -1.134775 |
| C | 4.430220 | 0.625005 | -1.963276 |

| | | | |
|---|---|---|---|
| C | 2.408594 | -2.745691 | -0.140109 |
| H | 4.581518 | -2.753026 | -1.754469 |
| H | 5.737040 | -0.852300 | -2.833145 |
| C | 2.900896 | 2.355822 | -0.983950 |
| H | 4.945140 | 1.455126 | -2.451839 |
| H | 0.501977 | 8.596698 | -0.336014 |
| H | 0.757585 | 8.545762 | 0.377790 |
| C | 4.052706 | 3.276472 | -0.549327 |
| C | 2.303791 | 2.911121 | -2.285700 |
| H | 2.121753 | 2.399145 | -0.205424 |
| H | -4.120710 | 1.008330 | 3.349003 |
| H | -2.758878 | 1.993692 | 2.783941 |
| H | -3.872447 | 1.228180 | 1.611334 |
| H | -4.121781 | -1.514786 | 3.053541 |
| H | -3.894514 | -1.281535 | 1.316719 |
| H | -2.774499 | -2.329417 | 2.234541 |
| H | -2.893825 | -0.478775 | 4.685715 |
| H | -1.388798 | -1.260197 | 4.181625 |
| H | -1.457564 | 0.511639 | 4.430027 |
| H | 2.874810 | 0.532852 | 4.677210 |
| H | 1.374120 | 1.300332 | 4.139253 |
| H | 1.435455 | -0.460968 | 4.454316 |
| H | 4.119150 | 1.493091 | 3.008410 |
| H | 3.888745 | 1.196117 | 1.281940 |
| H | 2.780187 | 2.290284 | 2.159087 |
| H | 4.102073 | -1.007642 | 3.394954 |
| H | 2.737379 | -2.009300 | 2.866711 |
| H | 3.852293 | -1.288964 | 1.666452 |
| C | 3.356223 | -3.880395 | 0.262823 |
| H | 1.892301 | -2.418058 | 0.773681 |
| C | 1.332955 | -3.281403 | -1.091321 |
| H | 0.831575 | -4.162058 | -0.661424 |
| H | 0.542907 | -2.539272 | -1.301169 |
| H | 1.759975 | -3.570026 | -2.064289 |
| H | 2.806062 | -4.646893 | 0.829598 |
| H | 3.793999 | -4.384895 | -0.612124 |
| H | 4.183791 | -3.523997 | 0.893138 |
| H | 3.665760 | 4.281424 | -0.320872 |
| H | 4.584545 | 2.910743 | 0.338536 |
| H | 4.794516 | 3.394461 | -1.353967 |
| H | 1.989877 | 3.957244 | -2.148239 |
| H | 3.044822 | 2.893515 | -3.099838 |
| H | 1.429194 | 2.344001 | -2.629505 |
| C | -3.968933 | -3.457128 | -0.475895 |
| C | -2.274939 | -3.006212 | -2.255304 |
| H | -2.072212 | -2.501315 | -0.173286 |
| C | -3.321009 | 3.800245 | 0.104529 |
| H | -1.870290 | 2.340255 | 0.656341 |
| C | -1.359822 | 3.072377 | -1.280789 |
| H | -1.876162 | -4.024297 | -2.127269 |
| H | -3.044932 | -3.049746 | -3.041302 |
| H | -1.465144 | -2.369095 | -2.634649 |

| | | | |
|---|---|---|---|
| H | -3.537436 | -4.450129 | -0.276813 |
| H | -4.479049 | -3.123048 | 0.437088 |
| H | -4.735471 | -3.592144 | -1.254241 |
| H | -0.793738 | 3.942090 | -0.913863 |
| H | -0.621776 | 2.280655 | -1.505592 |
| H | -1.822518 | 3.342243 | -2.242636 |
| H | -2.735624 | 4.591503 | 0.597095 |
| H | -3.785990 | 4.254766 | -0.783641 |
| H | -4.127154 | 3.501137 | 0.790077 |

122
tBuBDI Al Me (+) + PhC(H)=NtBu

| | | | |
|---|---|---|---|
| Al | 1.645590 | -0.067686 | -0.952283 |
| N | 1.893375 | -1.286128 | 0.424574 |
| N | 1.354269 | 1.544455 | -0.080581 |
| C | 1.300638 | 1.601264 | 1.275105 |
| C | 1.337447 | 2.658431 | -1.016608 |
| C | 1.832274 | -0.893903 | 1.723094 |
| C | 1.985788 | -2.638709 | -0.104139 |
| C | 2.085071 | -1.858029 | 2.919395 |
| C | 1.538877 | 0.445862 | 2.047453 |
| C | 0.975608 | 2.905037 | 2.061570 |
| C | 0.807566 | 2.653702 | 3.572122 |
| C | 2.099055 | 3.949744 | 1.911366 |
| C | -0.349041 | 3.509967 | 1.567637 |
| C | 2.122333 | -1.121154 | 4.271487 |
| C | 0.983424 | -2.930254 | 3.029904 |
| C | 3.445870 | -2.553730 | 2.758552 |
| H | 1.491678 | 0.626254 | 3.108268 |
| C | 1.717158 | -0.382373 | -2.860729 |
| H | 0.847629 | -0.963806 | -3.202527 |
| H | 1.733522 | 0.570497 | -3.411177 |
| H | 2.609623 | -0.958627 | -3.143993 |
| C | 3.223589 | -3.154284 | -0.569863 |
| C | 3.213523 | -4.405576 | -1.202081 |
| C | 2.038657 | -5.121006 | -1.390853 |
| C | 0.771011 | -3.346882 | -0.310742 |
| C | 0.831240 | -4.590106 | -0.949451 |
| C | 4.565137 | -2.433516 | -0.446068 |
| H | 4.155862 | -4.827271 | -1.558637 |
| H | 2.061552 | -6.093707 | -1.887731 |
| C | -0.589891 | -2.785965 | 0.097633 |
| H | -0.088579 | -5.155102 | -1.109122 |
| C | 2.576811 | 3.277036 | -1.331110 |
| C | 2.588599 | 4.275762 | -2.310391 |
| C | 1.427247 | 4.644697 | -2.981883 |
| C | 0.152385 | 2.994304 | -1.718797 |
| C | 0.231106 | 4.002315 | -2.690075 |
| C | 3.891983 | 2.847181 | -0.687593 |
| H | 3.528198 | 4.772718 | -2.558012 |
| H | 1.458650 | 5.428674 | -3.742154 |
| C | -1.191363 | 2.301445 | -1.512361 |

| | | | |
|---|---|---|---|
| H | -0.672605 | 4.286356 | -3.233605 |
| C | -2.350413 | 3.279723 | -1.271567 |
| C | -1.538374 | 1.408779 | -2.713588 |
| H | -1.108962 | 1.658076 | -0.621189 |
| H | 1.172824 | -3.534339 | 3.930209 |
| H | -0.014807 | -2.481080 | 3.139867 |
| H | 0.975903 | -3.615088 | 2.174179 |
| H | 3.657975 | -3.139949 | 3.665169 |
| H | 3.451718 | -3.248451 | 1.913340 |
| H | 4.263901 | -1.828465 | 2.633887 |
| H | 2.357253 | -1.855108 | 5.055621 |
| H | 2.902055 | -0.346090 | 4.310245 |
| H | 1.156638 | -0.669480 | 4.541965 |
| H | 0.526240 | 3.603972 | 4.048036 |
| H | 0.007808 | 1.931843 | 3.794190 |
| H | 1.735536 | 2.320088 | 4.059373 |
| H | -0.612217 | 4.363403 | 2.210317 |
| H | -0.273143 | 3.887085 | 0.543226 |
| H | -1.175593 | 2.786143 | 1.624300 |
| H | 1.861978 | 4.812988 | 2.551639 |
| H | 3.071273 | 3.553419 | 2.240545 |
| H | 2.192308 | 4.321761 | 0.884762 |
| C | 4.870017 | 4.000377 | -0.443427 |
| H | 3.661490 | 2.406116 | 0.293341 |
| C | 4.569389 | 1.756443 | -1.524744 |
| H | 5.504161 | 1.416931 | -1.052977 |
| H | 3.930020 | 0.865377 | -1.654755 |
| H | 4.806185 | 2.116493 | -2.537930 |
| H | 5.725960 | 3.648554 | 0.152482 |
| H | 5.280125 | 4.401472 | -1.382892 |
| H | 4.400257 | 4.832790 | 0.100254 |
| H | -3.260770 | 2.722016 | -1.005144 |
| H | -2.145993 | 3.993457 | -0.462675 |
| H | -2.579988 | 3.860692 | -2.178105 |
| H | -2.475612 | 0.862749 | -2.528415 |
| H | -1.677785 | 2.013134 | -3.623662 |
| H | -0.754046 | 0.672698 | -2.935564 |
| C | 5.649173 | -3.297059 | 0.218947 |
| C | 5.088221 | -1.972512 | -1.814791 |
| H | 4.413276 | -1.539729 | 0.180999 |
| C | -1.567548 | -3.857188 | 0.591967 |
| H | -0.431378 | -2.086574 | 0.931147 |
| C | -1.229482 | -1.983923 | -1.041147 |
| H | 6.044017 | -1.438305 | -1.701304 |
| H | 5.267786 | -2.832283 | -2.478713 |
| H | 4.390993 | -1.301791 | -2.333073 |
| H | 6.550098 | -2.692552 | 0.405624 |
| H | 5.329172 | -3.728447 | 1.176156 |
| H | 5.952167 | -4.129647 | -0.434226 |
| H | -2.237940 | -1.636857 | -0.767788 |
| H | -0.646140 | -1.082971 | -1.303805 |
| H | -1.311747 | -2.584731 | -1.960192 |

| | | | |
|---|---|---|---|
| H | -2.462985 | -3.380921 | 1.018506 |
| H | -1.914912 | -4.508572 | -0.224521 |
| H | -1.121783 | -4.498469 | 1.366403 |
| N | -5.417849 | -1.049026 | -0.689758 |
| C | -6.067741 | -0.186408 | -0.019974 |
| H | -7.091923 | 0.132059 | -0.289240 |
| C | -5.530038 | 0.468701 | 1.188761 |
| C | -4.317741 | 0.064437 | 1.771284 |
| C | -3.849544 | 0.684971 | 2.925744 |
| C | -4.580243 | 1.723505 | 3.514640 |
| C | -6.258203 | 1.505654 | 1.790820 |
| C | -5.785290 | 2.133587 | 2.943495 |
| H | -3.770367 | -0.757518 | 1.305533 |
| H | -2.915198 | 0.349859 | 3.384796 |
| H | -4.215251 | 2.204318 | 4.425974 |
| H | -7.209141 | 1.820632 | 1.350676 |
| H | -6.363816 | 2.939191 | 3.402257 |
| C | -5.996618 | -1.716735 | -1.859713 |
| C | -5.944425 | -3.222255 | -1.554131 |
| C | -7.433974 | -1.313333 | -2.209192 |
| C | -5.073454 | -1.400150 | -3.046297 |
| H | -6.282320 | -3.808633 | -2.422460 |
| H | -4.918865 | -3.529199 | -1.301189 |
| H | -6.592870 | -3.469163 | -0.699505 |
| H | -7.774123 | -1.875573 | -3.091310 |
| H | -8.134756 | -1.535608 | -1.389173 |
| H | -7.515067 | -0.242187 | -2.453125 |
| H | -5.397393 | -1.943739 | -3.947056 |
| H | -5.086533 | -0.322968 | -3.274759 |
| H | -4.037330 | -1.691253 | -2.819529 |

124

tBuBDI Al Me (+) + PhC(H)=NtBu + H2

| | | | |
|---|---|---|---|
| Al | 1.600106 | -0.157213 | -0.775435 |
| N | 1.304099 | -1.572381 | 0.386098 |
| N | 2.745799 | 0.961465 | 0.162537 |
| C | 3.119632 | 0.664031 | 1.434499 |
| C | 3.255119 | 2.030255 | -0.682806 |
| C | 1.893066 | -1.609932 | 1.607976 |
| C | 0.266169 | -2.445030 | -0.142311 |
| C | 1.728410 | -2.798370 | 2.599632 |
| C | 2.706516 | -0.541196 | 2.037032 |
| C | 3.998830 | 1.607749 | 2.306811 |
| C | 4.110881 | 1.124280 | 3.765364 |
| C | 5.437885 | 1.706280 | 1.762879 |
| C | 3.378557 | 3.013612 | 2.357330 |
| C | 2.658223 | -2.677084 | 3.821704 |
| C | 0.288604 | -2.886620 | 3.142953 |
| C | 2.082237 | -4.122763 | 1.905604 |
| H | 3.091418 | -0.672892 | 3.034477 |
| C | 0.908516 | 0.094839 | -2.565400 |
| H | -0.171377 | 0.306421 | -2.550244 |

| | | | |
|---|---|---|---|
| H | 1.418850 | 0.931483 | -3.066116 |
| H | 1.041252 | -0.805954 | -3.181509 |
| C | 0.581407 | -3.476028 | -1.065062 |
| C | -0.484131 | -4.173021 | -1.651888 |
| C | -1.807293 | -3.864067 | -1.365309 |
| C | -1.082978 | -2.102942 | 0.146156 |
| C | -2.099769 | -2.835573 | -0.475463 |
| C | 2.000322 | -3.870680 | -1.472008 |
| H | -0.266545 | -4.978671 | -2.356645 |
| H | -2.617153 | -4.422381 | -1.840423 |
| C | -1.456509 | -0.946090 | 1.070698 |
| H | -3.143361 | -2.592824 | -0.268070 |
| C | 4.436225 | 1.773138 | -1.429168 |
| C | 4.879190 | 2.750633 | -2.326126 |
| C | 4.175542 | 3.936931 | -2.509128 |
| C | 2.503989 | 3.215324 | -0.887439 |
| C | 3.000336 | 4.154860 | -1.802203 |
| C | 5.191644 | 0.450867 | -1.330228 |
| H | 5.791770 | 2.578546 | -2.899369 |
| H | 4.540175 | 4.688076 | -3.213677 |
| C | 1.170966 | 3.514931 | -0.206939 |
| H | 2.444823 | 5.080880 | -1.965799 |
| C | 1.131891 | 4.891128 | 0.473922 |
| C | 0.010440 | 3.425938 | -1.210029 |
| H | 1.010252 | 2.751552 | 0.572153 |
| H | 0.246574 | -3.691854 | 3.892125 |
| H | -0.017456 | -1.955768 | 3.643164 |
| H | -0.440537 | -3.129968 | 2.361401 |
| H | 2.065060 | -4.932687 | 2.650078 |
| H | 1.358765 | -4.381670 | 1.126812 |
| H | 3.090714 | -4.098192 | 1.466265 |
| H | 2.532317 | -3.577935 | 4.439258 |
| H | 3.720653 | -2.622579 | 3.541314 |
| H | 2.414539 | -1.818717 | 4.464791 |
| H | 4.690713 | 1.868674 | 4.329607 |
| H | 3.131388 | 1.035568 | 4.258331 |
| H | 4.647165 | 0.168905 | 3.862405 |
| H | 3.947347 | 3.630253 | 3.069206 |
| H | 3.419178 | 3.516912 | 1.386574 |
| H | 2.334536 | 2.985617 | 2.703908 |
| H | 6.034223 | 2.315941 | 2.458636 |
| H | 5.917066 | 0.717961 | 1.698397 |
| H | 5.484683 | 2.192116 | 0.781532 |
| C | 6.710081 | 0.589941 | -1.473509 |
| H | 4.999842 | 0.024207 | -0.334800 |
| C | 4.659512 | -0.551709 | -2.360415 |
| H | 5.177064 | -1.519603 | -2.277278 |
| H | 3.579857 | -0.752156 | -2.237616 |
| H | 4.788171 | -0.178981 | -3.388355 |
| H | 7.199291 | -0.368915 | -1.243902 |
| H | 7.006788 | 0.860810 | -2.498319 |
| H | 7.121134 | 1.349462 | -0.792740 |

| | | | |
|---|---|---|---|
| H | 0.194918 | 5.003538 | 1.040758 |
| H | 1.966805 | 5.048931 | 1.169248 |
| H | 1.157292 | 5.705630 | -0.266079 |
| H | -0.953072 | 3.585684 | -0.702374 |
| H | 0.105580 | 4.198294 | -1.989153 |
| H | -0.033732 | 2.456277 | -1.723133 |
| C | 2.274417 | -5.373101 | -1.296638 |
| C | 2.302235 | -3.491481 | -2.929459 |
| H | 2.703063 | -3.321370 | -0.824479 |
| C | -2.704126 | -1.219809 | 1.916888 |
| H | -0.621324 | -0.788598 | 1.768336 |
| C | -1.636466 | 0.359491 | 0.289028 |
| H | 3.332732 | -3.774053 | -3.194346 |
| H | 1.627422 | -4.017002 | -3.622829 |
| H | 2.192660 | -2.416300 | -3.120942 |
| H | 3.341047 | -5.584320 | -1.468879 |
| H | 2.015055 | -5.743968 | -0.296523 |
| H | 1.707796 | -5.969904 | -2.027720 |
| H | -1.910328 | 1.186033 | 0.962155 |
| H | -0.715503 | 0.672609 | -0.235369 |
| H | -2.422007 | 0.269486 | -0.476600 |
| H | -2.826115 | -0.428226 | 2.671968 |
| H | -3.622322 | -1.227414 | 1.310612 |
| H | -2.640611 | -2.182539 | 2.444775 |
| N | -6.247430 | 2.022717 | -0.128660 |
| C | -6.734642 | 0.981758 | 0.411691 |
| H | -7.305206 | 1.006118 | 1.359533 |
| C | -6.594132 | -0.364001 | -0.180120 |
| C | -5.992532 | -0.554569 | -1.435367 |
| C | -5.892726 | -1.831172 | -1.981262 |
| C | -6.387835 | -2.938121 | -1.281261 |
| C | -7.089068 | -1.479236 | 0.512485 |
| C | -6.984392 | -2.759954 | -0.032000 |
| H | -5.626525 | 0.323215 | -1.971825 |
| H | -5.439503 | -1.967569 | -2.966911 |
| H | -6.321394 | -3.938211 | -1.717965 |
| H | -7.568955 | -1.338552 | 1.485875 |
| H | -7.380400 | -3.619486 | 0.514703 |
| C | -6.419101 | 3.357330 | 0.446706 |
| C | -7.160476 | 4.185601 | -0.615272 |
| C | -7.186126 | 3.413769 | 1.773167 |
| C | -5.004214 | 3.924130 | 0.644076 |
| H | -7.253194 | 5.234993 | -0.295367 |
| H | -6.622318 | 4.157255 | -1.573839 |
| H | -8.172510 | 3.786418 | -0.782910 |
| H | -7.254405 | 4.455128 | 2.121166 |
| H | -8.215444 | 3.036089 | 1.669526 |
| H | -6.684008 | 2.834549 | 2.564672 |
| H | -5.046926 | 4.970404 | 0.984113 |
| H | -4.453482 | 3.340190 | 1.398715 |
| H | -4.440659 | 3.884919 | -0.299857 |
| H | -4.536637 | 2.611428 | -3.030761 |

| | | | |
|---|---|---|---|
| H | -4.151566 | 2.684229 | -3.681769 |

124
TS1

| | | | |
|---|---|---|---|
| Al | 0.600477 | 0.115493 | -1.043318 |
| N | 1.322656 | -1.297290 | -0.001989 |
| N | 0.556692 | 1.589265 | 0.114223 |
| C | 0.190400 | 1.224599 | 1.380655 |
| C | 0.904235 | 2.951242 | -0.250485 |
| C | 1.155419 | -1.182633 | 1.331303 |
| C | 1.915368 | -2.422066 | -0.716443 |
| C | 1.703716 | -2.200603 | 2.372710 |
| C | 0.452005 | -0.068586 | 1.861490 |
| C | -0.556054 | 2.179044 | 2.362100 |
| C | -1.306630 | 1.389247 | 3.454228 |
| C | 0.403138 | 3.138422 | 3.089516 |
| C | -1.617200 | 2.991202 | 1.605620 |
| C | 1.404212 | -1.780324 | 3.824202 |
| C | 1.109948 | -3.606878 | 2.186694 |
| C | 3.235777 | -2.272378 | 2.243604 |
| H | 0.138328 | -0.212104 | 2.884071 |
| C | 0.844791 | 0.092247 | -2.965761 |
| H | -0.099663 | 0.236849 | -3.510187 |
| H | 1.537775 | 0.881616 | -3.296218 |
| H | 1.268004 | -0.875611 | -3.277084 |
| C | 3.274802 | -2.395544 | -1.125048 |
| C | 3.779181 | -3.493387 | -1.836337 |
| C | 2.983947 | -4.579449 | -2.172118 |
| C | 1.077191 | -3.501801 | -1.109090 |
| C | 1.640266 | -4.567277 | -1.820479 |
| C | 4.221729 | -1.222967 | -0.901468 |
| H | 4.827930 | -3.488081 | -2.140400 |
| H | 3.404601 | -5.424405 | -2.722212 |
| C | -0.426808 | -3.532186 | -0.869509 |
| H | 1.005032 | -5.405992 | -2.112130 |
| C | 2.088867 | 3.519738 | 0.300657 |
| C | 2.396690 | 4.852991 | 0.008153 |
| C | 1.600529 | 5.617401 | -0.835311 |
| C | 0.134147 | 3.698788 | -1.180016 |
| C | 0.499508 | 5.027481 | -1.438300 |
| C | 3.123795 | 2.712712 | 1.077344 |
| H | 3.294197 | 5.298090 | 0.441490 |
| H | 1.853959 | 6.659533 | -1.043173 |
| C | -1.049425 | 3.155601 | -1.972373 |
| H | -0.101362 | 5.615379 | -2.135596 |
| C | -2.354780 | 3.927757 | -1.732543 |
| C | -0.726794 | 3.172948 | -3.475815 |
| H | -1.222694 | 2.110887 | -1.659728 |
| H | 1.467542 | -4.254660 | 3.001574 |
| H | 0.011509 | -3.589305 | 2.236146 |
| H | 1.413838 | -4.070100 | 1.241890 |
| H | 3.635795 | -2.892333 | 3.060181 |

| | | | |
|---|---|---|---|
| H | 3.544593 | -2.732533 | 1.299858 |
| H | 3.697478 | -1.276679 | 2.324116 |
| H | 1.868351 | -2.515907 | 4.497175 |
| H | 1.825721 | -0.796479 | 4.076406 |
| H | 0.328308 | -1.773961 | 4.054946 |
| H | -1.932354 | 2.095923 | 4.018598 |
| H | -1.969482 | 0.617669 | 3.037530 |
| H | -0.634504 | 0.920675 | 4.188069 |
| H | -2.213950 | 3.568134 | 2.327830 |
| H | -1.171981 | 3.706445 | 0.906816 |
| H | -2.305360 | 2.332914 | 1.053817 |
| H | -0.163726 | 3.703032 | 3.846122 |
| H | 1.198924 | 2.588453 | 3.613247 |
| H | 0.861584 | 3.864362 | 2.407955 |
| C | 3.796641 | 3.461942 | 2.233344 |
| H | 2.635004 | 1.821536 | 1.498209 |
| C | 4.196063 | 2.238983 | 0.087013 |
| H | 4.925093 | 1.575445 | 0.577825 |
| H | 3.753571 | 1.702458 | -0.762827 |
| H | 4.748179 | 3.098642 | -0.324505 |
| H | 4.425509 | 2.767519 | 2.811717 |
| H | 4.462567 | 4.260687 | 1.872350 |
| H | 3.074744 | 3.916523 | 2.924957 |
| H | -3.163610 | 3.505356 | -2.348445 |
| H | -2.678456 | 3.902604 | -0.684506 |
| H | -2.251037 | 4.983957 | -2.024304 |
| H | -1.509082 | 2.657299 | -4.053953 |
| H | -0.678175 | 4.206431 | -3.851382 |
| H | 0.236691 | 2.698628 | -3.702465 |
| C | 5.575894 | -1.628761 | -0.301958 |
| C | 4.465336 | -0.483320 | -2.226731 |
| H | 3.738526 | -0.526635 | -0.199287 |
| C | -0.956321 | -4.880267 | -0.363381 |
| H | -0.662936 | -2.772060 | -0.108784 |
| C | -1.160387 | -3.153113 | -2.163597 |
| H | 5.161825 | 0.356403 | -2.083488 |
| H | 4.913226 | -1.158316 | -2.972605 |
| H | 3.538403 | -0.086932 | -2.662751 |
| H | 6.160641 | -0.730073 | -0.052271 |
| H | 5.477329 | -2.230135 | 0.611694 |
| H | 6.178153 | -2.208801 | -1.017914 |
| H | -2.244778 | -3.081605 | -1.990652 |
| H | -0.813879 | -2.192882 | -2.571242 |
| H | -0.990950 | -3.913311 | -2.942144 |
| H | -2.019542 | -4.790382 | -0.091055 |
| H | -0.895315 | -5.658825 | -1.138925 |
| H | -0.411683 | -5.246428 | 0.517279 |
| N | -3.825185 | -0.509489 | -1.092057 |
| C | -4.593695 | -0.415070 | -0.074911 |
| H | -5.623624 | -0.037262 | -0.193949 |
| C | -4.269845 | -0.819343 | 1.298072 |
| C | -3.258713 | -1.749489 | 1.592265 |

| | | | |
|---|---|---|---|
| C | -3.061015 | -2.178097 | 2.902288 |
| C | -3.856024 | -1.675500 | 3.936899 |
| C | -5.074327 | -0.333229 | 2.343998 |
| C | -4.860450 | -0.746882 | 3.656735 |
| H | -2.661839 | -2.163684 | 0.778485 |
| H | -2.295491 | -2.926167 | 3.120856 |
| H | -3.702885 | -2.022163 | 4.961919 |
| H | -5.879833 | 0.372523 | 2.121401 |
| H | -5.490382 | -0.359570 | 4.460843 |
| C | -4.384306 | -0.253899 | -2.447110 |
| C | -5.095330 | -1.541558 | -2.895016 |
| C | -5.368672 | 0.924116 | -2.480034 |
| C | -3.215552 | 0.037085 | -3.388940 |
| H | -5.469983 | -1.430079 | -3.923690 |
| H | -4.410531 | -2.402034 | -2.873677 |
| H | -5.951920 | -1.769225 | -2.242202 |
| H | -5.650925 | 1.136629 | -3.521383 |
| H | -6.303159 | 0.718181 | -1.937649 |
| H | -4.915683 | 1.834429 | -2.058984 |
| H | -3.578829 | 0.188654 | -4.415700 |
| H | -2.679142 | 0.946440 | -3.084017 |
| H | -2.504094 | -0.800162 | -3.403664 |
| H | -2.130981 | -0.444217 | -0.895165 |
| H | -1.302053 | -0.343646 | -0.759879 |

124
Intermediate

| | | | |
|---|---|---|---|
| Al | -0.014107 | -0.147005 | -0.644702 |
| N | -0.889177 | 1.589282 | -0.459459 |
| N | -1.529140 | -1.305718 | -0.360497 |
| C | -2.489963 | -0.920115 | -1.241072 |
| C | -1.636990 | -2.414143 | 0.541569 |
| C | -2.060613 | 1.631764 | -1.099905 |
| C | -0.198701 | 2.681143 | 0.178360 |
| C | -2.914024 | 2.897517 | -1.409732 |
| C | -2.626165 | 0.427218 | -1.616064 |
| C | -3.392243 | -1.904488 | -2.050696 |
| C | -4.852683 | -1.420145 | -2.138795 |
| C | -2.789396 | -1.920757 | -3.477833 |
| C | -3.405981 | -3.360800 | -1.568320 |
| C | -2.964263 | 3.076225 | -2.945099 |
| C | -4.343844 | 2.668500 | -0.871821 |
| C | -2.428692 | 4.228967 | -0.819150 |
| H | -3.402002 | 0.595877 | -2.354420 |
| C | 0.635755 | -0.356290 | -2.515542 |
| H | 0.815299 | -1.409604 | -2.791629 |
| H | -0.123286 | 0.016727 | -3.223199 |
| H | 1.562993 | 0.195522 | -2.734944 |
| C | 0.787203 | 3.407551 | -0.540757 |
| C | 1.468189 | 4.443685 | 0.110510 |
| C | 1.210806 | 4.766533 | 1.437481 |
| C | -0.438650 | 2.974701 | 1.544007 |

| | | | |
|---|---|---|---|
| C | 0.269815 | 4.025480 | 2.141593 |
| C | 1.146957 | 3.130656 | -1.994833 |
| H | 2.211557 | 5.021458 | -0.444579 |
| H | 1.741170 | 5.591120 | 1.920233 |
| C | -1.426919 | 2.205512 | 2.408876 |
| H | 0.070604 | 4.272428 | 3.187441 |
| C | -0.633077 | -3.421218 | 0.555828 |
| C | -0.727273 | -4.470687 | 1.476838 |
| C | -1.774794 | -4.554616 | 2.384560 |
| C | -2.706871 | -2.487497 | 1.480099 |
| C | -2.746508 | -3.563008 | 2.376566 |
| C | 0.531184 | -3.463292 | -0.424977 |
| H | 0.035057 | -5.253806 | 1.471100 |
| H | -1.834373 | -5.385852 | 3.091078 |
| C | -3.816275 | -1.447953 | 1.614258 |
| H | -3.568434 | -3.621282 | 3.094111 |
| H | 1.150305 | -0.257833 | 0.499389 |
| H | 2.981479 | -0.308289 | 0.907317 |
| N | 3.885558 | -0.301406 | 1.415438 |
| C | 4.976745 | -0.634282 | 0.807274 |
| H | 5.885795 | -0.608156 | 1.415733 |
| C | 5.142403 | -1.030039 | -0.569164 |
| C | 6.451771 | -1.356276 | -0.984178 |
| C | 6.697044 | -1.743223 | -2.296352 |
| C | 5.638991 | -1.805372 | -3.206544 |
| C | 4.082395 | -1.097661 | -1.497313 |
| C | 4.336075 | -1.482154 | -2.806749 |
| H | 7.276876 | -1.303993 | -0.268499 |
| H | 7.711372 | -1.995406 | -2.612355 |
| H | 5.828920 | -2.107395 | -4.239591 |
| H | 3.059662 | -0.851143 | -1.204031 |
| H | 3.513550 | -1.528529 | -3.523164 |
| C | 3.749031 | 0.128440 | 2.839521 |
| C | 2.756568 | -0.840884 | 3.493191 |
| C | 5.091392 | 0.089502 | 3.561347 |
| C | 3.194189 | 1.557413 | 2.803046 |
| H | 2.555305 | -0.519129 | 4.524855 |
| H | 1.799142 | -0.861848 | 2.952800 |
| H | 3.161628 | -1.863287 | 3.524849 |
| H | 4.931911 | 0.406101 | 4.601359 |
| H | 5.522204 | -0.923366 | 3.595720 |
| H | 5.825815 | 0.783917 | 3.124417 |
| H | 3.002565 | 1.902083 | 3.829041 |
| H | 3.911503 | 2.248918 | 2.336763 |
| H | 2.246919 | 1.614931 | 2.247557 |
| C | -5.225252 | -2.052157 | 1.522311 |
| C | -3.683525 | -0.686387 | 2.938127 |
| H | -3.710506 | -0.715637 | 0.800405 |
| H | -3.378531 | -2.598489 | -4.115682 |
| H | -2.795692 | -0.925776 | -3.945126 |
| H | -1.751068 | -2.284369 | -3.468229 |
| H | -3.971308 | -3.959724 | -2.298892 |

| | | | |
|---|---|---|---|
| H | -2.399859 | -3.789050 | -1.501807 |
| H | -3.885954 | -3.490611 | -0.592170 |
| H | -5.461042 | -2.188320 | -2.640135 |
| H | -5.288615 | -1.244957 | -1.145816 |
| H | -4.966200 | -0.497841 | -2.725821 |
| H | -3.534032 | 3.987274 | -3.184498 |
| H | -1.956118 | 3.191042 | -3.370542 |
| H | -3.455027 | 2.239981 | -3.461633 |
| H | -4.962346 | 3.553553 | -1.087373 |
| H | -4.836514 | 1.797671 | -1.323664 |
| H | -4.338607 | 2.522462 | 0.219211 |
| H | -3.147698 | 5.009755 | -1.111346 |
| H | -2.384661 | 4.219065 | 0.275721 |
| H | -1.445206 | 4.535063 | -1.190802 |
| H | -5.983190 | -1.252945 | 1.520702 |
| H | -5.372180 | -2.656039 | 0.616528 |
| H | -5.444082 | -2.699248 | 2.385939 |
| H | -4.420852 | 0.130069 | 2.995345 |
| H | -3.860506 | -1.351431 | 3.798347 |
| H | -2.681240 | -0.258069 | 3.054766 |
| C | 1.883974 | -3.551180 | 0.289969 |
| C | 0.394835 | -4.608093 | -1.439255 |
| H | 0.521988 | -2.530233 | -1.007390 |
| H | 2.711354 | -3.498909 | -0.435456 |
| H | 2.002156 | -2.737420 | 1.020685 |
| H | 1.995559 | -4.501695 | 0.834450 |
| H | 0.396770 | -5.588460 | -0.937194 |
| H | -0.536374 | -4.536150 | -2.018834 |
| H | 1.234145 | -4.598518 | -2.153156 |
| C | -2.512529 | 3.106895 | 3.011382 |
| H | -1.924609 | 1.461491 | 1.769121 |
| C | -0.687322 | 1.447388 | 3.519581 |
| C | 2.600458 | 2.652595 | -2.112057 |
| H | 0.497211 | 2.320191 | -2.354616 |
| C | 0.922527 | 4.342806 | -2.909348 |
| H | 2.835204 | 2.360054 | -3.147588 |
| H | 3.307183 | 3.447970 | -1.826307 |
| H | 2.795773 | 1.787586 | -1.460529 |
| H | 1.133666 | 4.075572 | -3.956719 |
| H | -0.111005 | 4.714122 | -2.862291 |
| H | 1.586126 | 5.181373 | -2.645931 |
| H | -3.243792 | 2.503399 | 3.570502 |
| H | -2.090135 | 3.840792 | 3.715576 |
| H | -3.061094 | 3.667684 | 2.240688 |
| H | -1.393281 | 0.923269 | 4.181024 |
| H | 0.000884 | 0.698720 | 3.100995 |
| H | -0.102065 | 2.134871 | 4.150526 |

124
TS2

| | | | |
|---|---|---|---|
| Al | -0.237693 | 0.003262 | -0.699995 |
| N | 0.172849 | 1.874107 | -0.455109 |

| | | | |
|---|---|---|---|
| N | -2.045769 | -0.097564 | -0.018936 |
| C | -2.755982 | 1.036503 | 0.099051 |
| C | -2.530612 | -1.435487 | 0.252503 |
| C | -0.851723 | 2.746727 | -0.346685 |
| C | 1.568154 | 2.176863 | -0.273337 |
| C | -0.738988 | 4.300982 | -0.472061 |
| C | -2.172986 | 2.298563 | -0.176452 |
| C | -4.247239 | 1.164313 | 0.558917 |
| C | -5.047702 | 1.896041 | -0.545040 |
| C | -5.017697 | -0.126498 | 0.879475 |
| C | -4.272076 | 2.020666 | 1.847203 |
| C | -1.365655 | 4.664129 | -1.839365 |
| C | -1.534169 | 5.002730 | 0.651183 |
| C | 0.670804 | 4.908300 | -0.434309 |
| H | -2.890811 | 3.107038 | -0.149521 |
| C | -0.021895 | -0.690945 | -2.520233 |
| H | -0.330544 | -1.744473 | -2.567956 |
| H | -0.635225 | -0.134644 | -3.248979 |
| H | 1.013420 | -0.661096 | -2.885799 |
| C | 2.462927 | 2.196954 | -1.380028 |
| C | 3.831457 | 2.375468 | -1.127037 |
| C | 4.326475 | 2.534617 | 0.162894 |
| C | 2.062689 | 2.351224 | 1.048680 |
| C | 3.440205 | 2.525704 | 1.236583 |
| C | 2.023644 | 2.095391 | -2.839659 |
| H | 4.526248 | 2.409696 | -1.969293 |
| H | 5.395908 | 2.687151 | 0.328961 |
| C | 1.166067 | 2.356382 | 2.281837 |
| H | 3.825318 | 2.676661 | 2.248314 |
| C | -3.087282 | -2.218867 | -0.796101 |
| C | -3.528537 | -3.513935 | -0.493474 |
| C | -3.417334 | -4.049394 | 0.783282 |
| C | -2.383404 | -1.983290 | 1.553817 |
| C | -2.838563 | -3.286688 | 1.789569 |
| C | -3.266953 | -1.742800 | -2.236033 |
| H | -3.978041 | -4.117595 | -1.284975 |
| H | -3.779394 | -5.058897 | 0.992860 |
| C | -1.776377 | -1.229839 | 2.732482 |
| H | -2.748626 | -3.709377 | 2.793272 |
| N | 3.451375 | -1.116419 | 0.713009 |
| C | 2.584735 | -1.343313 | -0.256253 |
| C | 2.254219 | -2.686052 | -0.806011 |
| C | 1.304265 | -3.552415 | -0.253692 |
| C | 1.068923 | -4.798013 | -0.836344 |
| C | 1.776596 | -5.187600 | -1.974237 |
| C | 2.945386 | -3.071599 | -1.966880 |
| C | 2.712880 | -4.320269 | -2.540392 |
| H | 0.723345 | -3.240475 | 0.615648 |
| H | 0.318721 | -5.461776 | -0.400901 |
| H | 1.590318 | -6.163773 | -2.428289 |
| H | 3.678853 | -2.398289 | -2.418489 |
| H | 3.261643 | -4.612992 | -3.438568 |

| | | | |
|---|---|---|---|
| C | 4.133308 | -2.015882 | 1.695342 |
| C | 5.137337 | -1.115164 | 2.423850 |
| C | 3.137298 | -2.600032 | 2.699130 |
| C | 4.888237 | -3.132729 | 0.965300 |
| H | 5.691058 | -1.701551 | 3.169766 |
| H | 5.871364 | -0.684393 | 1.725016 |
| H | 4.630171 | -0.294202 | 2.954163 |
| H | 3.683075 | -3.195661 | 3.445617 |
| H | 2.591541 | -1.808405 | 3.230310 |
| H | 2.413526 | -3.264692 | 2.210139 |
| H | 5.494035 | -3.688839 | 1.695810 |
| H | 4.211186 | -3.847786 | 0.480953 |
| H | 5.569157 | -2.722351 | 0.204614 |
| C | -2.707717 | -2.757909 | -3.246126 |
| C | -4.733855 | -1.462026 | -2.596349 |
| H | -2.710537 | -0.801350 | -2.351308 |
| H | -2.628634 | -2.300181 | -4.244134 |
| H | -1.714221 | -3.133220 | -2.964109 |
| H | -3.371827 | -3.630703 | -3.345662 |
| H | -4.812225 | -1.158279 | -3.652082 |
| H | -5.355184 | -2.362168 | -2.465002 |
| H | -5.177291 | -0.662208 | -1.990362 |
| C | -0.526807 | -1.949838 | 3.250420 |
| H | -1.470471 | -0.232676 | 2.378404 |
| C | -2.768110 | -1.034945 | 3.888729 |
| H | -0.078704 | -1.402071 | 4.093052 |
| H | -0.767285 | -2.961980 | 3.611282 |
| H | 0.233684 | -2.046133 | 2.463627 |
| H | -2.309629 | -0.432513 | 4.688658 |
| H | -3.689077 | -0.525556 | 3.574341 |
| H | -3.061234 | -1.998601 | 4.333840 |
| C | 2.848493 | 1.068791 | -3.629409 |
| H | 0.972130 | 1.773391 | -2.850059 |
| C | 2.105538 | 3.444334 | -3.573061 |
| H | 1.811740 | 3.317639 | -4.626678 |
| H | 1.447019 | 4.207543 | -3.139561 |
| H | 3.132252 | 3.843340 | -3.561000 |
| H | 2.389974 | 0.886119 | -4.612912 |
| H | 3.873164 | 1.427162 | -3.814250 |
| H | 2.927638 | 0.098812 | -3.116691 |
| C | 1.384057 | 1.092049 | 3.118439 |
| C | 1.354024 | 3.601969 | 3.158194 |
| H | 0.120495 | 2.345170 | 1.941827 |
| H | 0.680545 | 1.058997 | 3.964538 |
| H | 1.236882 | 0.184105 | 2.517179 |
| H | 2.402198 | 1.065964 | 3.539917 |
| H | 0.621327 | 3.599627 | 3.980059 |
| H | 2.353852 | 3.633412 | 3.618687 |
| H | 1.220343 | 4.536645 | 2.595402 |
| H | -1.315906 | 5.753871 | -1.990084 |
| H | -0.826063 | 4.184574 | -2.669550 |
| H | -2.420297 | 4.362599 | -1.908184 |

| | | | |
|---|---|---|---|
| H | 0.576448 | 5.991984 | -0.602278 |
| H | 1.166208 | 4.771389 | 0.533614 |
| H | 1.336765 | 4.518545 | -1.208024 |
| H | -1.361016 | 6.087756 | 0.590885 |
| H | -2.619888 | 4.853603 | 0.581109 |
| H | -1.209492 | 4.668303 | 1.647801 |
| H | -6.109494 | 1.927400 | -0.257630 |
| H | -4.726816 | 2.934707 | -0.701286 |
| H | -4.978181 | 1.376451 | -1.511571 |
| H | -6.036281 | 0.161196 | 1.181711 |
| H | -5.111747 | -0.803057 | 0.024911 |
| H | -4.582400 | -0.694690 | 1.707267 |
| H | -5.309970 | 2.111335 | 2.203090 |
| H | -3.683876 | 1.553999 | 2.650919 |
| H | -3.882672 | 3.035678 | 1.694491 |
| H | 0.931565 | -0.833349 | 0.231137 |
| H | 2.518946 | -0.511964 | -0.963558 |
| H | 3.705489 | -0.132211 | 0.812864 |

124
tBuBDI Al Me (+) + PhC(H)2-N(H)tBu

| | | | |
|---|---|---|---|
| Al | -1.069957 | 0.045834 | 0.322786 |
| N | -1.974551 | 1.590364 | -0.155493 |
| N | -2.344710 | -1.280016 | 0.100207 |
| C | -3.593154 | -0.995923 | -0.333761 |
| C | -1.757742 | -2.579245 | 0.360173 |
| C | -3.263728 | 1.557844 | -0.563069 |
| C | -1.127976 | 2.744889 | 0.073409 |
| C | -4.093883 | 2.784987 | -1.038643 |
| C | -3.958465 | 0.333637 | -0.625684 |
| C | -4.745033 | -2.026405 | -0.514991 |
| C | -5.331375 | -1.895296 | -1.939263 |
| C | -4.393376 | -3.509129 | -0.324600 |
| C | -5.835199 | -1.675615 | 0.525083 |
| C | -4.390304 | 2.574576 | -2.542495 |
| C | -5.425319 | 2.841643 | -0.255608 |
| C | -3.442994 | 4.167705 | -0.889913 |
| H | -4.981415 | 0.435070 | -0.966266 |
| C | 0.738757 | -0.135498 | 0.971147 |
| H | 0.976983 | -1.190173 | 1.175050 |
| H | 1.468033 | 0.235357 | 0.234924 |
| H | 0.891357 | 0.445338 | 1.893863 |
| C | -0.163805 | 3.108343 | -0.895117 |
| C | 0.733247 | 4.135355 | -0.570229 |
| C | 0.691185 | 4.772589 | 0.663450 |
| C | -1.190144 | 3.385246 | 1.336175 |
| C | -0.265321 | 4.398800 | 1.604159 |
| C | -0.075031 | 2.468091 | -2.276074 |
| H | 1.481651 | 4.442633 | -1.304486 |
| H | 1.403170 | 5.568569 | 0.893938 |
| C | -2.226156 | 2.997860 | 2.386313 |
| H | -0.295016 | 4.913322 | 2.566351 |

| | | | |
|---|---|---|---|
| C | -1.159045 | -3.271689 | -0.720759 |
| C | -0.479400 | -4.461001 | -0.442820 |
| C | -0.384339 | -4.951501 | 0.857480 |
| C | -1.660886 | -3.051861 | 1.688036 |
| C | -0.969829 | -4.252020 | 1.905334 |
| C | -1.236888 | -2.740219 | -2.147412 |
| H | -0.016620 | -5.020155 | -1.257635 |
| H | 0.149800 | -5.884468 | 1.052053 |
| C | -2.275561 | -2.331488 | 2.881801 |
| H | -0.890017 | -4.646317 | 2.921190 |
| N | 6.900145 | 0.921655 | 0.679007 |
| C | 6.023087 | -0.160430 | 1.077301 |
| C | 5.447437 | -0.969334 | -0.073915 |
| C | 5.064428 | -2.303564 | 0.114864 |
| C | 4.501586 | -3.042393 | -0.927623 |
| C | 4.314807 | -2.455033 | -2.180838 |
| C | 5.252205 | -0.388737 | -1.332884 |
| C | 4.691962 | -1.124109 | -2.378591 |
| H | 5.224858 | -2.777984 | 1.088561 |
| H | 4.226766 | -4.088320 | -0.765400 |
| H | 3.898342 | -3.038679 | -3.006475 |
| H | 5.577814 | 0.644445 | -1.477375 |
| H | 4.564952 | -0.660822 | -3.361287 |
| C | 8.358241 | 0.699396 | 0.661253 |
| C | 8.981589 | 1.985604 | 0.108672 |
| C | 8.919949 | 0.427458 | 2.069354 |
| C | 8.698907 | -0.470769 | -0.266625 |
| H | 10.078236 | 1.906662 | 0.061667 |
| H | 8.601272 | 2.195771 | -0.902047 |
| H | 8.739704 | 2.849963 | 0.749922 |
| H | 10.012561 | 0.293006 | 2.043937 |
| H | 8.702755 | 1.269050 | 2.747729 |
| H | 8.491282 | -0.485288 | 2.512602 |
| H | 9.790131 | -0.601322 | -0.325973 |
| H | 8.273307 | -1.419927 | 0.093441 |
| H | 8.315320 | -0.293360 | -1.281920 |
| C | -0.096751 | -1.756975 | -2.434518 |
| C | -1.269649 | -3.838181 | -3.213862 |
| H | -2.181196 | -2.179620 | -2.239484 |
| H | -0.163175 | -1.357740 | -3.458097 |
| H | -0.109195 | -0.882411 | -1.756172 |
| H | 0.889364 | -2.228935 | -2.308031 |
| H | -1.475324 | -3.398998 | -4.201834 |
| H | -0.308581 | -4.368845 | -3.294007 |
| H | -2.050936 | -4.585247 | -3.007809 |
| C | -1.207799 | -1.908998 | 3.900425 |
| H | -2.765353 | -1.416451 | 2.508227 |
| C | -3.359255 | -3.174176 | 3.569324 |
| H | -1.660983 | -1.325576 | 4.716599 |
| H | -0.720637 | -2.784806 | 4.356412 |
| H | -0.415090 | -1.297800 | 3.444070 |
| H | -3.828663 | -2.604293 | 4.386157 |

| | | | |
|---|---|---|---|
| H | -4.153513 | -3.480073 | 2.873093 |
| H | -2.935806 | -4.090248 | 4.009594 |
| C | 1.302616 | 1.847238 | -2.541845 |
| H | -0.824687 | 1.659936 | -2.322842 |
| C | -0.419229 | 3.468038 | -3.390296 |
| H | -0.408172 | 2.968394 | -4.371412 |
| H | -1.411607 | 3.921250 | -3.255415 |
| H | 0.314825 | 4.287807 | -3.430268 |
| H | 1.312320 | 1.335860 | -3.516505 |
| H | 2.091214 | 2.614746 | -2.570243 |
| H | 1.591199 | 1.113926 | -1.775434 |
| C | -1.692124 | 1.911748 | 3.327078 |
| C | -2.749634 | 4.188819 | 3.195188 |
| H | -3.090996 | 2.569171 | 1.855567 |
| H | -2.450574 | 1.626111 | 4.072121 |
| H | -1.406787 | 0.990643 | 2.789815 |
| H | -0.795393 | 2.255517 | 3.865892 |
| H | -3.595908 | 3.874912 | 3.825087 |
| H | -1.984494 | 4.600838 | 3.870826 |
| H | -3.097702 | 5.005807 | 2.545345 |
| H | -4.978990 | 3.425048 | -2.918917 |
| H | -3.462373 | 2.523970 | -3.131508 |
| H | -4.965016 | 1.658772 | -2.738545 |
| H | -4.141855 | 4.911844 | -1.300662 |
| H | -3.256335 | 4.441513 | 0.155173 |
| H | -2.501844 | 4.267468 | -1.440507 |
| H | -5.990758 | 3.729196 | -0.576503 |
| H | -6.072665 | 1.970593 | -0.424338 |
| H | -5.253422 | 2.932844 | 0.827745 |
| H | -6.124366 | -2.646678 | -2.069666 |
| H | -5.782240 | -0.914393 | -2.141173 |
| H | -4.567641 | -2.083404 | -2.709292 |
| H | -5.317106 | -4.091190 | -0.461750 |
| H | -3.667942 | -3.874642 | -1.061285 |
| H | -4.009321 | -3.740106 | 0.674643 |
| H | -6.672566 | -2.383083 | 0.425812 |
| H | -5.450093 | -1.756465 | 1.552651 |
| H | -6.238235 | -0.661806 | 0.394040 |
| H | 6.486019 | -0.861693 | 1.800951 |
| H | 5.170828 | 0.283688 | 1.622769 |
| H | 6.706148 | 1.750741 | 1.232970 |

Autoinduced Catalytic Cycle

124

A-K11 koordiniere Amin

| | | | |
|---|---|---|---|
| Al | 0.150956 | -0.217877 | -0.738324 |
| N | 2.037370 | -0.134385 | -0.319834 |
| N | -0.495792 | 1.520747 | -0.131734 |
| C | 0.390221 | 2.504063 | -0.377431 |
| C | -1.860669 | 1.710262 | 0.310414 |
| C | 2.536527 | 1.066905 | -0.709074 |
| C | 2.846886 | -1.220931 | 0.177149 |
| C | 3.991414 | 1.316688 | -1.247548 |
| C | 1.708111 | 2.198616 | -0.805089 |
| C | 0.140131 | 4.049333 | -0.306119 |
| C | 0.207214 | 4.593689 | -1.753906 |
| C | -1.175805 | 4.532709 | 0.325428 |
| C | 1.270383 | 4.723692 | 0.508921 |
| C | 3.871917 | 1.206086 | -2.787302 |
| C | 4.503328 | 2.739199 | -0.922903 |
| C | 5.099316 | 0.358261 | -0.778297 |
| H | 2.230456 | 3.067986 | -1.177497 |
| C | -0.008644 | -0.395417 | -2.690039 |
| H | -0.956272 | -0.030145 | -3.111460 |
| H | 0.788533 | 0.218530 | -3.141781 |
| H | 0.127490 | -1.420339 | -3.066603 |
| C | 3.170908 | -2.331875 | -0.647583 |
| C | 3.895563 | -3.389126 | -0.079918 |
| C | 4.326933 | -3.354298 | 1.239961 |
| C | 3.259216 | -1.184961 | 1.538903 |
| C | 4.015340 | -2.254810 | 2.032454 |
| C | 2.881329 | -2.402093 | -2.147119 |
| H | 4.155588 | -4.249190 | -0.701884 |
| H | 4.908633 | -4.182435 | 1.651893 |
| C | 2.880302 | -0.050530 | 2.492455 |
| H | 4.354920 | -2.236756 | 3.069592 |
| C | -2.903829 | 1.957817 | -0.629208 |
| C | -4.207562 | 2.124229 | -0.141307 |
| C | -4.502426 | 2.051156 | 1.213312 |
| C | -2.158618 | 1.590534 | 1.697813 |
| C | -3.483014 | 1.776419 | 2.116945 |
| C | -2.714715 | 2.120110 | -2.138308 |
| H | -5.013602 | 2.331056 | -0.848797 |
| H | -5.525522 | 2.202903 | 1.565382 |
| C | -1.096970 | 1.311030 | 2.759627 |
| H | -3.721384 | 1.713231 | 3.179670 |
| N | -1.242624 | -1.561441 | 0.162595 |
| C | -2.128296 | -1.930699 | -0.992764 |
| H | -2.197779 | -1.026116 | -1.609303 |
| C | -3.530315 | -2.417070 | -0.718004 |
| C | -3.953742 | -3.651377 | -1.227734 |
| C | -5.264315 | -4.091735 | -1.034440 |
| C | -6.169168 | -3.300105 | -0.327037 |
| C | -4.454742 | -1.617679 | -0.031235 |
| C | -5.761279 | -2.060296 | 0.170707 |
| H | -3.252432 | -4.278906 | -1.785806 |
| H | -5.578032 | -5.057251 | -1.438535 |

| | | | |
|---|---|---|---|
| H | -7.194503 | -3.643950 | -0.171044 |
| H | -4.161899 | -0.634275 | 0.348154 |
| H | -6.469730 | -1.428750 | 0.712364 |
| C | -0.921990 | -2.710410 | 1.133106 |
| C | 0.083718 | -2.181740 | 2.142631 |
| C | -2.174605 | -3.164167 | 1.897098 |
| C | -0.312256 | -3.890303 | 0.385851 |
| H | 0.354439 | -2.984394 | 2.842882 |
| H | 1.010791 | -1.856024 | 1.656998 |
| H | -0.329211 | -1.356694 | 2.734986 |
| H | -1.857229 | -3.825891 | 2.716587 |
| H | -2.710433 | -2.315788 | 2.349254 |
| H | -2.884400 | -3.721361 | 1.276038 |
| H | -0.079137 | -4.681976 | 1.112136 |
| H | -0.995164 | -4.329043 | -0.354860 |
| H | 0.630753 | -3.616230 | -0.105100 |
| H | -1.779726 | -0.889177 | 0.728302 |
| H | -1.586622 | -2.662025 | -1.604527 |
| C | -3.493508 | 1.068349 | -2.943561 |
| C | -3.157782 | 3.504646 | -2.643797 |
| H | -1.643667 | 2.000515 | -2.362161 |
| H | -3.266498 | 1.166105 | -4.016247 |
| H | -3.269010 | 0.035520 | -2.648957 |
| H | -4.579976 | 1.202587 | -2.827431 |
| H | -2.918806 | 3.607251 | -3.713661 |
| H | -4.246257 | 3.634853 | -2.542758 |
| H | -2.677594 | 4.336851 | -2.114762 |
| C | -1.631189 | 0.485565 | 3.937023 |
| H | -0.293512 | 0.734271 | 2.271604 |
| C | -0.447782 | 2.587107 | 3.310026 |
| H | -0.796797 | 0.131133 | 4.560598 |
| H | -2.278451 | 1.086271 | 4.594063 |
| H | -2.213227 | -0.391088 | 3.616369 |
| H | 0.262748 | 2.336769 | 4.112897 |
| H | 0.106244 | 3.135444 | 2.539774 |
| H | -1.205056 | 3.264844 | 3.734916 |
| C | 1.912059 | -3.525425 | -2.535191 |
| H | 2.417505 | -1.448713 | -2.437248 |
| C | 4.169233 | -2.564728 | -2.972757 |
| H | 3.943216 | -2.475690 | -4.046777 |
| H | 4.930636 | -1.811857 | -2.728611 |
| H | 4.623959 | -3.555405 | -2.818099 |
| H | 1.789368 | -3.559882 | -3.628844 |
| H | 2.285788 | -4.511040 | -2.215612 |
| H | 0.914171 | -3.391846 | -2.097888 |
| C | 2.608944 | -0.533688 | 3.923492 |
| C | 3.922520 | 1.073630 | 2.547708 |
| H | 1.948549 | 0.392656 | 2.103208 |
| H | 2.152377 | 0.276769 | 4.512119 |
| H | 1.937690 | -1.402480 | 3.961501 |
| H | 3.539000 | -0.810037 | 4.443901 |
| H | 3.626406 | 1.831593 | 3.289755 |

| | | | |
|---|---|---|---|
| H | 4.909403 | 0.684815 | 2.845382 |
| H | 4.035170 | 1.584632 | 1.584712 |
| H | 4.854976 | 1.399376 | -3.244545 |
| H | 3.548135 | 0.206401 | -3.105249 |
| H | 3.160683 | 1.940621 | -3.193056 |
| H | 6.035727 | 0.661221 | -1.270798 |
| H | 5.270182 | 0.405858 | 0.303664 |
| H | 4.919425 | -0.685615 | -1.040867 |
| H | 5.562924 | 2.804936 | -1.209288 |
| H | 3.988432 | 3.532230 | -1.482951 |
| H | 4.437548 | 2.976991 | 0.148714 |
| H | -0.032052 | 5.668159 | -1.747057 |
| H | 1.206107 | 4.484408 | -2.198692 |
| H | -0.510238 | 4.095120 | -2.418391 |
| H | -1.155890 | 5.632970 | 0.336403 |
| H | -2.069251 | 4.232033 | -0.226151 |
| H | -1.296805 | 4.202873 | 1.362936 |
| H | 1.076641 | 5.805922 | 0.554001 |
| H | 1.310289 | 4.353374 | 1.543358 |
| H | 2.266390 | 4.589450 | 0.070086 |

126

A-K2 Al+H2+Amin

| | | | |
|---|---|---|---|
| Al | 0.166488 | -0.225244 | -0.732457 |
| N | 2.064114 | -0.178229 | -0.370246 |
| N | -0.417818 | 1.537775 | -0.129015 |
| C | 0.470737 | 2.490385 | -0.467808 |
| C | -1.751112 | 1.769996 | 0.385560 |
| C | 2.571111 | 0.993978 | -0.831606 |
| C | 2.869613 | -1.255961 | 0.149883 |
| C | 4.004508 | 1.184365 | -1.444707 |
| C | 1.760941 | 2.137694 | -0.943430 |
| C | 0.246399 | 4.040500 | -0.470225 |
| C | 0.242592 | 4.501010 | -1.948329 |
| C | -1.030793 | 4.578809 | 0.195105 |
| C | 1.426318 | 4.739527 | 0.247074 |
| C | 3.812075 | 1.014206 | -2.971668 |
| C | 4.564240 | 2.604565 | -1.200642 |
| C | 5.107530 | 0.213227 | -0.990803 |
| H | 2.279923 | 2.976497 | -1.384854 |
| C | -0.054970 | -0.416227 | -2.678187 |
| H | -1.010446 | -0.044568 | -3.076207 |
| H | 0.734533 | 0.183410 | -3.160889 |
| H | 0.058051 | -1.447097 | -3.046339 |
| C | 3.126879 | -2.415868 | -0.628401 |
| C | 3.850302 | -3.462748 | -0.041027 |
| C | 4.343199 | -3.372278 | 1.254693 |
| C | 3.345095 | -1.161679 | 1.487644 |
| C | 4.096089 | -2.225625 | 2.001731 |
| C | 2.755234 | -2.551783 | -2.104081 |
| H | 4.059124 | -4.360242 | -0.628457 |
| H | 4.922234 | -4.193953 | 1.682965 |

| | | | |
|---|---|---|---|
| C | 3.039707 | 0.028908 | 2.396885 |
| H | 4.483333 | -2.163760 | 3.020417 |
| C | -2.846143 | 1.971486 | -0.505070 |
| C | -4.122933 | 2.155474 | 0.044060 |
| C | -4.342525 | 2.148443 | 1.414836 |
| C | -1.968888 | 1.737731 | 1.792782 |
| C | -3.270420 | 1.937347 | 2.273061 |
| C | -2.738378 | 2.066121 | -2.027669 |
| H | -4.967276 | 2.324811 | -0.628023 |
| H | -5.346346 | 2.308943 | 1.815236 |
| C | -0.851245 | 1.539144 | 2.814650 |
| H | -3.447804 | 1.935516 | 3.349591 |
| N | -1.223411 | -1.544486 | 0.203396 |
| C | -2.065555 | -2.063431 | -0.927301 |
| H | -2.114953 | -1.252137 | -1.663326 |
| C | -3.472805 | -2.520310 | -0.626914 |
| C | -3.884878 | -3.807141 | -0.996397 |
| C | -5.198647 | -4.226553 | -0.778550 |
| C | -6.118535 | -3.361330 | -0.186384 |
| C | -4.412290 | -1.650316 | -0.054714 |
| C | -5.721994 | -2.070675 | 0.171894 |
| H | -3.172910 | -4.492832 | -1.465357 |
| H | -5.502797 | -5.233863 | -1.073075 |
| H | -7.146247 | -3.688404 | -0.011231 |
| H | -4.128513 | -0.629426 | 0.218778 |
| H | -6.441278 | -1.383223 | 0.623713 |
| C | -0.924656 | -2.579212 | 1.306243 |
| C | 0.143251 | -1.986147 | 2.208825 |
| C | -2.179699 | -2.855913 | 2.146919 |
| C | -0.401880 | -3.881742 | 0.709256 |
| H | 0.358747 | -2.684989 | 3.029168 |
| H | 1.083518 | -1.826702 | 1.667936 |
| H | -0.182490 | -1.042895 | 2.660803 |
| H | -1.884528 | -3.435428 | 3.034077 |
| H | -2.647057 | -1.924944 | 2.503618 |
| H | -2.940148 | -3.433594 | 1.609915 |
| H | -0.160207 | -4.566576 | 1.534699 |
| H | -1.141084 | -4.393242 | 0.078069 |
| H | 0.523231 | -3.728064 | 0.137876 |
| H | -1.793778 | -0.830413 | 0.679381 |
| H | -1.502258 | -2.866408 | -1.417311 |
| C | -3.549166 | 0.973379 | -2.739911 |
| C | -3.214858 | 3.424268 | -2.572139 |
| H | -1.679820 | 1.941621 | -2.301613 |
| H | -3.370890 | 1.011225 | -3.825574 |
| H | -3.307914 | -0.039604 | -2.395296 |
| H | -4.629881 | 1.112979 | -2.583202 |
| H | -3.041253 | 3.473846 | -3.658276 |
| H | -4.295351 | 3.560729 | -2.411638 |
| H | -2.704499 | 4.280973 | -2.115069 |
| C | -1.305734 | 0.752590 | 4.051777 |
| H | -0.051734 | 0.968580 | 2.314392 |

| | | | |
|---|---|---|---|
| C | -0.223105 | 2.858431 | 3.283882 |
| H | -0.432335 | 0.439639 | 4.643136 |
| H | -1.929801 | 1.368874 | 4.716734 |
| H | -1.885293 | -0.147110 | 3.799460 |
| H | 0.522271 | 2.664570 | 4.070709 |
| H | 0.287163 | 3.389735 | 2.472617 |
| H | -0.985481 | 3.531739 | 3.706745 |
| C | 1.710592 | -3.641846 | -2.369672 |
| H | 2.321688 | -1.593082 | -2.422565 |
| C | 3.985055 | -2.820326 | -2.987277 |
| H | 3.703126 | -2.778813 | -4.050910 |
| H | 4.790324 | -2.090490 | -2.826728 |
| H | 4.405063 | -3.821007 | -2.802049 |
| H | 1.511191 | -3.726255 | -3.449304 |
| H | 2.056362 | -4.627616 | -2.020470 |
| H | 0.753275 | -3.432246 | -1.874105 |
| C | 2.788563 | -0.378967 | 3.854828 |
| C | 4.130572 | 1.107246 | 2.373454 |
| H | 2.116575 | 0.492817 | 2.012042 |
| H | 2.387133 | 0.476660 | 4.419210 |
| H | 2.079423 | -1.212600 | 3.948836 |
| H | 3.719603 | -0.676664 | 4.361479 |
| H | 3.887222 | 1.911703 | 3.085117 |
| H | 5.107209 | 0.689217 | 2.665447 |
| H | 4.241002 | 1.566675 | 1.384512 |
| H | 4.777538 | 1.164353 | -3.479737 |
| H | 3.452316 | 0.009798 | -3.231796 |
| H | 3.100000 | 1.748180 | -3.376912 |
| H | 6.028278 | 0.473619 | -1.534516 |
| H | 5.328311 | 0.291110 | 0.080307 |
| H | 4.887944 | -0.833393 | -1.209990 |
| H | 5.610786 | 2.633109 | -1.536942 |
| H | 4.041931 | 3.387686 | -1.767712 |
| H | 4.552647 | 2.885504 | -0.137576 |
| H | 0.033942 | 5.581111 | -1.988778 |
| H | 1.208982 | 4.335652 | -2.444552 |
| H | -0.528704 | 3.989136 | -2.538661 |
| H | -0.998928 | 5.677379 | 0.138223 |
| H | -1.952753 | 4.257214 | -0.294075 |
| H | -1.105442 | 4.313184 | 1.254816 |
| H | 1.245195 | 5.824847 | 0.252902 |
| H | 1.522451 | 4.415405 | 1.293298 |
| H | 2.394568 | 4.574124 | -0.240758 |
| H | -10.033166 | 0.783598 | -2.288746 |
| H | -9.673748 | 0.292123 | -2.743395 |

126

A-TS1

| | | | |
|---|---|---|---|
| Al | 0.609869 | -0.034804 | 0.456294 |
| N | 1.511008 | 1.519701 | -0.081000 |
| N | 1.619173 | -1.428073 | -0.255295 |
| C | 2.519783 | -1.151696 | -1.236662 |

| | | | |
|---|---|---|---|
| C | 1.517102 | -2.684947 | 0.465268 |
| C | 2.532734 | 1.422069 | -0.955749 |
| C | 1.001381 | 2.731066 | 0.535597 |
| C | 3.489110 | 2.571420 | -1.396502 |
| C | 2.852728 | 0.176067 | -1.547022 |
| C | 3.190655 | -2.211682 | -2.161577 |
| C | 4.665021 | -1.854068 | -2.454421 |
| C | 3.197624 | -3.667749 | -1.671588 |
| C | 2.389076 | -2.154980 | -3.484998 |
| C | 4.934135 | 2.129693 | -1.054222 |
| C | 3.372480 | 2.763981 | -2.925671 |
| C | 3.295878 | 3.945743 | -0.738272 |
| H | 3.583661 | 0.260451 | -2.340126 |
| C | -0.652306 | -0.167217 | 1.909261 |
| H | -0.169014 | -0.564096 | 2.815717 |
| H | -1.078908 | 0.817499 | 2.153971 |
| H | -1.482068 | -0.846879 | 1.666646 |
| C | 1.299223 | 2.991481 | 1.897484 |
| C | 0.765752 | 4.147643 | 2.480494 |
| C | -0.061829 | 5.007221 | 1.768612 |
| C | 0.128380 | 3.577723 | -0.194939 |
| C | -0.383510 | 4.710060 | 0.450232 |
| C | 2.186047 | 2.098129 | 2.759213 |
| H | 1.002481 | 4.378695 | 3.520875 |
| H | -0.465222 | 5.903342 | 2.245855 |
| C | -0.294841 | 3.333806 | -1.639510 |
| H | -1.049136 | 5.379365 | -0.099539 |
| C | 2.452980 | -2.940199 | 1.500110 |
| C | 2.345246 | -4.141069 | 2.212180 |
| C | 1.337779 | -5.058709 | 1.941654 |
| C | 0.462634 | -3.593507 | 0.202456 |
| C | 0.403077 | -4.773793 | 0.953572 |
| C | 3.558318 | -1.969497 | 1.901944 |
| H | 3.067819 | -4.359762 | 3.002042 |
| H | 1.274919 | -5.991365 | 2.507098 |
| C | -0.603210 | -3.377041 | -0.865093 |
| H | -0.393682 | -5.493889 | 0.752781 |
| N | -3.772605 | -0.108914 | -0.896512 |
| C | -4.304350 | 0.665782 | 0.225724 |
| H | -3.497186 | 0.720731 | 0.977052 |
| C | -5.558389 | 0.170281 | 0.926459 |
| C | -6.712331 | 0.961073 | 0.987845 |
| C | -7.858365 | 0.511728 | 1.648256 |
| C | -7.865996 | -0.740856 | 2.261363 |
| C | -5.576734 | -1.080817 | 1.560560 |
| C | -6.718222 | -1.536388 | 2.218330 |
| H | -6.716186 | 1.948087 | 0.515141 |
| H | -8.748880 | 1.144505 | 1.683281 |
| H | -8.761496 | -1.095711 | 2.777370 |
| H | -4.682931 | -1.713277 | 1.545571 |
| H | -6.712612 | -2.514459 | 2.706453 |
| C | -4.548592 | -0.213063 | -2.163014 |

| | | | |
|---|---|---|---|
| C | -3.559536 | -0.679253 | -3.239827 |
| C | -5.696037 | -1.233690 | -2.064271 |
| C | -5.115766 | 1.150713 | -2.563489 |
| H | -4.068654 | -0.829607 | -4.203958 |
| H | -2.758322 | 0.060992 | -3.384580 |
| H | -3.092884 | -1.638538 | -2.962371 |
| H | -6.208344 | -1.336435 | -3.033761 |
| H | -5.317077 | -2.230785 | -1.784942 |
| H | -6.444233 | -0.936831 | -1.316761 |
| H | -5.554015 | 1.086503 | -3.570339 |
| H | -5.914804 | 1.481693 | -1.884448 |
| H | -4.332903 | 1.922779 | -2.585903 |
| H | -3.580961 | -1.058329 | -0.579432 |
| H | -4.462672 | 1.702064 | -0.105323 |
| H | -1.420869 | 0.028246 | -1.027819 |
| H | -0.679808 | 0.016400 | -1.271939 |
| H | 3.686660 | -4.279874 | -2.444198 |
| H | 3.763676 | -3.799240 | -0.741231 |
| H | 2.198345 | -4.081248 | -1.515532 |
| H | 5.127445 | -2.682070 | -3.011070 |
| H | 4.789311 | -0.957971 | -3.077964 |
| H | 5.242217 | -1.712423 | -1.528574 |
| H | 2.812033 | -2.877046 | -4.200755 |
| H | 1.332227 | -2.417589 | -3.329912 |
| H | 2.427765 | -1.157808 | -3.947478 |
| H | 5.630621 | 2.932933 | -1.338160 |
| H | 5.054587 | 1.950876 | 0.024863 |
| H | 5.245764 | 1.219200 | -1.582007 |
| H | 4.099138 | 4.604518 | -1.101177 |
| H | 2.345075 | 4.422526 | -0.992132 |
| H | 3.372455 | 3.912249 | 0.354201 |
| H | 4.032708 | 3.588709 | -3.233818 |
| H | 3.676083 | 1.875633 | -3.496761 |
| H | 2.349117 | 3.026934 | -3.227563 |
| C | 1.579584 | 1.835341 | 4.143471 |
| H | 2.285866 | 1.121674 | 2.250553 |
| C | 3.604829 | 2.664638 | 2.910945 |
| H | 2.168784 | 1.077497 | 4.680830 |
| H | 1.587686 | 2.741385 | 4.767990 |
| H | 0.539834 | 1.483753 | 4.083161 |
| H | 4.214925 | 2.013326 | 3.555660 |
| H | 4.122646 | 2.760210 | 1.946857 |
| H | 3.579376 | 3.662766 | 3.375808 |
| C | 0.007406 | 4.523875 | -2.563200 |
| H | 0.264185 | 2.464490 | -2.021004 |
| C | -1.789708 | 3.002878 | -1.727912 |
| H | -0.228683 | 4.262471 | -3.606396 |
| H | -0.607238 | 5.399626 | -2.304339 |
| H | 1.059064 | 4.839368 | -2.527147 |
| H | -2.066962 | 2.777724 | -2.769367 |
| H | -2.074469 | 2.139304 | -1.112187 |
| H | -2.401983 | 3.858495 | -1.402388 |

| | | | |
|---|---|---|---|
| C | 3.380320 | -1.515314 | 3.357143 |
| C | 4.963297 | -2.550920 | 1.696552 |
| H | 3.481664 | -1.075104 | 1.263651 |
| H | 4.119694 | -0.741521 | 3.615181 |
| H | 2.375569 | -1.105527 | 3.538222 |
| H | 3.522748 | -2.350208 | 4.060416 |
| H | 5.729775 | -1.808063 | 1.967005 |
| H | 5.129238 | -3.437560 | 2.328064 |
| H | 5.140859 | -2.850697 | 0.653937 |
| C | -0.571558 | -4.449880 | -1.964068 |
| C | -2.003737 | -3.328797 | -0.240809 |
| H | -0.407753 | -2.406185 | -1.347832 |
| H | -1.322207 | -4.226318 | -2.737999 |
| H | 0.406672 | -4.517627 | -2.459546 |
| H | -0.806006 | -5.446237 | -1.558537 |
| H | -2.263727 | -4.287248 | 0.233532 |
| H | -2.082744 | -2.553388 | 0.535025 |
| H | -2.764761 | -3.136328 | -1.012130 |

126

A-I1 AlHMe+Ammonium

| | | | |
|---|---|---|---|
| Al | 0.659065 | -0.073520 | -0.997543 |
| N | 2.415693 | -0.575319 | -0.390405 |
| N | 0.489230 | 1.698535 | -0.196435 |
| C | 1.628169 | 2.399548 | -0.192723 |
| C | -0.828057 | 2.149550 | 0.167478 |
| C | 3.282950 | 0.442996 | -0.603161 |
| C | 2.732152 | -1.861508 | 0.170064 |
| C | 4.776970 | 0.263703 | -1.012333 |
| C | 2.846561 | 1.780928 | -0.595200 |
| C | 1.804782 | 3.892009 | 0.220549 |
| C | 2.335326 | 4.684205 | -0.996261 |
| C | 0.556195 | 4.624998 | 0.731316 |
| C | 2.846296 | 3.951826 | 1.361247 |
| C | 4.830383 | 0.617856 | -2.519261 |
| C | 5.706656 | 1.220443 | -0.239776 |
| C | 5.346709 | -1.153154 | -0.871249 |
| H | 3.638390 | 2.491448 | -0.801962 |
| C | 0.575807 | 0.046741 | -2.965970 |
| H | -0.432725 | 0.255393 | -3.351521 |
| H | 1.232081 | 0.857282 | -3.327088 |
| H | 0.910885 | -0.872967 | -3.468618 |
| C | 2.556416 | -3.040628 | -0.603566 |
| C | 2.727734 | -4.288073 | 0.007893 |
| C | 3.096626 | -4.400897 | 1.342958 |
| C | 3.131975 | -1.968259 | 1.530003 |
| C | 3.309033 | -3.244113 | 2.081834 |
| C | 2.254594 | -3.015814 | -2.096230 |
| H | 2.594467 | -5.194916 | -0.587484 |
| H | 3.236073 | -5.383782 | 1.799575 |
| C | 3.381587 | -0.774643 | 2.445160 |
| H | 3.620797 | -3.330780 | 3.125721 |

| | | | |
|---|---|---|---|
| C | -1.682247 | 2.723513 | -0.815910 |
| C | -2.971975 | 3.120201 | -0.435501 |
| C | -3.439923 | 2.952312 | 0.861996 |
| C | -1.313979 | 1.931448 | 1.483331 |
| C | -2.612871 | 2.350237 | 1.803154 |
| C | -1.285149 | 2.954956 | -2.270257 |
| H | -3.622896 | 3.586053 | -1.179205 |
| H | -4.442176 | 3.289653 | 1.136678 |
| C | -0.491269 | 1.286755 | 2.591448 |
| H | -2.975797 | 2.220207 | 2.826373 |
| N | -2.942505 | -1.681313 | 0.235619 |
| C | -3.769175 | -0.778518 | -0.636871 |
| H | -3.306531 | 0.215926 | -0.525367 |
| C | -5.234288 | -0.720102 | -0.298532 |
| C | -6.194584 | -1.147968 | -1.223952 |
| C | -7.554978 | -1.078458 | -0.918538 |
| C | -7.968808 | -0.583729 | 0.318513 |
| C | -5.661614 | -0.200501 | 0.932103 |
| C | -7.019268 | -0.141857 | 1.242988 |
| H | -5.880179 | -1.533185 | -2.198335 |
| H | -8.293636 | -1.411793 | -1.651303 |
| H | -9.033144 | -0.531971 | 0.559877 |
| H | -4.930615 | 0.181966 | 1.651476 |
| H | -7.340105 | 0.262820 | 2.205902 |
| C | -3.117358 | -3.205387 | 0.217657 |
| C | -1.902958 | -3.756591 | 0.969890 |
| C | -4.405034 | -3.588640 | 0.945274 |
| C | -3.127924 | -3.686164 | -1.229912 |
| H | -1.965646 | -4.853138 | 1.006711 |
| H | -0.954347 | -3.489280 | 0.480908 |
| H | -1.872173 | -3.396630 | 2.010510 |
| H | -4.457316 | -4.685201 | 1.008991 |
| H | -4.420963 | -3.200277 | 1.975952 |
| H | -5.305830 | -3.236906 | 0.427058 |
| H | -3.121438 | -4.785250 | -1.236969 |
| H | -4.030613 | -3.364919 | -1.768386 |
| H | -2.238420 | -3.348994 | -1.782859 |
| H | -3.042820 | -1.370467 | 1.209011 |
| H | -3.609801 | -1.098208 | -1.673598 |
| C | -2.227882 | 2.210087 | -3.227039 |
| C | -1.259197 | 4.442644 | -2.651722 |
| H | -0.272279 | 2.552637 | -2.413073 |
| H | -1.856151 | 2.272068 | -4.261305 |
| H | -2.322507 | 1.145286 | -2.968681 |
| H | -3.238987 | 2.647010 | -3.216601 |
| H | -0.931323 | 4.560048 | -3.696329 |
| H | -2.258505 | 4.898465 | -2.569203 |
| H | -0.576887 | 5.029430 | -2.022229 |
| C | -1.141795 | -0.020021 | 3.067456 |
| H | 0.496128 | 1.036038 | 2.176071 |
| C | -0.274798 | 2.228978 | 3.783876 |
| H | -0.608088 | -0.437084 | 3.934059 |

| | | | |
|---|---|---|---|
| H | -2.185743 | 0.140669 | 3.382915 |
| H | -1.118130 | -0.782688 | 2.274311 |
| H | 0.382680 | 1.757484 | 4.530136 |
| H | 0.190198 | 3.177986 | 3.481672 |
| H | -1.222420 | 2.472649 | 4.289576 |
| C | 0.833029 | -3.501889 | -2.401791 |
| H | 2.332522 | -1.969496 | -2.425912 |
| C | 3.270110 | -3.822685 | -2.918117 |
| H | 3.087886 | -3.679657 | -3.994809 |
| H | 4.306135 | -3.517856 | -2.710377 |
| H | 3.197416 | -4.902675 | -2.716483 |
| H | 0.622061 | -3.452164 | -3.481891 |
| H | 0.696743 | -4.547988 | -2.083395 |
| H | 0.078614 | -2.891324 | -1.882726 |
| C | 2.421411 | -0.783606 | 3.639754 |
| C | 4.828526 | -0.719914 | 2.956331 |
| H | 3.190431 | 0.144969 | 1.872016 |
| H | 2.524390 | 0.140055 | 4.230969 |
| H | 1.378101 | -0.873768 | 3.313308 |
| H | 2.629439 | -1.627979 | 4.315717 |
| H | 4.993192 | 0.194476 | 3.548078 |
| H | 5.054706 | -1.576318 | 3.610798 |
| H | 5.564601 | -0.729678 | 2.141031 |
| H | 5.862414 | 0.502746 | -2.886423 |
| H | 4.187154 | -0.052092 | -3.110034 |
| H | 4.514264 | 1.651872 | -2.717279 |
| H | 6.373334 | -1.151838 | -1.268498 |
| H | 5.394472 | -1.500533 | 0.167242 |
| H | 4.776878 | -1.891053 | -1.445262 |
| H | 6.752845 | 1.000280 | -0.501095 |
| H | 5.538295 | 2.278820 | -0.483113 |
| H | 5.602248 | 1.102438 | 0.847998 |
| H | 2.413904 | 5.748957 | -0.728203 |
| H | 3.332555 | 4.358180 | -1.321807 |
| H | 1.657815 | 4.605088 | -1.858900 |
| H | 0.854420 | 5.647217 | 1.010241 |
| H | -0.236649 | 4.710611 | -0.018447 |
| H | 0.124094 | 4.158277 | 1.623605 |
| H | 2.987305 | 4.998251 | 1.673443 |
| H | 2.506197 | 3.383870 | 2.240839 |
| H | 3.825879 | 3.554884 | 1.064957 |
| H | -0.497260 | -0.989952 | -0.283684 |
| H | -1.933872 | -1.463157 | 0.000934 |

153

A-I2 AlH+Ammonium+Imin

| | | | |
|---|---|---|---|
| Al | -1.738483 | -0.236773 | 0.806543 |
| N | -3.022634 | -1.500440 | 0.111550 |
| N | -2.712500 | 1.445048 | 0.600886 |
| C | -4.022328 | 1.330368 | 0.857643 |
| C | -1.986722 | 2.670547 | 0.372172 |
| C | -4.258375 | -1.247163 | 0.597326 |

| | | | |
|---|---|---|---|
| C | -2.654170 | -2.584712 | -0.758852 |
| C | -5.352488 | -2.326240 | 0.873975 |
| C | -4.617078 | 0.047251 | 1.021259 |
| C | -5.049955 | 2.494690 | 1.033308 |
| C | -5.594411 | 2.440302 | 2.479925 |
| C | -4.547801 | 3.927388 | 0.798261 |
| C | -6.215142 | 2.274913 | 0.041703 |
| C | -5.344896 | -2.532897 | 2.408292 |
| C | -6.752730 | -1.845243 | 0.439785 |
| C | -5.133733 | -3.704112 | 0.234851 |
| H | -5.614943 | 0.095181 | 1.439034 |
| C | -1.374588 | -0.575773 | 2.717919 |
| H | -0.570270 | 0.051029 | 3.128987 |
| H | -2.279844 | -0.374694 | 3.316663 |
| H | -1.087601 | -1.616212 | 2.927676 |
| C | -1.838107 | -3.646807 | -0.283938 |
| C | -1.413420 | -4.631155 | -1.184384 |
| C | -1.790682 | -4.608623 | -2.522015 |
| C | -3.040200 | -2.552217 | -2.125268 |
| C | -2.604575 | -3.578036 | -2.975130 |
| C | -1.445329 | -3.805058 | 1.179030 |
| H | -0.792318 | -5.452947 | -0.819080 |
| H | -1.463201 | -5.397064 | -3.204206 |
| C | -3.901076 | -1.453080 | -2.735750 |
| H | -2.912696 | -3.565453 | -4.023640 |
| C | -1.387194 | 3.361619 | 1.461349 |
| C | -0.708017 | 4.559931 | 1.206262 |
| C | -0.596536 | 5.081160 | -0.076302 |
| C | -1.814259 | 3.157781 | -0.949025 |
| C | -1.135577 | 4.369068 | -1.140344 |
| C | -1.439216 | 2.891166 | 2.909950 |
| H | -0.267988 | 5.107105 | 2.043387 |
| H | -0.088212 | 6.033658 | -0.245285 |
| C | -2.334951 | 2.446487 | -2.191134 |
| H | -1.038949 | 4.770130 | -2.152506 |
| N | 1.895398 | 0.183461 | -1.280647 |
| C | 2.768739 | 0.779130 | -0.204214 |
| H | 2.088332 | 1.001981 | 0.631345 |
| C | 3.486217 | 2.027204 | -0.648126 |
| C | 4.884940 | 2.070394 | -0.701732 |
| C | 5.539879 | 3.235819 | -1.102721 |
| C | 4.804276 | 4.366914 | -1.457880 |
| C | 2.753293 | 3.176124 | -0.984338 |
| C | 3.409600 | 4.335703 | -1.396261 |
| H | 5.471506 | 1.190160 | -0.424686 |
| H | 6.631457 | 3.255229 | -1.138642 |
| H | 5.317984 | 5.277897 | -1.774549 |
| H | 1.660811 | 3.186157 | -0.901575 |
| H | 2.828641 | 5.224335 | -1.654037 |
| C | 2.421098 | -0.961280 | -2.149157 |
| C | 1.273986 | -1.309422 | -3.099668 |
| C | 3.642170 | -0.477461 | -2.925426 |

| | | | |
|---|---|---|---|
| C | 2.754376 | -2.149954 | -1.252197 |
| H | 1.579824 | -2.141536 | -3.748922 |
| H | 0.370989 | -1.627297 | -2.556215 |
| H | 1.014693 | -0.461538 | -3.753314 |
| H | 3.957398 | -1.271583 | -3.617439 |
| H | 3.418702 | 0.417718 | -3.526516 |
| H | 4.493947 | -0.251879 | -2.270199 |
| H | 3.066058 | -2.990918 | -1.887841 |
| H | 3.580265 | -1.948251 | -0.553559 |
| H | 1.876046 | -2.480343 | -0.679054 |
| H | 1.624476 | 0.948092 | -1.911767 |
| H | 3.469212 | 0.007888 | 0.138676 |
| C | -0.025160 | 2.605741 | 3.437014 |
| C | -2.137835 | 3.890355 | 3.843394 |
| H | -2.008373 | 1.952005 | 2.941459 |
| H | -0.067528 | 2.154861 | 4.440608 |
| H | 0.526919 | 1.917468 | 2.779767 |
| H | 0.566242 | 3.531550 | 3.517035 |
| H | -2.195296 | 3.479802 | 4.863611 |
| H | -1.587704 | 4.842457 | 3.905518 |
| H | -3.162106 | 4.122617 | 3.520702 |
| C | -1.178617 | 2.130918 | -3.148935 |
| H | -2.780907 | 1.491852 | -1.873280 |
| C | -3.416215 | 3.252505 | -2.923430 |
| H | -1.542166 | 1.639118 | -4.062284 |
| H | -0.646496 | 3.043066 | -3.459667 |
| H | -0.449570 | 1.457271 | -2.672754 |
| H | -3.793000 | 2.693473 | -3.793963 |
| H | -4.274396 | 3.476132 | -2.274570 |
| H | -3.024164 | 4.212642 | -3.294425 |
| C | 0.051913 | -3.554681 | 1.393200 |
| H | -1.999298 | -3.045168 | 1.748722 |
| C | -1.830682 | -5.175004 | 1.755597 |
| H | -1.630133 | -5.201076 | 2.838163 |
| H | -2.896150 | -5.403592 | 1.608457 |
| H | -1.251383 | -5.992145 | 1.298185 |
| H | 0.318142 | -3.641608 | 2.458356 |
| H | 0.657729 | -4.290293 | 0.839932 |
| H | 0.348330 | -2.549240 | 1.058118 |
| C | -3.165003 | -0.762944 | -3.889445 |
| C | -5.259709 | -1.968201 | -3.229965 |
| H | -4.088427 | -0.697899 | -1.957543 |
| H | -3.729577 | 0.113469 | -4.244006 |
| H | -2.164129 | -0.430363 | -3.583669 |
| H | -3.041752 | -1.438313 | -4.750638 |
| H | -5.864938 | -1.137727 | -3.626614 |
| H | -5.138464 | -2.702710 | -4.041898 |
| H | -5.839675 | -2.453147 | -2.433305 |
| H | -6.098387 | -3.288530 | 2.681388 |
| H | -4.365823 | -2.893305 | 2.758662 |
| H | -5.579405 | -1.608100 | 2.954431 |
| H | -5.953238 | -4.364026 | 0.558500 |

| | | | |
|---|---|---|---|
| H | -5.143988 | -3.678939 | -0.861131 |
| H | -4.196585 | -4.172722 | 0.548937 |
| H | -7.471865 | -2.669714 | 0.558636 |
| H | -7.133285 | -1.009254 | 1.042618 |
| H | -6.769402 | -1.535935 | -0.615320 |
| H | -6.289524 | 3.279463 | 2.636743 |
| H | -6.145363 | 1.515704 | 2.699725 |
| H | -4.785736 | 2.532786 | 3.219545 |
| H | -5.401993 | 4.608125 | 0.935451 |
| H | -3.769218 | 4.237280 | 1.502165 |
| H | -4.164993 | 4.086801 | -0.215972 |
| H | -6.943147 | 3.094091 | 0.147031 |
| H | -5.858427 | 2.277020 | -0.999429 |
| H | -6.749741 | 1.331049 | 0.207631 |
| H | -0.436931 | -0.185308 | -0.190140 |
| H | 0.984520 | -0.112928 | -0.841041 |
| N | 5.634728 | -1.668198 | 1.712693 |
| C | 6.790070 | -1.139073 | 1.652058 |
| C | 7.469227 | -0.821621 | 0.381071 |
| C | 8.580146 | 0.036004 | 0.389894 |
| C | 9.248694 | 0.346488 | -0.794770 |
| C | 8.824147 | -0.212048 | -2.001431 |
| C | 7.055757 | -1.385063 | -0.837808 |
| C | 7.729869 | -1.083696 | -2.018413 |
| H | 8.923581 | 0.462694 | 1.337068 |
| H | 10.111811 | 1.016466 | -0.774361 |
| H | 9.355800 | 0.017648 | -2.928308 |
| H | 6.217181 | -2.084702 | -0.829353 |
| H | 7.415813 | -1.542326 | -2.959801 |
| C | 5.029182 | -2.043794 | 2.996698 |
| C | 4.808015 | -3.563564 | 2.926852 |
| C | 5.854678 | -1.701655 | 4.242564 |
| C | 3.671922 | -1.330088 | 3.073906 |
| H | 4.274713 | -3.920536 | 3.821354 |
| H | 4.217443 | -3.829252 | 2.037663 |
| H | 5.770005 | -4.094916 | 2.865953 |
| H | 5.315601 | -2.025278 | 5.145013 |
| H | 6.829874 | -2.212979 | 4.245070 |
| H | 6.031083 | -0.618342 | 4.337017 |
| H | 3.129494 | -1.622594 | 3.985768 |
| H | 3.805084 | -0.236891 | 3.094021 |
| H | 3.046407 | -1.591527 | 2.207436 |
| H | 7.375424 | -0.885242 | 2.554680 |

153

A-TS2*

| | | | |
|---|---|---|---|
| Al | 2.131220 | 0.076346 | 0.901408 |
| N | 3.667897 | 1.201026 | 0.540440 |
| N | 2.717167 | -1.551371 | -0.037374 |
| C | 4.019485 | -1.810425 | 0.076888 |
| C | 1.696359 | -2.368176 | -0.632790 |
| C | 4.800038 | 0.531307 | 0.858718 |

| | | | |
|---|---|---|---|
| C | 3.584411 | 2.550778 | 0.070579 |
| C | 6.048548 | 1.176060 | 1.538772 |
| C | 4.877138 | -0.865782 | 0.716000 |
| C | 4.765295 | -3.083849 | -0.425244 |
| C | 5.353399 | -3.827427 | 0.794278 |
| C | 3.936345 | -4.098439 | -1.225571 |
| C | 5.915691 | -2.632995 | -1.353444 |
| C | 6.014735 | 0.689091 | 3.008087 |
| C | 7.367818 | 0.709791 | 0.893470 |
| C | 6.062228 | 2.709030 | 1.586575 |
| H | 5.831998 | -1.282768 | 1.017745 |
| C | 2.030507 | -0.282933 | 2.859835 |
| H | 2.272309 | -1.320909 | 3.135833 |
| H | 2.740332 | 0.347382 | 3.421321 |
| H | 1.029735 | -0.066304 | 3.272010 |
| C | 2.781554 | 3.496268 | 0.765951 |
| C | 2.637203 | 4.786872 | 0.245126 |
| C | 3.262250 | 5.173172 | -0.933697 |
| C | 4.219453 | 2.938036 | -1.143398 |
| C | 4.044573 | 4.246672 | -1.611099 |
| C | 2.095565 | 3.194694 | 2.091614 |
| H | 2.028518 | 5.513500 | 0.789870 |
| H | 3.143720 | 6.188328 | -1.320439 |
| C | 5.062550 | 2.004574 | -2.006152 |
| H | 4.534984 | 4.545211 | -2.541089 |
| C | 1.033742 | -3.357442 | 0.142620 |
| C | 0.016177 | -4.111377 | -0.455315 |
| C | -0.369825 | -3.902239 | -1.774595 |
| C | 1.271990 | -2.111492 | -1.960353 |
| C | 0.250256 | -2.897240 | -2.508255 |
| C | 1.359834 | -3.641312 | 1.603078 |
| H | -0.473303 | -4.894978 | 0.129020 |
| H | -1.142846 | -4.526155 | -2.231984 |
| C | 1.876895 | -1.019258 | -2.827767 |
| H | -0.054011 | -2.722656 | -3.543666 |
| N | -4.818865 | 0.764331 | -0.447979 |
| C | -5.912711 | 1.392133 | -0.697396 |
| C | -6.387052 | 2.783972 | -0.713443 |
| C | -7.787743 | 2.927653 | -0.613718 |
| C | -8.388163 | 4.182432 | -0.616571 |
| C | -7.600454 | 5.325651 | -0.759192 |
| C | -5.611766 | 3.949217 | -0.871709 |
| C | -6.215634 | 5.202534 | -0.898741 |
| H | -8.414136 | 2.035357 | -0.524008 |
| H | -9.472825 | 4.268430 | -0.521121 |
| H | -8.066148 | 6.314012 | -0.775764 |
| H | -4.533710 | 3.886086 | -1.000537 |
| H | -5.598548 | 6.093640 | -1.034237 |
| C | -3.492981 | 1.255142 | 0.014487 |
| C | -2.717194 | 0.024709 | 0.508678 |
| C | -2.719974 | 1.856160 | -1.169472 |
| C | -3.630025 | 2.226636 | 1.193663 |

| | | | |
|---|---|---|---|
| H | -1.723757 | 0.329396 | 0.865979 |
| H | -3.239481 | -0.473723 | 1.339591 |
| H | -2.542891 | -0.698213 | -0.302820 |
| H | -1.718726 | 2.162462 | -0.833713 |
| H | -2.585350 | 1.110132 | -1.966159 |
| H | -3.213616 | 2.730242 | -1.612835 |
| H | -2.627028 | 2.493470 | 1.556157 |
| H | -4.150768 | 3.157861 | 0.943313 |
| H | -4.170220 | 1.745531 | 2.022747 |
| C | 0.193516 | -3.216408 | 2.506249 |
| C | 1.720706 | -5.108772 | 1.869264 |
| H | 2.230687 | -3.028234 | 1.875401 |
| H | 0.459637 | -3.332335 | 3.568678 |
| H | -0.085025 | -2.165851 | 2.342424 |
| H | -0.697279 | -3.837396 | 2.315500 |
| H | 2.028801 | -5.240582 | 2.918415 |
| H | 0.864098 | -5.779675 | 1.696740 |
| H | 2.545894 | -5.459258 | 1.232818 |
| C | 0.828267 | 0.048711 | -3.166884 |
| H | 2.667580 | -0.531775 | -2.238248 |
| C | 2.518686 | -1.576995 | -4.104692 |
| H | 1.274003 | 0.860388 | -3.762814 |
| H | 0.002889 | -0.375709 | -3.761585 |
| H | 0.407934 | 0.490008 | -2.251580 |
| H | 2.991208 | -0.768806 | -4.683424 |
| H | 3.292964 | -2.326794 | -3.883958 |
| H | 1.773359 | -2.055133 | -4.760211 |
| C | 0.570209 | 3.324651 | 1.995303 |
| H | 2.324345 | 2.153912 | 2.360481 |
| C | 2.632335 | 4.066837 | 3.235751 |
| H | 2.167341 | 3.777827 | 4.191785 |
| H | 3.721755 | 3.969931 | 3.351335 |
| H | 2.412322 | 5.133435 | 3.069600 |
| H | 0.097568 | 3.028105 | 2.945771 |
| H | 0.267820 | 4.363418 | 1.785481 |
| H | 0.170659 | 2.683822 | 1.195569 |
| C | 4.367431 | 1.722625 | -3.343757 |
| C | 6.473548 | 2.548847 | -2.271931 |
| H | 5.165923 | 1.044306 | -1.479684 |
| H | 4.929469 | 0.975430 | -3.926634 |
| H | 3.346481 | 1.349472 | -3.194319 |
| H | 4.298979 | 2.635788 | -3.956341 |
| H | 7.079308 | 1.800510 | -2.807393 |
| H | 6.445746 | 3.451538 | -2.902276 |
| H | 7.005191 | 2.813295 | -1.347272 |
| H | 6.876509 | 1.106364 | 3.552994 |
| H | 5.099637 | 1.025619 | 3.518005 |
| H | 6.060763 | -0.406256 | 3.086895 |
| H | 6.925710 | 3.028821 | 2.190426 |
| H | 6.158293 | 3.172753 | 0.598219 |
| H | 5.161638 | 3.117738 | 2.058584 |
| H | 8.210373 | 1.247078 | 1.355657 |

| | | | |
|---|---|---|---|
| H | 7.560496 | -0.362872 | 1.036528 |
| H | 7.391724 | 0.916538 | -0.185371 |
| H | 5.860973 | -4.742891 | 0.452358 |
| H | 6.093315 | -3.230257 | 1.344851 |
| H | 4.567106 | -4.125704 | 1.503237 |
| H | 4.611641 | -4.902384 | -1.557454 |
| H | 3.136379 | -4.563803 | -0.640495 |
| H | 3.482957 | -3.660311 | -2.122836 |
| H | 6.455971 | -3.517782 | -1.725188 |
| H | 5.527824 | -2.086287 | -2.226850 |
| H | 6.641650 | -1.983151 | -0.848019 |
| H | 0.818924 | 0.604044 | 0.145741 |
| N | -5.209245 | -1.823719 | -0.643494 |
| C | -6.266808 | -2.210510 | 0.334070 |
| C | -5.816489 | -2.010296 | 1.759277 |
| C | -6.269397 | -0.922465 | 2.516047 |
| C | -5.841701 | -0.745524 | 3.833063 |
| C | -4.950635 | -1.653204 | 4.406988 |
| C | -4.932139 | -2.924654 | 2.351169 |
| C | -4.497366 | -2.745299 | 3.664036 |
| H | -6.975358 | -0.211114 | 2.078319 |
| H | -6.211589 | 0.101863 | 4.415438 |
| H | -4.617084 | -1.516488 | 5.438408 |
| H | -4.589643 | -3.799294 | 1.788361 |
| H | -3.812257 | -3.467783 | 4.113714 |
| C | -5.358176 | -2.340716 | -2.059302 |
| C | -4.219050 | -1.721766 | -2.874071 |
| C | -5.226264 | -3.867595 | -2.071910 |
| C | -6.711069 | -1.914735 | -2.629159 |
| H | -4.264286 | -2.084267 | -3.910961 |
| H | -4.287360 | -0.624725 | -2.899725 |
| H | -3.232373 | -2.001467 | -2.471786 |
| H | -5.256340 | -4.231885 | -3.108974 |
| H | -4.266087 | -4.190860 | -1.638650 |
| H | -6.041149 | -4.371557 | -1.531921 |
| H | -6.782852 | -2.242389 | -3.676068 |
| H | -7.556368 | -2.367567 | -2.090521 |
| H | -6.831649 | -0.820760 | -2.622813 |
| H | -4.323041 | -2.196810 | -0.288420 |
| H | -5.001526 | -0.583437 | -0.579237 |
| H | -7.162278 | -1.609673 | 0.127522 |
| H | -6.536757 | -3.265369 | 0.174885 |
| H | -6.739023 | 0.707413 | -0.953833 |

153

A-K31 AlHMe+Iminium+Amin

| | | | |
|---|---|---|---|
| Al | -2.115363 | -0.264330 | 0.571322 |
| N | -3.472845 | -1.435584 | -0.160641 |
| N | -3.142625 | 1.411724 | 0.652514 |
| C | -4.433709 | 1.264586 | 0.948626 |
| C | -2.404138 | 2.638072 | 0.511997 |
| C | -4.641618 | -1.278888 | 0.506087 |

| | | | |
|---|---|---|---|
| C | -3.237493 | -2.344161 | -1.246428 |
| C | -5.626195 | -2.441521 | 0.844491 |
| C | -4.992044 | -0.042994 | 1.074056 |
| C | -5.477878 | 2.400673 | 1.175465 |
| C | -6.039019 | 2.287514 | 2.610733 |
| C | -4.988127 | 3.843733 | 0.986796 |
| C | -6.627882 | 2.194594 | 0.163086 |
| C | -5.396041 | -2.740485 | 2.346183 |
| C | -7.099295 | -2.038735 | 0.636466 |
| C | -5.393626 | -3.756701 | 0.090498 |
| H | -5.946901 | -0.055730 | 1.587180 |
| C | -1.660723 | -0.819907 | 2.421562 |
| H | -0.899072 | -0.178947 | 2.890609 |
| H | -2.549448 | -0.790934 | 3.075511 |
| H | -1.266295 | -1.847461 | 2.475056 |
| C | -2.250948 | -3.360329 | -1.127536 |
| C | -1.951274 | -4.156931 | -2.237880 |
| C | -2.610984 | -3.995792 | -3.450050 |
| C | -3.921469 | -2.178869 | -2.482378 |
| C | -3.592060 | -3.018340 | -3.554749 |
| C | -1.530092 | -3.662214 | 0.178630 |
| H | -1.193278 | -4.938743 | -2.141980 |
| H | -2.369676 | -4.633493 | -4.304037 |
| C | -4.995380 | -1.125628 | -2.732302 |
| H | -4.119746 | -2.895280 | -4.503987 |
| C | -1.806792 | 3.244348 | 1.650935 |
| C | -1.078795 | 4.428787 | 1.476003 |
| C | -0.904519 | 5.004199 | 0.223111 |
| C | -2.182474 | 3.186450 | -0.777109 |
| C | -1.442747 | 4.370317 | -0.890397 |
| C | -1.901871 | 2.684042 | 3.065774 |
| H | -0.644795 | 4.916773 | 2.352542 |
| H | -0.350678 | 5.940524 | 0.115188 |
| C | -2.714491 | 2.557249 | -2.056259 |
| H | -1.297138 | 4.811408 | -1.879909 |
| N | 4.196432 | 1.247672 | -0.743310 |
| C | 4.717080 | 2.425051 | -0.858003 |
| C | 6.106755 | 2.751779 | -1.064773 |
| C | 6.432639 | 4.122494 | -1.150911 |
| C | 7.749829 | 4.521110 | -1.345340 |
| C | 8.753226 | 3.555569 | -1.456053 |
| C | 7.128774 | 1.784978 | -1.179212 |
| C | 8.441232 | 2.192661 | -1.373391 |
| H | 5.642908 | 4.873687 | -1.063532 |
| H | 7.996790 | 5.582709 | -1.411121 |
| H | 9.790063 | 3.865368 | -1.609223 |
| H | 6.912075 | 0.714811 | -1.122216 |
| H | 9.229491 | 1.442822 | -1.462848 |
| C | 2.757060 | 0.904526 | -0.513253 |
| C | 2.690939 | 0.191566 | 0.842617 |
| C | 1.892264 | 2.157388 | -0.510531 |
| C | 2.343834 | -0.034450 | -1.651603 |

| | | | |
|---|---|---|---|
| H | 1.663963 | -0.163823 | 1.008265 |
| H | 3.366694 | -0.676979 | 0.891219 |
| H | 2.953467 | 0.879005 | 1.660745 |
| H | 0.847742 | 1.853998 | -0.351763 |
| H | 2.152331 | 2.849637 | 0.304847 |
| H | 1.928893 | 2.693778 | -1.471258 |
| H | 1.291255 | -0.320279 | -1.511398 |
| H | 2.440278 | 0.457627 | -2.630960 |
| H | 2.946067 | -0.955079 | -1.656434 |
| C | -0.506341 | 2.357195 | 3.617917 |
| C | -2.621392 | 3.628146 | 4.039527 |
| H | -2.472548 | 1.745353 | 3.020037 |
| H | -0.584007 | 1.822862 | 4.577614 |
| H | 0.070006 | 1.727264 | 2.924939 |
| H | 0.076324 | 3.274815 | 3.799130 |
| H | -2.704399 | 3.160386 | 5.033231 |
| H | -2.069988 | 4.573244 | 4.167725 |
| H | -3.636543 | 3.883476 | 3.706560 |
| C | -1.560066 | 2.122204 | -2.969376 |
| H | -3.274973 | 1.653512 | -1.773901 |
| C | -3.673282 | 3.489195 | -2.809082 |
| H | -1.942587 | 1.661996 | -3.893466 |
| H | -0.940235 | 2.982704 | -3.269106 |
| H | -0.915582 | 1.386268 | -2.467158 |
| H | -4.077169 | 2.986732 | -3.701102 |
| H | -4.524739 | 3.798563 | -2.185260 |
| H | -3.165462 | 4.404817 | -3.151601 |
| C | -0.044156 | -3.288560 | 0.108542 |
| H | -1.997413 | -3.042983 | 0.957648 |
| C | -1.686278 | -5.125100 | 0.617120 |
| H | -1.241212 | -5.275113 | 1.613700 |
| H | -2.741806 | -5.429252 | 0.673294 |
| H | -1.180332 | -5.817271 | -0.074263 |
| H | 0.449003 | -3.460380 | 1.079080 |
| H | 0.480143 | -3.898242 | -0.645386 |
| H | 0.090130 | -2.231305 | -0.164017 |
| C | -4.562541 | -0.141786 | -3.825769 |
| C | -6.347917 | -1.744973 | -3.112945 |
| H | -5.136361 | -0.550669 | -1.805133 |
| H | -5.295231 | 0.674532 | -3.926797 |
| H | -3.581121 | 0.297466 | -3.606441 |
| H | -4.490036 | -0.638967 | -4.806074 |
| H | -7.118935 | -0.963019 | -3.201256 |
| H | -6.293682 | -2.259582 | -4.085324 |
| H | -6.696952 | -2.479984 | -2.374765 |
| H | -6.051198 | -3.567852 | 2.662598 |
| H | -4.355002 | -3.042540 | 2.536930 |
| H | -5.616535 | -1.870330 | 2.980877 |
| H | -6.093397 | -4.509383 | 0.485762 |
| H | -5.569280 | -3.674165 | -0.988498 |
| H | -4.379919 | -4.145320 | 0.233831 |
| H | -7.744209 | -2.914514 | 0.806642 |

| | | | |
|---|---|---|---|
| H | -7.437569 | -1.259486 | 1.333832 |
| H | -7.284537 | -1.680943 | -0.386136 |
| H | -6.737286 | 3.118838 | 2.793895 |
| H | -6.593534 | 1.355004 | 2.783636 |
| H | -5.240753 | 2.347802 | 3.364639 |
| H | -5.848374 | 4.515177 | 1.133474 |
| H | -4.215993 | 4.139992 | 1.704153 |
| H | -4.596771 | 4.029835 | -0.020063 |
| H | -7.381171 | 2.986760 | 0.297136 |
| H | -6.259845 | 2.254928 | -0.872717 |
| H | -7.131000 | 1.226425 | 0.283657 |
| H | -0.902970 | -0.026663 | -0.461306 |
| N | 5.477521 | -2.664471 | 1.804160 |
| C | 6.790972 | -2.194710 | 1.420664 |
| C | 6.943231 | -2.006888 | -0.074623 |
| C | 8.223890 | -1.846077 | -0.627390 |
| C | 8.398536 | -1.676380 | -2.000954 |
| C | 7.293169 | -1.664422 | -2.856969 |
| C | 5.842133 | -2.000714 | -0.943399 |
| C | 6.015315 | -1.833002 | -2.323337 |
| H | 9.099337 | -1.874975 | 0.028672 |
| H | 9.406852 | -1.571796 | -2.410481 |
| H | 7.431194 | -1.548643 | -3.934664 |
| H | 4.847990 | -2.175833 | -0.524532 |
| H | 5.147863 | -1.861524 | -2.988830 |
| C | 5.194799 | -2.825782 | 3.247344 |
| C | 3.790105 | -3.430352 | 3.340662 |
| C | 6.197090 | -3.762058 | 3.945741 |
| C | 5.201941 | -1.454947 | 3.933097 |
| H | 3.492288 | -3.574606 | 4.389667 |
| H | 3.048822 | -2.777034 | 2.856622 |
| H | 3.749678 | -4.416054 | 2.847889 |
| H | 5.915353 | -3.924295 | 4.997338 |
| H | 6.225842 | -4.748017 | 3.453644 |
| H | 7.218455 | -3.351002 | 3.944238 |
| H | 4.895684 | -1.553985 | 4.985101 |
| H | 6.201638 | -0.993254 | 3.933059 |
| H | 4.501819 | -0.766352 | 3.436491 |
| H | 5.304326 | -3.561801 | 1.349949 |
| H | 4.813638 | 0.428890 | -0.806361 |
| H | 6.973399 | -1.220337 | 1.907804 |
| H | 7.622994 | -2.847351 | 1.759214 |
| H | 4.016291 | 3.262316 | -0.786182 |

153

A-TS3*

| | | | |
|---|---|---|---|
| Al | 1.895933 | 0.243966 | -0.576667 |
| N | 3.575694 | -0.704116 | -0.677514 |
| N | 2.428458 | 1.898297 | 0.275648 |
| C | 3.728161 | 2.237241 | 0.275928 |
| C | 1.342178 | 2.701173 | 0.799407 |
| C | 4.720519 | 0.009164 | -0.617535 |

| | | | |
|---|---|---|---|
| C | 3.471919 | -2.139637 | -0.678055 |
| C | 6.141483 | -0.517029 | -1.003340 |
| C | 4.712944 | 1.366602 | -0.253691 |
| C | 4.353412 | 3.552606 | 0.851347 |
| C | 5.155460 | 4.256750 | -0.269347 |
| C | 3.403117 | 4.606917 | 1.441190 |
| C | 5.324903 | 3.153281 | 1.987276 |
| C | 6.498251 | 0.165578 | -2.345345 |
| C | 7.185150 | -0.114368 | 0.062161 |
| C | 6.301792 | -2.031570 | -1.199493 |
| H | 5.695413 | 1.817046 | -0.290134 |
| C | 0.937625 | 0.480747 | -2.269560 |
| H | 0.026882 | 1.075678 | -2.114138 |
| H | 1.550223 | 1.005415 | -3.021956 |
| H | 0.608569 | -0.462377 | -2.726222 |
| C | 3.081526 | -2.844956 | -1.850830 |
| C | 2.878964 | -4.230265 | -1.758435 |
| C | 3.051471 | -4.920852 | -0.563702 |
| C | 3.663900 | -2.838459 | 0.545657 |
| C | 3.445631 | -4.222165 | 0.574239 |
| C | 2.923829 | -2.199464 | -3.226376 |
| H | 2.595525 | -4.785566 | -2.655432 |
| H | 2.900463 | -6.002598 | -0.524534 |
| C | 4.097158 | -2.154560 | 1.837650 |
| H | 3.606505 | -4.768150 | 1.507337 |
| C | 0.637662 | 3.591889 | -0.056827 |
| C | -0.418159 | 4.336383 | 0.485645 |
| C | -0.799722 | 4.210870 | 1.815208 |
| C | 0.942425 | 2.542867 | 2.152139 |
| C | -0.125829 | 3.311422 | 2.631325 |
| C | 0.964250 | 3.821024 | -1.530956 |
| H | -0.953425 | 5.039521 | -0.156198 |
| H | -1.621443 | 4.811001 | 2.213556 |
| C | 1.621303 | 1.596614 | 3.136467 |
| H | -0.428238 | 3.210749 | 3.676604 |
| N | -0.067003 | -3.131339 | 0.672631 |
| C | -0.150569 | -2.117719 | -0.171191 |
| C | -1.396278 | -1.361817 | -0.471084 |
| C | -1.876843 | -0.295025 | 0.296824 |
| C | -3.063145 | 0.346764 | -0.059866 |
| C | -3.779916 | -0.066225 | -1.184403 |
| C | -2.106128 | -1.759100 | -1.616835 |
| C | -3.295185 | -1.119725 | -1.962658 |
| H | -1.304934 | 0.057187 | 1.156912 |
| H | -3.424135 | 1.185063 | 0.539944 |
| H | -4.706177 | 0.442736 | -1.462493 |
| H | -1.734642 | -2.580992 | -2.234955 |
| H | -3.843161 | -1.439916 | -2.851824 |
| C | -0.995282 | -3.657696 | 1.720365 |
| C | -0.349818 | -4.963496 | 2.198804 |
| C | -1.123278 | -2.683253 | 2.892985 |
| C | -2.370501 | -3.963332 | 1.115451 |

| | | | |
|---|---|---|---|
| H | -0.973923 | -5.425119 | 2.976162 |
| H | -0.250813 | -5.687697 | 1.375118 |
| H | 0.646061 | -4.784427 | 2.633158 |
| H | -1.753280 | -3.135484 | 3.673112 |
| H | -0.143825 | -2.458274 | 3.336542 |
| H | -1.599275 | -1.742935 | 2.586438 |
| H | -2.982984 | -4.480970 | 1.868417 |
| H | -2.906681 | -3.053846 | 0.816131 |
| H | -2.282445 | -4.623329 | 0.239473 |
| C | -0.288708 | 3.719251 | -2.415808 |
| C | 1.631034 | 5.180180 | -1.792850 |
| H | 1.670594 | 3.038810 | -1.844534 |
| H | -0.002488 | 3.647060 | -3.476324 |
| H | -0.911334 | 2.847672 | -2.170965 |
| H | -0.922889 | 4.613950 | -2.315362 |
| H | 1.810298 | 5.311707 | -2.871549 |
| H | 0.986338 | 6.009856 | -1.461786 |
| H | 2.596820 | 5.287787 | -1.283669 |
| C | 0.634368 | 0.536964 | 3.637153 |
| H | 2.434343 | 1.077488 | 2.604730 |
| C | 2.237038 | 2.328395 | 4.338028 |
| H | 1.123952 | -0.156396 | 4.337554 |
| H | -0.211324 | 0.997001 | 4.171696 |
| H | 0.224818 | -0.051396 | 2.804956 |
| H | 2.771730 | 1.616597 | 4.986476 |
| H | 2.951256 | 3.107172 | 4.038082 |
| H | 1.463108 | 2.812232 | 4.954139 |
| C | 1.605238 | -2.590627 | -3.909021 |
| H | 2.927892 | -1.108821 | -3.085408 |
| C | 4.080564 | -2.551061 | -4.176308 |
| H | 3.909190 | -2.092932 | -5.162838 |
| H | 5.053682 | -2.195101 | -3.815135 |
| H | 4.157582 | -3.639812 | -4.325337 |
| H | 1.439630 | -1.970329 | -4.802728 |
| H | 1.617036 | -3.639646 | -4.243669 |
| H | 0.731141 | -2.464919 | -3.253282 |
| C | 2.942702 | -2.108446 | 2.843218 |
| C | 5.326825 | -2.807992 | 2.482240 |
| H | 4.362039 | -1.114488 | 1.598891 |
| H | 3.232360 | -1.544244 | 3.743079 |
| H | 2.054021 | -1.627791 | 2.410979 |
| H | 2.661659 | -3.122766 | 3.170586 |
| H | 5.653394 | -2.220276 | 3.354180 |
| H | 5.110006 | -3.825470 | 2.843359 |
| H | 6.177688 | -2.877810 | 1.789761 |
| H | 7.495654 | -0.165885 | -2.674129 |
| H | 5.778873 | -0.098192 | -3.134836 |
| H | 6.517659 | 1.261429 | -2.263911 |
| H | 7.333499 | -2.221321 | -1.532794 |
| H | 6.150139 | -2.598265 | -0.273754 |
| H | 5.635574 | -2.448264 | -1.958817 |
| H | 8.152423 | -0.574768 | -0.189518 |

| | | | |
|---|---|---|---|
| H | 7.358609 | 0.968370 | 0.121665 |
| H | 6.898777 | -0.467513 | 1.064048 |
| H | 5.536443 | 5.216591 | 0.110804 |
| H | 6.025062 | 3.680377 | -0.612667 |
| H | 4.528975 | 4.469937 | -1.147436 |
| H | 4.018227 | 5.451115 | 1.788510 |
| H | 2.686677 | 5.003791 | 0.715897 |
| H | 2.836342 | 4.242732 | 2.303507 |
| H | 5.767573 | 4.062296 | 2.423028 |
| H | 4.801794 | 2.618139 | 2.793314 |
| H | 6.148079 | 2.514533 | 1.641310 |
| H | 0.874313 | -0.763195 | 0.364394 |
| N | -8.502535 | -0.310093 | 0.569685 |
| C | -8.783046 | -0.123382 | -0.836959 |
| C | -7.827750 | 0.854623 | -1.488956 |
| C | -7.485462 | 0.721227 | -2.841755 |
| C | -6.658551 | 1.655906 | -3.468499 |
| C | -6.152394 | 2.738775 | -2.746498 |
| C | -7.307541 | 1.940942 | -0.771550 |
| C | -6.478738 | 2.875525 | -1.394933 |
| H | -7.880559 | -0.122961 | -3.415678 |
| H | -6.411512 | 1.539458 | -4.527226 |
| H | -5.509762 | 3.474769 | -3.236657 |
| H | -7.569821 | 2.036185 | 0.284660 |
| H | -6.092612 | 3.724109 | -0.823087 |
| C | -9.471249 | -1.045620 | 1.398354 |
| C | -8.830670 | -1.176369 | 2.784389 |
| C | -10.764729 | -0.230655 | 1.520816 |
| C | -9.787130 | -2.448195 | 0.846736 |
| H | -9.504069 | -1.690823 | 3.486065 |
| H | -7.895538 | -1.760083 | 2.733557 |
| H | -8.591171 | -0.183109 | 3.193022 |
| H | -11.461367 | -0.716950 | 2.220363 |
| H | -10.547453 | 0.782041 | 1.892176 |
| H | -11.289121 | -0.137047 | 0.557393 |
| H | -10.464021 | -2.997680 | 1.519526 |
| H | -10.276328 | -2.398340 | -0.138503 |
| H | -8.866032 | -3.044905 | 0.738104 |
| H | -8.785118 | -1.060265 | -1.434432 |
| H | -9.803113 | 0.287991 | -0.935552 |
| H | 0.561261 | -2.202743 | -0.997055 |
| H | -7.580470 | -0.729819 | 0.678760 |
| H | 0.802791 | -3.660095 | 0.592524 |

153

A-K33 AlMe+Amin+Amin

| | | | |
|---|---|---|---|
| Al | -3.042751 | -0.108916 | -0.796561 |
| N | -2.944084 | -1.893947 | -0.307274 |
| N | -3.828657 | 0.692307 | 0.676602 |
| C | -4.140734 | -0.012571 | 1.785823 |
| C | -3.903479 | 2.123093 | 0.458389 |
| C | -3.356705 | -2.315024 | 0.910436 |

| | | | |
|---|---|---|---|
| C | -2.472446 | -2.694913 | -1.419805 |
| C | -3.254472 | -3.772949 | 1.445875 |
| C | -3.895980 | -1.399983 | 1.836204 |
| C | -4.818695 | 0.563443 | 3.061798 |
| C | -3.998580 | 0.161536 | 4.308823 |
| C | -4.992292 | 2.087815 | 3.128986 |
| C | -6.228002 | -0.068444 | 3.149007 |
| C | -2.252267 | -3.752799 | 2.624379 |
| C | -4.639859 | -4.231940 | 1.954614 |
| C | -2.774333 | -4.848578 | 0.460793 |
| H | -4.186334 | -1.855682 | 2.774672 |
| C | -2.535291 | 0.691940 | -2.478244 |
| H | -3.370506 | 1.258306 | -2.917460 |
| H | -1.706520 | 1.403543 | -2.339005 |
| H | -2.219703 | -0.075540 | -3.200875 |
| C | -1.086024 | -2.786887 | -1.677652 |
| C | -0.680178 | -3.456904 | -2.840121 |
| C | -1.603584 | -4.005321 | -3.721619 |
| C | -3.430177 | -3.241680 | -2.308920 |
| C | -2.965942 | -3.895760 | -3.454014 |
| C | -0.020411 | -2.207919 | -0.754960 |
| H | 0.386474 | -3.550180 | -3.057696 |
| H | -1.262716 | -4.521675 | -4.622080 |
| C | -4.927632 | -3.115285 | -2.054609 |
| H | -3.682753 | -4.332493 | -4.151875 |
| C | -2.795779 | 2.915102 | 0.850617 |
| C | -2.801200 | 4.268364 | 0.501426 |
| C | -3.861041 | 4.826282 | -0.209731 |
| C | -4.986231 | 2.667773 | -0.269052 |
| C | -4.938752 | 4.033190 | -0.583066 |
| C | -1.630278 | 2.321699 | 1.635437 |
| H | -1.961584 | 4.901556 | 0.791240 |
| H | -3.845488 | 5.887053 | -0.470817 |
| C | -6.205173 | 1.857904 | -0.696676 |
| H | -5.767717 | 4.483938 | -1.133914 |
| N | 4.984506 | 2.404685 | -0.627559 |
| C | 4.039691 | 3.277860 | 0.035941 |
| H | 3.629245 | 2.727560 | 0.901480 |
| C | 2.861471 | 3.714832 | -0.819750 |
| C | 2.168638 | 4.896913 | -0.524597 |
| C | 1.060797 | 5.287323 | -1.279796 |
| C | 0.628230 | 4.499267 | -2.349057 |
| C | 2.419566 | 2.932134 | -1.892696 |
| C | 1.314404 | 3.320619 | -2.652947 |
| H | 2.512956 | 5.531962 | 0.298309 |
| H | 0.546839 | 6.224075 | -1.046689 |
| H | -0.226358 | 4.813245 | -2.954147 |
| H | 2.983673 | 2.026847 | -2.130413 |
| H | 0.997134 | 2.708401 | -3.502323 |
| C | 6.196873 | 2.984521 | -1.234523 |
| C | 6.927971 | 1.826692 | -1.922455 |
| C | 5.810595 | 4.035874 | -2.279656 |

| | | | |
|---|---|---|---|
| C | 7.128389 | 3.617220 | -0.183392 |
| H | 7.853876 | 2.173582 | -2.405816 |
| H | 7.205962 | 1.047197 | -1.193773 |
| H | 6.284882 | 1.366503 | -2.687738 |
| H | 6.712765 | 4.430874 | -2.771103 |
| H | 5.157246 | 3.603722 | -3.051705 |
| H | 5.281406 | 4.889839 | -1.829530 |
| H | 8.040043 | 4.021762 | -0.650139 |
| H | 6.640566 | 4.449185 | 0.348742 |
| H | 7.439160 | 2.869634 | 0.564384 |
| H | 4.506432 | 4.183619 | 0.473705 |
| C | -0.581710 | 1.695523 | 0.710347 |
| C | -0.960844 | 3.319201 | 2.584851 |
| H | -2.037220 | 1.510781 | 2.261325 |
| H | 0.247563 | 1.261083 | 1.288952 |
| H | -0.994044 | 0.868556 | 0.099976 |
| H | -0.163514 | 2.431218 | 0.006612 |
| H | -0.243220 | 2.796007 | 3.234928 |
| H | -0.395606 | 4.090564 | 2.040201 |
| H | -1.693832 | 3.825794 | 3.230828 |
| C | -6.419191 | 1.890485 | -2.215849 |
| H | -6.033523 | 0.808427 | -0.403054 |
| C | -7.481173 | 2.326754 | 0.018466 |
| H | -7.261703 | 1.241706 | -2.500427 |
| H | -6.657532 | 2.906122 | -2.567361 |
| H | -5.531636 | 1.553290 | -2.770643 |
| H | -8.334396 | 1.688444 | -0.259034 |
| H | -7.382301 | 2.298345 | 1.112908 |
| H | -7.738082 | 3.360056 | -0.262136 |
| C | 0.813774 | -1.129140 | -1.458992 |
| H | -0.533789 | -1.733733 | 0.098336 |
| C | 0.897852 | -3.294977 | -0.178690 |
| H | 1.619745 | -2.852028 | 0.524621 |
| H | 0.336498 | -4.073744 | 0.357931 |
| H | 1.476591 | -3.792781 | -0.972190 |
| H | 1.531086 | -0.674076 | -0.759339 |
| H | 1.394098 | -1.554093 | -2.292692 |
| H | 0.190433 | -0.323898 | -1.874650 |
| C | -5.493266 | -1.862121 | -2.733305 |
| C | -5.727791 | -4.353765 | -2.468910 |
| H | -5.071831 | -2.991017 | -0.969654 |
| H | -6.566467 | -1.743402 | -2.518800 |
| H | -4.989606 | -0.938211 | -2.397745 |
| H | -5.364547 | -1.908462 | -3.825890 |
| H | -6.771913 | -4.259054 | -2.133633 |
| H | -5.754098 | -4.485654 | -3.561405 |
| H | -5.314932 | -5.274528 | -2.029940 |
| H | -2.152758 | -4.769848 | 3.033402 |
| H | -1.254245 | -3.425850 | 2.295686 |
| H | -2.570898 | -3.093859 | 3.443904 |
| H | -2.732556 | -5.804237 | 1.004804 |
| H | -3.455908 | -4.987332 | -0.386736 |

| | | | |
|---|---|---|---|
| H | -1.772164 | -4.656722 | 0.063114 |
| H | -4.565093 | -5.271579 | 2.306879 |
| H | -5.020362 | -3.634630 | 2.794082 |
| H | -5.392348 | -4.207893 | 1.151699 |
| H | -4.466567 | 0.603250 | 5.201157 |
| H | -3.952652 | -0.923159 | 4.474881 |
| H | -2.966920 | 0.541401 | 4.253906 |
| H | -5.507394 | 2.324481 | 4.072153 |
| H | -4.036882 | 2.626201 | 3.138042 |
| H | -5.602664 | 2.492761 | 2.315166 |
| H | -6.743090 | 0.313743 | 4.043585 |
| H | -6.840639 | 0.193293 | 2.273456 |
| H | -6.197337 | -1.164360 | 3.224249 |
| H | 5.257805 | 1.650515 | -0.002999 |
| N | 7.818172 | -1.960039 | 0.317784 |
| C | 7.060312 | -2.580726 | 1.382702 |
| C | 6.089818 | -1.621342 | 2.039585 |
| C | 4.854799 | -2.073019 | 2.522541 |
| C | 3.984799 | -1.208175 | 3.190295 |
| C | 4.335159 | 0.129593 | 3.379554 |
| C | 6.429114 | -0.274546 | 2.230112 |
| C | 5.561435 | 0.593204 | 2.895346 |
| H | 4.575521 | -3.122136 | 2.382436 |
| H | 3.031717 | -1.584985 | 3.572258 |
| H | 3.662286 | 0.806746 | 3.912631 |
| H | 7.391495 | 0.075738 | 1.849564 |
| H | 5.851053 | 1.636197 | 3.050938 |
| C | 8.991888 | -2.670055 | -0.220249 |
| C | 9.486558 | -1.840700 | -1.409647 |
| C | 10.093449 | -2.719643 | 0.845813 |
| C | 8.659180 | -4.096287 | -0.695788 |
| H | 10.381807 | -2.293023 | -1.861689 |
| H | 8.715187 | -1.775030 | -2.195427 |
| H | 9.736176 | -0.817686 | -1.090698 |
| H | 11.012215 | -3.159274 | 0.429051 |
| H | 10.324956 | -1.706281 | 1.206581 |
| H | 9.807075 | -3.335130 | 1.712696 |
| H | 9.539190 | -4.579925 | -1.147758 |
| H | 8.326091 | -4.736938 | 0.135728 |
| H | 7.859608 | -4.081326 | -1.455025 |
| H | 7.192180 | -1.717568 | -0.448756 |
| H | 6.500278 | -3.490602 | 1.077881 |
| H | 7.769341 | -2.920831 | 2.157774 |

124

A-K3 koordiniere Amin

| | | | |
|---|---|---|---|
| Al | 0.150956 | -0.217877 | -0.738324 |
| N | 2.037370 | -0.134385 | -0.319834 |
| N | -0.495792 | 1.520747 | -0.131734 |
| C | 0.390221 | 2.504063 | -0.377431 |
| C | -1.860669 | 1.710262 | 0.310414 |
| C | 2.536527 | 1.066905 | -0.709074 |

| | | | |
|---|---|---|---|
| C | 2.846886 | -1.220931 | 0.177149 |
| C | 3.991414 | 1.316688 | -1.247548 |
| C | 1.708111 | 2.198616 | -0.805089 |
| C | 0.140131 | 4.049333 | -0.306119 |
| C | 0.207214 | 4.593689 | -1.753906 |
| C | -1.175805 | 4.532709 | 0.325428 |
| C | 1.270383 | 4.723692 | 0.508921 |
| C | 3.871917 | 1.206086 | -2.787302 |
| C | 4.503328 | 2.739199 | -0.922903 |
| C | 5.099316 | 0.358261 | -0.778297 |
| H | 2.230456 | 3.067986 | -1.177497 |
| C | -0.008644 | -0.395417 | -2.690039 |
| H | -0.956272 | -0.030145 | -3.111460 |
| H | 0.788533 | 0.218530 | -3.141781 |
| H | 0.127490 | -1.420339 | -3.066603 |
| C | 3.170908 | -2.331875 | -0.647583 |
| C | 3.895563 | -3.389126 | -0.079918 |
| C | 4.326933 | -3.354298 | 1.239961 |
| C | 3.259216 | -1.184961 | 1.538903 |
| C | 4.015340 | -2.254810 | 2.032454 |
| C | 2.881329 | -2.402093 | -2.147119 |
| H | 4.155588 | -4.249190 | -0.701884 |
| H | 4.908633 | -4.182435 | 1.651893 |
| C | 2.880302 | -0.050530 | 2.492455 |
| H | 4.354920 | -2.236756 | 3.069592 |
| C | -2.903829 | 1.957817 | -0.629208 |
| C | -4.207562 | 2.124229 | -0.141307 |
| C | -4.502426 | 2.051156 | 1.213312 |
| C | -2.158618 | 1.590534 | 1.697813 |
| C | -3.483014 | 1.776419 | 2.116945 |
| C | -2.714715 | 2.120110 | -2.138308 |
| H | -5.013602 | 2.331056 | -0.848797 |
| H | -5.525522 | 2.202903 | 1.565382 |
| C | -1.096970 | 1.311030 | 2.759627 |
| H | -3.721384 | 1.713231 | 3.179670 |
| N | -1.242624 | -1.561441 | 0.162595 |
| C | -2.128296 | -1.930699 | -0.992764 |
| H | -2.197779 | -1.026116 | -1.609303 |
| C | -3.530315 | -2.417070 | -0.718004 |
| C | -3.953742 | -3.651377 | -1.227734 |
| C | -5.264315 | -4.091735 | -1.034440 |
| C | -6.169168 | -3.300105 | -0.327037 |
| C | -4.454742 | -1.617679 | -0.031235 |
| C | -5.761279 | -2.060296 | 0.170707 |
| H | -3.252432 | -4.278906 | -1.785806 |
| H | -5.578032 | -5.057251 | -1.438535 |
| H | -7.194503 | -3.643950 | -0.171044 |
| H | -4.161899 | -0.634275 | 0.348154 |
| H | -6.469730 | -1.428750 | 0.712364 |
| C | -0.921990 | -2.710410 | 1.133106 |
| C | 0.083718 | -2.181740 | 2.142631 |
| C | -2.174605 | -3.164167 | 1.897098 |

| | | | |
|---|---|---|---|
| C | -0.312256 | -3.890303 | 0.385851 |
| H | 0.354439 | -2.984394 | 2.842882 |
| H | 1.010791 | -1.856024 | 1.656998 |
| H | -0.329211 | -1.356694 | 2.734986 |
| H | -1.857229 | -3.825891 | 2.716587 |
| H | -2.710433 | -2.315788 | 2.349254 |
| H | -2.884400 | -3.721361 | 1.276038 |
| H | -0.079137 | -4.681976 | 1.112136 |
| H | -0.995164 | -4.329043 | -0.354860 |
| H | 0.630753 | -3.616230 | -0.105100 |
| H | -1.779726 | -0.889177 | 0.728302 |
| H | -1.586622 | -2.662025 | -1.604527 |
| C | -3.493508 | 1.068349 | -2.943561 |
| C | -3.157782 | 3.504646 | -2.643797 |
| H | -1.643667 | 2.000515 | -2.362161 |
| H | -3.266498 | 1.166105 | -4.016247 |
| H | -3.269010 | 0.035520 | -2.648957 |
| H | -4.579976 | 1.202587 | -2.827431 |
| H | -2.918806 | 3.607251 | -3.713661 |
| H | -4.246257 | 3.634853 | -2.542758 |
| H | -2.677594 | 4.336851 | -2.114762 |
| C | -1.631189 | 0.485565 | 3.937023 |
| H | -0.293512 | 0.734271 | 2.271604 |
| C | -0.447782 | 2.587107 | 3.310026 |
| H | -0.796797 | 0.131133 | 4.560598 |
| H | -2.278451 | 1.086271 | 4.594063 |
| H | -2.213227 | -0.391088 | 3.616369 |
| H | 0.262748 | 2.336769 | 4.112897 |
| H | 0.106244 | 3.135444 | 2.539774 |
| H | -1.205056 | 3.264844 | 3.734916 |
| C | 1.912059 | -3.525425 | -2.535191 |
| H | 2.417505 | -1.448713 | -2.437248 |
| C | 4.169233 | -2.564728 | -2.972757 |
| H | 3.943216 | -2.475690 | -4.046777 |
| H | 4.930636 | -1.811857 | -2.728611 |
| H | 4.623959 | -3.555405 | -2.818099 |
| H | 1.789368 | -3.559882 | -3.628844 |
| H | 2.285788 | -4.511040 | -2.215612 |
| H | 0.914171 | -3.391846 | -2.097888 |
| C | 2.608944 | -0.533688 | 3.923492 |
| C | 3.922520 | 1.073630 | 2.547708 |
| H | 1.948549 | 0.392656 | 2.103208 |
| H | 2.152377 | 0.276769 | 4.512119 |
| H | 1.937690 | -1.402480 | 3.961501 |
| H | 3.539000 | -0.810037 | 4.443901 |
| H | 3.626406 | 1.831593 | 3.289755 |
| H | 4.909403 | 0.684815 | 2.845382 |
| H | 4.035170 | 1.584632 | 1.584712 |
| H | 4.854976 | 1.399376 | -3.244545 |
| H | 3.548135 | 0.206401 | -3.105249 |
| H | 3.160683 | 1.940621 | -3.193056 |
| H | 6.035727 | 0.661221 | -1.270798 |

| | | | |
|---|---|---|---|
| H | 5.270182 | 0.405858 | 0.303664 |
| H | 4.919425 | -0.685615 | -1.040867 |
| H | 5.562924 | 2.804936 | -1.209288 |
| H | 3.988432 | 3.532230 | -1.482951 |
| H | 4.437548 | 2.976991 | 0.148714 |
| H | -0.032052 | 5.668159 | -1.747057 |
| H | 1.206107 | 4.484408 | -2.198692 |
| H | -0.510238 | 4.095120 | -2.418391 |
| H | -1.155890 | 5.632970 | 0.336403 |
| H | -2.069251 | 4.232033 | -0.226151 |
| H | -1.296805 | 4.202873 | 1.362936 |
| H | 1.076641 | 5.805922 | 0.554001 |
| H | 1.310289 | 4.353374 | 1.543358 |
| H | 2.266390 | 4.589450 | 0.070086 |

124

A-Product non koordinirend

| | | | |
|---|---|---|---|
| Al | -1.069957 | 0.045834 | 0.322786 |
| N | -1.974551 | 1.590364 | -0.155493 |
| N | -2.344710 | -1.280016 | 0.100207 |
| C | -3.593154 | -0.995923 | -0.333761 |
| C | -1.757742 | -2.579245 | 0.360173 |
| C | -3.263728 | 1.557844 | -0.563069 |
| C | -1.127976 | 2.744889 | 0.073409 |
| C | -4.093883 | 2.784987 | -1.038643 |
| C | -3.958465 | 0.333637 | -0.625684 |
| C | -4.745033 | -2.026405 | -0.514991 |
| C | -5.331375 | -1.895296 | -1.939263 |
| C | -4.393376 | -3.509129 | -0.324600 |
| C | -5.835199 | -1.675615 | 0.525083 |
| C | -4.390304 | 2.574576 | -2.542495 |
| C | -5.425319 | 2.841643 | -0.255608 |
| C | -3.442994 | 4.167705 | -0.889913 |
| H | -4.981415 | 0.435070 | -0.966266 |
| C | 0.738757 | -0.135498 | 0.971147 |
| H | 0.976983 | -1.190173 | 1.175050 |
| H | 1.468033 | 0.235357 | 0.234924 |
| H | 0.891357 | 0.445338 | 1.893863 |
| C | -0.163805 | 3.108343 | -0.895117 |
| C | 0.733247 | 4.135355 | -0.570229 |
| C | 0.691185 | 4.772589 | 0.663450 |
| C | -1.190144 | 3.385246 | 1.336175 |
| C | -0.265321 | 4.398800 | 1.604159 |
| C | -0.075031 | 2.468091 | -2.276074 |
| H | 1.481651 | 4.442633 | -1.304486 |
| H | 1.403170 | 5.568569 | 0.893938 |
| C | -2.226156 | 2.997860 | 2.386313 |
| H | -0.295016 | 4.913322 | 2.566351 |
| C | -1.159045 | -3.271689 | -0.720759 |
| C | -0.479400 | -4.461001 | -0.442820 |
| C | -0.384339 | -4.951501 | 0.857480 |
| C | -1.660886 | -3.051861 | 1.688036 |

| | | | |
|---|---|---|---|
| C | -0.969829 | -4.252020 | 1.905334 |
| C | -1.236888 | -2.740219 | -2.147412 |
| H | -0.016620 | -5.020155 | -1.257635 |
| H | 0.149800 | -5.884468 | 1.052053 |
| C | -2.275561 | -2.331488 | 2.881801 |
| H | -0.890017 | -4.646317 | 2.921190 |
| N | 6.900145 | 0.921655 | 0.679007 |
| C | 6.023087 | -0.160430 | 1.077301 |
| C | 5.447437 | -0.969334 | -0.073915 |
| C | 5.064428 | -2.303564 | 0.114864 |
| C | 4.501586 | -3.042393 | -0.927623 |
| C | 4.314807 | -2.455033 | -2.180838 |
| C | 5.252205 | -0.388737 | -1.332884 |
| C | 4.691962 | -1.124109 | -2.378591 |
| H | 5.224858 | -2.777984 | 1.088561 |
| H | 4.226766 | -4.088320 | -0.765400 |
| H | 3.898342 | -3.038679 | -3.006475 |
| H | 5.577814 | 0.644445 | -1.477375 |
| H | 4.564952 | -0.660822 | -3.361287 |
| C | 8.358241 | 0.699396 | 0.661253 |
| C | 8.981589 | 1.985604 | 0.108672 |
| C | 8.919949 | 0.427458 | 2.069354 |
| C | 8.698907 | -0.470769 | -0.266625 |
| H | 10.078236 | 1.906662 | 0.061667 |
| H | 8.601272 | 2.195771 | -0.902047 |
| H | 8.739704 | 2.849963 | 0.749922 |
| H | 10.012561 | 0.293006 | 2.043937 |
| H | 8.702755 | 1.269050 | 2.747729 |
| H | 8.491282 | -0.485288 | 2.512602 |
| H | 9.790131 | -0.601322 | -0.325973 |
| H | 8.273307 | -1.419927 | 0.093441 |
| H | 8.315320 | -0.293360 | -1.281920 |
| C | -0.096751 | -1.756975 | -2.434518 |
| C | -1.269649 | -3.838181 | -3.213862 |
| H | -2.181196 | -2.179620 | -2.239484 |
| H | -0.163175 | -1.357740 | -3.458097 |
| H | -0.109195 | -0.882411 | -1.756172 |
| H | 0.889364 | -2.228935 | -2.308031 |
| H | -1.475324 | -3.398998 | -4.201834 |
| H | -0.308581 | -4.368845 | -3.294007 |
| H | -2.050936 | -4.585247 | -3.007809 |
| C | -1.207799 | -1.908998 | 3.900425 |
| H | -2.765353 | -1.416451 | 2.508227 |
| C | -3.359255 | -3.174176 | 3.569324 |
| H | -1.660983 | -1.325576 | 4.716599 |
| H | -0.720637 | -2.784806 | 4.356412 |
| H | -0.415090 | -1.297800 | 3.444070 |
| H | -3.828663 | -2.604293 | 4.386157 |
| H | -4.153513 | -3.480073 | 2.873093 |
| H | -2.935806 | -4.090248 | 4.009594 |
| C | 1.302616 | 1.847238 | -2.541845 |
| H | -0.824687 | 1.659936 | -2.322842 |

| | | | |
|---|---|---|---|
| C | -0.419229 | 3.468038 | -3.390296 |
| H | -0.408172 | 2.968394 | -4.371412 |
| H | -1.411607 | 3.921250 | -3.255415 |
| H | 0.314825 | 4.287807 | -3.430268 |
| H | 1.312320 | 1.335860 | -3.516505 |
| H | 2.091214 | 2.614746 | -2.570243 |
| H | 1.591199 | 1.113926 | -1.775434 |
| C | -1.692124 | 1.911748 | 3.327078 |
| C | -2.749634 | 4.188819 | 3.195188 |
| H | -3.090996 | 2.569171 | 1.855567 |
| H | -2.450574 | 1.626111 | 4.072121 |
| H | -1.406787 | 0.990643 | 2.789815 |
| H | -0.795393 | 2.255517 | 3.865892 |
| H | -3.595908 | 3.874912 | 3.825087 |
| H | -1.984494 | 4.600838 | 3.870826 |
| H | -3.097702 | 5.005807 | 2.545345 |
| H | -4.978990 | 3.425048 | -2.918917 |
| H | -3.462373 | 2.523970 | -3.131508 |
| H | -4.965016 | 1.658772 | -2.738545 |
| H | -4.141855 | 4.911844 | -1.300662 |
| H | -3.256335 | 4.441513 | 0.155173 |
| H | -2.501844 | 4.267468 | -1.440507 |
| H | -5.990758 | 3.729196 | -0.576503 |
| H | -6.072665 | 1.970593 | -0.424338 |
| H | -5.253422 | 2.932844 | 0.827745 |
| H | -6.124366 | -2.646678 | -2.069666 |
| H | -5.782240 | -0.914393 | -2.141173 |
| H | -4.567641 | -2.083404 | -2.709292 |
| H | -5.317106 | -4.091190 | -0.461750 |
| H | -3.667942 | -3.874642 | -1.061285 |
| H | -4.009321 | -3.740106 | 0.674643 |
| H | -6.672566 | -2.383083 | 0.425812 |
| H | -5.450093 | -1.756465 | 1.552651 |
| H | -6.238235 | -0.661806 | 0.394040 |
| H | 6.486019 | -0.861693 | 1.800951 |
| H | 5.170828 | 0.283688 | 1.622769 |
| H | 6.706148 | 1.750741 | 1.232970 |

## 2. References


[S1] P. H. M. Budzelaar, A. B. van Oort, a G. Orpen, *Eur. J. Inorg. Chem.* **1998**, *1998*, 1485–1494.

[S2] T. X. Gentner, B. Rösch, G. Ballmann, J. Langer, H. Elsen, S. Harder, *Angew. Chem. Int. Ed.* **2019**, *58*, 607–611;*Angew. Chem.* **2019**, *131*, 617–621.

[S3] B. Rösch, T. X. Gentner, J. Eyselein, A. Friedrich, J. Langer, S. Harder, *Chem. Commun.* **2020**, *56*, 11402–11405.

[S4] C. Cui, H. W. Roesky, H. Hao, H.-G. Schmidt, M. Noltemeyer, *Angew. Chem. Int. Ed.* **2000**, *39*, 1815–1817.

[S5] C. E. Radzewich, I. A. Guzei, R. F. Jordan, *J. Am. Chem. Soc.* **1999**, *121*, 8673–8674.

[S6] (a) Rigaku Oxford Diffraction, **2019**, CrysAlisPro Software system, version 1.171.40.67a, Rigaku Corporation, Oxford, UK; (b) Rigaku Oxford Diffraction, **2019**, CrysAlisPro Software system, version 1.171.40.53, Rigaku Corporation, Oxford, UK; (c) Rigaku Oxford Diffraction, **2018**, CrysAlisPro Software system, version 1.171.40.18b, Rigaku Corporation, Oxford, UK; (d) Rigaku Oxford Diffraction, **2018**, CrysAlisPro Software system, version 1.171.39.46, Rigaku Corporation, Oxford, UK.

[S7] O. V. Dolomanov, L. J. Bourhis, R.J. Gildea, J. A. K. Howard and H. Puschmann, *J. Appl. Crystallogr.*, **2009**, *42*, 339–341.

[S8] G. M. Sheldrick, *Acta Cryst. A,* **2015**, *71*, 3–8.

[S9] G. M. Sheldrick, *Acta Cryst. C,* **2015**, *71*, 3–8.

[S10] A. Thorn, B. Dittrich and G. M. Sheldrick, *Acta Cryst. A,* **2012**, *68*, 448–451.

[S11] P. van der Sluis, A. L. Spek, *Acta Cryst. A,* **1990**, *46*,194-201.

[S12] J.-S. Jiang, A. T. Brünger, *J. Mol. Biol.,* **1994**, *243*, 100-115.

[S13] M. J. Frisch, G. W. Trucks, H. B. Schlegel, G. E. Scuseria, M. A. Robb, J. R. Cheeseman, G. Scalmani, V. Barone, G. A. Petersson, H. Nakatsuji, X. Li, M. Caricato, A. V. Marenich, J. Bloino, B. G. Janesko, R. Gomperts, B. Mennucci, H. P. Hratchian, J. V. Ortiz, A. F. Izmaylov, J. L. Sonnenberg, D. Williams-Young, F. Ding, F. Lipparini, F. Egidi, J. Goings, B. Peng, A. Petrone, T. Henderson, D. Ranasinghe, V. G. Zakrzewski, J. Gao, N. Rega, G. Zheng, W. Liang, M. Hada, M. Ehara, K. Toyota, R. Fukuda, J. Hasegawa, M. Ishida, T. Nakajima, Y. Honda, O. Kitao, H. Nakai, T. Vreven, K. Throssell, J. A. Montgomery, J. E. Peralta, F. Ogliaro, M. J. Bearpark, J. J. Heyd, E. N. Brothers, K. N. Kudin, V. N. Staroverov, T. A. Keith, R. Kobayashi, J. Normand, K. Raghavachari, A. P. Rendell, J. C. Burant, S. S. Iyengar, J. Tomasi, M. Cossi, J. M. Millam, M. Klene, C. Adamo, R. Cammi, J. W. Ochterski, R. L. Martin, K. Morokuma, O. Farkas, J. B. Foresman and D. J. Fox, Gaussian 16 Rev. A.03, Wallingford CT, **2016**.

[S14] A. D. Becke, *J. Chem. Phys.*, **1993,** *98,* 5648-5652; C. Lee, W. Yang and R. G. Parr, *Phys. Rev.B*, **1988**, 37, 785-789; F. Weigend and R. Ahlrichs, *Phys. Chem. Chem. Phys.* **2005**, *7,* 3297-305; F. Weigend, *Phys. Chem. Chem. Phys.*, **2006**, *8* 1057-1065.

[S15] A. V. Marenich, C. J. Cramer and D. G. Truhlar, *J. Phys. Chem. B* **2009**, *113*, 6378–6396.

[S16] N. van Eikema Hommes, Molecule, Erlangen, **2018**.


## Author Contributions

A.F. conducted the experimental work. DFT calculations were done by J.E. and H.E; crystal structure determination was done by J.P, M.W. and J.L. The results were reported by S.H. and A.F. whereas S.H. was additionally responsible for supervision.
